# Preparation of Functionalized Amides Using Dicarbamoylzincs

**DOI:** 10.1002/anie.202205440

**Published:** 2022-06-13

**Authors:** Dimitrije Djukanovic, Maximilian A. Ganiek, Kohei Nishi, Konstantin Karaghiosoff, Kazushi Mashima, Paul Knochel

**Affiliations:** ^1^ Department Chemie Ludwig-Maximilians-Universität München Butenandtstraße 5–13, Haus F 81377 München Germany; ^2^ Patheon, by Thermo Fisher Scientific Patheon Regensburg Gmbh Donaustaufer Straße 378 93055 Regensburg Germany; ^3^ Graduate School of Engineering Science Osaka University 1-3 Machikaneyama Toyonaka 565-0871 Osaka Japan; ^4^ Graduate School of Pharmaceutical Sciences Osaka University 1-6 Yamadaoka Suita 565-0871 Osaka Japan

**Keywords:** Amides, Carbamoyl, Lithium, Metalation, Zinc

## Abstract

We report a new convenient preparation of dicarbamoylzincs of type (R^1^R^2^NCO)_2_Zn by the treatment of ZnCl_2_ and formamides R^1^R^2^NCHO with LiTMP in THF (15 °C, 15 min) or by the reaction of formamides R^1^R^2^NCHO with TMP_2_Zn (25 °C, 16 h). This second method tolerates sensitive groups such as an ester, ketone or nitro function. Reaction of these dicarbamoylzincs with allylic, benzylic, aryl, alkenyl bromides, acid chlorides, aldehydes or enones provided various polyfunctional amides in 47–97 % yields. ^13^C NMR characterization of these new carbamoylzinc derivatives is reported.

## Graphical abstract

Polyfunctional dicarbamoylzincs of type (R^1^R^2^NCO)_2_Zn were prepared in THF by treating a mixture of formamides and ZnCl_2_ with LiTMP (THF, 15 °C, 15 min) or by reacting formamides with TMP_2_Zn (THF, 25 °C, 16 h). These zinc reagents reacted with allylic, benzylic, aryl and alkenyl bromides, acid chlorides, aldehydes or enones providing various amides in 47–97 % yields. ^13^C NMR data showed a characteristic carbonyl signal at 219.4 ppm for (Bu_2_NCO)_2_Zn.

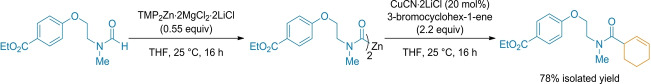

Reagents displaying an umpolung of reactivity have attracted much attention.^[1]^ Especially, acyl anion equivalents have found many synthetic applications.^[2]^ Also, related carbamoyl organometallics of type **1** have been prepared either by reduction of the corresponding carbamoyl chloride **2** by lithium metal (pathway A),^[3]^ by the insertion of CO to copper or lithium amides of type **3** (pathway B)^[4]^ or by the metalation of various formamides **4** with lithium bases such as LDA or *t*‐BuLi at low temperature (Scheme 1).[^5^, ^6^] Recently, Reeves used carbamoyllithiums prepared in toluene by lithiation with LDA for the addition to *N*‐sulfonyl imines producing α‐amino acids.^[7]^ All these methods suffer from drawbacks such as a limited functional group compatibility, the use of a toxic gas or cryogenic reaction temperatures. Recently, we have reported that lithium amides like LiTMP (TMP=2,2,6,6‐tetramethylpiperidyl) were compatible with metallic salts such as ZnCl_2_⋅2 LiCl, MgCl_2_ and CuCN⋅2 LiCl at low temperature.^[8]^ The stability of such Lewis pairs, which may be considered as frustrated Lewis pairs,^[9]^ allowed in situ trapping metalations of various arenes and heteroarenes.^[8]^ This in situ protocol was expanded by generating carbamoyllithiums of type **1 a** in the presence of various electrophiles in continuous flow.^[10]^ The Barbier procedure was essential for the success of the reaction conducted in continuous flow and allowed to prepare a wide range of products of type **5**. Although this reaction represented a synthetic advance, it did not allow the performance of cross‐couplings with aryl and heteroaryl halides and required a flow apparatus. Catalytic aminocarbonylation protocols^[11]^ involve usually highly toxic CO gas, an amine and an aryl halide. Those performed in the absence of CO gas are scarce.[^6e^, ^6f^, ^12^] Herein, we have reported the synthesis of a new room temperature stable dicarbamoylzinc species **6** (stable at least 16 h at 25 °C)^[13]^ using two complementary methods (Method A and B) and their reactions with a range of electrophiles such as allylic and benzylic bromides, aldehydes, acid chlorides, enones and heteroaryl or alkenyl bromides producing functionalized amides of type **7**.

**Scheme 1:**
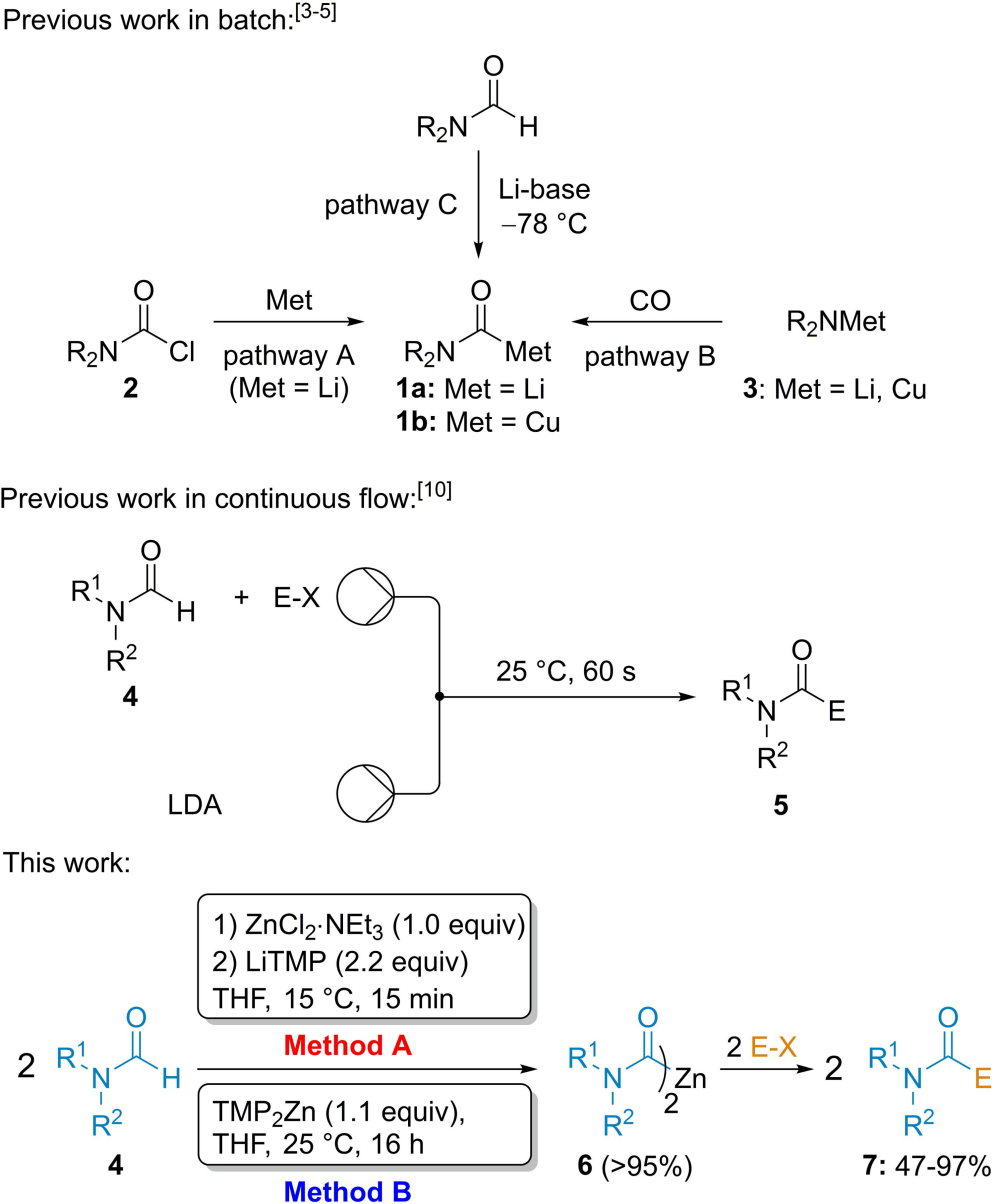
Preparations of carbamoylmetal reagents.

Thus, in preliminary experiments we have treated a THF mixture of formamides of type **4** (1.0 equiv) and ZnCl_2_ (0.5 equiv) in the presence (or absence) of Et_3_N (0.5 equiv)^[14]^ with various lithium amide bases such as LDA, Cy_2_NLi (Cy=cyclohexyl)^[15]^ and LiTMP in order to prepare the dicarbamoylzinc species **6** at temperatures between 0–25 °C for 15 min. The conversion to the zinc reagent **6** was evaluated by performing copper‐catalyzed allylations with allyl bromide on reaction aliquots.^[16]^ These experiments showed that LiTMP (2.2 equiv; 0.5 M in THF) was the best base for achieving this lithiation (performed in the presence of ZnCl_2_) providing the dicarbamoylzinc **6**.^[17]^

With these conditions in hand, we have examined the reaction scope. Thus, *N,N*‐dibutylformamide (**4 a**) was converted to the dicarbamoylzinc **6 a** (LiTMP, 2.2 equiv; ZnCl_2_⋅NEt_3_, 1.0 equiv; 15 °C, 15 min). We have isolated, after a copper‐catalyzed allylation with allyl bromide, the expected amide **8 a** in 94 % isolated yield (both carbamoyl moieties were reacting). Various formamides (**4 b**–**4 h**) were zincated by this procedure leading to **6 b**–**j**, which provided the desired allylated products **8 b**–**8 j** in 57–97 % yield (Scheme 2). Interestingly, although copper‐zinc cuprates of type RCu(CN)ZnX^[18]^ gave usually S_N_2′‐substitution allylation products, we have observed the formation of only S_N_2‐substitution allylation products using prenyl bromide (**8 h** and **8 i**; 61–62 % yield) or cinammyl bromide (**8 j**: 57 % yield, S_N_2/S_N_2′>9 : 1).^[19]^ This unusual regioselectivity may be due to the carbonyl group coordination to the copper center resulting in a different Zn/Cu‐cluster. In contrast, with propargyl bromide, we have obtained only the S_N_2′ product, i.e. the allenic amide **8 k** (58 % yield). Interestingly, we have also used this method for the preparation of ^13^C‐labeled amide **8 l** from Bu_2_N^13^CHO.^[20]^

**Scheme 2:**
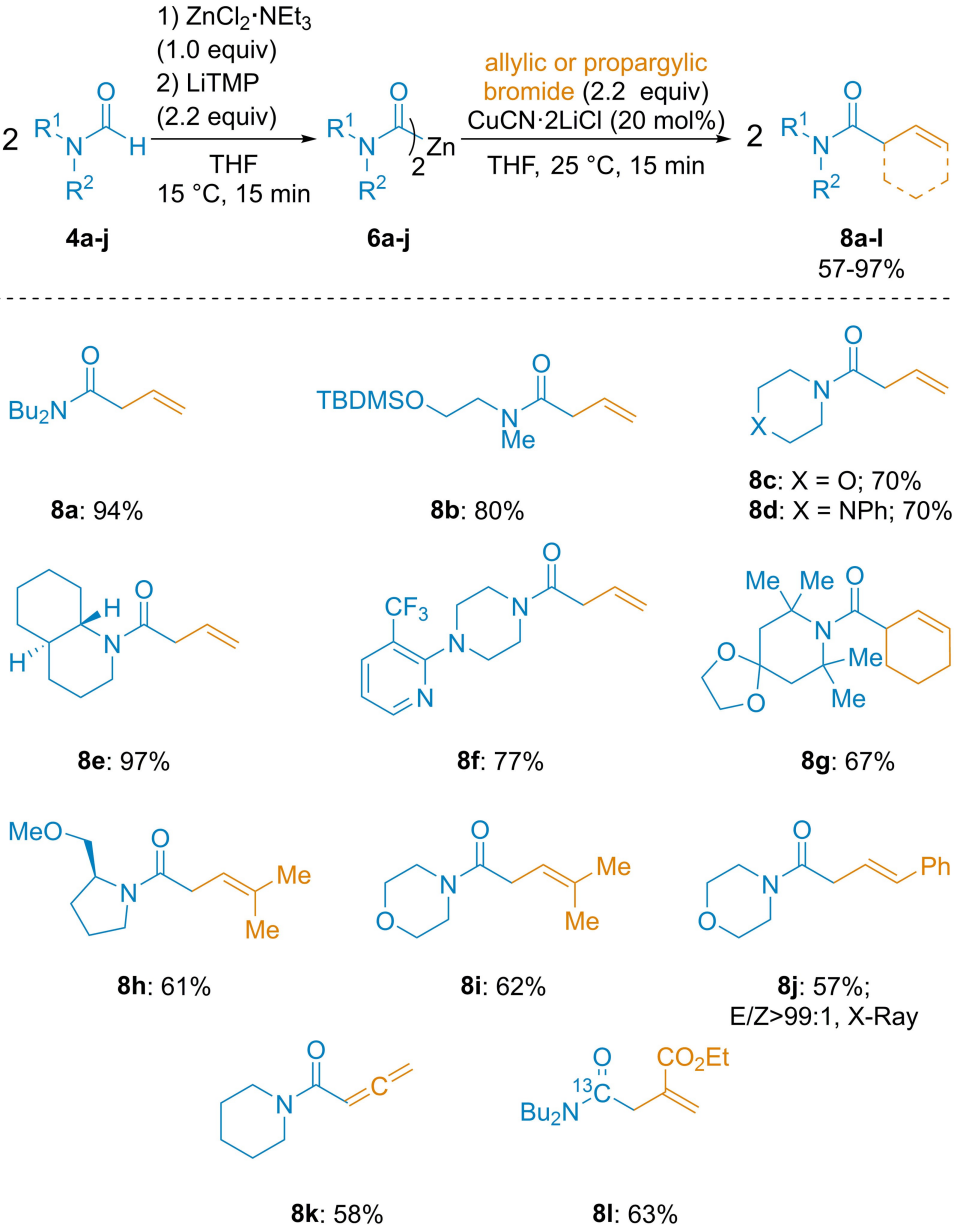
Allylation of dicarbamoylzincs of type **6** with allylic and propargylic bromides providing polyfunctional amides of type **8**. The indicated yields refer to analytically pure isolated product.

In order to tolerate more sensitive groups such as an ester, ketone or a nitro function, we have directly treated several formamides (**4 k**–**o**) with TMP_2_Zn^[21]^ in THF at 25 °C for 16 h (Method B) affording the desired zinc reagents **6 k**–**o** which after allylation gave the desired polyfunctional products **8 m**–**q** containing an ester, a ketone, an imide and a nitro group (Scheme 3).

**Scheme 3:**
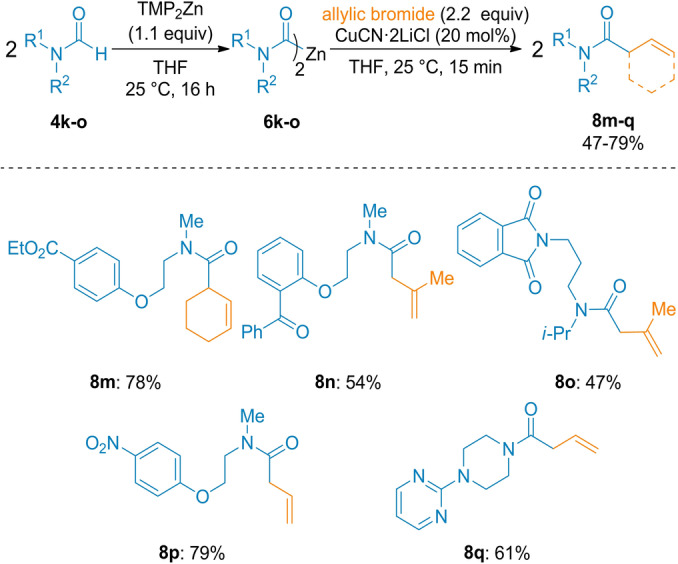
Allylation of dicarbamoylzincs of type **6** with allylic bromides providing polyfunctional amides of type **8**. The indicated yields refer to analytically pure isolated product.

Dicarbamoylzincs of type **6** also underwent smooth benzylations with various benzylic bromides in the presence of MgCl_2_⋅LiCl (1.0 equiv) affording polyfunctional arylacetamide derivatives (**9 a**–**9 i**) in 57–88 % yield (Scheme 4). In the absence of MgCl_2_⋅LiCl, a homo‐coupling product of benzylic bromide (1,2‐diarylethane) was observed. The positive effect of MgCl_2_ was also mandatory for performing addition reactions to aldehydes.^[22]^ Thus, the reaction of **6 a** and **6 e** with benzaldehydes in the presence of MgCl_2_⋅LiCl (1.0 equiv) gave the expected α‐hydroxyamides (**10 a, b**) in 57–74 % yield (Scheme 5).^[23]^

**Scheme 4:**
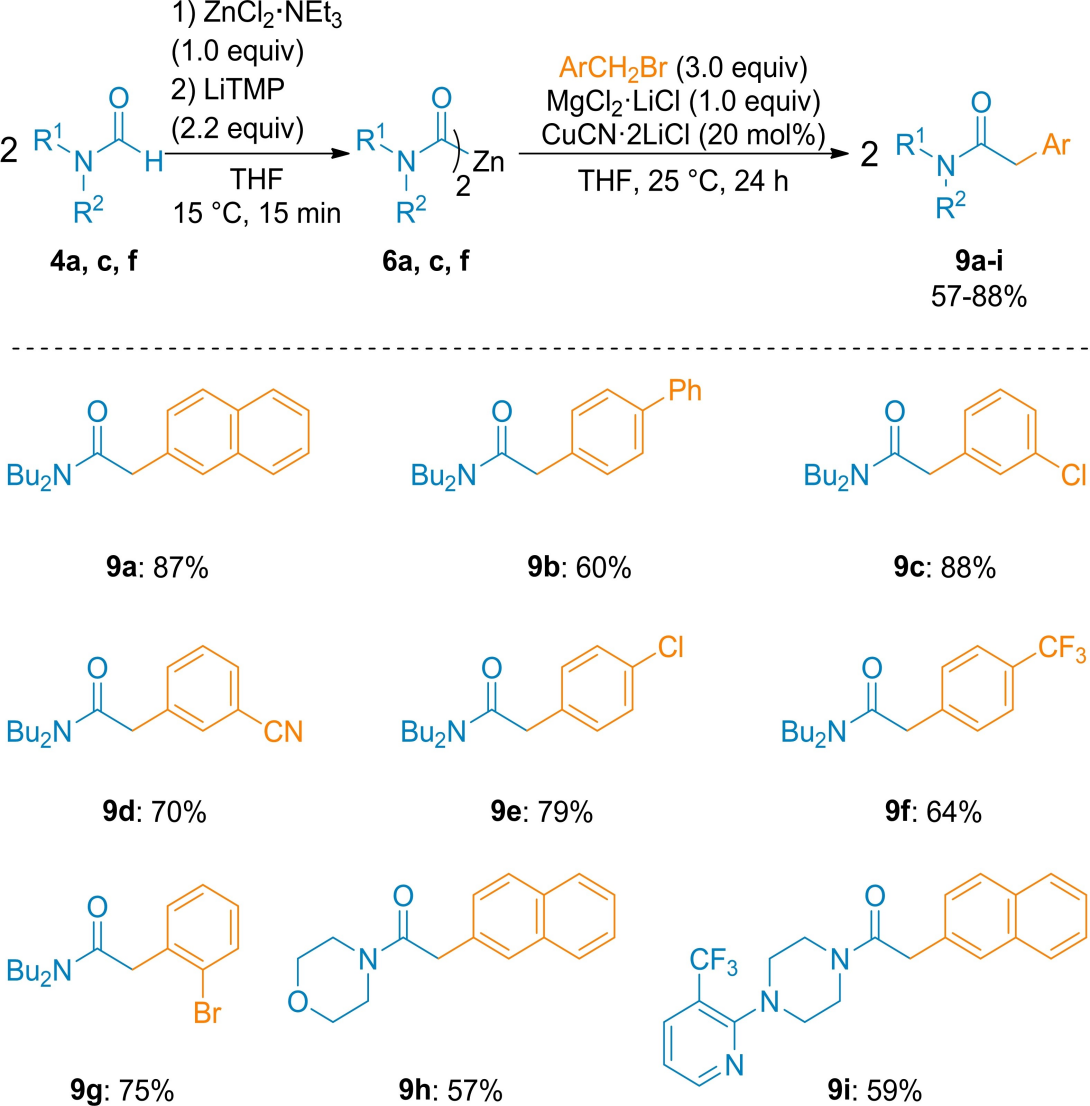
Cu‐catalyzed benzylation of dicarbamoylzincs **6** with benzylic bromides. The indicated yields refer to analytically pure isolated product.

**Scheme 5:**
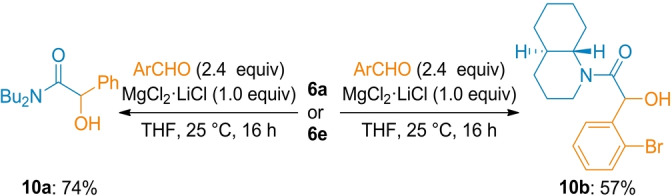
Mg‐mediated hydroxyalkylation of dicarbamoylzincs **6** with aldehydes. The indicated yields refer to analytically pure isolated product.

Acylation with various acid chlorides were performed in the absence of any catalyst and a complete acylation of various dicarbamoylzinc reagents of type **6** with acid chlorides at 25 °C, 16 h resulting in the formation of α‐ketoamides (**11 a**–**11 d**) in 54–84 % yield (Scheme 6). In the reaction of **6 a** with diphenylphosphinic chloride *N*,*N*‐dibutyl‐1‐(diphenylphosphoryl)formamide **11 e** was produced in 70 % yield.

**Scheme 6:**
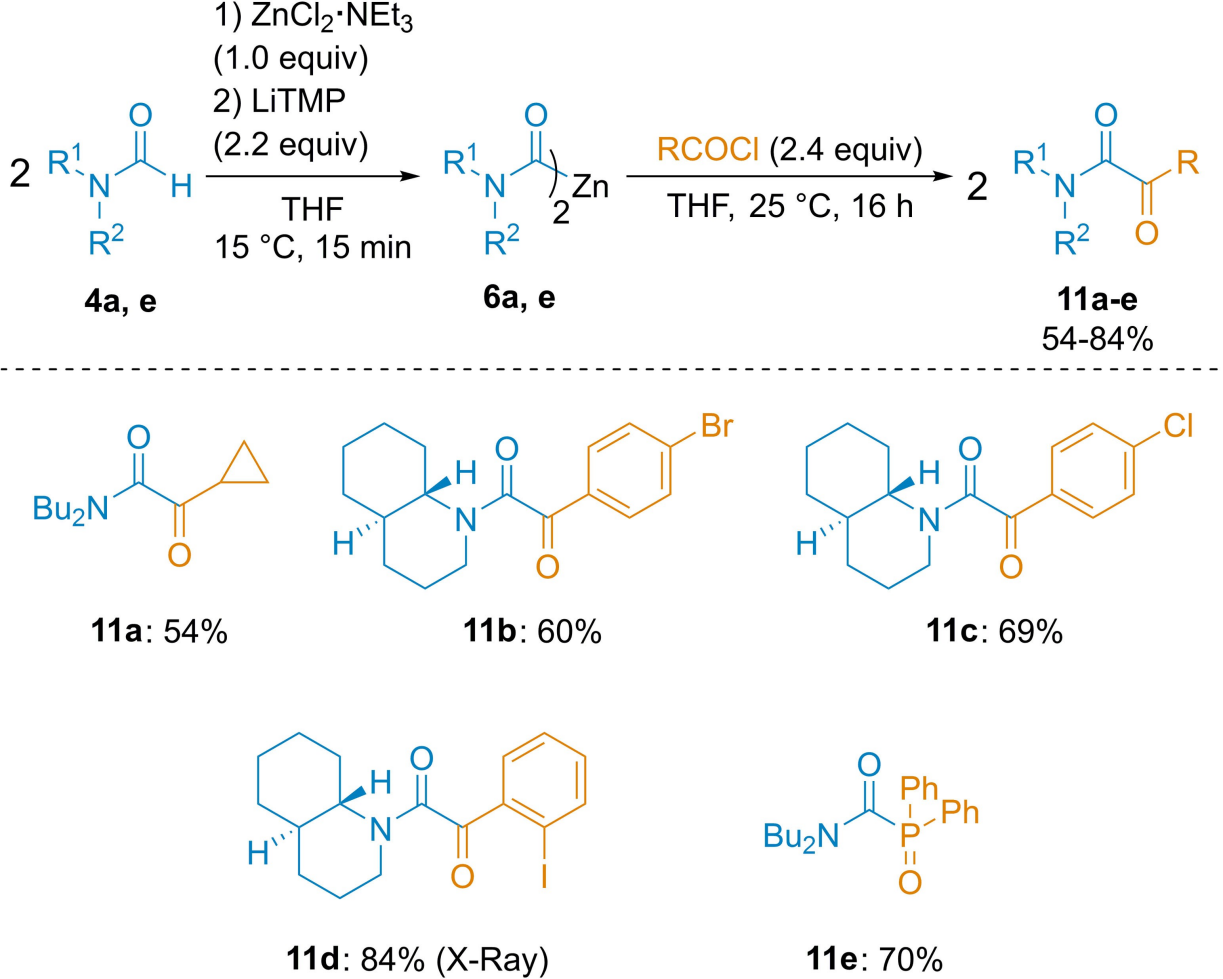
Acylation of dicarbamoylzincs **6** with acid chlorides. The indicated yields refer to analytically pure isolated product.

Interestingly, a 1,4‐addition was achieved starting with 2‐cyclohexen‐1‐one and amide **4 a**. Thus, the corresponding zinc reagent **6 a** was cooled to −78 °C and treated with CuCN⋅2 LiCl (1.0 equiv) for 0.5 h followed by BF_3_⋅OEt_2_ (1.0 equiv)^[24]^ and cyclohexenone (1.0 equiv) to give after 16 h at −78 °C the Michael adduct **12** in 54 % isolated yield (Scheme 7).

**Scheme 7:**
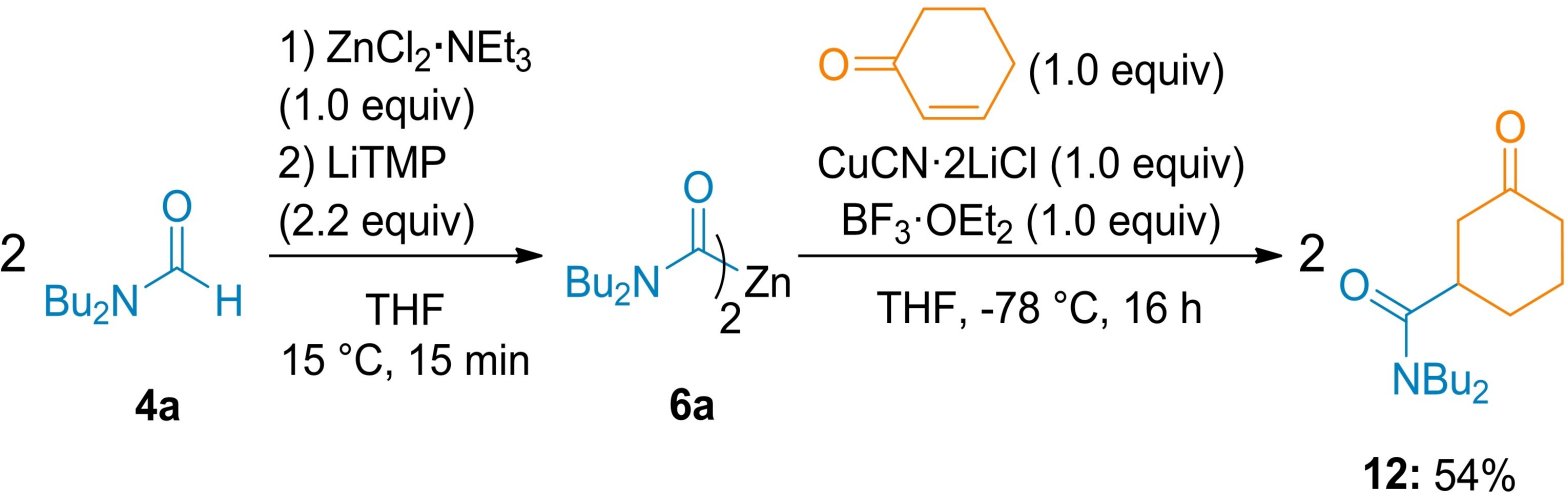
Cu‐mediated 1,4‐addition of **6 a** to 2‐cyclohexen‐1‐one in the presence of BF_3_⋅OEt_2_. The indicated yields refer to analytically pure isolated product.

We have examined cross‐coupling reactions with various functionalized aryl bromides and noticed that a dual‐catalysis^[25]^ involving a copper catalyst (4 mol % CuCN⋅2 LiCl) and a palladium catalyst (10 mol % Pd(dppf)Cl_2_) (dppf=1,1′‐bis(diphenylphosphino)ferrocene) was required. Using only Pd(dppf)Cl_2_ or a CuCN⋅2 LiCl gave almost no product. In a typical experiment, we have prepared **6 a** from *N,N*‐dibutylformamide (2.0 equiv) with the usual procedure (Method A). Addition of 10 mol % Pd(dppf)Cl_2_, aryl/alkenyl bromide and 4 mol % CuCN⋅2 LiCl gave after heating the reaction mixture for 16 h at 45 °C in a sealed tube the desired cross‐coupling products **13 a**–**13 q** in 53–93 % isolated yield (Scheme 8). Scale‐up of this procedure has been demonstrated in the preparation of **13 k** (10 mmol scale; Scheme 8) with reduction of catalyst loading (2 mol % Pd(dppf)Cl_2_, 0.8 mol % CuCN⋅2 LiCl).

**Scheme 8:**
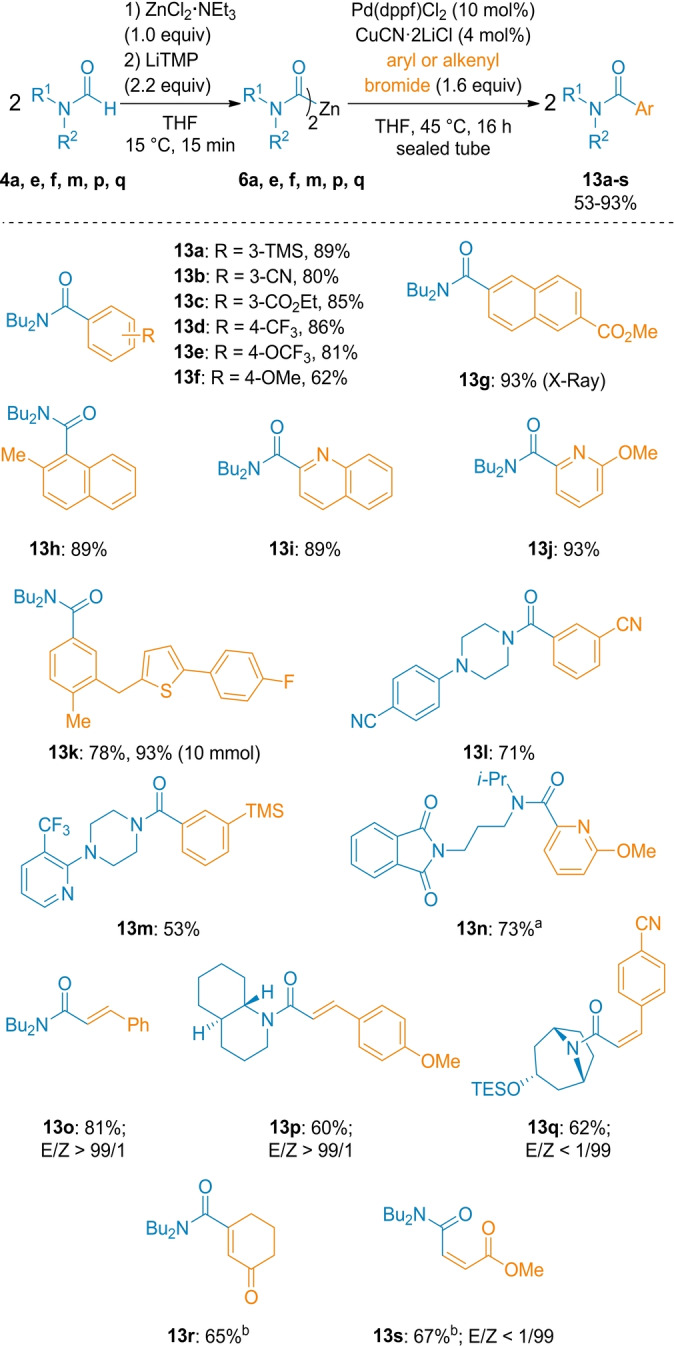
Pd‐ and Cu‐dual catalyzed cross‐couplings of dicarbamoylzincs **6** with aryl and alkenyl bromides. The indicated yields refer to analytically pure isolated product. [a] Metalation performed with TMP_2_Zn⋅2 MgCl_2_⋅2 LiCl. [b] Reaction performed from alkenyl iodides using CuCN⋅2 LiCl (1.0 equiv) without [Pd] catalyst.

A ^13^C NMR‐characterisation of *N*,*N*‐dibutylcarbamoylzinc reagent was done. Thus, the ^13^C NMR spectra of the reaction mixture obtained by treating **4 a**/ZnCl_2_ mixture with LiTMP showed a new characteristic carbonyl signal (δ=219.4 ppm), together with a broad signal around δ=225 ppm (Figure 1a). To confirm the assigment of these resonances, we have prepared dicarbamoylzinc **6 a** by an alternative method. Thus, treatment of Bu_2_NLi at −78 °C with CO gas led to *N*,*N*‐dibutylcarbamoyllithium[^4d^, ^4e^] (1.0 equiv) which was transmetalated under CO atmosphere with ZnCl_2_ (0.5 equiv) to give the dicarbamoylzinc reagent **6 a**. Indeed, an identical ^13^C NMR signal with a chemical shift for the carbonyl group δ=219.4 ppm (Figure 1b) was observed. Also, by using 0.3 equiv of ZnCl_2_ we obtained the zincate **14 a** (Figure 1c). Finally, TMP_2_Zn⋅2 LiCl as a metalation reagent afforded spectroscopically pure diorganozinc reagent **6 a** (Figure 1d).

**Figure 1:**
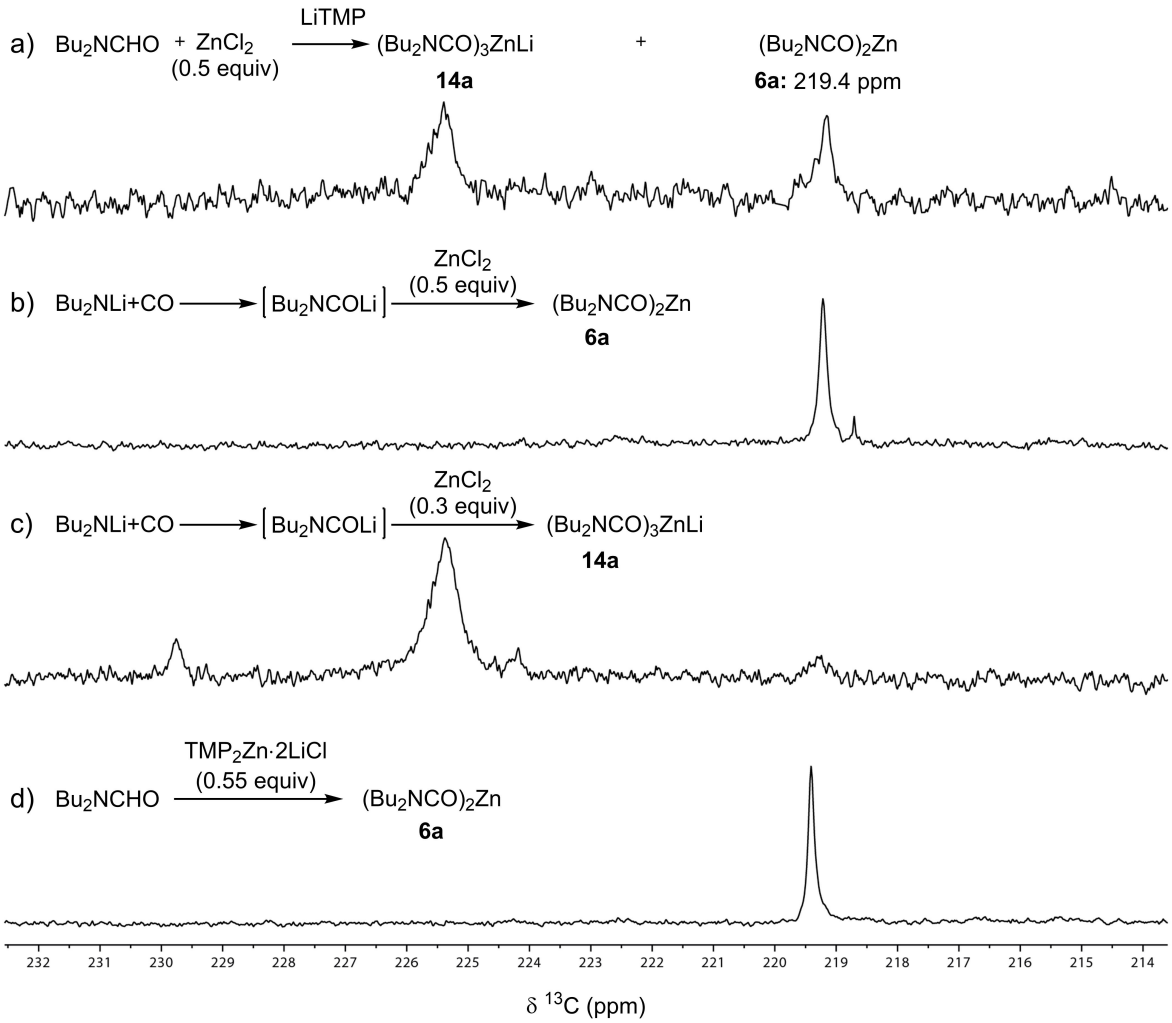
^13^C NMR spectra of dicarbamoylzinc **6 a** and lithium tricarbamoylzincate **14 a** generated via different methods.

In summary, we have reported a new convenient in situ lithiation with LiTMP of various formamides **4** in the presence of ZnCl_2_ providing new dicarbamoylzincs **6** which underwent allylations, benzylations, arylations, alkenylations, acylations, hydroxyalkylations and 1,4‐additions providing polyfunctional amides in good yields (Method A). Alternatively, we have also demonstrated that the reaction of polyfunctional formamides with TMP_2_Zn provides dicarbamoylzincs containing sensitive functions such as ester, ketone or nitro (Method B). ^13^C NMR investigations confirmed the formation of (R_2_NCO)_2_Zn and related aggregate (R_2_NCO)_3_ZnLi under these reaction conditions.

## Conflict of interest

The authors declare no conflict of interest.

## Supporting information

As a service to our authors and readers, this journal provides supporting information supplied by the authors. Such materials are peer reviewed and may be re‐organized for online delivery, but are not copy‐edited or typeset. Technical support issues arising from supporting information (other than missing files) should be addressed to the authors.

Supporting Information

Supporting Information

Supporting Information

Supporting Information

Supporting Information

Supporting Information

## Data Availability

The data that support the findings of this study are available in the Supporting Information of this article.
