# Supplementary material for: Preparation of Functionalized Amides Using Dicarbamoylzincs
Source: Angew Chem Int Ed Engl. 2022 Jun 13;61(31):e202205440. doi: 10.1002/anie.202205440 (PMC9401601; doi:10.1002/anie.202205440)
Supplement: Supplementary file 6 — Supporting Information [file ANIE-61-0-s005.pdf]

Angewandte Chemie
Eine Zeitschrift der Gesellschaft Deutscher Chemiker

Supporting Information

Preparation of Functionalized Amides Using Dicarbamoylzincs

*D. Djukanovic, M. A. Ganiek, K. Nishi, K. Karaghiosoff, K. Mashima, P. Knochel**

Preparation of Functionalized Amides using Dicarbamoylzincs

Dimitrije Djukanovic, Maximilian A. Ganiek, Kohei Nishi, Konstantin Karaghiosoff, Kazushi Mashima, Paul Knochel

Department Chemie, Ludwig-Maximilians-Universität München
Butenandtstr. 5-13, 81377 München, Germany
Paul.Knochel@cup.uni-muenchen.de

Supporting Information

Table of Contents

General Information 2
Reagents 2
Titration of organometallic reagents 3
Chromatography 3
Analytical Data 3
Optimization and screening 6
Optimization of metalation conditions 6
Stability study of dicarbamoylzinc 6a 7
Optimization of the benzylation quench 7
Optimization of the cross-coupling reaction 8
Typical Procedures 9
Preparation of starting materials 12
Preparation of products 21
NMR spectra of starting materials 50
NMR spectra of products 66
NMR characterization of carbamoylzinc reagents 127
Single Crystal X-Ray Diffraction Studies 134
References 154

General Information

All reactions were carried out under argon or nitrogen atmosphere in glassware dried with a heat gun (650 °C) under high vacuum (<1 mbar). Syringes which were used to transfer anhydrous solvents or reagents were purged thrice with argon or nitrogen prior to use. Indicated yields are isolated yields of compounds estimated to be >95% pure as determined by ^{1}H-NMR (25 °C) and capillary GC. Unless otherwise indicated, all reagents were obtained from commercial sources.

Solvents

Solvents were dried according to standard procedures by distillation over drying agents and stored under argon.

THF was continuously refluxed and freshly distilled from sodium benzophenone ketyl under nitrogen.

Solvents for column chromatography were distilled on a rotary evaporator prior to use.

Reagents

***i*PrMgCl·LiCl:** Magnesium turnings (2.67 g, 110 mmol) and anhydrous LiCl (4.66 g, 110 mmol) were placed in an argon-flushed flask and THF (50 mL) was added. A solution of *i*PrCl (9.13 mL, 100 mmol) in THF (50 mL) was slowly added at 25 °C. The reaction begins within a few minutes. After addition, the reaction mixture was stirred for 12 h at 25 °C. The grey solution of *i*PrMgCl·LiCl was cannulated to another flask under argon and removed in this way from excess of magnesium. A yield of ca. 95-98% of *i*PrMgCl·LiCl is obtained. [1]

CuCN·2LiCl solution (1.00 M) was prepared by drying CuCN (80.0 mmol, 7.17 g) and LiCl (160 mmol, 6.78 g) in a Schlenk-flask under vacuum at 140 °C for 5 h. After cooling, dry THF (80 mL) was added and stirring continued until the salts were dissolved. [2]

$ZnCl_2$ solution (1.00 M) was prepared by drying $ZnCl_2$ (200 mmol, 27.3 g) in a Schlenk-flask under vacuum at 140 °C for 5 h. After cooling, dry THF (200 mL) was added and stirring continued until the salt was dissolved.

TMPH (2,2,6,6-tetramethylpiperidine) was distilled under atmospheric pressure (bp. 152 °C) to obtain a yellow liquid. Drying and distillation over CaH_2 under reduced pressure (65 mbar) afforded near-colorless liquid. It was stored under argon in a bottle sealed with septum.

TMPMgCl·LiCl: A dry and argon flushed 250 mL flask, equipped with a magnetic stirrer and a septum,was charged with freshly titrated *i*-PrMgCl·LiCl (100 mL, 1.2 M in THF, 120 mmol). TMPH (19.8 g, 126 mmol, 1.05 equiv) was dropwise added at room temperature. The reaction mixture was stirred at 25 °C until gas evolution was completed (ca. 24 h). Titration of the base with benzoic acid using 4-(phenylazo)diphenylamine as an indicator afforded ca. 1M solution of TMPMgCl·LiCl. [3]

---

[1] A. Krasovskiy, P. Knochel, *Angew. Chem. Int. Ed. Engl.* **2004**, *43*, 3333.
[2] P. Knochel, M. C. P. Yeh, S. C. Berk, J. Talbert, *J. Org. Chem.* **1988**, *53*, 2390.
[3] A. Krasovskiy, V. Krasovskaya, P. Knochel, *Angew. Chem. Int. Ed.* **2006**, *45*, 2958-2961.

TMPLi: Tetramethylpiperidine (169 mg, 1.2 mmol) was dissolved in THF (2 mL) and cooled to –40 °C. Then, *n*-BuLi 1.75 M in hexane (0.7 mL, 1.2 mmol) was dropwise added and the reaction allowed to warm to 0 °C over 1 h. [4]

$TMP_2Zn \cdot 2MgCl_2 \cdot 2LiCl$: Freshly titrated TMPMgCl·LiCl (100 mmol, 1.0 equiv) was dropwise added to a solution of $ZnCl_2$ in THF (1.0 M, 50 mL, 50 mmol, 0.5 equiv). The resulting mixture was protected from light using aluminium foil and stirred at r.t. for 24 h before titrating the base with benzoic acid using 4-(phenylazo)diphenylamine as an indicator. [5]

$MgCl_2 \cdot LiCl$: A dry and argon flushed Schlenk-flask, equipped with a magnetic stirrer and a septum was charged with Mg turnings (2.55 g, 105 mmol), anhydrous LiCl (4.45 g, 105 mmol) and THF (200mL). 1,2-Dichloroethane (9.90 g, 100 mmol) was dropwise added over 1h. The reaction mixture was stirred at 25 °C until gas evolution was complete.

$BF_3 \cdot Et_2O$ was distilled and stored in fridge (5-8 °C) under argon.

Titration of organometallic reagents

n-BuLi was titrated with isopropanol and 1,10-phenanthroline as indicator in THF.

*i*PrMgCl·LiCl was titrated with I_2 in THF. [6]

TMPMgCl·LiCl and $TMP_2Zn \cdot 2MgCl_2 \cdot 2LiCl$ were titrated with benzoic acid and 4-(phenylazo)diphenylamine as indicator in THF. [3,5]

Chromatography

Flash column chromatography was performed using silica gel 60 (0.040-0.063 mm) from MERCK.
Thin layer chromatography was performed using SiO_2 pre-coated aluminum plates (Merck 60, F-254). The chromatograms were examined under 254 nm UV irradiation and/or by staining the TLC plate with a $KMnO_4$ solution followed by heating with a heat gun.
$KMnO_4$ solution: $KMnO_4$ (1.5 g), K_2CO_3 (10 g) and 1.25 mL 10% NaOH in water (200 mL).

Analytical Data

^{1}H-NMR and **^{13}C-NMR** spectra were recorded on VARIAN Mercury 200, BRUKER ARX 300, VARIAN VXR 400 S and BRUKER AMX 600 instruments. Chemical shifts are reported as values in ppm relative to tetramethylsilane. $CDCl_3$ peaks were set to 7.26 ppm in ^{1}H NMR and 77.16 ppm in ^{13}C NMR experiments. The following abbreviations were used to characterize signal multiplicities: s (singlet), d (doublet), dd (doublet of doublets), t (triplet), q (quartet), hept (heptet) as well as m (multiplet).

Mass spectroscopy: High resolution (HRMS) and low resolution (MS) spectra were recorded on a FINNIGAN MAT 95Q instrument. Electron impact ionization (EI) was conducted with an ionization energy of 70 eV. For coupled gas chromatography/mass spectrometry, a HEWLETT-PACKARD HP 6890/MSD 5973 GC/MS system was used. Molecular fragments are reported starting at a relative intensity of 10.

---

[4] R. A. Olofson, C. M. Dougherty, *J. Am. Chem. Soc.* **1973**, *95*, 582-584; M. Campbell, V. Snieckus, E. W. Baxter *Encyclopedia of Reagents for Organic Synthesis*, Wiley, **2001**.
[5] S. H. Wunderlich, P. Knochel, *Angew. Chem. Int. Ed.* **2007**, *46*, 7685.
[6] P. Knochel, A. Krasovskiy, *Synthesis* **2006**, *5*, 890.

Infrared spectra (IR) were recorded from 4500 cm-1 to 650 cm-1 on a PERKIN ELMER Spectrum BX-59343 instrument. For detection a SMITHS DETECTION DuraSample*IR* II Diamond ATR sensor was used. The main absorption peaks are reported in cm-1.

Melting points (m.p.) were determined on a BÜCHI B-540 melting point apparatus and are uncorrected.
List of formamides of type **4** used in Schemes 2-8:

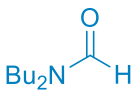

4a

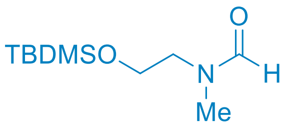

4b

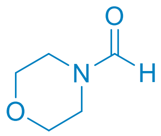

4c

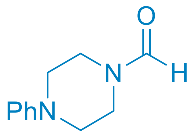

4d

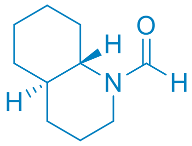

4e

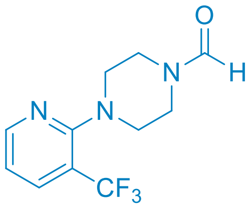

4f

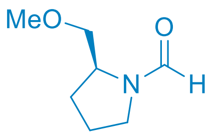

4g

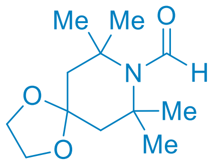

4h

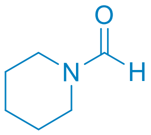

4i

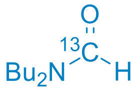

4j

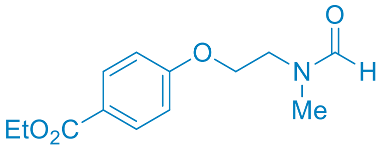

4k

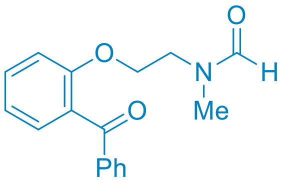

4l

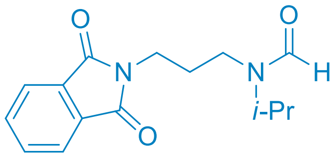

4m

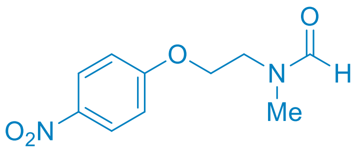

4n

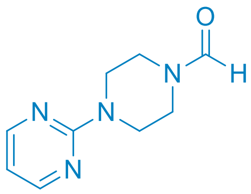

4o

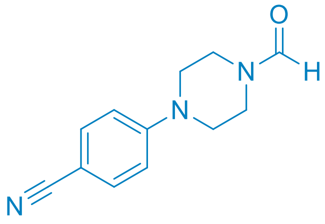

4p

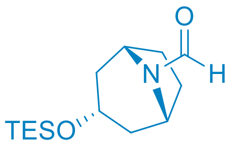

4q

List of dicarbamoylzincs of type **6** used in Schemes 2-8:

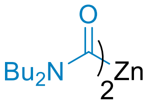

6a

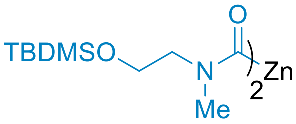

6b

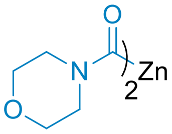

6c

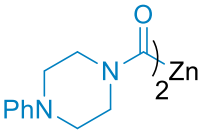

6d

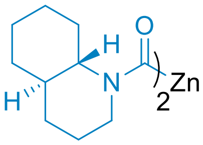

6e

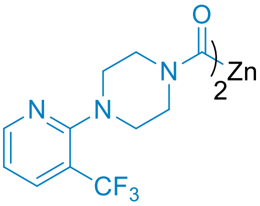

6f

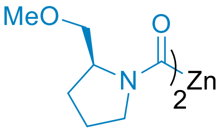

6g

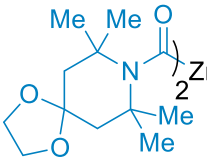

6h

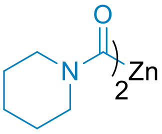

6i

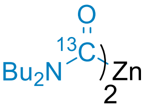

6j

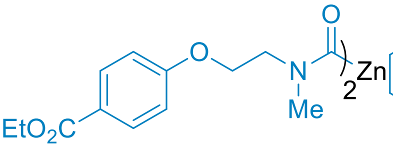

6k

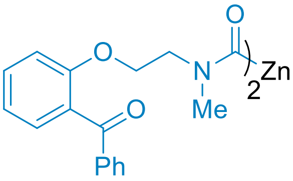

6l

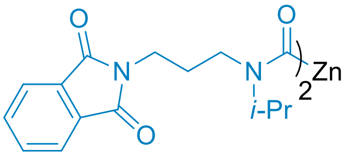

6m

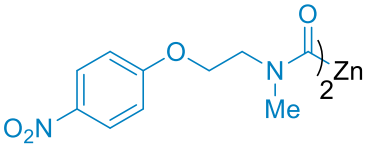

6n

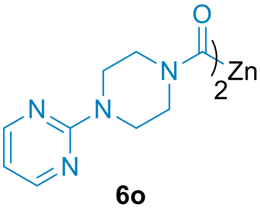

6o

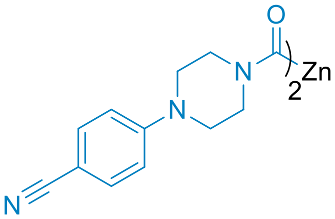

6p

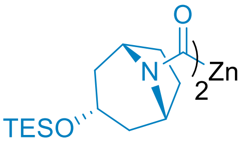

6q

Optimization and screening

Optimization of metalation conditions

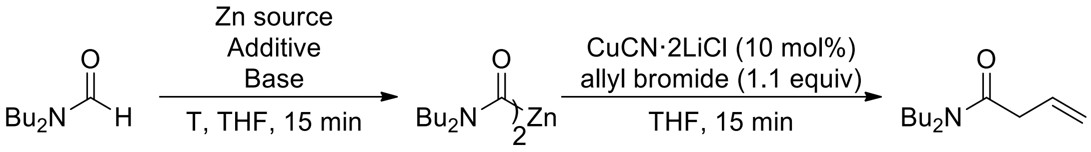

Entry	T [°C]	Base	Zinc source	Additive	Conversion [%]	GC yield [%]
1	15	LDA (1.1 equiv)	$ZnCl_2$ (0.5 equiv)	/	85	70
2	15	Cy_2NLi (1.1 equiv)	$ZnCl_2$ (0.5 equiv)	/	86	71
3	15	TMPLi (1.1 equiv)	$ZnCl_2$ (1.0 equiv)	/	72	53
4	15	TMPLi (1.1 equiv)	$ZnCl_2$ (0.5 equiv)	/	99	97
5	15	TMPLi (1.1 equiv)	$ZnCl_2$ (0.33 equiv)	/	>99	97
6	15	TMPLi (1.1 equiv)	$ZnCl_2$ (0.5 equiv)	Et_3N (0.5 equiv)	>99	98 (94)
6	25	TMPLi (1.1 equiv)	$ZnCl_2$ (0.5 equiv)	Et_3N (0.5 equiv)	>99	90
7	0	TMPLi (1.1 equiv)	$ZnCl_2$ (0.5 equiv)	Et_3N (0.5 equiv)	>99	98
8	15	TMPLi (1.1 equiv)	TMPZnCl·LiCl (0.5 equiv)	/	>99	40
9	15	TMPLi (1.1 equiv)	$TMP_2Zn\cdot 2LiCl$ (0.5 equiv)	/	78	<5
10	15	TMPLi (1.5 equiv)	$ZnCl_2$ (0.5 equiv)	/	>99	75
11	15	$TMP_2Zn\cdot 2LiCl$ (0.5 equiv)	/	/	39	<5

In all reaction where excess base was used (entries 8, 9, 10), double allylation side product was observed.

Stability study of dicarbamoylzinc 6a

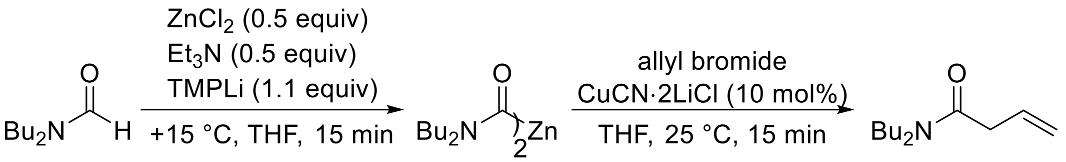

Entry	Time [h]	4a/Standard ration	GC yield [%]*
1	0	4.41	100
2	12	4.41	100
3	24	4.36	98.9
4	48	4.30	97.5

*Entry at t = 0 h was calibrated to 100%.

From our experience in the reactions with various electrophiles and based on the results of these stability studies we suggest using the reagent within 16 h.

Optimization of the benzylation quench

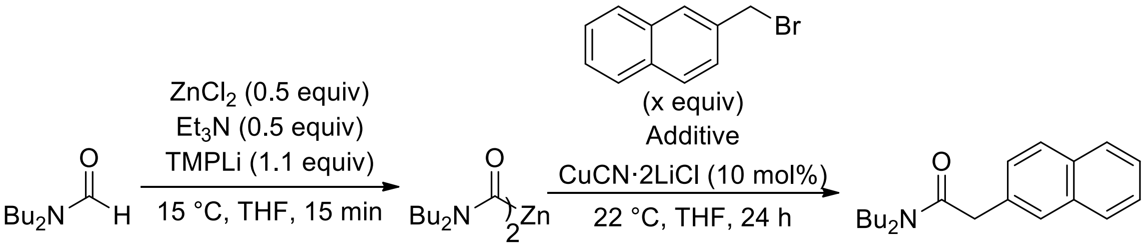

Entry	Additive	Equiv	Conversion [%]	Yield [%]
1	/	1.5	73	60
2	$MgCl_2$·LiCl	1.2	>99	62
3	$MgCl_2$·LiCl	1.5	>99	87 (87)
4	$MgCl_2$·LiCl	2.0	>99	86
5*	$MgCl_2$·LiCl	1.5	>99	87

***Without Et_3N**

Optimization of the cross-coupling reaction

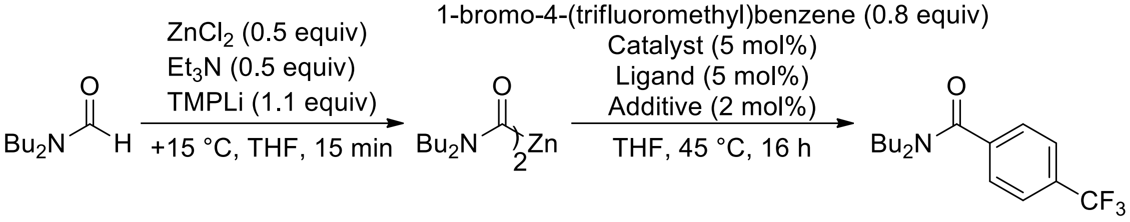

Entry	Precatalyst	Ligand	Additive	Conversion of ArBr [%]	GC yield [%]
1	$PdCl_2$(dppf)	/	CuCN·2LiCl	>99	88 (86)
2*	$PdCl_2$(dppf)	/	CuCN·2LiCl	>99	29
3	$PdCl_2$(dppf)	/	/	15	14
4	/	dppf	/	<5	0
5	/	dppf	CuCN·2LiCl	<5	0
6	/	/	CuCN·2LiCl	<5	0
7	$Pd(dba)_2$	dppf	CuCN·2LiCl	ND	82
8	$Pd(dba)_2$	dppf	/	ND	55
9	$Pd(OAc)_2$	dppf	CuCN·2LiCl	ND	78
10	$Pd(OAc)_2$	dppf	/	ND	54
11	$PdCl_2(PPh_3)$	/	/	ND	15
12	$PdCl_2(PPh_3)$	/	CuCN·2LiCl	ND	6
13	Pd1	/	/	ND	6
14	Pd1	/	CuCN·2LiCl	ND	0
15	XantPhos$PdCl_2$	/	/	>99	84
16	XantPhos$PdCl_2$	/	CuCN·2LiCl	20	17
17	$Pd(OAc)_2$	SPhos	/	<5	0
18	$Pd(OAc)_2$	SPhos	CuCN·2LiCl	<5	0
19	Pd2	/	/	>99	78
20	Pd2	/	CuCN·2LiCl	<5	0
21	$Pd(dba)_2$	dppbe	/	<5	1
22	$Pd(dba)_2$	dppbe	CuCN·2LiCl	<5	9

*Metalation performed with $TMP_2Zn·2MgCl_2·2LiCl$ ($MgCl_2$ has negative impact on cross coupling). Tetradecane was used as internal standard
Pd1: Bis(di-tert-butyl(4-dimethylaminophenyl)phoshine)dichloropalladium(II)
Pd2: [1,2-Bis(diphenylphosphino)ethane]dichloropalladium(II)
dppf: 1,1′-Ferrocenediyl-bis(diphenylphosphine)
dppbe: 1,2-Bis(diphenylphosphino)benzene

Typical Procedures

Typical Procedure 1A (TP1A): Cu-catalyzed allylation/propargylation of carbamoylzinc reagents **6a-6j** with allylic or propargylic bromides (Method A)

A heat and vacuum dried flask flushed with argon was placed into a water bath at 15 °C. Formamide (1.00 mmol, 1.0 equiv), $ZnCl_2$ solution 1 M in THF (500 µL, 0.50 mmol, 0.5 equiv), Et_3N (51 mg, 0.50 mmol, 0.5 equiv) and THF (2 mL) were added. A freshly prepared TMPLi as ca. 0.5 M solution in THF (1.1 mmol, 1.1 equiv) was dropwise added over 1-2 min. The reaction mixture was stirred for additional 15 min. A solution of dicarbamoylzinc reagent $(R^1R^2NCO)_2Zn$ was obtained. The allylic or propargylic bromide (1.1 mmol, 1.1 equiv) was added neat at 22 °C quickly followed by CuCN·2LiCl 1 M solution in THF (100 µL, 0.01 mmol, 10 mol%). The reaction mixture was stirred for 15 min and then quenched with saturated $NH_4Cl_{(aq)}$ (2 mL). The product was extracted with ethyl acetate or dichloromethane (3 x 100 mL) and the organic layer was dried with $MgSO_4$. After removal of the solvent *in vacuo*, flash column chromatography purification afforded analytically pure products of type **8**.

Typical Procedure 1B (TP1B): Cu-catalyzed allylation of carbamoylzinc reagents **6k-6o** with allylic or propargylic bromides (Method B)

A heat and vacuum dried flask flushed with argon was placed into a water bath at 22 °C. Neat formamide (1.00 mmol, 1.0 equiv) was placed in the flask (oily substrates were transferred as a THF solution and THF was removed under high vacuum) and solution of TMP_2Zn (0.55 mmol, 0.55 equiv) in THF was dropwise added. After stirring for the indicated time (2-24 h) a solution of dicarbamoylzinc reagent $(R^1R^2NCO)_2Zn$ was obtained. After dilution with THF (10 mL) allylic bromide (1.1 mmol, 1.1 equiv) was added neat at 22 °C quickly followed by CuCN·2LiCl 1 M solution in THF (100 µL, 0.01 mmol, 10 mol%). The reaction mixture was stirred for the indicated time (15 min to 12 h) and then quenched with saturated $NH_4Cl_{(aq)}$ (2 mL). The product was extracted with ethylacetate or DCM (3 x 100 mL) and the organic layer was dried with $MgSO_4$. After removal of the solvent *in vacuo*, flash column chromatography purification afforded analytically pure products of type **8**.

NOTE: Examples **8l**, **8m**, **8n** and **8p** were made using $TMP_2Zn·2MgCl_2·2LiCl$ and example **8o** was prepared using $TMP_2Zn·2LiCl$ (without $MgCl_2$).

Typical Procedure 2 (TP2): Benzylation of carbamoylzinc reagents **6a**, **6d** and **6g** with benzylic bromides

A heat and vacuum dried flask flushed with argon was placed into a water bath at 15 °C. Formamide (1.00 mmol, 1.0 equiv), $ZnCl_2$ solution 1 M in THF (500 µL, 0.50 mmol, 0.5 equiv), Et_3N (51 mg, 0.50 mmol, 0.5 equiv) and THF (2 mL) were added. Freshly prepared TMPLi as ca. 0.5 M solution in THF (1.1 mmol, 1.1 equiv) was dropwise added over 1-2 min. The reaction mixture was stirred for additional 15 min. A solution of dicarbamoylzinc reagent $(R^1R^2NCO)_2Zn$ was obtained. A solution of $MgCl_2·LiCl$ ca. 0.5 M in THF (2.0 mL) was dropwise added at 22 °C. After 5 min of stirring CuCN·2LiCl 1 M solution in THF (100 µL, 0.01 mmol, 10 mol%) at room temperature. The benzylic bromide was added to the reaction mixture at room temperature neat if it is a liquid or as a 1 M solution in THF if it is a solid. The reaction mixture was stirred for 24 h and then quenched with saturated $NH_4Cl_{(aq)}$ (2 mL). The product was extracted with ethyl acetate or DCM (3 x 100 mL) and organic layer was dried with $MgSO_4$. After removal of the solvent *in vacuo*, flash column chromatography purification afforded analytically pure products of type **9**.

Typical Procedure 3 (TP3): Reaction of carbamoylzinc reagents **6a** and **6f** with aldehydes

A heat and vacuum dried flask flushed with argon was placed into water bath at 15 °C. Formamide (1.00 mmol, 1.0 equiv), $ZnCl_2$ solution 1 M in THF (500 µL, 0.50 mmol, 0.5 equiv), Et_3N (51 mg, 0.50 mmol, 0.5 equiv) and THF (2.0 mL) were added. Freshly prepared TMPLi as ca. 0.5 M solution in THF (1.1 mmol, 1.1 equiv) was dropwise added over 1-2 min. The reaction mixture was stirred for additional 15 min. A solution of dicarbamoylzinc reagent $(R^1R^2NCO)_2Zn$ was obtained. A solution of $MgCl_2$·LiCl ca. 0.5 M in THF (2.0 mL, 1.0 mmol, 1.0 equiv) was dropwise added at 22 °C. After 5 min of stirring, neat aldehyde (1.2 mmol, 1.2 equiv) was added at room temperature. The reaction mixture was stirred for 24 h and then quenched with saturated $NH_4Cl_{(aq)}$ (2 mL). The product was extracted with ethyl acetate or DCM (3 x 100 mL) and the organic layer was dried with $MgSO_4$. After removal of the solvent *in vacuo*, flash column chromatography purification afforded analytically pure products of type **10**.

Typical Procedure 4 (TP4): Acylation of carbamoylzinc reagents **6a** and **6f** with acyl chlorides

A heat and vacuum dried flask flushed with argon was placed into water bath at 15 °C. Formamide (1.00 mmol, 1.0 equiv), $ZnCl_2$ solution 1 M in THF (500 µL, 0.50 mmol, 0.5 equiv), Et_3N (51 mg, 0.50 mmol, 0.5 equiv) and THF (2.0 mL) were added. Freshly prepared TMPLi as ca. 0.5 M solution in THF (1.1 mmol, 1.1 equiv) was dropwise added over 1-2 min. The reaction mixture was stirred for additional 15 min. A solution of dicarbamoylzinc reagent $(R^1R^2NCO)_2Zn$ was obtained. Freshly distilled acyl chloride was added at 22 °C. The reaction mixture was stirred for 16 h and then quenched with saturated $NH_4Cl_{(aq)}$ (2 mL). The product was extracted with ethyl acetate or DCM (3 x 100 mL) and organic layer was dried with $MgSO_4$. After removal of the solvent *in vacuo*, flash column chromatography purification afforded analytically pure products of type **11**.

Typical Procedure 5 (TP5): 1,4-Addition of carbamoylzinc reagents **6a**

A heat and vacuum dried flask flushed with argon was placed into water bath at 15 °C. Formamide (2.00 mmol, 2.0 equiv), $ZnCl_2$ solution 1 M in THF (1.00 mL, 1.00 mmol, 1.0 equiv), Et_3N (101 mg, 1.00 mmol, 1.0 equiv) and THF (4.0 mL) were added. Freshly prepared TMPLi as ca. 0.5 M solution in THF (2.2 mmol, 2.2 equiv) was dropwise added over 1-2 min. Reaction mixture was stirred for additional 15 min and then was cooled to –78 °C. A solution of dicarbamoylzinc reagent $(R^1R^2NCO)_2Zn$ was obtained. CuCN·2LiCl 1 M solution in THF (1.00 mL, 1.00 mmol, 1.0 equiv) was dropwise added. Stirring was continued for 30 min after which a BF_3·Et_2O (123 µL, 1.0 mmol, 1.0 equiv) was added. It was immediately followed by a neat cyclohex-2-en-1-one (96.0 mg, 1.00 mmol, 1.0 equiv). The reaction temperature was maintained at -78 °C using a cryostat for 24 h. It was then quenched with 10 mL of saturated $NH_4Cl_{(aq)}$. The product was extracted with ethyl acetate (3 x 100 mL) and organic layer was dried with $MgSO_4$. After removal of the solvent *in vacuo*, flash column chromatography purification afforded analytically pure product **12a**.

Typical Procedure 6 (TP6): Cross-coupling of carbamoyl zinc reagents **6a**, **6e**, **6f**, **6m**, **6p** and **6q** with aryl or alkenyl bromides

To a flame dried 20 mL pressure tube formamide (1.00 mmol, 1.0 equiv) in THF, $ZnCl_2$ as 1 M solution in THF (500 µL, 0.50 mmol, 0.5 equiv) and Et_3N (51.0 mg, 0.50 mmol, 0.5 equiv) and THF (2 mL) were added. At 15 °C, a 0.5 M solution of TMPLi (1.1 equiv) in THF was dropwise added over 2 min. The mixture was left to stir for 15 min to obtain clear solution of dicarbamoylzinc $(R^1R^2NCO)_2Zn$. In a separate flask Pd(dppf)Cl_2 (36.6 mg, 0.05 mmol, 5 mol%), aryl bromide (0.80 mmol, 0.8 equiv) and THF (2 mL) were added to form a fine suspension. The suspension was quantitatively transfered into a solution of the biscarbamoylzinc reagent followed by a 1 M solution of CuCN·2LiCl (20 µL, 20.0 µmol, 2

mol%). The pressure tube sealed with a single-use Teflon stopper and the reaction mixture was stirred at 45 °C for 16 h. After cooling the reaction mixture to 22 °C, the sealed tube was carefully opened and saturated $NH_4Cl_{(aq)}$ (2 mL) was added. The product was extracted with ethyl acetate or DCM (3 x 100 mL) and organic layer was dried with $MgSO_4$. After removal of the solvent *in vacuo*, flash column chromatography purification afforded analytically pure products of type **13**.

Preparation of starting materials

N-(2-((tert-Butyldimethylsilyl)oxy)ethyl)-N-methylformamide (4b)

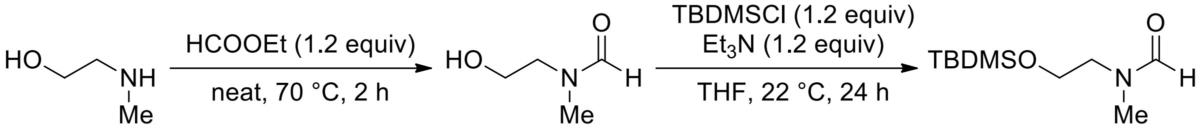

INT-4b1

N-Methylaminoethanol (51.08 g, 0.68 mol, 1.0 equiv) was cooled to 0 °C (ice/water bath) and HCOOEt (74.08, 1.00 mol, 1.47 equiv) was added portionwise. The reaction mixture was heated to reflux for 1 h, concentrated *in vacuo* and then distilled by slow fractional distillation (6 mbar, 160-165 °C) to give N-(2-hydroxyethyl)-N-methylformamide (64.4 g, 0.624 mol, 92% yield) as clear, colorless liquid. Small amound of bisformylation impurities can be present.

^{1}H-NMR (400 MHz, $CDCl_3$): δ / ppm = 7.92/7.88 (2 x s, 1H), 4.40/3.96 (t, *J* = 5.7 Hz, 1H), 3.68 – 3.54 (m, 2H), 3.39 – 3.33/3.29 – 3.21 (2 x m, 2H), 2.93/2.81 – 2.73 (s + m, 3H) (mixture of rotamers).
^{13}C-NMR (101 MHz, $CDCl_3$): δ / ppm = 163.6, 163.6, 59.5, 58.4, 52.1, 47.1, 35.8, 29.7.
IR (Diamond-ATR, neat): $\tilde{\nu}$ / cm^{-1} = 3368, 2961, 2923, 1646, 1441, 1394, 1258, 1204, 1081, 1049, 866, 795.
MS (EI, 70 eV): *m/z* (%) = 72 (14), 45 (14), 44 (51), 43 (100), 42 (15).
HRMS (EI): *m/z* calc. for [$C_4H_9NO_2$]: 103.0633; found 103.0636.

N-(2-Hydroxyethyl)-*N*-methylformamide (2.50 g, 24.2 mmol, 1.0 equiv) was dissolved in THF (30 mL) and TBDMSCl (4.39 g, 29.1 mmol, 1.2 equiv) and Et_3N (2.94 g, 29.0 mmol, 1.2 equiv) were added. After 24 h, the white suspension was quenched with 50 mL distilled H_2O and transferred into separatory funnel. The mixture was extracted with DCM (3 x 200 mL), combined organic layers dried with $MgSO_4$, filtered and solvent removed *in vacuo*. Purification by flash column chromatography with pentane:ethyl acetate = 1:1 afforded *N*-(2-((tert-butyldimethylsilyl)oxy)ethyl)-*N*-methylformamide (**4b)** (4.78 g, 22 mmol, 91% yield) as colorless liquid.

^{1}H-NMR (400 MHz, $CDCl_3$): δ / ppm = 8.00 (s, 1H), 3.66 (t, *J* = 5.1 Hz, 2H), 3.30 (t, *J* = 5.1 Hz, 2H), 2.86 (s, 3H), 0.85 – 0.82 (m, 9H), 0.02 – 0.00 (m, 6H) (major rotamer).
8.02 (s, 1H), 3.74 (t, *J* = 5.4 Hz, 2H), 3.41 (t, *J* = 5.4 Hz, 2H), 3.02 (s, 3H), 0.88 – 0.85 (m, 9H), 0.04 – 0.02 (m, 6H) (minor rotamer).
^{13}C-NMR (101 MHz, $CDCl_3$): δ / ppm = 163.42, 162.7, 61.4, 60.2, 52.1, 47.2, 36.6, 30.2, 25.9, 25.9, 18.3, 18.2, -5.4, -5.4, -5.4.
IR (Diamond-ATR, neat): $\tilde{\nu}$ / cm^{-1} = 2928, 2852, 1649, 1462, 1443, 1414, 1366, 1352, 1318, 1302, 1269, 1253, 1245, 1207, 1186, 1154, 1136, 1098, 1060, 992, 958, 904, 891, 840, 821, 774, 717.
MS (EI, 70 eV): *m/z* (%) = 160 (100), 116 (33), 86 (21), 75 (45).
HRMS (EI): *m/z* calc. for [$C_{12}H_{23}NO$]: 217.1498; found: 202.1256 (M-CH_3).

***trans*-Octahydroquinoline-1(2H)-carbaldehyde (4e)**

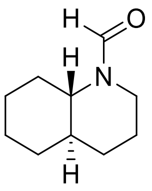

The flask was heat dried under vacuum and filled with Ar. Acetic acid ahnhydride (7.37 mL, 78.0 mmol, 2.6 eq.) and formic acid (3.62 mL, 96.0 mmol, 3.2 equiv) were mixed in THF (100 mL) and heated at 40 °C for 1 h. The reaction mixture was cooled to −10 °C with ice/$NaCl_{(aq)}$ bath. (*trans*)-Decahydroisoquinoline (4.1 g, 30.0 mmol, 1.0 equiv) was dissolved in THF (50 mL), dropwise added to the reaction mixture and stirred for 2 h in the ice/$NaCl_{(aq)}$ bath. The solution was neutralized with saturated $Na_2CO_{3(aq)}$ solution. Complete consumption of starting material and product formation was verified by GC/MS analysis. The product was extracted with DCM (4 x 300 mL), combined organic layers dried over $MgSO_4$, filtered and the solvent removed *in vacuo*. After a flash column chromatography with ethyl acetate:pentane = 4:6, *trans*-octahydroisoquinoline-2(1H)-carbaldehyde (4.82 g, 28.8 mmol, 96% yield) was obtained as a yellowish solid.

^{1}H-NMR (400 MHz, $CDCl_3$): δ / ppm = 8.15 (s, 1H), 4.63 – 4.55 (m, 1H), 2.83 – 2.72 (m, 1H), 2.39 (td, *J* = 12.9, 3.0 Hz, 1H), 2.11 – 2.02 (m, 1H), 1.94 – 1.87 (m, 1H), 1.78 – 1.71 (m, 1H), 1.70 – 1.61 (m, 3H), 1.61 – 1.52 (m, 1H), 1.51 – 1.36 (m, 1H), 1.35 – 1.10 (m, 4H), 1.09 – 0.96 (m, 1H).
^{13}C-NMR (101 MHz, $CDCl_3$): δ / ppm = 158.7, 62.7, 43.4, 42.0, 32.8, 32.7, 29.2, 25.8, 25.5, 25.4.
IR (Diamond-ATR, neat): $\tilde{\nu}$ / cm^{-1} = 2928, 2852, 1649, 1462, 1443, 1414, 1366, 1352, 1318, 1302, 1269, 1253, 1245, 1207, 1186, 1154, 1136, 1098, 1060, 992, 958, 904, 891, 840, 821, 774, 717.
MS (EI, 70 eV): *m/z* (%) = 167 (29), 124 (100), 96 (61), 79 (12).
HRMS (EI): *m/z* calc. for [$C_{12}H_{23}NO$]: 167.1310; found: 167.1303 (M+H).
M.p. (°C): 41.5-43.6.

4-(3-(Trifluoromethyl)pyridin-2-yl)piperazine-1-carbaldehyde (4f)

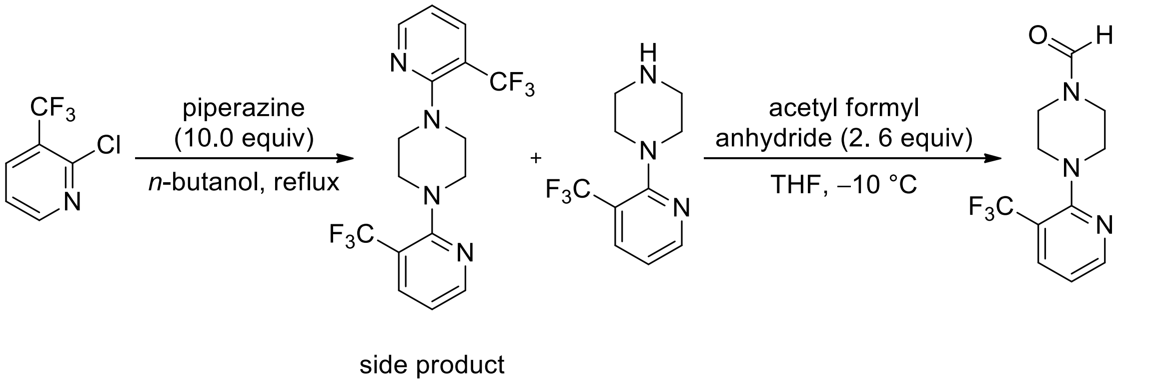

To a solution of 2-chloro-3-(trifluoromethyl)pyridine (4.5 g, 25 mmol, 1.0 equiv) in *n*-butanol was added piperazine (21.5 g, 250 mmol, 10.0 equiv) in one portion and resulting suspension was heated under reflux (oil bath temperature 130 °C) for 17 h. Heating was stopped, warm reaction mixture was transferred into aqueous 2M $NaOH_{(aq)}$ solution (400 mL) and product extracted with ethyl acetate (4x700 mL). Organic layers were combined, dried over $MgSO_4$, filtered and the solvent removed *in vacuo* to obtain crude 1-(3-(trifluoromethyl)pyridin-2-yl)piperazine. The product was purified *via* column chromatography using first pentane:ethyl acetate:Et_3N = 6:3:1 to elute bisarylation side product and then increasing polarity to ethyl acetate:Et_3N=9:1. The fractions containing the product were combined and concentrated *in vacuo* to yield 1-(3-(trifluoromethyl)pyridin-2-yl)piperazine (5.3 g, 22.9 mmol, 92% yield) as redish oil.

The flask was heat dried under vacuum and filled with Ar. Acetic acid ahnhydride (5.4 mL, 57.2 mmol, 2.6 eq.) and formic acid (2.66 mL, 70.2 mmol, 3.2 equiv) were mixed in THF (100 mL) and heated at 40 °C for 1 h. The reaction mixture was cooled to −10°C with ice/$NaCl_{(aq)}$

bath. 1-(3-(Trifluoromethyl)pyridin-2-yl)piperazine (5.09 g, 22.0 mmol, 1.0 equiv) was dissolved in THF (50 mL), dropwise added to the reaction mixture and stirred for 2 h in the ice/$NaCl_{(aq)}$ bath. The solution was neutralized with sat. Na_2CO_3. Complete consumption of starting material and product formation was verified by GC/MS analysis. The product was extracted with DCM (4 x 300 mL), combined organic layers dried over $MgSO_4$, filtered and the solvent removed *in vacuo*. After a flash column chromatography with ethyl acetate:pentane 1:1, 4-(3-(Trifluoromethyl)pyridin-2-yl)piperazine-1-carbaldehyde (5.08 g, 19.6 mmol, 89% yield) was obtained as a redish solid.

^{1}H-NMR (400 MHz, $CDCl_3$): δ / ppm = 8.47 – 8.41 (m, 1H), 8.08 (s, 1H), 7.89 (dd, *J* = 7.8, 1.9 Hz, 1H), 7.11 – 7.02 (m, 1H), 3.71 – 3.65 (m, 2H), 3.54 – 3.46 (m, 2H), 3.28 – 3.23 (m, 2H), 3.22 – 3.17 (m, 2H).
^{13}C-NMR (101 MHz, $CDCl_3$): δ / ppm = 161.0, 151.4 (q, *J* = 1.2 Hz), 137.3 (q, *J* = 5.0 Hz), 123.9 (q, *J* = 272.6 Hz), 118.3, 118.1 (q, *J* = 30.9 Hz), 51.5 (q, *J* = 1.2 Hz), 50.7 (q, *J* = 1.3 Hz), 45.8, 40.2.
^{19}F-NMR (377 MHz, $CDCl_3$): –60.4.
IR (Diamond-ATR, neat): $\tilde{\nu}$ / cm^{-1} = 1664, 1589, 1571, 1438, 1401, 1368, 1307, 1280, 1251, 1225, 1192, 1137, 1116, 1079, 1062, 1024, 1010, 932, 812, 796, 782, 671.
MS (EI, 70 eV): *m/z* (%) = 201 (20), 189 (21), 188 (41), 187 (100), 175 (70), 173 (14), 167 (18), 155 (34), 147 (34), 146 (22), 128 (54), 127 (12).
HRMS (EI): *m/z* calc. for [$C_{12}H_{23}NO$]: 259.0932; found: 260.1005 (M+H).
M.p. (°C): 76.6-77.8.

7,7,9,9-Tetramethyl-1,4-dioxa-8-azaspiro[4.5]decane-8-carbaldehyde (4h)

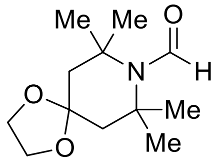

The compound was synthesized according to the modified literature procedure. [7]
4,4-Ethylenedioxy-2,2,6,6-tetramethylpiperidine (2.12 g, 10.6 mmol, 1.0 equiv), benzyltriethylammonium chloride (1.55 g, 6.80 mmol, 0.6 eq.) as phase-transfer-catalyst, 50 wt.-% aqueous NaOH (37 mL), $CHCl_3$ (11.1 mL, 136 mmol, 8.5 equiv) and CH_2Cl_2 (49 mL) were mixed and refluxed for 24 h at 50 °C. On cooling, the mixture was diluted with water and extracted with CH_2Cl_2. The organic layers were dried, filtered and evaporated for the purification of the crude product by column chromatography (SiO_2, 1 L isohexane:ethyl acetate = 4:1, then isohexane:ethyl acetate = 7:3). The fractions containing the product were combined and concentrated *in vacuo* to yield *N*-formyl-4,4-ethylenedioxy-2,2,6,6-tetramethylpiperidine (2.14 g, 9.44 mmol, 89% yield) as light brown oil which solidified over time.

^{1}H-NMR (400 MHz, $CDCl_3$): δ / ppm = 8.47 (s, 1H), 3.88 (s, 4H), 1.96 (s, 2H), 1.93 (s, 2H), 1.52 (s, 6H), 1.43 (s, 6H).
^{13}C-NMR (101 MHz, $CDCl_3$): δ / ppm = 162.7, 105.7, 63.9, 56.1, 55.5, 48.0, 47.9, 32.4, 28.2.
IR (Diamond-ATR, neat): $\tilde{\nu}$ / cm^{-1} = 2974, 2928, 2882, 1646, 1453, 1434, 1422, 1398, 1376, 1363, 1332, 1319, 1282, 1242, 1220, 1188, 1178, 1135, 1100, 1068, 1021, 994, 978, 952, 934, 910, 861, 846, 801, 710.
MS (EI, 70 eV): *m/z* (%) = 212 (16), 127 (100), 98 (24), 83 (15).
HRMS (EI): *m/z* calc. for [$C_{12}H_{23}NO$]: 227.1521; found 227. 1513.
M.p. (°C): 50.6-51.9.

***N*,*N*-Dibutyl[^{13}C]formamide (4j)**

[7] Z. Blum, K. Nyberg, *Acta Chem. Scand. Ser. B* **1981**, *35*, 743.

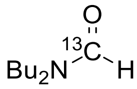

To DCC (5.11 g, 24.8 mmol, 1.2 equiv) dissolved in dry DCM (20 mL), was added HOBt (3.35 g, 24.8 mmol, 1.2 equiv) in one portion at 25 °C. After 20-30 min, a clear solution was obtained and cooled to –25 °C. In a separate flask, $H^{13}COOH$ (95 wt% in H_2O, 99% ^{13}C atom) was dissolved in dissolved DCM (20 mL) and cooled to –25 °C (suspension formed). The solution of DCC/HOBt was dropwise added over 15 min into the solution of $H^{13}COOH$. After stirring for 30 min at –25 °C, neat Bu_2NH (3.33 g, 25.8 mmol, 1.25 equiv) was dropwie added. The suspension was allowed to slowly warm to 22 °C and stirred for 24 h. The reaction mixture was filtered over sinter to remove *N,N'*-dicyclohexylurea. The DCM layer was washed with 10% Na_2CO_3(aq) (3 x 30 mL) and then with 1 M HCl (3 x 10 mL). After drying over $MgSO_4$, filtrating and concentrating the organic layers distillation under reduced pressure (69 °C, 0.15 mbar) gave pure $Bu_2N^{13}CHO$ (2.06 g, 13.1 mmol, 64% yield) as colorless liquid.

^{1}H-NMR (400 MHz, $CDCl_3$): δ / ppm = 8.01 (d, *J* = 190.5 Hz, 1H), 3.30 – 3.22 (m, 2H), 3.21 – 3.12 (m, 2H), 1.54 – 1.43 (m, 4H), 1.35 – 1.21 (m, 4H), 0.91 (td, *J* = 7.3, 2.8 Hz, 6H).
^{13}C-NMR (101 MHz, $CDCl_3$): δ / ppm = 162.8, 47.2 (d, *J* = 4.6 Hz), 41.9, 30.8, 29.5, 20.3, 19.7, 13.9, 13.7.
IR (Diamond-ATR, neat): $\tilde{\nu}$ / cm^{-1} = 2958, 2932, 2873, 1626, 1460, 1422, 1398, 1378, 1212, 1198, 1113, 950, 733.
MS (EI, 70 eV): *m/z* (%) = 158 (12), 114 (100), 72 (50).
HRMS (EI): *m/z* calc. for [^{13}C-$C_9H_{19}NO$]: 158.1501; found 159.1572 (M+H).

Ethyl 4-(2-(*N*-methylformamido)ethoxy)benzoate (4k)

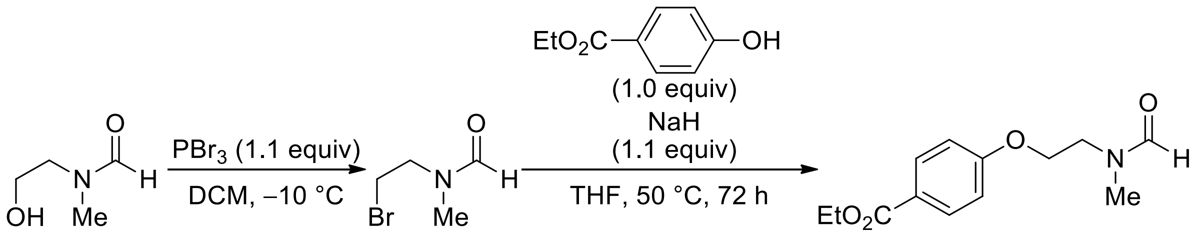

INT-4k1

N-(2-Hydroxyethyl)-*N*-methylformamide (10.31 g, 100 mmol, 1.0 equiv) in DCM (100mL) was cooled to –10 °C and PBr_3 (28.4 g, 105 mmol, 1.05 equiv) was added via dropping funnel over 1 h. The solution was stirred for 12 h and was then neutralized with distilled water (200 mL). After stirring for ca. 45 min the reaction mixture was extracted with DCM (3 x 200 mL). Combined organic layers were dried over $MgSO_4$, filtered and the solvent removed *in vacuo*. After a fractional distillation (63 °C, 0.12 mbar), *N*-(2-bromoethyl)-*N*-methylformamide (6.43 g, 39.0 mmol, 39% yield) was obtained as a colorless liquid.

^{1}H-NMR (400 MHz, $CDCl_3$): δ / ppm = 8.02 (s, 1H), 3.65/3.58 (t, J = 6.6 Hz/6.1 Hz, 2H), 3.46 – 3.40 (m, 2H), 2.99/2.82 (2 x s, 3H) (mixture of rotamers).
^{13}C-NMR (101 MHz, $CDCl_3$): δ / ppm = 162.9, 162.9, 50.9, 46.3, 35.5, 29.4, 29.3, 28.1 (mixture of rotamers).
IR (Diamond-ATR, neat): $\tilde{\nu}$ / cm^{-1} = 3356, 2957, 2775, 1717, 1584, 1461, 1422, 1390, 1353, 1153, 1074, 1026, 994, 900, 847, 794, 661.
MS (EI, 70 eV): *m/z* (%) = 82 (14), 80 (14), 72 (20), 70 (11), 61 (16), 45 (15), 44 (99), 43 (100), 41 (11).
HRMS (EI): *m/z* calc. for [C_4H_8BrNO]: 164.9789; found 164.9789.

Ethyl 4-hydroxybenzoate (4.15 g, 25 mmol, 1.0 equiv) was dissolved in THF (50 mL) and 60% NaH (1.20 g, 30 mmol, 1.2 equiv) was added portionwise over 30 min at 22 °C. Thereafter *N*-(2-bromoethyl)-*N*-methylformamide was dropwise added and solution heated to 50 °C (oil bath temp.) over 72 h. Extracted with DCM (3 x 200 mL) and combined organic layers were dried over $MgSO_4$, filtered and the solvent removed *in vacuo*. Column chromatography on SiO_2 with ethyl acetate afforded ethyl 4-(2-(N-methylformamido)ethoxy)benzoate (3.68 g, 14.6 mmol, 59% yield) as white solid.

^{1}H-NMR (400 MHz, $CDCl_3$): δ / ppm = 8.11/8.05 (2 x s, 1H), 7.99 – 7.92 (m, 2H), 6.91 – 6.80 (m, 2H), 4.36 – 4.25 (m, 2H), 4.15 (t, *J* = 5.2 Hz, 1H), 4.08 (t, *J* = 5.1 Hz, 1H), 3.70 (t, *J* = 5.2 Hz, 1H), 3.62 (t, *J* = 5.1 Hz, 1H), 3.08/2.92 (2 x s, 2H), 1.37 – 1.31 (m, 3H).
^{13}C-NMR (101 MHz, $CDCl_3$): δ / ppm = 166.2, 166.2, 163.2, 162.9, 162.0, 161.7, 131.6, 131.6, 123.7, 123.4, 114.0, 114.0, 77.2, 66.3, 65.0, 60.8, 60.7, 48.8, 44.1, 36.4, 30.2, 14.4.
IR (Diamond-ATR, neat): $\tilde{\nu}$ / cm^{-1} = 1696, 1677, 1665, 1606, 1582, 1510, 1470, 1425, 1391, 1363, 1271, 1252, 1213, 1175, 1126, 1102, 1087, 1078, 1043, 1016, 980, 904, 878, 844, 770, 694, 657.
MS (EI, 70 eV): *m/z* (%) = 86 (100), 72 (13), 58 (19), 44 (19).
HRMS (EI): *m/z* calc. for [$C_{13}H_{17}NO_4$]: 251.1158; found 250.1075 (M-H).
M.p. (°C): 94.7-97.3.

***N*-(2-(2-Benzoylphenoxy)ethyl)-*N*-methylformamide (4l)**

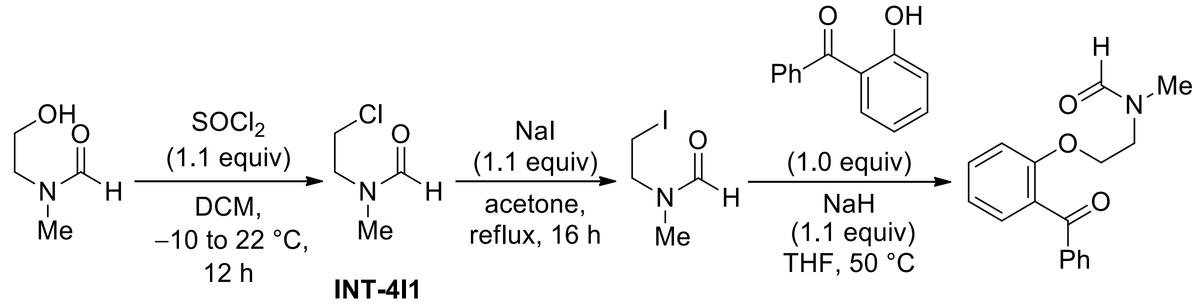

N-(2-Hydroxyethyl)-*N*-methylformamide (10.31 g, 0.10 mol, 1.0 equiv) in DCM (100 mL) was cooled to −10 °C and $SOCl_2$ (38.07 g, 0.32 mol, 1.02 equiv) was added via dropping funnel over 2 h. The solution was stirred for 12 h and was then concentrated *in vacuo*. Fractional distillation under reduced pressure with vigreaux column (0.1 mbar, 85-90 °C) afforded *N*-(2-chloroethyl)-*N*-methylformamide (36.0 g, 0.296 mol, 95% yield) as a colorless liquid.

^{1}H-NMR (400 MHz, $CDCl_3$): δ / ppm = 8.02/8.01 (2 x s, 1H), 3.64 – 3.55 (m, 3H), 3.54 – 3.49 (m, 1H), 3.00/2.83 (2 x s, 3H) (mixture of rotamers).
^{13}C-NMR (101 MHz, $CDCl_3$): δ / ppm = 163.1, 163.0, 51.0, 46.4, 41.2, 40.8, 35.8, 29.5 (mixture of rotamers).
IR (Diamond-ATR, neat): $\tilde{\nu}$ / cm^{-1} = 1659, 1485, 1439, 1396, 1362, 1298, 1261, 1235, 1163, 1072, 1035, 675.
MS (EI, 70 eV): *m/z* (%) = 121.0288 (19), 72 (83), 44 (100), 42 (38), 41 (14).
HRMS (EI): *m/z* calc. for [C_4H_8ClNO]: 121.0294; found 121.0288.

N-(2-Chloroethyl)-*N*-methylformamide (29.2 g, 0.24 mol, 1.0 equiv) in anhydrous acetone (25 mL) was dropwise added to NaI (37.5 g, 250 mmol, 1.04 equiv) in anhydrous acetone (250 mL) over 45 min at 22 °C. The solution was heated to 70 °C (oil bath temp) for 16 h. Small amount of *N*-(2-chloroethyl)-*N*-methylformamide (<5%) was left in the reaction mixture. The fractional distillation under reduced pressure (0.2 mbar, 118-122 °C) afforded *N*-(2-iodooethyl)-*N*-methylformamide (44.73 g, 210 mmol, 87% yield) as slightly yellowish oil. The iodide was stored in the freezer under argon.

(2-Hydroxyphenyl)(phenyl)methanone (5.94 g, 30 mmol, 1.0 equiv) was dissolved in THF (50 mL) and 60% NaH (1.32 g, 33 mmol, 1.1 equiv) was added portionwise over 30 min at 22 °C. Thereafter *N*-(2-iodoethyl)-*N*-methylformamide was dropwise added and solution heated to 50 °C (oil bath temp.) over 24 h. Extracted with DCM (3 x 200 mL) and combined organic layers were dried over $MgSO_4$, filtered and the solvent removed *in vacuo*. Column chromatography on SiO_2 with ethyl acetate afforded *N*-(2-(2-benzoylphenoxy)ethyl)-*N*-methylformamide (3.41 g, 12.0 mmol, 40% yield) as yellow oil.

^{1}H-NMR (400 MHz, $CDCl_3$): δ / ppm = 7.80 – 7.71 (m, 2H), 7.57 – 7.49 (m, 2H), 7.46 – 7.31 (m, 4H), 7.09 – 6.99 (m, 1H), 6.93 – 6.87 (m, 1H), 4.04 (t, *J* = 5.0 Hz, 1H), 3.95 (t, *J* = 5.0 Hz, 1H), 3.36 (t, *J* = 5.0 Hz, 1H), 3.22 (t, *J* = 5.0 Hz, 1H), 2.60/2.52 (2 x s, 3H) (mixture of rotamers).
^{13}C-NMR (101 MHz, $CDCl_3$): δ / ppm = 196.4, 196.1, 162.8, 162.6, 156.0, 155.9, 138.0, 137.8, 133.1, 133.1, 132.1, 132.1, 130.0, 129.7, 129.6, 129.5, 129.1, 128.8, 128.3, 121.5, 121.1, 112.4, 112.0, 66.8, 66.2, 48.7, 44.2, 36.1, 30.0 (mixture of rotamers).
IR (Diamond-ATR, neat): $\tilde{\nu}$ / cm^{-1} = 1658, 1597, 1580, 1486, 1449, 1388, 1316, 1295, 1238, 1152, 1110, 1090, 1074, 927, 905, 750, 701.
MS (EI, 70 eV): *m/z* (%) = 197 (11), 152 (10), 91 (27), 86 (100), 77 (13), 58 (17).
HRMS (EI): *m/z* calc. for [$C_{17}H_{17}NO_3$]: 283.1208; found 282.1208.

N-(3-(1,3-Dioxoisoindolin-2-yl)propyl)-*N*-isopropylformamide (4m)

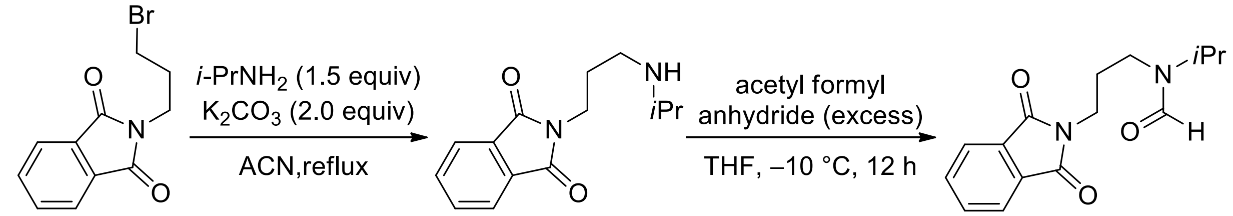

2-(3-Bromopropyl)isoindoline-1,3-dione (8.04 g, 30 mmol, 1.0 equiv), isopropylamine (2.66 g, 45.0 mmol, 1.5 equiv) and K_2CO_3 (10.37 g, 75.0 mmol, 2.5 equiv) were mixed in acetonitrile (100 mL) and refluxed for 16 h. Complete consumption of starting material and formation of 2-(3-(isopropylamino)propyl)isoindoline-1,3-dione was verified by GC/MS analysis. In a separate flask acetyl formyl anhydride (AFA) in dry THF was generated by heating a mixture of formic acid (12.1 mL, 320 mmol) and acetanhydride (24.6 mL, 260 mmol) in THF (150 mL) over 2 h at 40 °C. The reaction mixture was cooled to −10 °C with ice/$NaCl_{(aq)}$ bath. Crude reaction mixture containing 2-(3-(isopropylamino)propyl)isoindoline-1,3-dione in acetonitrile was added in small portions over 20 min to the cooled solution of acetyl formyl anhydride (AFA) in dry THF. After 12 h the reaction mixture was concentrated *in vacuo* (no aqueus work up). After a flash column chromatography on SiO_2 with pentane:ethyl acetate =1:1, *N*-(3-(1,3-dioxoisoindolin-2-yl)propyl)-*N*-isopropylformamide (7.01 g, 25.6 mmol, 85% yield) was obtained as a colorless oil which solidified over time.

^{1}H-NMR (400 MHz, $CDCl_3$): δ / ppm = 8.08/7.98 (2 x s, 1H), 7.78 – 7.72 (m, 2H), 7.68 – 7.61 (m, 2H), 3.70 – 3.59 (m, 3H), 3.26 – 3.15 (m, 2H), 1.92 – 1.82 (m, 2H), 1.24 – 1.10 (m, 6H) (mixture of rotamers).
^{13}C-NMR (101 MHz, $CDCl_3$): δ / ppm = 168.1, 168.1, 162.8, 162.2, 134.1, 133.9, 131.9, 131.7, 123.2, 123.1, 49.9, 44.6, 42.4, 38.2, 35.8, 35.3, 30.3, 28.2, 22.1, 20.2.
IR (Diamond-ATR, neat): $\tilde{\nu}$ / cm^{-1} = 1767, 1711, 1641, 1433, 1398, 1364, 1311, 1195, 1128, 1084, 1036, 1028, 1019, 890, 778, 713.
MS (EI, 70 eV): *m/z* (%) = 245 (27), 231 (20), 188 (41), 160 (100), 133 (14), 130 (25), 114 (24), 98 (12), 77 (12), 72 (18).
HRMS (EI): *m/z* calc. for [$C_{15}H_{18}N_2O_3$]: 274.1312; found 275.1383 (M+H).
M.p. (°C): 102.2 (ethyl acetate).
X-Ray: Crystals suitable for X-Ray diffraction were obtained from ethyl acetate at −18 °C. See pages 133-136.

_N_-Methyl-_N_-(2-(4-nitrophenoxy)ethyl)formamide (4n)

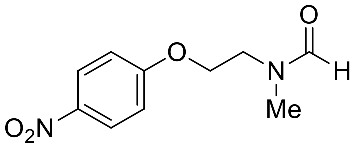

To a 2-(methylamino)ethanol (3.76 g, 50 mmol, 1.0 equiv) in THF (100 mL) at 0 °C was added 60% NaH (2.20 g, 55 mmol, 1.1 equiv) portionwise over 30 min. After stirring the obtained suspension for an additional 30 min at 22 °C, 1-fluoro-4-nitrobenzene (7.06 g, 50.0 mmol, 1.0 equiv) was added in one portion to the reaction mixture. In 1 h the reaction mixture was quenched with 2 M $NaOH_{(aq)}$ (200 mL). Deep red water layer was immediately extracted with DCM (3 x 200 mL), combined organic layers dried over $MgSO_4$, filtered and the solvent removed *in vacuo*. After a flash column chromatography on SiO_2 with ethyl acetate followed by ethyl acetate:triethylamine:methanol = 9:0.5:0.5, *N*-methyl-2-(4-nitrophenoxy)ethanamine (6.01 g, 31.1 mmol, 62% yield) was obtained as a yellow/orange liquid.

The flask was heat dried under vacuum and filled with Ar. Acetic acid ahnhydride (7.37 mL, 78.0 mmol, 2.6 eq.) and formic acid (3.62 mL, 96.0 mmol, 3.2 equiv) were mixed in THF (100 mL) and heated at 40 °C for 1 h. The reaction mixture was cooled to –10 °C with ice/$NaCl_{(aq)}$ bath. *N*-methyl-2-(4-nitrophenoxy)ethanamine (5.89 g, 30.0 mmol, 1.0 equiv) was dissolved in THF (50 mL), dropwise added to the reaction mixture and stirred for 2 h in the ice/$NaCl_{(aq)}$ bath. The solution was neutralized with sat. Na_2CO_3. Complete consumption of starting material and the product formation was verified by GC/MS analysis. The product was extracted with DCM (4 x 300 mL), combined organic layers dried over $MgSO_4$, filtered and the solvent removed *in vacuo*. After a flash column chromatography with ethyl acetate, *N*-methyl-*N*-(2-(4-nitrophenoxy)ethyl)formamide (5.90 g, 26.3 mmol, 88% yield) was obtained as an orange liquid.

^{1}H-NMR (400 MHz, $CDCl_3$): δ / ppm = 8.08 – 8.03/7.99 (m + s, 3H), 6.94 – 6.81 (m, 2H), 4.14 (t, *J* = 5.4 Hz, 1H), 4.10 (t, *J* = 5.1 Hz, 1H), 3.66 (t, *J* = 5.4 Hz, 1H), 3.62 (t, *J* = 5.1 Hz, 1H), 3.03/2.87 (2 x s, 3H) (mixture of rotamers).
^{13}C-NMR (101 MHz, $CDCl_3$): δ / ppm = 163.2, 163.0, 163.0, 162.8, 162.8, 141.6, 141.4, 125.7, 125.7, 125.7, 114.4, 114.3, 66.4, 65.4, 48.5, 43.7, 36.1, 29.9 (mixture of rotamers).
IR (Diamond-ATR, neat): $\tilde{\nu}$ / cm^{-1} = 1664, 1608, 1591, 1507, 1497, 1389, 1336, 1299, 1253, 1173, 1110, 1090, 1040, 906, 844, 751, 690.
MS (EI, 70 eV): *m/z* (%) = 165 (10), 86 (100), 72 (39), 58 (16), 44 (43).
HRMS (EI): *m/z* calc. for [$C_{10}H_{12}N_2O_4$]: 224.0797; found 224.0790.

4-(4-Formylpiperazin-1-yl)benzonitrile (4p)

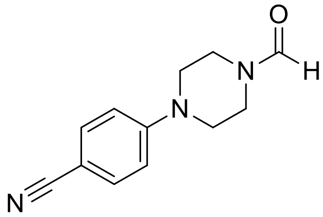

To a toluene solution of 4-fluorobenzonitrile (6.05 g, 50 mmol, 1.0 equiv) was added piperazine (43.07 g, 500 mmol, 10.0 equiv) in one portion and resulting suspension was heated under reflux (oil bath temperature 130 °C). During heating piperazine dissolved. After 12 h, heating was stopped, the warm reaction mixture was transferred into aqueous 2M NaOH (400 mL) and the product extracted with Et_2O (4x700 mL). Organic layers were combined, dried over

$MgSO_4$, filtered and the solvent removed *in vacuo* to obtain 4-(piperazin-1-yl)benzonitrile. This crude product was used in the following step without further purification.

The flask was heat dried under Ar-atmosphere. Acetic acid anhydride (12.0 mL, 127 mmol, 2.5 equiv) and formic acid (6.00 mL, 159 mmol, 3.2 equiv) were mixed in THF (100 mL) and heated at 40 °C for 1 h. The reaction mixture was cooled to −10 °C (ice/$NaCl_{(aq)}$). Crude 4-(piperazin-1-yl)benzonitrile was dissolved in THF (50 mL), dropwise added to the reaction mixture and stirred for 2 h at −10 °C. The white suspension was neutralized with sat. Na_2CO_3 (400 mL). Complete consumption of the starting material and the product formation was verified by GC/MS analysis. The product was extracted with Et_2O (4x700 mL), combined organic layers dried over $MgSO_4$, filtered and the solvent removed *in vacuo*. After a flash column chromatography with ethyl acetate:pentane = 1:1 (1 L), followed by pure ethyl acetate (3 L), 4-(4-formylpiperazin-1-yl)benzonitrile (8.14 g, 37.9 mmol, 76% yield over 2 steps) was obtained as a white crystalline solid.

^{1}H-NMR (400 MHz, $CDCl_3$): δ / ppm = 8.04 (s, 1 H), 7.42 (d, *J* = 9.0 Hz, 2 H), 6.83 (d, *J* = 9.0 Hz, 2 H), 3.61 (dd, *J* = 6.6, 5.1 Hz, 2 H), 3.47 (dd, *J* = 6.9, 4.9 Hz, 2 H), 3.30 (dd, *J* = 5.4, 3.4 Hz, 2 H), 3.25 (dd, *J* = 5.5, 3.5 Hz, 2 H).
^{13}C-NMR (101 MHz, $CDCl_3$): δ / ppm = 160.6, 152.9, 133.4, 119.6, 114.9, 101.1, 48.1, 46.9, 44.7, 39.3.
IR (Diamond-ATR, neat): $\tilde{\nu}$ / cm^{-1} = 2846, 2210, 1666, 1600, 1514, 1436.
MS (EI, 70 eV): *m/z* (%) = 215 (39), 186 (24), 157 (81), 145 (41), 144 (100), 130 (10), 129 (93), 116 (13).
HRMS (EI): *m/z* calc. for [$C_{12}H_{13}N_3O$]: 215.1059; 215.1052.
M.p. (°C): 144.2-146.3.
X-Ray: Crystals suitable for X-Ray diffraction were obtained from $CDCl_3$ by slow evaporation of the solvent at rt. See pages 137-140.

(1*R*,3*r*,5*S*)-3-((Triethylsilyl)oxy)-8-azabicyclo[3.2.1]octane-8-carbaldehyde (4q)

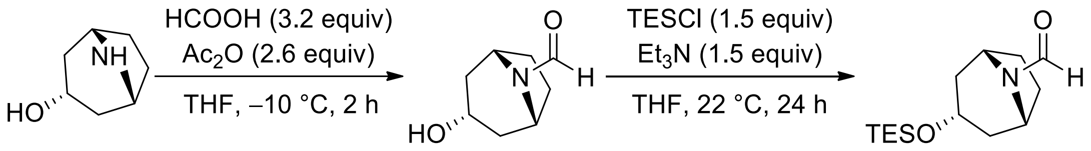

INT-4q1

The flask was heat dried under Ar-atmosphere. Acetic acid anhydride (4.92 mL, 52.0 mmol, 2.6 equiv) and formic acid (2.41 mL, 64 mmol, 3.2 equiv) were mixed in THF (50 mL) and heated at 40 °C for 1 h. The reaction mixture was cooled to −10 °C (ice/$NaCl_{(aq)}$). Nortropine (2.54 g, 20 mmol, 1.0 equiv) was added as a suspension in THF (50 mL) and stirred for 2 h at −10 °C. The white suspension was neutralized with aqueous 2M NaOH (150 mL). Complete consumption of starting material and product formation was verified by GC/MS analysis. The product was extracted with CH_2Cl_2 (3 x 300 mL), combined organic layers dried over $MgSO_4$, filtered and the solvent removed *in vacuo*. After a flash column chromatography on SiO_2 with CH_2Cl_2:EtOH=95:5, followed by CH_2Cl_2:MeOH=90:10 (1*R*,3*r*,5*S*)-3-hydroxy-8-azabicyclo[3.2.1]octane-8-carbaldehyde (2.64 g, 19.4 mmol, 97% yield) was obtained as a white solid.

^{1}H-NMR (400 MHz, $CDCl_3$): δ / ppm = 8.06 (s, 1H), 4.56 – 4.50 (m, 1H), 4.17 – 4.11 (m, 1H), 4.03 – 3.97 (m, 1H), 2.47 (d, *J* = 10.0 Hz, 1H), 2.30 – 2.21 (m, 2H), 2.09 – 2.03 (m, 1H), 1.99 – 1.93 (m, 2H), 1.93 – 1.83 (m, 3H), 1.83 – 1.78 (m, 1H).
^{13}C-NMR (101 MHz, $CDCl_3$): δ / ppm = 157.4, 64.9, 54.1, 49.2, 41.4, 38.9, 28.3, 27.7.

IR (Diamond-ATR, neat): $\tilde{\nu}$ / cm^{-1} = 3341, 1634, 1448, 1399, 1360, 1310, 1085, 1047, 958, 902, 818, 778, 740, 691, 662.
MS (EI, 70 eV): *m/z* (%) = 155 (46), 127 (14), 126 (38), 126 (12), 112 (42), 110 (20), 108 (12), 99 (21), 98 (21), 97 (10), 96 (13), 95 (12), 84 (13), 83 (23), 82 (69), 80 (16), 69 (31), 68 (100), 67 (18), 56 (12), 55 (12), 54 (10), 54 (16), 43 (12), 41 (25).
HRMS (EI): *m/z* calc. for [$C_8H_{13}NO_2$]: 155.0946; found 155.0940.
M.p. (°C): 136.3-138.0.

(1*R*,3*r*,5*S*)-3-Hydroxy-8-azabicyclo[3.2.1]octane-8-carbaldehyde (2.33 g, 15.0 mmol, 1.0 equiv), TESCl (3.39 g, 22.5 mmol, 1.5 equiv) and Et_3N (2.28 g, 22.5 mmol, 1.5 equiv) were mixed in THF (30 mL) at 22 °C and stirred for 24 h. The reaction mixture was quenched with distilled water (100 mL) and the product was extracted with CH_2Cl_2 (3 x 300 mL). Combined organic layers were dried over $MgSO_4$, filtered and the solvent was removed *in vacuo*. After a flash column chromatography on SiO_2 with pentane:ethyl acetate = 1:1, (1R,3r,5S)-3-((triethylsilyl)oxy)-8-azabicyclo[3.2.1]octane-8-carbaldehyde (3.68 g, 13.6 mmol, 91% yield) was obtained as a colorless liquid.

^{1}H-NMR (400 MHz, $CDCl_3$): δ / ppm = 8.08 (s, 1H), 4.58 – 4.50 (m, 1H), 4.10 – 4.04 (m, 1H), 4.01 – 3.96 (m, 1H), 2.36 – 2.29 (m, 2H), 2.07 – 1.99 (m, 1H), 1.96 – 1.90 (m, 1H), 1.88 – 1.81 (m, 3H), 1.80 – 1.73 (m, 1H), 0.98 – 0.92 (m, 9H), 0.61 – 0.53 (m, 6H).
^{13}C-NMR (101 MHz, $CDCl_3$): δ / ppm = 157.3, 65.5, 54.4, 49.4, 42.2, 39.5, 28.3, 27.6, 7.0, 4.8.
IR (Diamond-ATR, neat): $\tilde{\nu}$ / cm^{-1} = 2951, 2912, 2875, 1665, 1458, 1429, 1371, 1318, 1308, 1298, 1238, 1224, 1167, 1081, 1051, 1003, 972, 923, 859, 836, 788, 723, 685.
MS (EI, 70 eV): *m/z* (%) = 269 (37), 241 (15), 240 (100), 212 (14), 198 (14), 171 (50), 143 (11), 138 (12), 110 (12), 103 (49), 93 (18), 87 (13), 82 (13), 75 (55), 70 (77), 68 (19), 67 (12), 59 (15), 46 (15), 40 (10).
HRMS (EI): *m/z* calc. for [$C_{12}H_{23}NO$]: 269.1811; found 270.1820 (M+H).

Preparation of products

***N,N*-Dibutylbut-3-enamide (8a)**

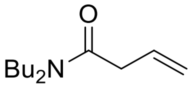

Following **TP1A**, *N,N*-dibutylformamide (**4a**) (157 mg, 1.00 mmol, 1.0 equiv), $ZnCl_2$ (500 µL, 0.500 mmol, 0.50 equiv) and Et_3N (56.0 mg, 0.500 mmol, 0.50 equiv) were mixed in THF (2.0 mL) and freshly prepared solution of TMPLi (1.10 mmol, 1.1 equiv) was dropwise added at 15 °C followed by allyl bromide (145 mg, 1.20 mmol, 1.2 equiv) and CuCN·2LiCl as 1 M solution in THF (100 µL, 0.100 mmol, 0.10 equiv). Thereafter, the reaction mixture was quenched with saturated $NH_4Cl_{(aq)}$ solution. After workup, the crude product was purified *via* column chromatography (pentane:Et_2O = 7:3) to give **8a** (185 mg, 0.940 mmol, 94% yield) as a colorless oil.

^{1}H-NMR (400 MHz, $CDCl_3$): δ / ppm = 5.98 (ddt, *J* = 16.9, 10.3, 6.6 Hz, 1H), 5.19 – 5.08 (m, 2H), 3.33 – 3.27 (m, 2H), 3.24 – 3.18 (m, 2H), 3.11 (dt, *J* = 6.6, 1.5 Hz, 2H), 1.59 – 1.45 (m, 4H), 1.38 – 1.22 (m, 5H), 0.99 – 0.89 (m, 5H).
^{13}C-NMR (101 MHz, $CDCl_3$): δ / ppm = 170.5, 132.4, 117.4, 48.0, 45.8, 38.8, 31.4, 30.0, 20.4, 20.3, 14.0, 14.0.
IR (Diamond-ATR, neat): $\tilde{\nu}$ / cm^{-1} = 2958, 2932, 2874, 1545, 1456, 1426, 1376, 1144, 1112, 1101, 994, 911.
MS (EI, 70 eV): *m/z* (%) = 198 (11), 182 (36), 168 (22), 156 (65), 154 (100), 140 (22), 126 (13), 113 (34), 112 (48), 100 (21), 86 (70), 57 (27), 44 (19).
HRMS (EI): *m/z* calc. for [$C_{12}H_{23}NO$]: 197.1780; found 198.1850 (M+H).

***N*-(2-((tert-Butyldimethylsilyl)oxy)ethyl)-*N*-methylbut-3-enamide (8b)**

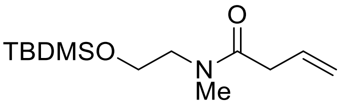

Following **TP1A**, *N*-(2-((tert-butyldimethylsilyl)oxy)ethyl)-N-methylformamide (**4b**) (217 mg, 1.0 equiv, 1.00 mmol, 1.0 equiv), $ZnCl_2$ (500 µL, 0.500 mmol, 0.50 equiv) and Et_3N (56.0 mg, 0.500 mmol, 0.5 equiv) were mixed in THF (2.0 mL) and a freshly prepared solution of TMPLi (1.10 mmol, 1.1 equiv) was dropwise added at 15 °C followed by allyl bromide (145 mg, 1.20 mmol, 1.2 equiv) and CuCN·2LiCl as 1 M solution in THF (100 µL, 0.100 mmol, 0.10 equiv). Thereafter, the reaction mixture was quenched with saturated $NH_4Cl_{(aq)}$ solution. After workup, the crude product was purified *via* column chromatography (pentane:ethyl acetate = 85:15) to give **8b** (206 mg, 0.800 mmol, 80% yield) as a colorless oil.

^{1}H-NMR (400 MHz, $CDCl_3$): δ / ppm = 6.04 – 5.87 (m, 1H), 5.19 – 5.04 (m, 2H), 3.78 – 3.68 (m, 2H), 3.46 (t, *J* = 5.4 Hz, 1H), 3.41 (t, *J* = 5.5 Hz, 1H), 3.21 (dt, *J* = 6.7, 1.6 Hz, 1H), 3.13 – 3.10 (m, 1H), 3.08/2.93 (2 x s, 3H), 0.87 (s, 9H), 0.03 (s, 3H), 0.02 (s, 3H).
^{13}C-NMR (101 MHz, $CDCl_3$): δ / ppm = 171.6, 171.0, 132.2, 131.6, 117.8, 117.5, 61.8, 60.7, 52.1, 50.9, 39.1, 38.7, 37.9, 33.9, 26.0, 25.9, 18.3, 18.3, -5.3, -5.4.
IR (Diamond-ATR, neat): $\tilde{\nu}$ / cm-1 = 2954, 2929, 2886, 2857, 1647, 1472, 1464, 1399, 1361, 1253, 1100, 1005, 921, 828, 811, 775, 733, 662.
MS (EI, 70 eV): *m/z* (%) = 200 (100), 132 (15).
HRMS (EI): *m/z* calc. for [$C_{13}H_{27}NO_2Si$]: 242.1576 (M-CH_3); found 242.1569 (M-CH_3).

1-Morpholinobut-3-en-1-one (8c)

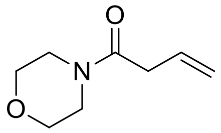

Following **TP1A**, morpholine-4-carbaldehyde (**4c**) (115 mg, 1.00 mmol, 1.0 equiv), $ZnCl_2$ (500 µL, 0.500 mmol, 0.50 equiv) and Et_3N (56.0 mg, 0.500 mmol, 0.50 equiv) were mixed in THF (2.0 mL) and a freshly prepared solution of TMPLi (1.10 mmol, 1.1 equiv) was dropwise added at 15 °C followed by allyl bromide (145 mg, 1.20 mmol, 1.2 equiv) and CuCN·2LiCl as 1 M solution in THF (100 µL, 0.100 mmol, 0.10 equiv). Thereafter, the reaction mixture was quenched with saturated $NH_4Cl_{(aq)}$ solution. After workup, the crude product was purified *via* column chromatography (pentane:Et_2O = 3:7) to give **8c** (109 mg, 0.700 mmol, 70% yield) as a colorless oil.

^{1}H-NMR (400 MHz, $CDCl_3$): δ / ppm = 6.03 – 5.86 (m, 1H), 5.23 – 5.07 (m, 2H), 3.70 – 3.59 (m, 6H), 3.50 – 3.41 (m, 2H), 3.14 (dt, *J* = 6.6, 1.6 Hz, 2H).
^{13}C-NMR (101 MHz, $CDCl_3$): δ / ppm = 169.6, 131.3, 118.1, 67.0, 66.7, 46.3, 42.1, 38.7.
IR (Diamond-ATR, neat): $\tilde{\nu}$ / cm-1 = 2858, 1645, 1436, 1300, 1272, 1255, 1228, 1197, 1114, 1069, 1038, 995, 968, 920, 850.
MS (EI, 70 eV): *m/z* (%) = 155 (16), 114 (100), 70 (29).
HRMS (EI): *m/z* calc. for [$C_8H_{13}NO_2$]: 155.0946; found 155.0940.

1-(4-Phenylpiperazin-1-yl)but-3-en-1-one (8d)

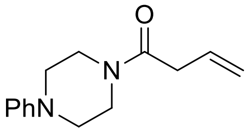

Following **TP1A**, 4-phenylpiperazine-1-carbaldehyde (**4d**) (190 mg, 1.00 mmol, 1.0 equiv), $ZnCl_2$ (500 µL, 0.500 mmol, 0.50 equiv) and Et_3N (56.0 mg, 0.500 mmol, 0.50 equiv) were mixed in THF (2.0 mL) and a freshly prepared solution of TMPLi (1.10 mmol, 1.1 equiv) was dropwise added at 15 °C followed by allyl bromide (145 mg, 1.20 mmol, 1.2 equiv) and CuCN·2LiCl as 1 M solution in THF (100 µL, 0.100 mmol, 0.10 equiv). Thereafter, the reaction mixture was quenched with saturated $NH_4Cl_{(aq)}$ solution. After workup, the crude product was purified *via* column chromatography (pentane:ethyl acetate = 7:3) to give **8d** (161 mg, 0.700 mmol, 70% yield) as a colorless oil.

^{1}H-NMR (400 MHz, $CDCl_3$): δ / ppm = 7.78 – 7.58 (m, 2H), 7.38 – 7.19 (m, 3H), 6.34 (ddt, *J* = 16.9, 10.2, 6.5 Hz, 1H), 5.67 – 5.45 (m, 2H), 4.20 – 4.11 (m, 2H), 4.03 – 3.95 (m, 2H), 3.59 – 3.54 (m, 2H), 3.56 – 3.49 (m, 4H).
^{13}C-NMR (101 MHz, $CDCl_3$): δ / ppm = 169.4, 151.0, 131.4, 129.3, 120.6, 118.0, 116.7, 49.8, 49.4, 45.8, 41.6, 38.8.
IR (Diamond-ATR, neat): $\tilde{\nu}$ / cm-1 = 2917, 2855, 2820, 1643, 1598, 1579, 1495, 1436, 1386, 1334, 1276, 1258, 1227, 1208, 1154, 1119, 1096, 1028, 992, 964, 916, 898, 799, 757, 692.
MS (EI, 70 eV): *m/z* (%) = 231 (12), 230 (68), 215 (15), 202 (15), 161 (29), 160 (21), 158 (12), 133 (14), 132 (100), 124 (12), 120 (69), 119 (70), 106 (28), 105 (43), 104 (54), 91 (28), 69 (11), 56 (68), 41 (16), 40 (52).
HRMS (EI): *m/z* calc. for [C9H5F3OS]: 230.1419; found 230.1420.

1-(*trans*-Octahydroquinolin-1(2H)-yl)but-3-en-1-one (8e)

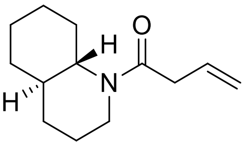

Following **TP1A**, *trans*-octahydroisoquinoline-2(1H)-carbaldehyde (**4e**) (167 mg, 1.00 mmol, 1.0 equiv), $ZnCl_2$ (500 µL, 0.500 mmol, 0.50 equiv) and Et_3N (56.0 mg, 0.500 mmol, 0.50 equiv) were mixed in THF (2.0 mL) and freshly prepared solution of TMPLi (1.10 mmol, 1.1 equiv) was dropwise added at 15 °C followed by allyl bromide (145 mg, 1.20 mmol, 1.2 equiv) and CuCN·2LiCl as 1 M solution in THF (100 µL, 0.100 mmol, 0.10 equiv). Thereafter, the reaction mixture was quenched with NH_4Cl. After workup, the crude product was purified *via* column chromatography (pentane:ethyl acetate = 85:15) to give **8e** (201 mg, 0.700 mmol, 97% yield) as a colorless oil.

^{1}H-NMR (400 MHz, $CDCl_3$): δ / ppm = 6.03 – 5.91 (m, 1H), 5.15 – 5.07 (m, 2H), 3.82 – 3.50 (m, 1H), 3.39 – 3.27 (m, 1H), 3.23 – 3.05 (m, 3H), 2.14 – 2.05 (m, 1H), 1.84 – 1.73 (m, 2H), 1.72 – 1.61 (m, 3H), 1.60 – 1.50 (m, 2H), 1.47 – 1.37 (m, 1H), 1.33 – 1.21 (m, 2H), 1.17 – 1.00 (m, 2H).
^{13}C-NMR (101 MHz, $CDCl_3$): δ / ppm = 170.3 , 132.3 , 117.4 , 61.5 , 39.6 , 38.4 , 33.1 , 31.0 , 26.4 , 26.0 , 25.5 , 23.0.
IR (Diamond-ATR, neat): $\tilde{\nu}$ / cm-1 = 2923, 2886, 2855, 1628, 1422, 1362, 1287, 1267, 1223, 1172, 1137, 1008, 994, 911.
MS (EI, 70 eV): *m/z* (%) = 192 (20), 167 (10), 166 (92), 164 (27), 138 (17), 136 (11), 96 (100), 86 (14), 81 (22), 79 (16), 67 (11).
HRMS (EI): *m/z* calc. for [$C_{13}H_{21}NO$]: 207.1623; found 207.1617.

1-(4-(3-(Trifluoromethyl)pyridin-2-yl)piperazin-1-yl)but-3-en-1-one (8f)

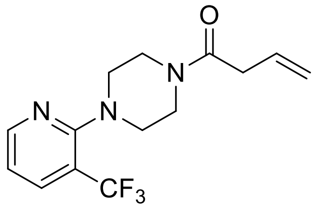

Following **TP1A**, 4-(3-(trifluoromethyl)pyridin-2-yl)piperazine-1-carbaldehyde (**4f**) (259 mg, 1.00 mmol, 1.0 equiv), $ZnCl_2$ (500 µL, 0.500 mmol, 0.50 equiv) and Et_3N (56.0 mg, 0.500 mmol, 0.50 equiv) were mixed in THF (2.0 mL) and a freshly prepared solution of TMPLi (1.10 mmol, 1.1 equiv) was dropwise added at 15 °C followed by allyl bromide (145 mg, 1.20 mmol, 1.2 equiv) and CuCN·2LiCl as 1 M solution in THF (100 µL, 0.100 mmol, 0.10 equiv). Thereafter, the reaction mixture was quenched with saturated $NH_4Cl_{(aq)}$ solution. After workup, the crude product was purified *via* column chromatography (pentane:ethyl acetate = 7:3) to give **8f** (230 mg, 0.770 mmol, 77% yield) as a yellow oil.

^{1}H-NMR (400 MHz, $CDCl_3$): δ / ppm = 8.43 (dd, *J* = 4.9, 1.8 Hz, 1H), 7.87 (dd, *J* = 7.8, 1.9 Hz, 1H), 7.09 – 6.98 (m, 1H), 5.95 (ddt, *J* = 16.9, 10.3, 6.5 Hz, 1H), 5.24 – 5.09 (m, 2H), 3.84 – 3.70 (m, 2H), 3.64 – 3.54 (m, 2H), 3.29 – 3.13 (m, 6H).
^{13}C-NMR (101 MHz, $CDCl_3$): δ / ppm = 169.7 , 159.7 , 151.3 , 137.3 , 131.4 , 123.9 (q, *J* = 272.5 Hz), 118.0, 118.0, 117.9 (q, *J* = 31.6 Hz), 50.9 , 50.8 , 45.9 , 41.8 , 38.9 .
^{19}F-NMR (377 MHz, CDCl3): –60.2.
IR (Diamond-ATR, neat): $\tilde{\nu}$ / cm-1 = 1646, 1591, 1568, 1436, 1369, 1308, 1248, 1223, 1144, 1102, 1082, 1024, 993, 974, 917, 793, 777.
MS (EI, 70 eV): *m/z* (%) = 213 (11), 201 (25), 189 (27), 188 (22), 187 (100), 175 (35), 173 (17), 169 (11), 167 (13), 155 (22), 149 (11), 147 (11), 146 (15), 128 (33).
HRMS (EI): *m/z* calc. for [$C_{14}H_{16}F_3N_3O$]: 299.1245; found 299.1244.

Cyclohex-2-en-1-yl(7,7,9,9-tetramethyl-1,4-dioxa-8-azaspiro[4.5]decan-8-yl)methanone (8g)

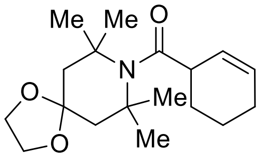

Following modified **TP1A**, 7,7,9,9-tetramethyl-1,4-dioxa-8-azaspiro[4.5]decane-8-carbaldehyde (**4h**) (227 mg, 1.00 mmol, 1.0 equiv), $ZnCl_2$ (500 µL, 0.500 mmol, 0.50 equiv) and Et_3N (56.0 mg, 0.500 mmol, 0.50 equiv) were mixed in THF (2.0 mL) and a freshly prepared solution of TMPLi (2.20 mmol, 2.2 equiv) was dropwise added at 15 °C followed by 3-bromocyclohex-1-ene (320 mg, 2.00 mmol, 2.0 equiv) and CuCN·2LiCl as 1 M solution in THF (200 µL, 0.200 mmol, 0.20 equiv). Thereafter, the reaction mixture was quenched with saturated $NH_4Cl_{(aq)}$ solution. After workup, the crude product was purified *via* column chromatography (pentane:ethyl acetate = 95:5) to give **8g** (206 mg, 0.670 mmol, 67% yield) as a yellow solid.

^{1}H-NMR (400 MHz, $CDCl_3$): δ / ppm = 5.86 – 5.80 (m, 1H), 5.55 – 5.49 (m, 1H), 3.92 – 3.83 (m, 4H), 3.48 – 3.41 (m, 1H), 2.14 – 2.03 (m, 3H), 2.00 – 1.93 (m, 3H), 1.89 – 1.80 (m, 2H), 1.79 – 1.71 (m, 1H), 1.56 – 1.41 (m, 13H).
^{13}C-NMR (101 MHz, $CDCl_3$): δ / ppm = 181.0, 129.5, 126.0, 105.8, 63.7, 55.7, 49.2, 45.5, 30.6, 29.9, 27.2, 24.7, 21.5.
IR (Diamond-ATR, neat): $\tilde{\nu}$ / cm-1 = 2967, 2934, 2361, 1635, 1458, 1384, 1368, 1338, 1306, 1269, 1269, 1217, 1168, 1139, 1102, 979, 956, 922, 900, 844, 816, 777, 740, 718.
MS (EI, 70 eV): *m/z* (%) = 184 (27), 170 (100), 114 (17), 98 (24), 87 (45), 84 (23).
HRMS (EI): *m/z* calc. for [$C_{18}H_{29}NO_3$]: 292.1913 (M-CH_3); found 292.1909 (M-CH_3).
M.p. (°C): 68.7-71.8.

(*S*)-1-(2-(Methoxymethyl)pyrrolidin-1-yl)-4-methylpent-3-en-1-one (8h)

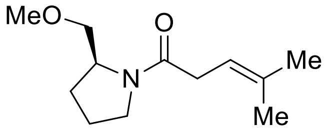

Following **TP1A**, (S)-2-(methoxymethyl)pyrrolidine-1-carbaldehyde (**4g**) (143 mg, 1.00 mmol, 1.0 equiv), $ZnCl_2$ (500 µL, 0.500 mmol, 0.50 equiv) and Et_3N (56.0 mg, 0.500 mmol, 0.50 equiv) were mixed in THF (2.0 mL) and a freshly prepared solution of TMPLi (1.10 mmol, 1.1 equiv) was dropwise added at 15 °C followed by prenyl bromide (164 mg, 1.10 mmol, 1.1 equiv) and CuCN·2LiCl as 1 M solution in THF (100 µL, 0.100 mmol, 0.10 equiv). Thereafter, the reaction mixture was quenched with saturated $NH_4Cl_{(aq)}$ solution. After workup, the crude product was purified *via* column chromatography (pentane:ethyl acetate = 1:1) to give **8h** (129 mg, 0.610 mmol, 61% yield) as a colorless oil.

^{1}H-NMR (400 MHz, CDCl3): δ / ppm = 5.35 – 5.28 (m, 1H), 4.25 – 4.18/4.05 – 3.98 (2 x m, 1H), 3.56 – 3.49 (m, 1H), 3.49 – 3.41 (m, 1H), 3.40 – 3.29 (m, 5H), 3.13 – 3.08/3.01 – 2.96 (2 x m, 1H), 2.04 – 1.91 (m, 2H), 1.91 – 1.80 (m, 2H), 1.74 – 1.69 (m, 3H), 1.66 – 1.59 (m, 3H) (mixture of rotamers).
^{13}C-NMR (101 MHz, $CDCl_3$): δ / ppm = 171.4, 171.0, 134.6, 134.3, 117.7, 117.0, 74.3, 72.4, 59.3, 59.1, 57.1, 56.5, 47.4, 45.8, 35.1, 34.3, 28.9, 27.5, 25.8, 24.3, 22.0, 18.2 (mixture of rotamers).
IR (Diamond-ATR, neat): $\tilde{\nu}$ / cm-1 = 2973, 2926, 2877, 1638, 1410, 1376, 1343, 1273, 1196, 1170, 1110, 1074, 970, 942, 900, 852, 792, 711.
MS (EI, 70 eV): *m/z* (%) = 179 (16), 167 (10), 166 (100), 142 (11), 114 (18), 82 (18), 70 (93),

HRMS (EI): *m/z* calc. for [$C_{12}H_{21}NO_2$]: 211.1572; found 211.1565.

4-Methyl-1-morpholinopent-3-en-1-one (8i)

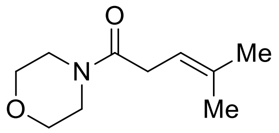

Following **TP1A**, morpholine-4-carbaldehyde (**4c**) (115 mg, 1.00 mmol, 1.0 equiv), $ZnCl_2$ (500 µL, 0.500 mmol, 0.50 equiv) and Et_3N (56.0 mg, 0.500 mmol, 0.50 equiv) were mixed in THF (2.0 mL) and a freshly prepared solution of TMPLi (1.10 mmol, 1.1 equiv) was dropwise added at 15 °C followed by prenyl bromide (164 mg, 1.10 mmol, 1.1 equiv) and CuCN·2LiCl as 1 M solution in THF (100 µL, 0.100 mmol, 0.10 equiv). Thereafter, the reaction mixture was quenched with saturated $NH_4Cl_{(aq)}$ solution. After workup, the crude product was purified *via* column chromatography (pentane:ethyl acetate = 3:7) to give **8i** (113 mg, 0.620 mmol, 62% yield) as a colorless oil.

^{1}H-NMR (400 MHz, $CDCl_3$): δ / ppm = 5.28 – 5.15 (m, 1H), 3.65 – 3.58 (m, 4H), 3.57 – 3.52 (m, 2H), 3.43 – 3.37 (m, 2H), 3.05 – 2.98 (m, 2H), 1.73 – 1.66 (m, 2H), 1.63 – 1.57 (m, 3H).
^{13}C-NMR (101 MHz, $CDCl_3$): δ / ppm = 170.7, 135.0, 116.6, 66.9, 66.7, 46.2, 42.0, 33.5, 25.7, 18.1.
IR (Diamond-ATR, neat): $\tilde{\nu}$ / cm-1 = 2970, 2921, 2857, 1621, 1435, 1377, 1361, 1300, 1270, 1232, 1155, 1115, 1069, 1045, 1023, 968, 848.
MS (EI, 70 eV): *m/z* (%) = 183 (12), 182 (28), 114 (22), 96 (28), 95 (13), 86 (36), 70 (26), 69 (17), 67 (10), 57 (14), 56 (28), 45 (13), 43 (100), 42 (12), 41 (16).
HRMS (EI): *m/z* calc. for [$C_{10}H_{17}NO_2$]: 183.1259; found 183.1253.

(*E*)-1-Morpholino-4-phenylbut-3-en-1-one (8j)

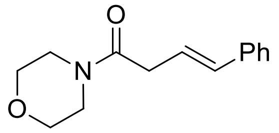

Following **TP1A**, morpholine-4-carbaldehyde (**4c**) (115 mg, 1.00 mmol, 1.0 equiv), $ZnCl_2$ (500 µL, 0.500 mmol, 0.50 equiv) and Et_3N (56.0 mg, 0.500 mmol, 0.50 equiv) were mixed in THF (2.0 mL) and a freshly prepared solution of TMPLi (1.10 mmol, 1.1 equiv) was dropwise added at 15 °C followed by prenyl bromide (164 mg, 1.10 mmol, 1.1 equiv) and CuCN·2LiCl as 1 M solution in THF (100 µL, 0.100 mmol, 0.10 equiv). Thereafter, the reaction mixture was quenched with saturated $NH_4Cl_{(aq)}$ solution. After workup, the crude product was purified *via* column chromatography (pentane:ethyl acetate = 6:4) to give **8j** (132 mg, 0.570 mmol, 57% yield) as a yellow solid.

^{1}H-NMR (400 MHz, $CDCl_3$): δ / ppm = 7.39 – 7.34 (m, 2H), 7.34 – 7.28 (m, 2H), 7.25 – 7.20 (m, 1H), 6.51 – 6.45 (m, 1H), 6.36 – 6.29 (m, 1H), 3.71 – 3.63 (m, 6H), 3.53 – 3.48 (m, 2H), 3.30 (dd, *J* = 6.7, 1.6 Hz, 2H).
^{13}C-NMR (101 MHz, $CDCl_3$): δ / ppm = 169.7, 136.9, 133.1, 128.7, 127.7, 126.3, 122.8, 66.8, 46.4, 42.2, 37.9.
IR (Diamond-ATR, neat): $\tilde{\nu}$ / cm-1 = 2950, 2919, 2865, 1638, 1598, 1459, 1434, 1404, 1390, 1361, 1303, 1272, 1228, 1196, 1113, 1069, 1032, 954, 937, 908, 858, 838, 778, 743, 698.
MS (EI, 70 eV): *m/z* (%) = 231 (22), 117 (25), 116 (10), 115 (33), 114 (100), 105 (15), 91 (13), 86 (13), 70 (67), 42 (21), 41 (15).
HRMS (EI): *m/z* calc. for [$C_{14}H_{17}NO_2$]: 231.1259; found 231.1253.
M.p. (°C): 94.9-95.2.

X-Ray: Crystals suitable for X-Ray diffraction were obtained by recrystalisation from Et_2O (complete dissolution on reflux). See pages 141-144.

1-(Piperidin-1-yl)buta-2,3-dien-1-one (8k)

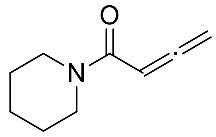

Following **TP1A**, piperidine-1-carbaldehyde (**4i**) (113 mg, 1.00 mmol, 1.0 equiv), $ZnCl_2$ (500 µL, 0.500 mmol, 0.50 equiv) and Et_3N (56.0 mg, 0.500 mmol, 0.50 equiv) were mixed in THF (2.0 mL) and a freshly prepared solution of TMPLi (1.10 mmol, 1.1 equiv) was dropwise added at 15 °C followed by propargyl bromide 80% in toluene (164 mg, 1.10 mmol, 1.1 equiv) and CuCN·2LiCl as 1 M solution in THF (100 µL, 0.100 mmol, 0.10 equiv). Thereafter, the reaction mixture was quenched with saturated $NH_4Cl_{(aq)}$ solution. After workup, the crude product was purified *via* column chromatography (pentane:ethyl acetate = 7:3) to give **8k** (88 mg, 0.580 mmol, 58% yield) as a yellow liquid.

^{1}H-NMR (400 MHz, CDCl$_3$): δ / ppm = 5.90 (t, *J* = 6.6 Hz, 1H), 5.09 (d, *J* = 6.6 Hz, 2H), 3.52 (dt, *J* = 33.3, 5.4 Hz, 4H), 1.66 – 1.49 (m, 6H).
^{13}C-NMR (101 MHz, CDCl$_3$): δ / ppm = 213.2, 163.5, 87.3, 78.6, 47.8, 43.4, 26.6, 25.6, 24.6.
IR (Diamond-ATR, neat): $\tilde{\nu}$ / cm-1 = 2935, 2855, 1946, 1615, 1440, 1413, 1367, 1351, 1250, 1216, 1138, 1124, 1012, 952, 849, 805, 756.
MS (EI, 70 eV): *m/z* (%) = 150 (100), 123 (20), 122 (24), 112 (20), 108 (15), 108 (13), 94 (14), 84 (21), 82 (45), 69 (25), 67 (43).
HRMS (EI): *m/z* calc. for [$C_9H_{13}NO$]: 151.0997; found 151.0945.

Ethyl 4-(dibutylamino)-2-methylene-4-oxobutanoate-4-[^{13}C] (8l)

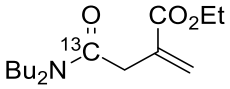

Following **TP1A**, *N*,*N*-dibutyl[^{13}C]formamide (**4a**) (158 mg, 1.00 mmol, 1.0 equiv), $ZnCl_2$ (500 µL, 0.500 mmol, 0.50 equiv) and Et_3N (56.0 mg, 0.500 mmol, 0.50 equiv) were mixed in THF (2.0 mL) and a freshly prepared solution of TMPLi (1.10 mmol, 1.1 equiv) was dropwise added at 15 °C followed by ethyl 2-(bromomethyl)acrylate (211 mg, 1.10 mmol, 1.1 equiv) and CuCN·2LiCl as 1 M solution in THF (100 µL, 0.100 mmol, 0.10 equiv). Thereafter, the reaction mixture was quenched with saturated $NH_4Cl_{(aq)}$ solution. After workup, the crude product was purified *via* column chromatography (pentane:ethyl acetate = 9:1) to give **8l** (170 mg, 0.630 mmol, 63% yield) as a yellow liquid.

^{1}H-NMR (400 MHz, CDCl$_3$): δ / ppm = 6.27 (s, 1H), 5.62 (s, 1H), 4.19 (q, *J* = 7.1 Hz, 2H), 3.34 – 3.26 (m, 4H), 3.25 – 3.19 (m, 2H), 1.61 – 1.53 (m, 2H), 1.53 – 1.45 (m, 2H), 1.36 – 1.25 (m, 7H), 0.94 (t, *J* = 7.4 Hz, 3H), 0.89 (t, *J* = 7.4 Hz, 3H) (mixture of rotamers).
^{13}C-NMR (101 MHz, CDCl$_3$): δ / ppm = 169.6, 166.7, 166.7, 135.5, 135.5, 127.2, 127.1, 77.2, 60.97, 48.1, 48.1, 46.0, 36.9, 36.5, 31.3, 29.9, 20.3, 20.2, 14.2, 14.0, 13.9 (mixture of rotamers).
IR (Diamond-ATR, neat): $\tilde{\nu}$ / cm-1 = 2959, 2932, 2874, 1717, 1602, 1444, 1420, 1370, 1334, 1317, 1300, 1254, 1198, 1174, 1134, 1114, 1028, 939, 861, 821, 734.
MS (EI, 70 eV): *m/z* (%) = 225 (12), 197 (29), 157 (11), 122 (17), 142 (30), 114 (100), 86 (16), 85 (53).
HRMS (EI): *m/z* calc. for [$C_9H_{13}NO$]: 270.2024; found 271.2098 (M+H).

Ethyl 4-(2-(*N*-methylcyclohex-2-enecarboxamido)ethoxy)benzoate (8m)

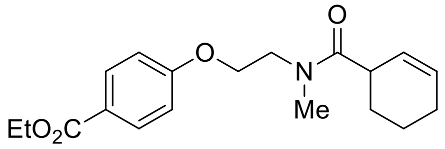

Following **TP1B**, ethyl 4-(2-(*N*-methylformamido)ethoxy)benzoate (**4k**) (251 mg, 1.00 mmol, 1.0 equiv) and $TMP_2Zn\cdot 2MgCl_2\cdot 2LiCl$ 0.33 M in THF (1.82 mL, 0.600 mmol, 0.6 equiv) were stirred at 22 °C for 24 h. 3-Bromocyclohex-1-ene (193 mg, 1.20 mmol, 1.2 equiv) and CuCN·2LiCl as 1 M solution in THF (100 µL, 0.100 mmol, 0.10 equiv) were added at 22 °C. After 16 h, the reaction mixture was quenched with saturated $NH_4Cl_{(aq)}$ solution. After workup, the crude product was purified *via* column chromatography (pentane:ethyl acetate = 6:4) to give **8m** (257 mg, 0.780 mmol, 78% yield) as a colorless oil.

^{1}H-NMR (400 MHz, $CDCl_3$): δ / ppm = 7.95 – 7.88 (m, 2H), 6.87 – 6.78 (m, 2H), 5.85 – 5.75 (m, 1H), 5.57 – 5.46 (m, 1H), 4.32 – 4.21 (m, 2H), 4.16 – 4.05 (m, 2H), 3.80 – 3.64 (m, 2H), 3.53 – 3.45/3.34 – 3.25 (2 x m, 1H), 3.14/2.94 (2 x s, 3H), 2.09 – 1.87 (m, 2H), 1.87 – 1.64 (m, 3H), 1.57 – 1.43 (m, 1H), 1.34 – 1.25 (m, 3H) (mixture of rotamers).
^{13}C-NMR (101 MHz, $CDCl_3$): δ / ppm = 174.8, 166.1, 162.2, 131.4, 129.6, 124.6, 123.0, 113.9, 66.5, 60.5, 48.0, 38.8, 37.4, 25.3, 24.5, 20.9, 14.3 (mixture of rotamers).
IR (Diamond-ATR, neat): $\tilde{\nu}$ / cm-1 = 2935, 1798, 1639, 1605, 1581, 1510, 1463, 1420, 1401, 1366, 1314, 1274, 1248, 1167, 1167, 1144, 1101, 1046, 1018, 922, 900, 848, 770, 730, 696, 666.
MS (EI, 70 eV): *m/z* (%) =167.1 (11), 166.1 (100), 138 (10), 81 (11).
HRMS (EI): *m/z* calc. for [$C_{19}H_{25}NO_4$]: 331.1784; found 331.1778.

N-(2-(2-Benzoylphenoxy)ethyl)-*N*,3-dimethylbut-3-enamide (8n)

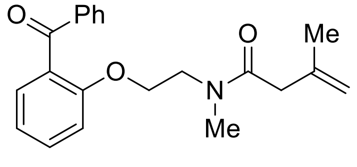

Following **TP1B**, *N*-(2-(2-benzoylphenoxy)ethyl)-*N*-methylformamide (**4l**) (283 mg, 1.00 mmol, 1.0 equiv) and $TMP_2Zn\cdot 2MgCl_2\cdot 2LiCl$ 0.33 M in THF (1.67 mL, 0.55 mmol, 0.55 equiv) were stirred at 22 °C for 24 h. 3-Bromo-2-methylprop-1-ene (148 mg, 1.10 mmol, 1.1 equiv) and CuCN·2LiCl as 1 M solution in THF (100 µL, 0.100 mmol, 0.10 equiv) were added at 22 °C. After 30 min, the reaction mixture was quenched with saturated $NH_4Cl_{(aq)}$ solution. After workup, the crude product was purified *via* column chromatography (pentane:ethyl acetate = 6:4) to give **8n** (181 mg, 0.540 mmol, 54% yield) as a colorless oil.

^{1}H-NMR (400 MHz, $CDCl_3$): δ / ppm = 7.80 – 7.74 (m, 2H), 7.56 – 7.51 (m, 1H), 7.46 – 7.37 (m, 3H), 7.36 – 7.32 (m, 1H), 7.09 – 7.00 (m, 1H), 6.96 – 6.91 (m, 1H), 4.81 (dd, *J* = 1.9, 1.0 Hz, 1H), 4.66 – 4.55 (m, 1H), 4.06 (2, *J* = 4.9 Hz, 2H), 3.41 (t, *J* = 4.9 Hz, 2H), 2.90 (s, 1H), 2.70/2.57 (2 x s, 1H), 2.57 (s, 2H), 1.69/1.66 (2 x s, 1H) (mixture of rotamers).
^{13}C-NMR (101 MHz, $CDCl_3$): δ / ppm = 196.5, 170.9, 156.3, 139.2, 137.9, 133.0, 132.1, 129.8, 129.6, 128.8, 128.3, 120.9, 113.3, 112.0, 67.4, 48.0, 43.2, 37.7, 22.6.
IR (Diamond-ATR, neat): $\tilde{\nu}$ / cm-1 = 1638, 1598, 1580, 1486, 1449, 1399, 1316, 1295, 1262, 1238, 1152, 1111, 1050, 1025, 925, 895, 804, 753, 728, 701.
MS (EI, 70 eV): *m/z* (%) = 197 (17), 152 (11), 140 (100), 124 (18), 121 (11), 105 (10), 91 (12), 83 (52), 77 (11).

HRMS (EI): *m/z* calc. for [$C_{21}H_{23}NO_3$]: 337.1678; found 337.1668.

N-(3-(1,3-Dioxoisoindolin-2-yl)propyl)-*N*-isopropyl-3-methylbut-3-enamide (8o)

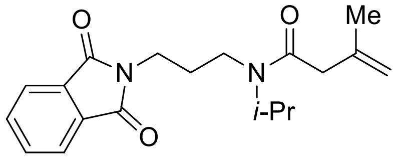

Following **TP1B**, *N*-(3-(1,3-dioxoisoindolin-2-yl)propyl)-*N*-isopropylformamide (**4m**) (274 mg, 1.00 mmol, 1.0 equiv), and $TMP_2Zn{\cdot}2MgCl_2{\cdot}2LiCl$ 0.33 M in THF (1.67 mL, 0.55 mmol, 0.55 equiv) were stirred at 22 °C for 24 h. 3-Bromo-2-methylprop-1-ene (148 mg, 1.10 mmol, 1.1 equiv) and CuCN·2LiCl as 1 M solution in THF (100 µL, 0.100 mmol, 0.10 equiv) were added at 22 °C. After 30 min, the reaction mixture was quenched with saturated $NH_4Cl_{(aq)}$ solution. After workup, the crude product was purified *via* column chromatography (pentane:ethyl acetate = 1:1) to give **8q** (155 mg, 0.470 mmol, 47% yield) as a colorless oil.

^{1}H-NMR (400 MHz, CDCl$_3$): δ / ppm = 7.77 – 7.74 (m, 2H), 7.65 – 7.62 (m, 2H), 4.84 – 4.80 (m, 1H), 4.71 – 4.67 (m, 1H), 3.95 (hept, *J* = 6.7 Hz, 1H), 3.65 (q, *J* = 6.8 Hz, 2H), 3.24 – 3.17 (m, 2H), 3.02 (s, 2H), 1.99 – 1.84 (m, 2H), 1.74 – 1.68 (m, 3H), 1.09 (d, *J* = 1.1 Hz, 6H) (major rotamer).
7.82 – 7.75 (m, 2H), 7.72 – 7.64 (m, 2H), 4.78 – 4.71 (m, 1H), 4.63 – 4.58 (m, 1H), 4.52 (hept, *J* = 6.8 Hz, 1H), 3.65 (q, *J* = 6.8 Hz, 2H), 3.17 – 3.10 (m, 2H), 2.94 (s, 2H), 1.97 – 1.85 (m, 2H), 1.67 – 1.60 (m, 3H), 1.07 (d, *J* = 1.2 Hz, 6H) (minor rotamer).
^{13}C-NMR (101 MHz, CDCl$_3$): δ / ppm = 170.1, 170.0, 168.3, 168.2, 140.0, 139.9, 134.2, 133.9, 132.0, 131.8, 123.3, 123.1, 112.9, 112.9, 77.2, 48.7, 45.9, 43.8, 43.8, 41.4, 38.5, 36.2, 35.6, 30.3, 28.8, 22.6, 22.6, 21.2, 20.4 (mixture of rotamers).
IR (Diamond-ATR, neat): $\tilde{\nu}$ / cm-1 = 1708, 1626, 1468, 1437, 1421, 1396, 1362, 1335, 1211, 1187, 1130, 1022, 908, 890, 717.
MS (EI, 70 eV): *m/z* (%) = 245 (14), 231 (22), 189 (11), 188 (99), 161 (10), 160 (100), 155 (13), 140 (24), 133 (10), 130 (20), 83 (25).
HRMS (EI): *m/z* calc. for [$C_{19}H_{24}N_2O_3$]: 328.1781; found 327.1697 (M-H).

N-Methyl-*N*-(2-(4-nitrophenoxy)ethyl)but-3-enamide (8p)

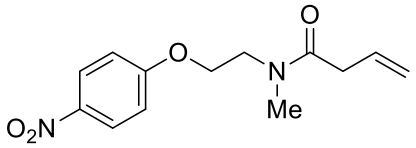

Following modified **TP1B**, *N*-methyl-*N*-(2-(4-nitrophenoxy)ethyl)formamide (**4n**) and $TMP_2Zn{\cdot}2LiCl$ (without $MgCl_2$; prepared by transmetalation of TMPLi with 0.5 equiv of $ZnCl_2$) in THF (1.82 mL, 0.6 mmol, 0.6 equiv) were stirred at 22 °C for 16 h. Allyl bromide (133 mg, 1.10 mmol, 1.1 equiv) and CuCN·2LiCl as 1 M solution in THF (100 µL, 0.100 mmol, 0.10 equiv) were added at 22 °C. After 15 min, the reaction mixture was quenched with saturated $NH_4Cl_{(aq)}$ solution. After workup, the crude product was purified *via* column chromatography (pentane:ethyl acetate = 1:1 to 3:7) to give **8p** (210 mg, 0.790 mmol, 79% yield) as a yellow oil.

^{1}H-NMR (400 MHz, CDCl3): δ / ppm = 8.16 – 8.08 (m, 2H), 6.95 – 6.86 (m, 2H), 6.02 – 5.84 (m, 1H), 5.17 – 5.03 (m, 2H), 4.23 – 4.13 (m, 2H), 3.77 – 3.71 (m, 2H), 3.26 – 2.96 (m, 5H).
^{13}C-NMR (101 MHz, CDCl3): δ / ppm = 171.4, 171.3, 163.6, 163.1, 141.9, 141.6, 131.8, 131.1, 125.9, 125.9, 117.9, 117.7, 114.4, 114.4, 67.0, 66.1, 48.8, 47.7, 38.8, 38.5, 37.6, 33.8.

IR (Diamond-ATR, neat): $\tilde{\nu}$ / cm-1 = 1643, 1608, 1591, 1509, 1497, 1400, 1331, 1298, 1256, 1173, 1138, 1109, 1075, 1044, 1025, 994, 915, 845, 752, 731, 690, 656.
MS (EI, 70 eV): *m/z* (%) = 126 (100).
HRMS (EI): *m/z* calc. for [$C_{13}H_{16}N_2O_4$]: 264.1110; found 265.1181 (M+H).

1-(4-(Pyrimidin-2-yl)piperazin-1-yl)but-3-en-1-one (8q)

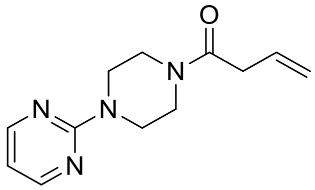

Following **TP1B**, 4-(pyrimidin-2-yl)piperazine-1-carbaldehyde (**4o**) (192 mg, 1.00 mmol, 1.0 equiv) and $TMP_2Zn \cdot 2MgCl_2 \cdot 2LiCl$ 0.33 M in THF (1.82 mL, 0.6 mmol, 0.6 equiv) were stirred at 22 °C for 2 h. Allyl bromide (133 mg, 1.10 mmol, 1.1 equiv) and CuCN·2LiCl as 1 M solution in THF (100 µL, 0.100 mmol, 0.10 equiv) were added at 22 °C. After 15 min, the reaction mixture was quenched with saturated $NH_4Cl_{(aq)}$ solution. After workup, the crude product was purified *via* column chromatography (pentane:ethyl acetate = 1:1 to 8:2) to give **8q** (142 mg, 0.610 mmol, 61% yield) as a white solid.

^{1}H-NMR (400 MHz, CDCl$_3$): δ / ppm = 8.26 (d, *J* = 4.8 Hz, 2H), 6.47 (t, *J* = 4.7 Hz, 1H), 5.91 (ddt, *J* = 16.9, 10.3, 6.5 Hz, 1H), 5.26 – 5.00 (m, 2H), 3.83 – 3.71 (m, 4H), 3.67 – 3.60 (m, 2H), 3.50 – 3.44 (m, 2H), 3.17 – 3.11 (m, 2H).
^{13}C-NMR (101 MHz, CDCl$_3$): δ / ppm = 169.51, 161.5, 157.7, 131.3, 117.9, 110.4, 77.2, 45.5, 43.7, 43.5, 41.4, 38.8.
IR (Diamond-ATR, neat): $\tilde{\nu}$ / cm-1 = 1643, 1582, 1547, 1490, 1432, 1391, 1354, 1306, 1262, 1249, 1224, 1184, 1031, 979, 916, 796.
MS (EI, 70 eV): *m/z* (%) = 232 (36), 191 (18), 163 (22), 162 (12), 136 (14), 135 (11), 14 (68), 122 (63), 121 (43), 120 (38), 109 (10), 108 (100), 96 (11), 80 (27), 79 (16), 56 (16), 41 (16), 39 (36).
HRMS (EI): *m/z* calc. for [$C_{12}H_{16}N_4O$]: 232.1324; found 232.1317.
M.p. (°C): 85.6-87.0.

***N*,*N*-Dibutyl-2-(naphthalen-2-yl)acetamide (9a)**

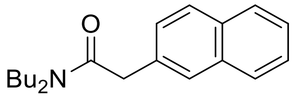

Following **TP2**, *N,N*-dibutylformamide (**4a**) (157 mg, 1.00 mmol, 1.0 equiv), $ZnCl_2$ (500 µL, 0.500 mmol, 0.50 equiv) and Et_3N (56.0 mg, 0.500 mmol, 0.50 equiv) were mixed in THF (2.0 mL) and a freshly prepared solution of TMPLi (1.10 mmol, 1.1 equiv) was dropwise added at 15 °C followed by $MgCl_2 \cdot 2LiCl$ ca. 0.5 M in THF (2.0 mL), 2-(bromomethyl)naphthalene (332 mg, 1.50 mmol, 1.5 equiv) and CuCN·2LiCl as 1 M solution in THF (100 µL, 0.100 mmol, 0.10 equiv). Thereafter, the reaction mixture was quenched with saturated $NH_4Cl_{(aq)}$ solution. After workup, the crude product was purified *via* column chromatography (pentane:ethyl acetate = 95:5) to give **9a** (258 mg, 0.870 mmol, 87% yield) as a colorless oil.

^{1}H-NMR (400 MHz, CDCl$_3$): δ / ppm = 7.84 – 7.76 (m, 3H), 7.69 (s, 1H), 7.49 – 7.38 (m, 3H), 3.86 (s, 2H), 3.38 – 3.32 (m, 2H), 3.26 – 3.19 (m, 2H), 1.58 – 1.42 (m, 4H), 1.37 – 1.22 (m, 4H), 0.90 (dt, *J* = 12.6, 7.3 Hz, 6H).

^{13}C-NMR (101 MHz, CDCl$_3$): δ / ppm = 170.5, 133.7, 133.3, 132.5, 128.4, 127.7, 127.7, 126.2, 125.7, 48.2, 45.9, 41.4, 31.3, 29.9, 20.4, 20.2, 14.0, 13.9 (mixture of rotamers).
IR (Diamond-ATR, neat): $\tilde{\nu}$ / cm-1 = 2957, 2930, 2872, 1636, 1456, 1423, 1374, 1316, 1296, 1268, 1210, 1132, 856, 801, 764, 764, 740.
MS (EI, 70 eV): *m/z* (%) = 297 (28), 182 (11), 156 (68), 142 (13), 142 (12), 141 (100), 139 (23), 115 (58), 100 (26), 57 (58).
HRMS (EI): *m/z* calc. for [$C_{20}H_{27}NO$]: 297.2093; found 297.2083.

2-([1,1'-Biphenyl]-4-yl)-*N*,*N*-dibutylacetamide (9b)

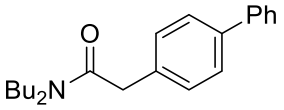

Following **TP2**, *N,N*-dibutylformamide (**4a**) (157 mg, 1.00 mmol, 1.0 equiv), $ZnCl_2$ (500 µL, 0.500 mmol, 0.50 equiv) and Et_3N (56.0 mg, 0.500 mmol, 0.50 equiv) were mixed in THF (2.0 mL) and a freshly prepared solution of TMPLi (1.10 mmol, 1.1 equiv) was dropwise added at 15 °C followed by $MgCl_2$·2LiCl ca. 0.5 M in THF (2.0 mL) 4-(bromomethyl)-1,1'-biphenyl (371 mg, 1.50 mmol, 1.5 equiv) and CuCN·2LiCl as 1 M solution in THF (100 µL, 0.100 mmol, 0.10 equiv). Thereafter, the reaction mixture was quenched with saturated $NH_4Cl_{(aq)}$ solution. After workup, the crude product was purified *via* column chromatography (isohexane:ethyl acetate = 93:7) to give **9b** (194 mg, 0.600 mmol, 60% yield) as a colorless oil.

^{1}H-NMR (400 MHz, CDCl$_3$): δ / ppm = 7.62 – 7.51 (m, 4H), 7.46 – 7.40 (m, 1H), 7.36 – 7.30 (m, 3H), 3.74 (s, 2H), 3.39 – 3.32 (m, 2H), 3.27 – 3.18 (m, 2H), 1.60 – 1.45 (m, 4H), 1.31 (hd, *J* = 7.3, 4.1 Hz, 4H), 0.93 (t, *J* = 7.3 Hz, 6H).
^{13}C-NMR (101 MHz, CDCl$_3$): δ / ppm = 170.4, 141.0, 139.7, 134.8, 129.3 (2C), 128.8 (2C), 127.4 (2C), 127.2, 127.1 (2C), 48.2, 45.9, 40.6, 31.3, 29.9, 20.4, 20.2, 14.0, 13.9.
IR (Diamond-ATR, neat): $\tilde{\nu}$ / cm-1 = 2957, 2930, 2872, 1635, 1487, 1455, 1424, 1375, 1291, 1257, 1195, 1132, 1112, 1076, 1008, 931, 860, 821, 756, 731, 715, 696.
MS (EI, 70 eV): *m/z* (%) = 323 (35), 208 (16), 194 (13), 168 (10), 168 (14), 167 (100), 166 (11), 166 (11), 165 (91), 156 (94), 152 (37), 100 (35), 57 (68).
HRMS (EI): *m/z* calc. for [$C_{22}H_{29}NO$]: 323.2249; found 323.2243.

***N*,*N*-Dibutyl-2-(3-chlorophenyl)acetamide (9c)**

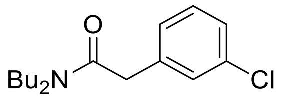

Following **TP2**, *N,N*-dibutylformamide (**4a**) (157 mg, 1.00 mmol, 1.0 equiv), $ZnCl_2$ (500 µL, 0.500 mmol, 0.50 equiv) and Et_3N (56.0 mg, 0.500 mmol, 0.50 equiv) were mixed in THF (2.0 mL) and a freshly prepared solution of TMPLi (1.10 mmol, 1.1 equiv) was dropwise added at 15 °C followed by $MgCl_2$·2LiCl ca. 0.5 M in THF (2.0 mL), 1-(bromomethyl)-3-chlorobenzene (308 mg, 1.50 mmol, 1.5 equiv) and CuCN·2LiCl as 1 M solution in THF (100 µL, 0.100 mmol, 0.10 equiv). Thereafter, the reaction mixture was quenched with saturated $NH_4Cl_{(aq)}$ solution. After workup, the crude product was purified *via* column chromatography (isohexane:ethyl acetate = 9:1) to give **9c** (247 mg, 0.880 mmol, 88% yield) as a colorless oil.

^{1}H-NMR (400 MHz, CDCl$_3$): δ 7.24 – 7.23 (m, 1H), 7.23 – 7.18 (m, 2H), 7.14 – 7.11 (m, 1H), 3.64 (s, 2H), 3.33 – 3.28 (m, 2H), 3.22 – 3.17 (m, 2H), 1.55 – 1.43 (m, 4H), 1.32 – 1.25 (m, 4H), 0.94 – 0.87 (m, 6H).
^{13}C-NMR (101 MHz, CDCl$_3$): δ / ppm = 169.8, 137.6, 134.4, 129.8, 129.1, 127.1, 126.9, 48.2, 45.9, 40.4, 31.3, 29.8, 20.3, 20.2, 13.9, 13.9 (mixture of rotamers).

IR (Diamond-ATR, neat): $\tilde{\nu}$ / cm-1 = 2958, 2931, 2873, 1636, 1597, 1574, 1457, 1432, 1375, 1320, 1291, 1254, 1196, 1136, 1097, 1079, 1000, 939, 868, 771, 684.
MS (EI, 70 eV): *m/z* (%) = 156 (100), 128 (11), 127 (18), 125 (54), 100 (17), 89 (18), 86 (22), 57 (26), 44 (10).
HRMS (EI): *m/z* calc. for [$C_{16}H_{24}ClNO$]: 281.1546; found 281.1540.

***N*,*N*-Dibutyl-2-(3-cyanophenyl)acetamide (9d)**

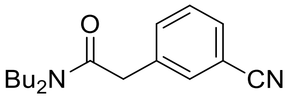

Following **TP2**, *N,N*-dibutylformamide (**4a**) (157 mg, 1.00 mmol, 1.0 equiv), $ZnCl_2$ (500 µL, 0.500 mmol, 0.50 equiv) and Et_3N (56.0 mg, 0.500 mmol, 0.50 equiv) were mixed in THF (2.0 mL) and a freshly prepared solution of TMPLi (1.10 mmol, 1.1 equiv) was dropwise added at 15 °C followed by $MgCl_2$·2LiCl ca. 0.5 M in THF (2.0 mL), 1-(bromomethyl)-3-cyanobenzene (294 mg, 1.50 mmol, 1.5 equiv) and CuCN·2LiCl as 1 M solution in THF (100 µL, 0.100 mmol, 0.10 equiv). Thereafter, the reaction mixture was quenched with saturated $NH_4Cl_{(aq)}$ solution. After workup, the crude product was purified *via* column chromatography (pentane:ethyl acetate = 9:1) to give **9d** (190 mg, 0.700 mmol, 70% yield) as a colorless oil.

^{1}H-NMR (400 MHz, CDCl3): δ / ppm = 7.55 – 7.46 (m, 3H), 7.43 – 7.38 (m, 1H), 3.69 (s, 2H), 3.35 – 3.27 (m, 2H), 3.26 – 3.18 (m, 2H), 1.58 – 1.43 (m, 4H), 1.38 – 1.20 (m, 4H), 0.99 – 0.82 (m, 6H).
^{13}C-NMR (101 MHz, $CDCl_3$): δ / ppm = 169.2, 137.2, 133.9, 132.7, 130.6, 129.3, 118.8, 112.6, 48.2, 46.1, 39.8, 31.4, 29.8, 20.3, 20.2, 13.9 (mixture of rotamers).
IR (Diamond-ATR, neat): $\tilde{\nu}$ / cm-1 = 2931, 2958, 2229, 1635, 1582, 1481, 1456, 1425, 1375, 1322, 1293, 1256, 1213, 1137, 1114, 1100, 930, 894, 781, 731, 688.
MS (EI, 70 eV): *m/z* (%) = 156 (87), 117 (13), 116 (100), 114 (10), 100 (26), 89 (27), 86 (69), 57 (18), 44 (28).
HRMS (EI): *m/z* calc. for [$C_{17}H_{24}N_2O$]: 272.1889; found 272.1882.

***N*,*N*-Dibutyl-2-(4-chlorophenyl)acetamide (9e)**

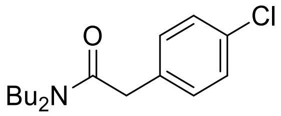

Following **TP2**, *N,N*-dibutylformamide (**4a**) (157 mg, 1.00 mmol, 1.0 equiv), $ZnCl_2$ (500 µL, 0.500 mmol, 0.50 equiv) and Et_3N (56.0 mg, 0.500 mmol, 0.50 equiv) were mixed in THF (2.0 mL) and a freshly prepared solution of TMPLi (1.10 mmol, 1.1 equiv) was dropwise added at 15 °C followed by $MgCl_2$·2LiCl ca. 0.5 M in THF (2.0 mL), 1-(bromomethyl)-4-chlorobenzene (308 mg, 1.50 mmol, 1.5 equiv) and CuCN·2LiCl as 1 M solution in THF (100 µL, 0.100 mmol, 0.10 equiv). Thereafter, the reaction mixture was quenched with saturated $NH_4Cl_{(aq)}$ solution. After workup, the crude product was purified *via* column chromatography (pentane:ethyl acetate = 93:7) to give **9e** (223 mg, 0.790 mmol, 79% yield) as a colorless oil.

^{1}H-NMR (400 MHz, $CDCl_3$): δ / ppm = 7.32 – 7.25 (m, 2H), 7.23 – 7.14 (m, 2H), 3.64 (s, 2H), 3.34 – 3.28 (m, 2H), 3.23 – 3.17 (m, 2H), 1.53 – 1.45 (m, 4H), 1.34 – 1.23 (m, 4H), 0.96 – 0.87 (m, 7H).
^{13}C-NMR (101 MHz, $CDCl_3$): δ / ppm = 170.1, 134.2, 132.6, 130.3, 128.8, 48.2, 45.9, 40.2, 31.3, 29.9, 20.4, 20.2, 14.0, 13.9 (mixture of rotamers).

IR (Diamond-ATR, neat): $\tilde{\nu}$ / cm-1 = 2958, 2931, 2873, 1635, 1492, 1456, 1425, 1375, 1319, 1296, 1255, 1198, 1131, 1113, 1090, 1016, 930, 858, 806, 735.
MS (EI, 70 eV): *m/z* (%) = 281 (18), 156 (100), 127 (28), 125 (85), 100 (29), 89 (21), 86 (24), 57 (42), 44 (12).
HRMS (EI): *m/z* calc. for [$C_{16}H_{24}ClNO$]: 281.1546; found 281.1539.

***N*,*N*-Dibutyl-2-(4-(trifluoromethyl)phenyl)acetamide (9f)**

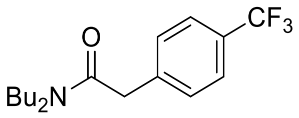

Following **TP2**, *N,N*-dibutylformamide (**4a**) (157 mg, 1.00 mmol, 1.0 equiv), $ZnCl_2$ (500 µL, 0.500 mmol, 0.50 equiv) and Et_3N (56.0 mg, 0.500 mmol, 0.50 equiv) were mixed in THF (2.0 mL) and a freshly prepared solution of TMPLi (1.10 mmol, 1.1 equiv) was dropwise added at 15 °C followed by $MgCl_2$·2LiCl ca. 0.5 M in THF (2.0 mL), 1-(bromomethyl)-4-(trifluoromethyl)benzene (359 mg, 1.50 mmol, 1.5 equiv) and CuCN·2LiCl as 1 M solution in THF (100 µL, 0.100 mmol, 0.10 equiv). Thereafter, the reaction mixture was quenched with saturated $NH_4Cl_{(aq)}$ solution. After workup, the crude product was purified *via* column chromatography (pentane:ethyl acetate = 96:4) to give **9f** (229 mg, 0.640 mmol, 64% yield) as a colorless oil.

^{1}H-NMR (400 MHz, $CDCl_3$): 7.56 (d, *J* = 8.0 Hz, 2H), 7.36 (d, *J* = 8.0 Hz, 2H), 3.73 (s, 2H), 3.36 – 3.28 (m, 2H), 3.26 – 3.18 (m, 2H), 1.55 – 1.46 (m, 4H), 1.36 – 1.23 (m, 4H), 0.95 – 0.86 (m, 6H).
^{13}C-NMR (101 MHz, CDCl3): δ / ppm = 169.6, 139.8, 129.6, 129.4, 129.3, 129.0, 128.7, 128.4, 125.7, 125.6, 125.6, 125.5, 125.5, 123.0, 120.3, 48.3, 46.0, 40.5, 31.3, 29.9, 20.4, 20.2, 14.0, 13.9 (mixture of rotamers).
^{19}F-NMR (377 MHz, CDCl3): δ / ppm = –62.5.
IR (Diamond-ATR, neat): $\tilde{\nu}$ / cm-1 = 2933, 2960, 2875, 1638, 1457, 1428, 1377, 1323, 1162, 1120, 1066, 1020, 824.
MS (EI, 70 eV): *m/z* (%) = 159 (82), 157 (10), 156 (100), 109 (27), 100 (18), 86 (22), 57 (23), 44 (13).
HRMS (EI): *m/z* calc. for [$C_{17}H_{24}F_3NO$]: 315.1810; found 315.1803.

2-(2-Bromophenyl)-*N*,*N*-dibutylacetamide (9g)

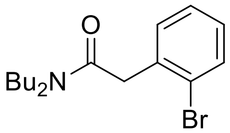

Following **TP2**, *N,N*-dibutylformamide (**4a**) (157 mg, 1.00 mmol, 1.0 equiv), $ZnCl_2$ (500 µL, 0.500 mmol, 0.50 equiv) and Et_3N (56.0 mg, 0.500 mmol, 0.50 equiv) were mixed in THF (2.0 mL) and a freshly prepared solution of TMPLi (1.10 mmol, 1.1 equiv) was dropwise added at 15 °C followed by $MgCl_2$·2LiCl ca. 0.5 M in THF (2.0 mL), 1-(bromomethyl)-4-(trifluoromethyl)benzene (374 mg, 1.50 mmol, 1.5 equiv) and CuCN·2LiCl as 1 M solution in THF (100 µL, 0.100 mmol, 0.10 equiv). Thereafter, the reaction mixture was quenched with saturated $NH_4Cl_{(aq)}$ solution. After workup, the crude product was purified *via* column chromatography (pentane:ethyl acetate = 95:5) to give **9g** (245 mg, 0.750 mmol, 75% yield) as a colorless oil.

^{1}H-NMR (400 MHz, $CDCl_3$): δ / ppm = 7.54 (dd, *J* = 8.0, 1.2 Hz, 1H), 7.32 – 7.22 (m, 2H), 7.10 (td, *J* = 7.6, 1.9 Hz, 1H), 3.79 (s, 2H), 3.37 – 3.30 (m, 2H), 3.26 – 3.18 (m, 2H), 1.60 – 1.48 (m, 4H), 1.38 – 1.24 (m, 4H), 0.92 (q, *J* = 7.1 Hz, 6H).
^{13}C-NMR (101 MHz, $CDCl_3$): δ / ppm = 169.6, 135.8, 132.7, 131.0, 128.5, 127.6, 124.8, 48.3, 46.1, 41.0, 31.3, 29.8, 20.4, 20.2, 14.0, 14.0 (mixture of rotamers).
IR (Diamond-ATR, neat): $\tilde{\nu}$ / cm-1 = 2957, 2930, 2872, 1640, 1466, 1456, 1440, 1425, 1375, 1322, 1290, 1259, 1216, 1199, 1138, 1113, 1048, 1026, 929, 745, 663.
MS (EI, 70 eV): *m/z* (%) = 247 (17), 246 (100), 190 (17), 171 (17), 169 (17), 134 (11).
HRMS (EI): *m/z* calc. for [$C_{16}H_{24}BrNO$]: 325.1036; found 326.1110 (M+H).

1-Morpholino-2-(naphthalen-2-yl)ethanone (9h)

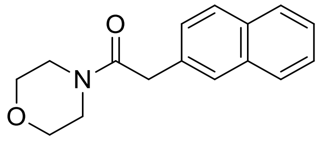

Following **TP2**, morpholine-4-carbaldehyde (**4c**) (115 mg, 1.00 mmol, 1.0 equiv), $ZnCl_2$ (500 µL, 0.500 mmol, 0.50 equiv) and Et_3N (56.0 mg, 0.500 mmol, 0.50 equiv) were mixed in THF (2.0 mL) and a freshly prepared solution of TMPLi (1.10 mmol, 1.1 equiv) was dropwise added at 15 °C followed by $MgCl_2$·2LiCl ca. 0.5 M in THF (2.0 mL), 1-(bromomethyl)-4-(trifluoromethyl)benzene (374 mg, 1.50 mmol, 1.5 equiv) and CuCN·2LiCl as 1 M solution in THF (100 µL, 0.100 mmol, 0.10 equiv). Thereafter, the reaction mixture was quenched with saturated $NH_4Cl_{(aq)}$ solution. After workup, the crude product was purified *via* column chromatography (isohexane:ethyl acetate = 9:1) to give **9l** (145 mg, 0.570 mmol, 57% yield) as a colorless oil.

^{1}H-NMR (400 MHz, $CDCl_3$): δ / ppm = 7.85 – 7.81 (m, 2H), 7.80 – 7.77 (m, 1H), 7.69 – 7.67 (m, 1H), 7.51 – 7.44 (m, 2H), 7.39 (dd, *J* = 8.5, 1.8 Hz, 1H), 3.89 (s, 2H), 3.71 – 3.62 (m, 4H), 3.46 (s, 4H).
^{13}C-NMR (101 MHz, $CDCl_3$): δ / ppm = 169.7, 133.6, 132.5, 132.4, 128.7, 127.8, 127.6, 127.1, 126.8, 126.4, 125.9, 66.9, 66.6, 46.6, 42.3, 41.2.
IR (Diamond-ATR, neat): $\tilde{\nu}$ / cm-1 = 1631, 1600, 1508, 1429, 1361, 1300, 1272, 1257, 1227, 1112, 1068, 1036, 966, 908, 859, 818, 795, 765, 725, 673.
MS (EI, 70 eV): *m/z* (%) = 256 (13), 255 (77), 212 (15), 168 (24), 141 (89), 140 (11), 139 (27), 115 (49), 114 (100), 70 (52).
HRMS (EI): *m/z* calc. for [$C_{16}H_{17}NO_2$]: 255.1259; found 255.1252.

2-(Naphthalen-2-yl)-1-(4-(3-(trifluoromethyl)pyridin-2-yl)piperazin-1-yl)ethanone (9i)

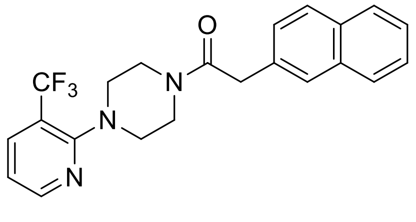

Following **TP2**, 4-(3-(trifluoromethyl)pyridin-2-yl)piperazine-1-carbaldehyde (**4f**) (115 mg, 1.00 mmol, 1.0 equiv), $ZnCl_2$ (500 µL, 0.500 mmol, 0.50 equiv) and Et_3N (56.0 mg, 0.500 mmol, 0.50 equiv) were mixed in THF (2.0 mL) and a freshly prepared solution of TMPLi (1.10 mmol, 1.1 equiv) was dropwise added at 15 °C followed by $MgCl_2$·2LiCl ca. 0.5 M in THF (2.0 mL), 2-(bromomethyl)naphthalene (332 mg, 1.50 mmol, 1.5 equiv) and CuCN·2LiCl as 1 M solution in THF (100 µL, 0.100 mmol, 0.10 equiv). Thereafter, the reaction mixture was quenched with saturated $NH_4Cl_{(aq)}$ solution. After workup, the crude product was purified *via* column

chromatography (isohexane:ethyl acetate = 9.0:1.0) to give **9i** (235 mg, 0.590 mmol, 59% yield) as a yellow amorphous solid.

^{1}H-NMR (400 MHz, CDCl3): δ / ppm = 8.46 – 8.34 (m, 1H), 7.89 – 7.77 (m, 4H), 7.74 – 7.69 (m, 1H), 7.52 – 7.39 (m, 3H), 7.03 – 6.97 (m, 1H), 3.94 (s, 2H), 3.87 – 3.78 (m, 2H), 3.67 – 3.58 (m, 2H), 3.28 – 3.21 (m, 2H), 3.15 – 3.06 (m, 2H).
^{13}C-NMR (101 MHz, CDCl3): δ / ppm = 169.7, 159.6, 151.2, 137.3 (q, *J* = 5.0 Hz), 133.7, 132.5 (d, *J* = 12.3 Hz), 128.6 , 127.7 (d, *J* = 6.0 Hz), 127.1, 126.9, 126.3, 125.8, 123.9 (q, *J* = 272.6 Hz), 117.9, 117.6 (q, *J* = 31.5 Hz), 50.8 (d, *J* = 6.3 Hz), 46.2, 41.9, 41.4.
19F-NMR (377 MHz, **CDCl3):** δ / ppm = □60.2.
IR (Diamond-ATR, neat): $\tilde{\nu}$ / cm-1 = 1640, 1591, 1568, 1438, 1378, 1308, 1282, 1232, 1146, 1105, 1082, 1025, 966, 906, 858, 794, 764, 725, 679.
MS (EI, 70 eV): *m/z* (%) = 399 (17), 258 (29), 230 (11), 216 (10), 214(18), 212 (20), 211 (29), 201 (37), 189 (47), 188 (61), 187 (41), 175 (65), 169 (15), 168 (52), 163 (11), 147 (16), 146 (12), 142 (21), 141 (100), 139 (12), 128 (19), 115 (30), 70 (14), 69 (12), 56 (43), 43 (21).
HRMS (EI): *m/z* calc. for [$C_{22}H_{20}F_3N_3O$]: 399.1558; found 399.1550.

N,*N*-Dibutyl-2-hydroxy-2-phenylacetamide (10a)

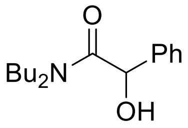

Following **TP3**, *N,N*-dibutylformamide (**4a**) (157 mg, 1.00 mmol, 1.0 equiv), $ZnCl_2$ (500 µL, 0.500 mmol, 0.50 equiv) and Et_3N (56.0 mg, 0.500 mmol, 0.50 equiv) were mixed in THF (2.0 mL) and a freshly prepared solution of TMPLi (1.10 mmol, 1.1 equiv) was dropwise added at 15 °C followed by $MgCl_2$·2LiCl ca. 0.5 M in THF (2.0 mL) and neat benzaldehyde (127 mg, 1.20 mmol, 1.2 equiv). Thereafter, the reaction mixture was quenched with saturated $NH_4Cl_{(aq)}$ solution. After workup, the crude product was purified *via* column chromatography (isohexane:ethyl acetate = 9:1) to give **10a** (195 mg, 0.740 mmol, 74% yield) as a colorless oil.

^{1}H-NMR (400 MHz, $CDCl_3$): δ / ppm = 7.39 – 7.27 (m, 6H), 5.14 (s, 1H), 4.69 (s, 1H), 3.50 (ddd, *J* = 13.4, 9.3, 6.0 Hz, 1H), 3.21 (ddd, *J* = 13.4, 9.3, 6.0 Hz, 1H), 3.08 (ddd, *J* = 14.5, 10.4, 5.7 Hz, 1H), 2.90 (ddd, *J* = 14.5, 10.6, 4.9 Hz, 1H), 1.60 – 1.44 (m, 2H), 1.43 – 1.22 (m, 3H), 1.18 – 1.04 (m, 2H), 1.03 – 0.88 (m, 4H), 0.80 (t, *J* = 7.3 Hz, 3H).
^{13}C-NMR (101 MHz, $CDCl_3$): δ / ppm = 171.9, 140.0, 129.1 (2C), 128.6, 127.6 (2C), 71.8, 46.7, 46.2, 30.2, 29.5, 20.3, 20.1, 13.9, 13.8.
IR (Diamond-ATR, neat): $\tilde{\nu}$ / cm-1 = 3385, 2958, 2932, 2873, 1636, 1466, 1456, 1397, 1374, 1294, 1255, 1232, 1189, 1080, 851, 766, 734, 714, 699, 668.
MS (EI, 70 eV): *m/z* (%) = 156 (92), 114 (100), 107 (12), 100 (42), 79 (24), 77 (15), 57 (55).
HRMS (EI): *m/z* calc. for [$C_{16}H_{25}NO_2$]: 263.1885; found 264.1958 (M+H).

2-(2-Bromophenyl)-2-hydroxy-1-(*trans*-octahydroquinolin-1(2H)-yl)ethanone (10b)

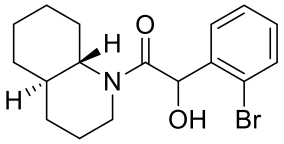

Following **TP3**, *trans*-octahydroquinoline-2(1H)-carbaldehyde (**4e**) (167 mg, 1.00 mmol, 1.0 equiv), $ZnCl_2$ (500 µL, 0.500 mmol, 0.50 equiv) and Et_3N (56.0 mg, 0.500 mmol, 0.50 equiv) were mixed in THF (2.0 mL) and a freshly prepared solution of TMPLi (1.10 mmol, 1.1 equiv)

was dropwise added at 15 °C followed by $MgCl_2 \cdot 2LiCl$ ca. 0.5 M in THF (2.0 mL) and neat 2-bromobenzaldehyde (222 mg, 1.20 mmol, 1.2 equiv). Thereafter, the reaction mixture was quenched with saturated $NH_4Cl_{(aq)}$ solution. After workup, the crude product was purified *via* column chromatography (isohexane:ethyl acetate = 9:1) to give **10b** (200 mg, 0.570 mmol, 57% yield) as a colorless oil.

^{1}H-NMR (400 MHz, $CDCl_3$): δ / ppm = 7.62 – 7.56 (m, 1H), 7.31 – 7.26 (m, 1H), 7.23 – 7.13 (m, 2H), 5.54 (s, 1H), 4.85 (s, 1H), 3.61 – 3.27 (m, 1H), 3.23 – 2.96 (m, 1H), 2.94 – 2.77/2.42 – 2.03 (m, 1H), 1.85 – 1.74 (m, 2H), 1.73 – 1.60 (m, 2H), 1.59 – 1.35 (m, 4H), 1.34 – 0.93 (m, 5H).
^{13}C-NMR (101 MHz, $CDCl_3$): δ / ppm = 171.3, 139.4, 139.3, 133.3, 133.3, 130.1, 130.1, 128.9, 128.5, 128.4, 124.5, 124.2, 70.7, 70.6, 62.87, 62.0, 38.9, 38.5, 38.1, 37.9, 32.9, 32.9, 30.1, 29.8, 26.3, 26.2, 25.9, 25.8, 25.5, 25.4, 22.7, 21.6.
IR (Diamond-ATR, neat): $\tilde{\nu}$ / cm-1 = 3376, 2925, 2886, 2857, 1634, 1592, 1568, 1460, 1440, 1391, 1359, 1220, 1287, 1273, 1248, 1214, 1188, 1172, 1138, 1120, 1072, 1046, 1020, 993, 972, 936, 917, 871, 857, 842, 832, 797, 759, 729.
MS (EI, 70 eV): *m/z* (%) = 183 (10), 167 (11), 166 (100), 138 (13), 86 (14), 81 (16).
HRMS (EI): *m/z* calc. for [$C_{17}H_{22}BrNO_2$]: 351.0834; found 350.0748 (M-H).

_N,N_-Dibutyl-2-cyclopropyl-2-oxoacetamide (11a)

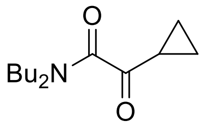

Following **TP4**, *N,N*-dibutylformamide (**4a**) (157 mg, 1.00 mmol, 1.0 equiv), $ZnCl_2$ (500 µL, 0.500 mmol, 0.50 equiv) and Et_3N (56.0 mg, 0.500 mmol, 0.50 equiv) were mixed in THF (2.0 mL) and a freshly prepared solution of TMPLi (1.10 mmol, 1.1 equiv) was dropwise added at 15 °C followed by neat cyclopropanecarbonyl chloride (125 mg, 1.20 mmol, 1.2 equiv). Thereafter, the reaction mixture was quenched with saturated $NH_4Cl_{(aq)}$ solution. After workup, the crude product was purified *via* column chromatography (pentane:ethyl acetate = 98:2) to give **11a** (122 mg, 0.540 mmol, 54% yield) as a yellow oil.

^{1}H-NMR (400 MHz, $CDCl_3$): δ / ppm = 3.38 – 3.32 (m, 2H), 3.24 – 3.15 (m, 2H), 2.31 – 2.24 (m, 1H), 1.61 – 1.48 (m, 4H), 1.37 – 1.29 (m, 2H), 1.28 – 1.21 (m, 2H), 1.21 – 1.17 (m, 2H), 1.10 – 1.05 (m, 2H), 0.92 (t, *J* = 7.4 Hz, 3H), 0.89 (t, *J* = 7.4 Hz, 3H).
^{13}C-NMR (101 MHz, $CDCl_3$): δ / ppm = 201.3, 167.6, 47.5, 44.6, 31.2, 29.5, 20.3, 20.0, 19.8, 13.9, 13.8, 12.8 (mixture of rotamers).
IR (Diamond-ATR, neat): $\tilde{\nu}$ / cm-1 = 2959, 2933, 2874, 1693, 1633, 1458, 1436, 1374, 1191, 1100, 1068, 953, 880, 677.
MS (EI, 70 eV): *m/z* (%) = 156 (91), 128 (28), 57 (100), 41 (17).
HRMS (EI): *m/z* calc. for [$C_{13}H_{23}NO_2$]: 225.1729; found 226.1801 (M+H).

1-(4-Bromophenyl)-2-(_trans_-octahydroquinolin-1(2H)-yl)ethane-1,2-dione (11b)

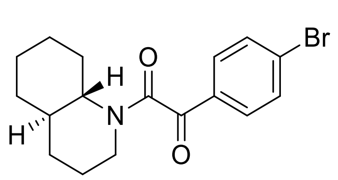

Following **TP4**, *trans*-octahydroquinoline-2(1H)-carbaldehyde (**4e**) (167 mg, 1.00 mmol, 1.0 equiv), $ZnCl_2$ (500 µL, 0.500 mmol, 0.50 equiv) and Et_3N (56.0 mg, 0.500 mmol, 0.50 equiv) were mixed in THF (2.0 mL) and a freshly prepared solution of TMPLi (1.10 mmol, 1.1 equiv)

was dropwise added at 15 °C followed by neat 4-bromobenzoyl chloride (263 mg, 1.20 mmol, 1.2 equiv). Thereafter, the reaction mixture was quenched with NH_4Cl. After workup, the crude product was purified *via* column chromatography (pentane:ethyl acetate = 98:2) to give **11b** (210 mg, 0.600 mmol, 60% yield) as a yellow oil.

^{1}H-NMR (400 MHz, $CDCl_3$): δ / ppm = 7.82 – 7.72 (m, 2H), 7.65 – 7.57 (m, 2H), 4.03 – 3.24 (m, 2H), 3.17 – 2.95 (m, 1H), 2.50 – 2.31 (m, 1H), 1.89 – 1.77 (m, 1H), 1.76 – 1.51 (m, 7H), 1.50 – 1.25 (m, 2H), 1.25 – 0.92 (m, 2H).
^{13}C-NMR (101 MHz, $CDCl_3$): δ / ppm = 190.4, 189.5, 166.3, 166.2, 132.2, 131.1, 130.0, 63.3, 62.0, 41.9, 40.7, 40.4, 38.2, 33.0, 32.4, 30.2, 29.6, 26.9, 26.0, 25.9, 25.5, 25.5, 24.0, 23.6 (mixture of rotamers).
IR (Diamond-ATR, neat): $\tilde{\nu}$ / cm-1 = 2926, 2856, 1681, 1633, 1585, 1485, 1447, 1398, 1361, 1284, 1257, 1214, 1187, 1168, 1069, 1009, 988, 948, 888, 849, 832, 798, 761, 726, 688, 661.
MS (EI, 70 eV): *m/z* (%) = 351 (14), 349 (14), 309 (15), 308 (95), 307 (16), 306 (100), 281 (11), 280 (25), 278 (26), 225 (10), 207 (26), 198 (21), 196 (21), 185 (11), 183 (11), 138 (28), 137 (92), 136 (22), 122 (20), 94 (12), 89 (21).
HRMS (EI): *m/z* calc. for [$C_{17}H_{20}BrNO_2$]: 349.0677; found 349.0670.

1-(4-Chlorophenyl)-2-(*trans*-octahydroquinolin-1(2H)-yl)ethane-1,2-dione (11c)

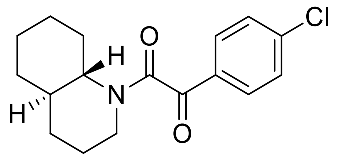

Following **TP4**, *trans*-octahydroquinoline-2(1H)-carbaldehyde (**4e**) (167 mg, 1.00 mmol, 1.0 equiv), $ZnCl_2$ (500 µL, 0.500 mmol, 0.50 equiv) and Et_3N (56.0 mg, 0.500 mmol, 0.50 equiv) were mixed in THF (2.0 mL) and a freshly prepared solution of TMPLi (1.10 mmol, 1.1 equiv) was dropwise added at 15 °C followed by neat 4-chlorobenzoyl chloride (210 mg, 1.20 mmol, 1.2 equiv). Thereafter, the reaction mixture was quenched with saturated $NH_4Cl_{(aq)}$ solution. After workup, the crude product was purified *via* column chromatography (pentane:ethyl acetate = 97:3) to give **11c** (211 mg, 0.690 mmol, 69% yield) as a yellow oil.

^{1}H-NMR (400 MHz, $CDCl_3$): δ / ppm = 7.91 – 7.79 (m, 2H), 7.47 – 7.37 (m, 2H), 4.05 – 3.22 (m, 2H), 3.15 – 2.96 (m, 1H), 2.53 – 2.29 (m, 1H), 1.94 – 1.77 (m, 1H), 1.76 – 1.50 (m, 7H), 1.47 – 1.23 (m, 2H), 1.23 – 0.90 (m, 2H).
^{13}C-NMR (101 MHz, $CDCl_3$): δ / ppm = 190.1, 189.3, 166.3, 166.3, 141.1, 140.7, 131.7, 131.0, 129.4, 63.3, 62.0, 41.9, 40.6, 40.4, 38.2, 33.0, 32.4, 30.2, 29.6, 26.9, 25.9, 25.8, 25.5, 25.4, 23.9, 23.5 (mixture of rotamers).
IR (Diamond-ATR, neat): $\tilde{\nu}$ / cm-1 = 2926, 2856, 1682, 1634, 1586, 1571, 1489, 1446, 1400, 1364, 1287, 1257, 1214, 1187, 1167, 1138, 1087, 1012, 948, 888, 850, 832, 798, 764, 753, 690, 667.
MS (EI, 70 eV): *m/z* (%) = 167 (10), 166 (100), 164 (30), 139 (28), 136 (21), 81 (11).
HRMS (EI): *m/z* calc. for [$C_{17}H_{20}ClNO_2$]: 305.1183; found 305.1175.

1-(2-Iodophenyl)-2-(*trans*-octahydroquinolin-1(2H)-yl)ethane-1,2-dione (11d)

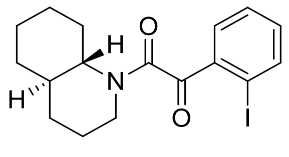

Following **TP4**, *trans*-octahydroquinoline-2(1H)-carbaldehyde (**4e**) (167 mg, 1.00 mmol, 1.0 equiv), $ZnCl_2$ (500 µL, 0.500 mmol, 0.50 equiv) and Et_3N (56.0 mg, 0.500 mmol, 0.50 equiv) were mixed in THF (2.0 mL) and a freshly prepared solution of TMPLi (1.10 mmol, 1.1 equiv) was dropwise added at 15 °C followed by neat 2-iodobenzoyl chloride (319 mg, 1.20 mmol, 1.2 equiv). Thereafter, the reaction mixture was quenched with saturated $NH_4Cl_{(aq)}$ solution. After workup, the crude product was purified *via* column chromatography (pentane:ethyl acetate = 98:2) to give **11d** (333 mg, 0.840 mmol, 84% yield) as a yellow oil.

^{1}H-NMR (400 MHz, $CDCl_3$): δ / ppm = 7.98 (dd, *J* = 8.0, 1.2 Hz, 1H), 7.72 (dd, *J* = 7.7, 1.7 Hz, 1H), 7.43 (td, *J* = 7.6, 1.1 Hz, 1H), 7.18 (td, 1H), 3.43 – 3.38 (m, 1H), 3.38 – 3.34 (m, 1H), 3.33 – 3.27 (m, 1H), , 2.44 – 2.38 (m, 1H), 1.90 – 1.76 (m, 2H), 1.76 – 1.57 (m, 6H), 1.56 – 1.45 (m, 1H), 1.45 – 1.34 (m, 1H), 1.34 – 1.24 (m, 1H), 1.24 – 0.90 (m, 3H) (major rotamer).
8.05 (d, *J* = 7.9 Hz, 1H), 7.79 (d, *J* = 7.8 Hz, 1H), 7.43 (td, *J* = 7.6, 1.1 Hz, 1H), 7.18 (td, 1H), 3.93 – 3.84 (m, 1H), 3.59 – 3.48 (m, 1H), 3.12 – 3.05 (m, 1H), 1.90 – 1.76 (m, 2H), 1.76 – 1.57 (m, 6H), 1.56 – 1.45 (m, 1H), 1.45 – 1.34 (m, 1H), 1.34 – 1.24 (m, 1H), 1.24 – 0.90 (m, 3H) (minor rotamer).
^{13}C-NMR (101 MHz, $CDCl_3$): δ / ppm = 191.3, 189.9, 165.5, 165.3, 142.8, 142.0, 136.7, 135.3, 133.6, 133.1, 128.3, 128.2, 94.0, 93.2, 63.3, 62.2, 41.7, 40.3, 40.1, 38.2, 33.0, 32.7, 29.7, 29.3, 26.8, 26.0, 25.6, 25.5, 23.6, 23.2 (mixture of rotamers).
IR (Diamond-ATR, neat): $\tilde{\nu}$ / cm-1 = 2925, 2855, 1685, 1629, 1578, 1560, 1445, 1428, 1364, 1278, 1266, 1253, 1210, 1185, 1170, 1126, 1053, 1016, 1008, 986, 948, 919, 907, 896, 886, 798, 732, 690.
MS (EI, 70 eV): *m/z* (%) = 231 (14), 167 (11), 166 (100), 81 (22), 76 (12), 67 (12), 55 (11), 41 (14).
HRMS (EI): *m/z* calc. for [$C_{17}H_{20}INO_2$]: 397.0533; found 397.0528.
M.p. (°C): 102.4-104.7.
X-Ray: Crystals suitable for X-Ray diffraction were obtained by recrystallization from Et_2O (complete dissolution on reflux). See pages 145-148.

***N*,*N*-Dibutyl-1-(diphenylphosphoryl)formamide (11e)**

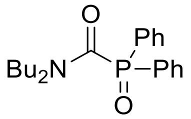

Following **TP4**, *N,N*-dibutylformamide (**4a**) (157 mg, 1.00 mmol, 1.0 equiv), $ZnCl_2$ (500 µL, 0.500 mmol, 0.50 equiv) and Et_3N (56.0 mg, 0.500 mmol, 0.50 equiv) were mixed in THF (2.0 mL) and a freshly prepared solution of TMPLi (1.10 mmol, 1.1 equiv) was dropwise added at 15 °C followed by neat diphenylphosphinic chloride (284 mg, 1.20 mmol, 1.2 equiv). Thereafter, the reaction mixture was quenched with NH_4Cl. After workup, the crude product was purified *via* column chromatography (pentane:ethyl acetate = 85:15) to give **11e** (250 mg, 0.700 mmol, 70% yield) as a colorless oil.

^{1}H-NMR (400 MHz, CDCl3): δ / ppm = 7.94 – 7.83 (m, 4H), 7.57 – 7.50 (m, 2H), 7.50 – 7.41 (m, 4H), 4.00 – 3.91 (m, 2H), 3.41 – 3.32 (m, 2H), 1.60 – 1.52 (m, 2H), 1.51 – 1.42 (m, 2H), 1.37 – 1.18 (m, 4H), 0.90 (t, *J* = 7.3 Hz, 3H), 0.85 (t, *J* = 7.3 Hz, 3H).
^{13}C-NMR (101 MHz, CDCl3): δ / ppm = 169.4, 168.2, 132.2, 132.1, 132.0, 131.9, 130.8, 128.6, 128.5, 46.8, 46.2 (d, *J* = 3.1 Hz), 31.7, 29.4, 20.4, 19.9, 13.9, 13.9 (mixture of rotamers).
^{31}P-NMR (162 MHz, $CDCl_3$): δ / ppm = 20.2.
IR (Diamond-ATR, neat): $\tilde{\nu}$ / cm-1 = 2958, 2932, 1605, 1465, 1457, 1437, 1375, 1224, 1190, 1110, 1094, 1070, 760, 727, 714, 697.
MS (EI, 70 eV): *m/z* (%) = 202 (35), 201 (100), 183 (11), 156 (26), 128 (56), 77 (32), 57 (78), 47 (10), 41 (11), 39 (34).
HRMS (EI): *m/z* calc. for [$C_{21}H_{28}NO_2P$]: calc. 357.1858; found 357.1855.
M.p. (°C): 95.9-98.3 °C.

_N,N_-Dibutyl-3-oxocyclohexanecarboxamide (12a)

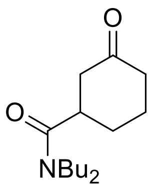

Following **TP5**, _N,N_-dibutylformamide (**4a**) (314 mg, 2.00 mmol, 2.0 equiv), $ZnCl_2$ (1.00 mL, 1.00 mmol, 1.0 equiv) and Et_3N (101 mg, 1.00 mmol, 1.0 equiv) were mixed in THF (4.0 mL) and a freshly prepared solution of TMPLi (2.20 mmol, 2.2 equiv) was dropwise added at 15 °C. After cooling to −78 °C CuCN·2LiCl (1.00 mL, 1.00 mmol, 1.0 equiv) and BF_3·Et_2O (123 µL, 1.0 mmol, 1.0 equiv) and cyclox-2-en-1-one (96.0 mg, 1.00 mmol, 1.0 equiv). Thereafter, the reaction mixture was quenched with saturated $NH_4Cl_{(aq)}$ solution. After workup, the crude product was purified _via_ column chromatography (pentane:ethyl acetate = 9:1 to 8:2) to give **12a** (137 mg, 0.540 mmol, 54% yield) as a colorless oil.

^{1}H-NMR (400 MHz, $CDCl_3$): 3.40 (dt, _J_ = 13.3, 7.6 Hz, 1H), 3.29 – 3.11 (m, 3H), 2.99 – 2.88 (m, 1H), 2.75 – 2.66 (m, 1H), 2.44 – 2.21 (m, 3H), 2.18 – 2.03 (m, 1H), 1.97 – 1.83 (m, 2H), 1.79 – 1.60 (m, 2H), 1.56 – 1.41 (m, 4H), 1.36 – 1.18 (m, 4H), 0.92 (dt, _J_ = 10.7, 7.3 Hz, 6H).
^{13}C-NMR (101 MHz, $CDCl_3$): δ / ppm = 210.9, 173.0, 47.7, 46.1, 44.3, 41.1, 40.7, 31.9, 30.0, 28.4, 25.0, 20.3, 20.2, 14.0, 13.9 (mixture of rotamers).
IR (Diamond-ATR, neat): $\tilde{\nu}$ / cm-1 = 2956, 2932, 2872, 1712, 1633, 1456, 1428, 1376, 1315, 1287, 1260, 1220, 1200, 1141, 1097, 732.
MS (EI, 70 eV): _m/z_ (%) = 211 (12), 210 (100), 184 (38), 168 (20), 156 (30), 154 (22), 138 (12), 130 (11), 128 (12), 128 (10), 125 (20), 114 (16), 97 (15), 86 (98), 69 (22), 57 (14), 44 (31).
HRMS (EI): _m/z_ calc. for [$C_{15}H_{27}NO_2$]: 253.2042; found 254.2114 (M+H).

_N,N_-Dibutyl-3-(trimethylsilyl)benzamide (13a)

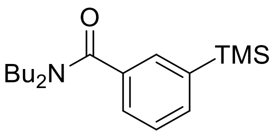

Following **TP6**, _N,N_-dibutylformamide (**4a**) (157 mg, 1.00 mmol, 1.0 equiv), $ZnCl_2$ (500 µL, 0.500 mmol, 1.0 equiv) and Et_3N (56.0 mg, 0.500 mmol, 1.0 equiv) were mixed in THF (2.0 mL) and a freshly prepared solution of TMPLi (1.10 mmol, 1.1 equiv) was dropwise added at 15 °C followed by Pd(dppf)Cl_2 (36.6 mg, 0.050 µmol, 0.05 equiv), 3-bromophenyl)trimethylsilane (183 mg, 0.80 mmol, 0.8 equiv) and CuCN·2LiCl 1M in THF (20.0 µL, 0.020 mmol, 0.02 equiv). Thereafter, the reaction mixture was quenched with saturated $NH_4Cl_{(aq)}$ solution. After workup, the crude product was purified _via_ column chromatography (pentane:ethyl acetate = 95:5) to give **13a** (217 mg, 0.712 mmol, 89% yield) as a colorless oil.

^{1}H-NMR (400 MHz, CDCl3): 7.51 (dt, _J_ = 7.1, 1.4 Hz, 1H), 7.49 – 7.44 (m, 1H), 7.37 – 7.32 (m, 1H), 7.31 (dt, _J_ = 7.6, 1.6 Hz, 1H), 3.60 – 3.38 (m, 2H), 3.23 – 3.01 (m, 2H), 1.73 – 1.59 (m, 2H), 1.55 – 1.32 (m, 4H), 1.20 – 1.07 (m, 2H), 1.02 – 0.90 (m, 3H), 0.85 – 0.70 (m, 3H), 0.26 (s, 9H).
^{13}C-NMR (101 MHz, CDCl3): δ / ppm = 172.1, 140.9, 136.7, 134.0, 131.1, 127.7, 126.9, 48.9, 44.7, 31.1, 29.8, 20.5, 19.9, 14.1, 13.8, -1.1 (3C) (mixture of rotamers).
IR (Diamond-ATR, neat): $\tilde{\nu}$ / cm-1 = 2955, 2931, 2872, 1630, 1463, 1422, 1388, 1377, 1296, 1247, 1122, 1101, 835, 806, 746, 705, 691.

MS (EI, 70 eV): *m/z* (%) = 262 (12), 178 (10), 177 (100), 149 (22), 121 (10).
HRMS (EI): *m/z* calc. for [$C_{18}H_{31}NOSi^{·+}$]: 304.2091; found 304.2084 (M-H).

N,N-Dibutyl-3-cyanobenzamide (13b)

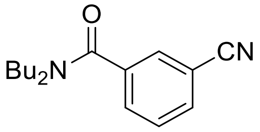

Following **TP6**, *N,N*-dibutylformamide (**4a**) (157 mg, 1.00 mmol, 1.0 equiv), $ZnCl_2$ (500 µL, 0.500 mmol, 1.0 equiv) and Et_3N (56.0 mg, 0.500 mmol, 1.0 equiv) were mixed in THF (2.0 mL) and a freshly prepared solution of TMPLi (1.10 mmol, 1.1 equiv) was dropwise added at 15 °C followed by Pd(dppf)Cl_2 (36.6 mg, 0.050 µmol, 0.05 equiv), 3-bromobenzonitrile (146 mg, 0.80 mmol, 0.8 equiv) and CuCN·2LiCl 1M in THF (20.0 µL, 0.020 mmol, 0.02 equiv). Thereafter, the reaction mixture was quenched with saturated $NH_4Cl_{(aq)}$ solution. After workup, the crude product was purified *via* column chromatography (pentane:ethyl acetate = 8:2) to give **13b** (165 mg, 0.640 mmol, 80% yield) as a colorless oil.

^{1}H-NMR (400 MHz, $CDCl_3$): δ / ppm = 7.69 – 7.64 (m, 1H), 7.64 – 7.62 (m, 1H), 7.59 – 7.55 (m, 1H), 7.51 (t, *J* = 7.7 Hz, 1H), 3.53 – 3.43 (m, 2H), 3.18 – 3.09 (m, 2H), 1.68 – 1.57 (m, 2H), 1.51 – 1.43 (m, 2H), 1.41 – 1.32 (m, 2H), 1.17 – 1.09 (m, 2H), 0.96 (t, *J* = 7.4 Hz, 3H), 0.77 (t, *J* = 7.3 Hz, 3H).
^{13}C-NMR (101 MHz, $CDCl_3$): δ / ppm = 169.2, 138.7, 132.7, 130.9, 130.2, 129.5, 118.2, 112.8, 48.9, 44.8, 30.8, 29.6, 20.3, 19.8, 14.0, 13.6 (mixture of rotamers).
IR (Diamond-ATR, neat): $\tilde{\nu}$ / cm-1 = 2957, 2931, 2871, 2230, 1670, 1629, 1464, 1429, 1413, 1376, 1297, 1199, 1094, 807, 747, 693, 656.
MS (EI, 70 eV): *m/z* (%) = 215 (18), 173 (16), 130 (100).
HRMS (EI): *m/z* calc. for [$C_{16}H_{22}N_2O^{·+}$]: 257.1648; found 257.1645.

Ethyl 3-(dibutylcarbamoyl)benzoate (13c)

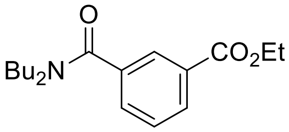

Following **TP6**, *N,N*-dibutylformamide (**4a**) (157 mg, 1.00 mmol, 1.0 equiv), $ZnCl_2$ (500 µL, 0.500 mmol, 1.0 equiv) and Et_3N (56.0 mg, 0.500 mmol, 1.0 equiv) were mixed in THF (2.0 mL) and a freshly prepared solution of TMPLi (1.10 mmol, 1.1 equiv) was dropwise added at 15 °C followed by Pd(dppf)Cl_2 (36.6 mg, 0.050 µmol, 0.05 equiv), ethyl 3-bromobenzoate (183 mg, 0.80 mmol, 0.8 equiv) and CuCN·2LiCl 1M in THF (20.0 µL, 0.020 mmol, 0.02 equiv). Thereafter, the reaction mixture was quenched with saturated $NH_4Cl_{(aq)}$ solution . After workup, the crude product was purified *via* column chromatography (pentane:ethyl acetate = 9:1) to give **13c** (207 mg, 0.680 mmol, 85% yield) as a colorless oil.

^{1}H-NMR (400 MHz, $CDCl_3$): δ / ppm = 8.05 (dt, *J* = 7.7, 1.5 Hz, 1H), 8.02 (td, *J* = 1.7, 0.6 Hz, 1H), 7.54 (dt, *J* = 7.6, 1.5 Hz, 1H), 7.46 (td, *J* = 7.7, 0.7 Hz, 1H), 4.37 (q, *J* = 7.1 Hz, 2H), 3.57 – 3.44 (m, 2H), 3.22 – 3.11 (m, 2H), 1.72 – 1.56 (m, 2H), 1.55 – 1.44 (m, 2H), 1.44 – 1.33 (m, 4H), 1.19 – 1.06 (m, 2H), 0.97 (t, *J* = 6.8 Hz, 3H), 0.77 (t, *J* = 6.7 Hz, 3H).
^{13}C-NMR (101 MHz, $CDCl_3$): δ / ppm = 170.7, 166.1, 137.7, 131.0, 130.7, 130.2, 128.7, 127.7, 61.3, 49.0, 44.8, 30.9, 29.8, 20.4, 19.8, 14.4, 14.1, 13.7 (mixture of rotamers).
IR (Diamond-ATR, neat): $\tilde{\nu}$ / cm-1 = 2956, 2931, 2872, 1718, 1630, 1464, 1418, 1367, 1251, 1227, 1178, 1121, 1097, 1080, 1022, 926, 823, 771, 731, 697, 680.
MS (EI, 70 eV): *m/z* (%) = 262 (18), 178 (11), 177 (100), 149 (37).

HRMS (EI): *m/z* calc. for [$C_{18}H_{27}NO_3$]: 305.1907; found 304.1903.

***N*,*N*-Dibutyl-4-(trifluoromethyl)benzamide (13d)**

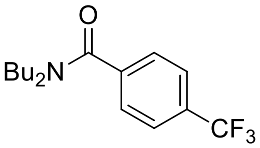

Following **TP6**, *N,N*-dibutylformamide (**4a**) (157 mg, 1.00 mmol, 1.0 equiv), $ZnCl_2$ (500 µL, 0.500 mmol, 1.0 equiv) and Et_3N (56.0 mg, 0.500 mmol, 1.0 equiv) were mixed in THF (2.0 mL) and a freshly prepared solution of TMPLi (1.10 mmol, 1.1 equiv) was dropwise added at 15 °C followed by Pd(dppf)Cl_2 (36.6 mg, 0.050 µmol, 0.05 equiv), 1-bromo-4-(trifluoromethyl)benzene (180 mg, 0.80 mmol, 0.8 equiv) and CuCN·2LiCl 1M in THF (20.0 µL, 0.020 mmol, 0.02 equiv). Thereafter, the reaction mixture was quenched with saturated $NH_4Cl_{(aq)}$ solution. After workup, the crude product was purified *via* column chromatography (pentane:ethyl acetate = 95:5) to give **13d** (207 mg, 0.690 mmol, 86% yield) as a colorless oil.

^{1}H-NMR (400 MHz, $CDCl_3$): δ / ppm = 7.65 (d, *J* = 8.0 Hz, 2H), 7.45 (d, *J* = 7.9 Hz, 2H), 3.58 – 3.38 (m, 2H), 3.22 – 3.05 (m, 2H), 1.72 – 1.56 (m, 2H), 1.55 – 1.29 (m, 4H), 1.19 – 1.06 (m, 2H), 0.97 (t, *J* = 7.3 Hz, 3H), 0.78 (t, *J* = 7.4 Hz, 3H).
^{13}C-NMR (101 MHz, $CDCl_3$): δ / ppm = 170.2, 141.0, 131.15 (q, *J* = 32.55 Hz), 127.0 (2C), 125.6 (q, J = 3.7 Hz) (2C), 123.95 (q, *J* = 272.15 Hz), 48.8, 44.7, 30.9, 29.7, 20.4, 19.9, 14.0, 13.7.
^{19}F-NMR (377 MHz, $CDCl_3$): δ / ppm = –62.8.
IR (Diamond-ATR, neat): $\tilde{\nu}$ / cm-1 = 2959, 2933, 1632, 1467, 1427, 1405, 1322, 1164, 1124, 1164, 1124, 1104, 1063, 1018, 849, 768, 737.
MS (EI, 70 eV): *m/z* (%) = 258 (15), 173 (100), 145 (18).
HRMS (EI): *m/z* calc. for [$C_{16}H_{22}F_3NO^{+}$]: 300.1570; found 300.1566.

***N*,*N*-Dibutyl-4-(trifluoromethoxy)benzamide (13e)**

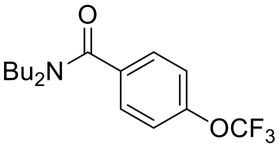

Following **TP6**, *N,N*-dibutylformamide (**4a**) (157 mg, 1.00 mmol, 1.0 equiv), $ZnCl_2$ (500 µL, 0.500 mmol, 1.0 equiv) and Et_3N (56.0 mg, 0.500 mmol, 1.0 equiv) were mixed in THF (2.0 mL) and a freshly prepared solution of TMPLi (1.10 mmol, 1.1 equiv) was dropwise added at 15 °C followed by Pd(dppf)Cl_2 (36.6 mg, 0.050 µmol, 0.05 equiv), 1-bromo-4-(trifluoromethoxy)benzene (193 mg, 0.80 mmol, 0.8 equiv) and CuCN·2LiCl 1M in THF (20.0 µL, 0.020 mmol, 0.02 equiv). Thereafter, the reaction mixture was quenched with saturated $NH_4Cl_{(aq)}$ solution. After workup, the crude product was purified *via* column chromatography (pentane:ethyl acetate = 95:5) to give **13e** (205 mg, 0.648 mmol, 81% yield) as a colorless oil.

^{1}H-NMR (400 MHz, $CDCl_3$): δ / ppm = ^{1}H NMR (400 MHz, Chloroform-*d*) δ 7.44 – 7.34 (m, 1H), 7.25 – 7.20 (m, 1H), 3.62 – 3.32 (m, 1H), 3.31 – 3.03 (m, 1H), 1.63 (s, 2H), 1.55 – 1.28 (m, 4H), 1.22 – 1.06 (m, 2H), 1.03 – 0.87 (m, 2H), 0.87 – 0.67 (m, 2H).
^{13}C-NMR (101 MHz, $CDCl_3$): δ / ppm = 170.4, 149.7 (q, *J* = 1.9 Hz), 136.1, 128.4 (2C), 120.9 (2C), 120.5 (*J* = 258.4 Hz), 48.9, 44.7, 30.9, 29.7, 20.4, 19.8, 14.0, 13.7 (mixture of rotamers).
^{19}F-NMR (377 MHz, $CDCl_3$): δ / ppm = –57.9.

IR (Diamond-ATR, neat): $\tilde{\nu}$ / cm-1 = 2959, 2932, 1631, 1467, 1425, 1251, 1218, 1159, 1099, 1020, 867, 764.
MS (EI, 70 eV): *m/z* (%) = 189 (100).
HRMS (EI): *m/z* calc. for [$C_{16}H_{22}F_3NO_2$]: 316.1519; found 316.1514.

***N,N*-Dibutyl-4-methoxybenzamide (13f)**

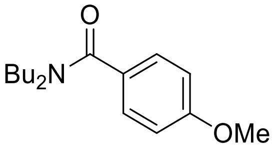

Following **TP6**, *N,N*-dibutylformamide (**4a**) (157 mg, 1.00 mmol, 1.0 equiv), $ZnCl_2$ (500 µL, 0.500 mmol, 1.0 equiv) and Et_3N (56.0 mg, 0.500 mmol, 1.0 equiv) were mixed in THF (2.0 mL) and a freshly prepared solution of TMPLi (1.10 mmol, 1.1 equiv) was dropwise added at 15 °C followed by Pd(dppf)Cl_2 (36.6 mg, 0.050 µmol, 0.05 equiv), 1-bromo-4-methoxybenzene (150 mg, 0.80 mmol, 0.8 equiv) and CuCN·2LiCl 1M in THF (20.0 µL, 0.020 mmol, 0.02 equiv). Thereafter, the reaction mixture was quenched with saturated $NH_4Cl_{(aq)}$ solution. After workup, the crude product was purified *via* column chromatography (pentane:ethyl acetate = 9:1) to give **13f** (130 mg, 0.496 mmol, 62% yield) as a colorless oil.

^{1}H-NMR (400 MHz, $CDCl_3$): 7.31 (d, *J* = 8.7 Hz, 2H), 6.88 (d, *J* = 8.7 Hz, 2H), 3.81 (s, 3H), 3.55 – 3.09 (m, 4H), 1.74 – 1.04 (m, 8H), 1.02 – 0.62 (m, 6H).
^{13}C-NMR (101 MHz, $CDCl_3$): δ / ppm = 171.6, 160.3, 129.7, 128.4 (2C), 113.7 (2C), 55.4, 49.1, 44.7, 30.9, 29.8, 20.3, 20.0, 13.9 (mixture of rotamers).
IR (Diamond-ATR, neat): $\tilde{\nu}$ / cm-1 = 2957, 2931, 2872, 1625, 1608, 1576, 1512, 1462, 1422, 1377, 1297, 1247, 1172, 1101, 1030, 956, 922, 838, 798, 764, 730.
MS (EI, 70 eV): *m/z* (%) = 262 (15), 135 (100).
HRMS (EI): *m/z* calc. for [$C_{16}H_{25}NO_2$]: 263.1802; found 263.1801.

Methyl 6-(dibutylcarbamoyl)-2-naphthoate (13g)

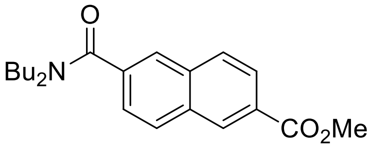

Following **TP6**, *N,N*-dibutylformamide (**4a**) (157 mg, 1.00 mmol, 1.0 equiv), $ZnCl_2$ (500 µL, 0.500 mmol, 1.0 equiv) and Et_3N (56.0 mg, 0.500 mmol, 1.0 equiv) were mixed in THF (2.0 mL) and a freshly prepared solution of TMPLi (1.10 mmol, 1.1 equiv) was dropwise added at 15 °C followed by Pd(dppf)Cl_2 (36.6 mg, 0.050 µmol, 0.05 equiv), methyl 6-bromo-2-naphthoate (212 mg, 0.80 mmol, 0.8 equiv) and CuCN·2LiCl 1M in THF (20.0 µL, 0.020 mmol, 0.02 equiv). Thereafter, the reaction mixture was quenched with saturated $NH_4Cl_{(aq)}$ solution. After workup, the crude product was purified *via* column chromatography (pentane:ethyl acetate = 95:5) to give **13g** (254 mg, 0.744 mmol, 93% yield) as a colorless oil.

^{1}H-NMR (400 MHz, $CDCl_3$):f 8.63 – 8.56 (m, 1H), 8.07 (dd, *J* = 8.6, 1.7 Hz, 1H), 7.95 (d, *J* = 8.4 Hz, 1H), 7.87 (d, *J* = 8.6 Hz, 1H), 7.85 – 7.83 (m, 1H), 7.49 (dd, *J* = 8.4, 1.6 Hz, 1H), 3.95 (s, 3H), 3.61 – 3.46 (m, 2H), 3.25 – 3.11 (m, 2H), 1.80 – 1.59 (m, 2H), 1.54 – 1.34 (m, 4H), 1.17 – 1.02 (m, 2H), 1.01 – 0.92 (m, 3H), 0.78 – 0.64 (m, 3H).
^{13}C-NMR (101 MHz, $CDCl_3$): δ / ppm = 171.0, 167.0, 137.1, 134.9, 132.4, 130.8, 129.7, 128.5, 128.2, 126.0, 125.7, 125.0, 52.3, 48.9, 44.6, 30.9, 29.7, 20.4, 19.7, 14.0, 13.6 (mixture of rotamers).

IR (Diamond-ATR, neat): $\tilde{\nu}$ / cm-1 = 2953, 2871, 1712, 1612, 1489, 1465, 1437, 1380, 1338, 1320, 1298, 1276, 1242, 1226, 1188, 1172, 1140, 1104, 1094, 994, 947, 936, 926, 876, 833, 815, 780, 764, 733, 718.
MS (EI, 70 eV): *m/z* (%) = 341 (11), 340 (15), 298 (14), 214 (14), 213 (20), 213 (100), 154 (11), 126 (10).
HRMS (EI): *m/z* calc. for [$C_{21}H_{27}NO_3$]: 340.1907; found 340.1904.
M.p. (°C): 83.7-85.1.
X-Ray: Crystals suitable for X-Ray diffraction were obtained from $CDCl_3$ by slow evaporation of the solvent at rt. See pages 149-153.

***N*,*N*-Dibutyl-2-methyl-1-naphthamide (13h)**

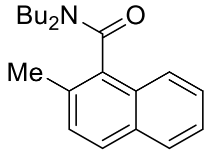

Following **TP6**, *N,N*-dibutylformamide (**4a**) (157 mg, 1.00 mmol, 1.0 equiv), $ZnCl_2$ (500 µL, 0.500 mmol, 1.0 equiv) and Et_3N (56.0 mg, 0.500 mmol, 1.0 equiv) were mixed in THF (2.0 mL) and a freshly prepared solution of TMPLi (1.10 mmol, 1.1 equiv) was dropwise added at 15 °C followed by Pd(dppf)Cl_2 (36.6 mg, 0.050 µmol, 0.05 equiv), 1-bromo-2-methylnaphthalene (177 mg, 0.80 mmol, 0.8 equiv) and CuCN·2LiCl 1M in THF (20.0 µL, 0.020 mmol, 0.02 equiv). Thereafter, the reaction mixture was quenched with saturated $NH_4Cl_{(aq)}$ solution. After workup, the crude product was purified *via* column chromatography (pentane:ethyl acetate = 95:5) to give **13h** (206 mg, 0.694 mmol, 87% yield) as a colorless oil.

^{1}H-NMR (400 MHz, $CDCl_3$): 7.79 (d, *J* = 7.3 Hz, 1H), 7.74 (d, *J* = 8.4 Hz, 1H), 7.67 (d, *J* = 8.3 Hz, 1H), 7.48 – 7.44 (m, 1H), 7.44 – 7.40 (m, 1H), 7.31 (d, *J* = 8.4 Hz, 1H), 3.73 – 3.67 (m, 1H), 3.63 – 3.57 (m, 1H), 3.00 – 2.95 (m, 2H), 2.43 (s, 3H), 1.83 – 1.75 (m, 2H), 1.48 (h, *J* = 7.4 Hz, 2H), 1.41 – 1.27 (m, 2H), 1.04 (t, *J* = 7.4 Hz, 3H), 0.95 (h, *J* = 7.4 Hz, 2H), 0.58 (t, *J* = 7.4 Hz, 3H).
^{13}C-NMR (101 MHz, $CDCl_3$): 170.3, 133.5, 131.9, 131.4, 130.0, 128.5, 128.3, 128.1, 126.8, 125.5, 124.8, 48.3, 44.5, 30.7, 29.8, 20.7, 19.9, 19.7, 14.1, 13.5 (mixture of rotamers).
IR (Diamond-ATR, neat): $\tilde{\nu}$ / cm-1 = 2957, 2930, 2872, 1626, 1510, 1466, 1424, 1377, 1294, 1254, 1218, 1129, 864, 810, 786, 742, 675.
MS (EI, 70 eV): *m/z* (%) = 282 (19), 170 (13), 168 (11), 141 (44), 115 (20).
HRMS (EI): *m/z* calc. for [$C_{20}H_{27}NO$]: 297.2093; found 297.2089.

***N*,*N*-Dibutylquinoline-2-carboxamide (13i)**

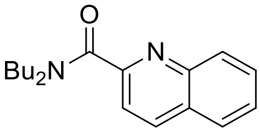

Following **TP6**, *N,N*-dibutylformamide (**4a**) (157 mg, 1.00 mmol, 1.0 equiv), $ZnCl_2$ (500 µL, 0.500 mmol, 1.0 equiv) and Et_3N (56.0 mg, 0.500 mmol, 1.0 equiv) were mixed in THF (2.0 mL) and a freshly prepared solution of TMPLi (1.10 mmol, 1.1 equiv) was dropwise added at 15 °C followed by Pd(dppf)Cl_2 (36.6 mg, 0.050 µmol, 0.05 equiv), 2-bromoquinoline (166 mg, 0.80 mmol, 0.8 equiv) and CuCN·2LiCl 1M in THF (20.0 µL, 0.020 mmol, 0.02 equiv). Thereafter, the reaction mixture was quenched with saturated $NH_4Cl_{(aq)}$ solution. After workup, the crude product was purified *via* column chromatography (pentane:ethyl acetate = 9:1) to give **13i** (202 mg, 0.712 mmol, 89% yield) as a colorless oil.

^{1}H-NMR (400 MHz, CDCl$_3$): δ / ppm = ^{1}H NMR (400 MHz, Chloroform-*d*) δ 8.22 (d, *J* = 8.4 Hz, 1H), 8.12 – 8.03 (m, 1H), 7.82 (dd, *J* = 8.2, 1.4 Hz, 1H), 7.76 – 7.70 (m, 1H), 7.66 (d, *J* = 8.4 Hz, 1H), 7.60 – 7.54 (m, 1H), 3.60 – 3.52 (m, 2H), 3.47 – 3.34 (m, 2H), 1.76 – 1.59 (m, 4H), 1.44 (h, *J* = 7.4 Hz, 2H), 1.14 (h, *J* = 7.4 Hz, 2H), 0.99 (t, *J* = 7.3 Hz, 3H), 0.77 (t, *J* = 7.4 Hz, 3H).
^{13}C-NMR (101 MHz, CDCl$_3$): δ / ppm = 168.9, 154.9, 146.7, 136.9, 129.9, 129.8, 128.0, 127.7, 127.4, 120.7, 48.9, 46.0, 31.2, 29.9, 20.5, 20.0, 14.1, 13.8.
IR (Diamond-ATR, neat): $\tilde{\nu}$ / cm-1 = 2956, 2929, 2871, 1625, 1597, 1561, 1479, 1466, 1420, 1374, 1294, 1126, 1098, 944, 838, 775, 765, 734.
MS (EI, 70 eV): *m/z* (%) = 129 (21), 128 (100), 128 (45).
HRMS (EI): *m/z* calc. for [$C_{18}H_{24}N_2O$]: 284.1889; found 285.1962 (M+H).

***N*,*N*-Dibutyl-6-methoxypicolinamide (13j)**

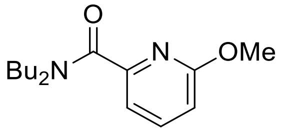

Following **TP6**, *N,N*-dibutylformamide (**4a**) (157 mg, 1.00 mmol, 1.0 equiv), $ZnCl_2$ (500 µL, 0.500 mmol, 1.0 equiv) and Et_3N (56.0 mg, 0.500 mmol, 1.0 equiv) were mixed in THF (2.0 mL) and a freshly prepared solution of TMPLi (1.10 mmol, 1.1 equiv) was dropwise added at 15 °C followed by Pd(dppf)Cl_2 (36.6 mg, 0.050 µmol, 0.05 equiv), 2-bromo-6-methoxypyridine (150 mg, 0.80 mmol, 0.8 equiv) and CuCN·2LiCl 1M in THF (20.0 µL, 0.020 mmol, 0.02 equiv). Thereafter, the reaction mixture was quenched with saturated $NH_4Cl_{(aq)}$ solution. After workup, the crude product was purified *via* column chromatography (pentane:ethyl acetate = 9:1) to give **13j** (196 mg, 0.744 mmol, 93% yield) as a colorless oil.

^{1}H-NMR (400 MHz, CDCl$_3$): δ / ppm = 7.61 (dd, *J* = 8.4, 7.3 Hz, 1H), 7.11 (dd, *J* = 7.3, 0.8 Hz, 1H), 6.74 (dd, *J* = 8.4, 0.8 Hz, 1H), 3.90 (s, 3H), 3.52 – 3.42 (m, 2H), 3.32 – 3.23 (m, 2H), 1.71 – 1.53 (m, 4H), 1.46 – 1.33 (m, 2H), 1.22 – 1.11 (m, 2H), 0.96 (t, *J* = 7.3 Hz, 3H), 0.80 (t, *J* = 7.3 Hz, 3H).
^{13}C-NMR (101 MHz, CDCl$_3$): δ / ppm = 168.7, 162.8, 152.8, 139.3, 116.1, 111.6, 53.6, 48.7, 45.6, 31.2, 29.7, 20.4, 20.1, 14.1, 13.8.
IR (Diamond-ATR, neat): $\tilde{\nu}$ / cm-1 = 2956, 2930, 2871, 1630, 1593, 1574, 1461, 1415, 1407, 1376, 1320, 1288, 1262, 1235, 1195, 1148, 1124, 1075, 1031, 987, 814, 763, 733, 712.
MS (EI, 70 eV): *m/z* (%) = 128 (100), 108 (22).
HRMS (EI): *m/z* calc. for [$C_{15}H_{24}N_2O_2$]: 264.1838; found 265.1909 (M+H).

***N*,*N*-Dibutyl-3-((5-(4-fluorophenyl)thiophen-2-yl)methyl)-4-methylbenzamide (13k)**

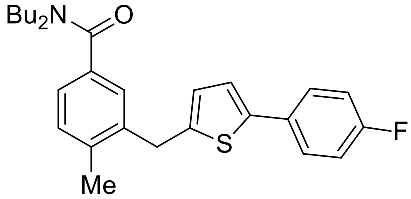

Following **TP6**, *N,N*-dibutylformamide (**4a**) (157 mg, 1.00 mmol, 1.0 equiv), $ZnCl_2$ (500 µL, 0.500 mmol, 1.0 equiv) and Et_3N (56.0 mg, 0.500 mmol, 1.0 equiv) were mixed in THF (2.0 mL) and freshly prepared solution of TMPLi (1.10 mmol, 1.1 equiv) was dropwise added at 15 °C followed by Pd(dppf)Cl_2 (36.6 mg, 0.050 µmol, 0.05 equiv), 2-(5-bromo-2-methylbenzyl)-5-(4-fluorophenyl)thiophene (289 mg, 0.80 mmol, 0.8 equiv) and CuCN·2LiCl 1M in THF (20.0 µL, 0.020 mmol, 0.02 equiv). Thereafter, the reaction mixture was quenched with saturated

$NH_4Cl_{(aq)}$ solution. After workup, the crude product was purified *via* column chromatography (pentane:ethyl acetate = 9:1) to give **13k** (273 mg, 0.624 mmol, 78% yield) as a colorless oil (turnes dark over time if not stored under Ar).

Scale up procedure (**modified TP6**):
A heat and vacuum dried 100 mL pressure tube was filled with argon and placed into a ice/water bath at 0 °C. *N*,*N*-Dibutylformamide (1.97 g, 12.5 mmol, 1.0 equiv), $ZnCl_2$ solution 1 M in THF (6.25 mL, 6.25 mmol, 0.5 equiv), Et_3N (632 mg, 6.25 mmol, 0.5 equiv) and THF (25 mL) were added. Freshly prepared TMPLi as ca. 0.5 M solution in THF (15.0 mmol, 1.2 equiv) was dropwise added over ca. 30 min. The reaction mixture was stirred for an additional 1 h at 0 °C. A solution of dicarbamoylzinc reagent $(Bu_2NCO)_2Zn$ was obtained. In a separate flask $Pd(dppf)Cl_2$ (91.5 mg, 0.125 mmol, 1 mol%), 2-(5-bromo-2-methylbenzyl)-5-(4-fluorophenyl)thiophene (3.61 g, 10.0 mmol, 0.8 equiv) and THF (10 mL) were added to form a fine suspension. The suspension was quantitatively transfered (the flask and the syringe were washed with THF 3 x 2 mL) into a solution of the dicarbamoylzinc reagent followed by a 1 M solution of CuCN·2LiCl (50 µL, 50.0 µmol, 0.4 mol%). The pressure tube was sealed using a screw cap (with rubber O-ring) and reaction mixture was vigorously stirred at 45 °C for 72 h. After cooling the reaction mixture to 22 °C, the sealed tube was carefully opened and saturated $NH_4Cl_{(aq)}$ (10 mL) was dropwise added. Bubbling was observed (CO gas release from slight excess of dicarbamoylzinc reagent). The reaction mixture was transferred into a separating funnel containing diluted $NH_4Cl_{(aq)}$ (100 mL) and was extracted with ethyl acetate (3 x 200 mL). Organic layer was dried with $MgSO_4$, filtered and the solvent was removed *in vacuo*, Flash column chromatography purification with pentane:ethyl acetate =9:1 to 8:2 afforded analytically pure product *N*,*N*-dibutyl-3-((5-(4-fluorophenyl)thiophen-2-yl)methyl)-4-methylbenzamide **13k** (4.08 g, 9.33 mmol, 93% yield) as a yellow oil.

^{1}H-NMR (400 MHz, $CDCl_3$): 7.49 – 7.42 (m, 2H), 7.22 (s, 1H), 7.18 (s, 2H), 7.06 – 6.97 (m, 3H), 6.71 – 6.65 (m, 1H), 4.12 (s, 2H), 3.57 – 3.36 (m, 2H), 3.32 – 3.06 (m, 2H), 2.33 (s, 3H), 1.78 – 1.55 (m, 2H), 1.54 – 1.28 (m, 4H), 1.24 – 1.06 (m, 2H), 1.03 – 0.89 (m, 3H), 0.86 – 0.68 (m, 3H).
^{13}C-NMR (101 MHz, $CDCl_3$): δ / ppm = 171.6, 162.13 (d, *J* = 246.8 Hz), 143.0, 141.7, 138.3, 137.5, 135.4, 130.86 (d, *J* = 3.4 Hz), 127.7 (d, *J* = 4.1 Hz), 127.40 – 126.95 (m), 126.1 (d, *J* = 6.2 Hz), 125.2, 122.7, 115.7 (d, *J* = 21.7 Hz), 49.0, 44.7, 34.0, 31.0, 29.7, 20.4, 20.0, 19.4, 19.4, 13.9, 13.8.
^{19}F-NMR (377 MHz, $CDCl_3$): δ / ppm = –115.1.
IR (Diamond-ATR, neat): $\tilde{\nu}$ / cm-1 = 2956, 2928, 2870, 1626, 1508, 1464, 1423, 1377, 1299, 1230, 1158, 1096, 831, 799, 756.
MS (EI, 70 eV): *m/z* (%) = 394 (14), 310 (20), 309 (100), 191 (19), 131 (21).
HRMS (EI): *m/z* calc. for $[C_{27}H_{32}FNOS^{\cdot+}]$: 436.2105; found 436.2100.

3-(4-(4-Cyanophenyl)piperazine-1-carbonyl)benzonitrile (13l)

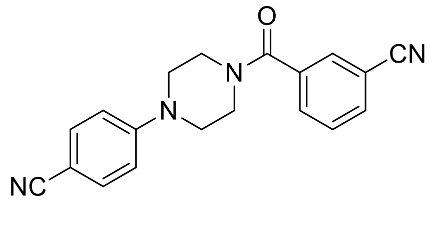

Following **TP6**, 4-(4-formylpiperazin-1-yl)benzonitrile (**4p**) (215 mg, 1.00 mmol, 1.0 equiv), $ZnCl_2$ (500 µL, 0.500 mmol, 1.0 equiv) and Et_3N (56.0 mg, 0.500 mmol, 1.0 equiv) were mixed in THF (2.0 mL) and a freshly prepared solution of TMPLi (1.10 mmol, 1.1 equiv) was dropwise added at 15 °C followed by $Pd(dppf)Cl_2$ (36.6 mg, 0.050 µmol, 0.05 equiv), 3-bromobenzonitrile (146 mg, 0.80 mmol, 0.8 equiv) and CuCN·2LiCl 1M in THF (20.0 µL, 0.020

mmol, 0.02 equiv). Thereafter, the reaction mixture was quenched with saturated $NH_4Cl_{(aq)}$ solution. After workup, the crude product was purified *via* column chromatography (pentane:ethyl acetate = 1:1 to 4:6) to give **13l** (179 mg, 0.568 mmol, 71% yield) as a non-crystaline solid.

^{1}H-NMR (400 MHz, CDCl$_3$): δ / ppm = 7.75 – 7.69 (m, 2H), 7.66 (dt, *J* = 7.9, 1.4 Hz, 1H), 7.58 – 7.53 (m, 1H), 7.50 – 7.45 (m, 2H), 6.85 (d, *J* = 9.0 Hz, 1H), 3.99 – 3.83 (m, 2H), 3.61 – 3.50 (m, 2H), 3.47 – 3.37 (m, 2H), 3.36 – 3.25 (m, 2H).
^{13}C-NMR (101 MHz, CDCl$_3$): δ / ppm = 168.0, 152.9, 136.4, 133.6 (2C), 133.6, 131.5, 130.8, 129.7, 119.8, 117.9, 114.8 (2C), 113.0, 101.3, 47.7, 47.0 (2C), 41.9.
IR (Diamond-ATR, neat): $\tilde{\nu}$ / cm-1 = 2214, 1632, 1602, 1680, 1515, 1486, 1460, 1438, 1415, 1391, 1287, 1244, 1221, 1198, 1179, 1139, 1016, 940, 902, 819, 797, 726, 685.
MS (EI, 70 eV): *m/z* (%) = 184 (17), 158 (10), 157 (100), 145 (19), 144 (23), 130 (19), 130 (24), 129 (60).
HRMS (EI): *m/z* calc. for [$C_{19}H_{16}N_4O$]: 316.1324; found 316.1318.

(4-(3-(Trifluoromethyl)pyridin-2-yl)piperazin-1-yl)(3-(trimethylsilyl)phenyl)methanone (13m)

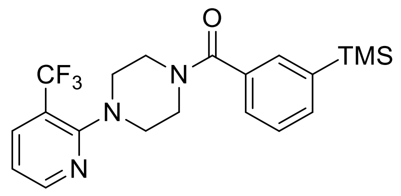

Following **TP6**, 4-(3-(trifluoromethyl)pyridin-2-yl)piperazine-1-carbaldehyde (**4f**) (157 mg, 1.00 mmol, 1.0 equiv), $ZnCl_2$ (500 µL, 0.500 mmol, 1.0 equiv) and Et_3N (56.0 mg, 0.500 mmol, 1.0 equiv) were mixed in THF (2.0 mL) and a freshly prepared solution of TMPLi (1.10 mmol, 1.1 equiv) was dropwise added at 15 °C followed by Pd(dppf)Cl_2 (36.6 mg, 0.050 µmol, 0.05 equiv), (3-bromophenyl)trimethylsilane (183 mg, 0.80 mmol, 0.8 equiv) and CuCN·2LiCl 1M in THF (20.0 µL, 0.020 mmol, 0.02 equiv). Thereafter, the reaction mixture was quenched with saturated $NH_4Cl_{(aq)}$ solution. After workup, the crude product was purified *via* column chromatography (pentane:ethyl acetate = 8:2) to give **13m** (173 mg, 0.424 mmol, 53% yield) as a yellow amorphous solid.

^{1}H-NMR (400 MHz, CDCl3): δ / ppm = 8.46 – 8.43 (m, 1H), 7.91 – 7.87 (m, 1H), 7.58 – 7.54 (m, 2H), 7.40 – 7.35 (m, 2H), 7.08 – 7.03 (m, 1H), 4.02 – 3.79 (m, 2H), 3.69 – 3.48 (m, 2H), 3.41 – 3.13 (m, 4H), 0.27 (s, 9H).
^{13}C-NMR (101 MHz, CDCl3): δ / ppm = 170.9 , 159.6 , 151.2 , 141.3 , 137.1 (q, *J* = 5.0 Hz), 135.0 , 134.6 , 131.7 , 127.6 , 127.2 , 123.8 (q, *J* = 272.6 Hz), 117.9 , 117.8 (q, *J* = 31.5 Hz) 51.0 (2C) , 47.8 , 42.2 , -1.2 (3C).
^{19}**F-NMR (377 MHz**, **CDCl3):** δ / ppm = –60.3.
IR (Diamond-ATR, neat): $\tilde{\nu}$ / cm-1 = 1634, 1590, 1568, 1430, 1390, 1377, 1369, 1309, 1285, 1248, 1234, 1165, 1145, 1115, 1105, 1081, 1018, 942, 896, 837, 807, 796, 779, 748, 732, 694.
MS (EI, 70 eV): *m/z* (%) =214 (13), 213 (25), 201 (38), 189 (27), 188 (22), 187 (64), 181 (12), 178 (10), 177 (100), 175 (22), 173 (16), 155 (11), 149 (19), 128 (15), 121 (17), 119 (12).
HRMS (EI): *m/z* calc. for [$C_{20}H_{24}F_3N_3OSi$]: 407.1641; found 408.1721 (M+H).

***N*-(3-(1,3-dioxoisoindolin-2-yl)propyl)-*N*-isopropyl-6-methoxypicolinamide (13n)**

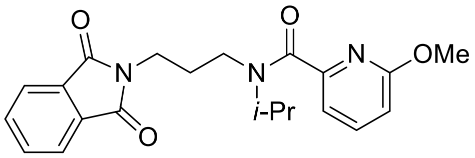

Following modified **TP6**, *N*-(3-(1,3-dioxoisoindolin-2-yl)propyl)-*N*-isopropylformamide (**4m**) (548 mg, 2.00 mmol, 1.0 equiv), and TMPZn·2$MgCl_2$·2LiCl solution 0.33M in THF (3.64 mL, 1.20 mmol, 1.2 equiv) were stirred under Ar atmosphere for 24 h and the mixture was diluted with THF (5 mL). Pd(dppf)Cl_2 (36.6 mg, 0.025 µmol, 0.025 equiv), 2-bromo-6-methoxypyridine (180 mg, 1.00 mmol, 0.5 equiv) and CuCN·2LiCl 1M in THF (20.0 µL, 0.020 mmol, 0.02 equiv) were added. After 24 h, the reaction mixture was quenched with saturated $NH_4Cl_{(aq)}$ solution. After extraction with DCM, the crude product was purified *via* column chromatography (pentane:ethyl acetate = 1:1) to give **13n** (249 mg, 0.731 mmol, 73% yield) as a colorless thick oil.

^{1}H-NMR (400 MHz, $CDCl_3$): δ / ppm = 7.81 – 7.75 (m, 2H), 7.66 – 7.62 (m, 2H), 7.58 – 7.53 (m, 1H), 7.05 – 7.00 (m, 1H), 6.71 – 6.64 (m, 1H), 4.09 – 4.02 (m, 1H), 3.86 – 3.80 (m, 3H), 3.74 – 3.71 (m, 2H), 3.41 – 3.35 (m, 2H), 2.12 – 2.03 (m, 2H), 1.16 – 1.08 (m, 6H).
(major rotamer).
7.75 – 7.70 (m, 2H), 7.70 – 7.66 (m, 2H), 7.36 – 7.30 (m, 1H), 6.91 – 6.84 (m, 1H), 6.19 (d, *J* = 8.1 Hz, 1H), 4.71 – 4.61 (m, 1H), 3.74 – 3.73 (m, 3H), 3.47 – 3.41 (m, 2H), 3.31 – 3.22 (m, 2H), 1.89 – 1.80 (m, 2H), 1.30 – 1.20 (m, 6H). (minor rotamer)
^{13}C-NMR (101 MHz, CDCl3): δ / ppm = 168.8, 168.6, 168.3 (2C), 168.0 (2C), 162.7, 162.6, 152.8, 152.5, 139.2, 138.9, 134.0 (2C), 133.9 (2C), 132.0 (2C), 131.7 (2C), 123.2 (2C), 123.1 (2C), 115.6, 114.9, 111.6, 111.0, 77.2, 53.5, 53.5, 49.8, 5.16, 41.9, 38.5, 36.1, 35.3, 30.6, 28.6, 21.3 (2C), 20.5 (2C).
IR (Diamond-ATR, neat): $\tilde{\nu}$ / cm-1 = 1770, 1707, 1626, 1595, 1574, 1464, 1437, 1413, 1395, 1358, 1327, 1295, 1262, 1208, 1187, 1126, 1074, 1023, 987, 913, 888, 815, 764, 717.
MS (EI, 70 eV): *m/z* (%) = 246 (15), 245 (100), 188 (29), 160 (39), 136 (13), 136 (15), 109 (20), 108 (59), 98 (41), 93 (13).
HRMS (EI): *m/z* calc. for [$C_{21}H_{23}N_3O_4$]: 381.1683; found 381.1678.

***N*,*N*-Dibutylcinnamamide (13o)**

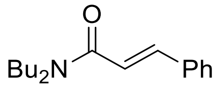

Following **TP6**, *N,N*-dibutylformamide (**4a**) (157 mg, 1.00 mmol, 1.0 equiv), $ZnCl_2$ (500 µL, 0.500 mmol, 1.0 equiv) and Et_3N (56.0 mg, 0.500 mmol, 1.0 equiv) were mixed in THF (2.0 mL) and a freshly prepared solution of TMPLi (1.10 mmol, 1.1 equiv) was dropwise added at 15 °C followed by Pd(dppf)Cl_2 (36.6 mg, 0.050 µmol, 0.05 equiv), (*E*)-(2-bromovinyl)benzene (146 mg, 0.80 mmol, 0.8 equiv) and CuCN·2LiCl 1M in THF (20.0 µL, 0.020 mmol, 0.02 equiv). Thereafter, the reaction mixture was quenched with saturated $NH_4Cl_{(aq)}$ solution. After workup, the crude product was purified *via* column chromatography (pentane:ethyl acetate = 9:1 to 8:2) to give **13o** (168 mg, 0.648 mmol, 81% yield) as a colorless oil.

^{1}H-NMR (400 MHz, $CDCl_3$): δ / ppm = ^{1}H NMR (600 MHz, Chloroform-*d*) δ 7.69 (d, *J* = 15.5 Hz, 1H), 7.51 – 7.47 (m, 2H), 7.38 – 7.29 (m, 3H), 6.82 (d, *J* = 15.3 Hz, 1H), 3.45 – 3.39 (m, 2H), 3.39 – 3.35 (m, 2H), 1.64 – 1.59 (m, 2H), 1.58 – 1.52 (m, 2H), 1.41 – 1.29 (m, 4H), 1.00 – 0.89 (m, 6H).
^{13}C-NMR (101 MHz, $CDCl_3$): δ / ppm = 166.0, 142.2, 135.6, 129.4, 128.8 (2C), 127.8 (2C), 117.9, 77.2, 48.0, 46.7, 32.0, 30.1, 20.4, 20.2, 14.0, 13.9.
IR (Diamond-ATR, neat): $\tilde{\nu}$ / cm-1 = 2957, 2930, 2872, 1648, 1603, 1578, 1496, 1453, 1439, 1422, 1372, 1325, 1301, 1251, 1208, 1139, 1113, 1101, 976, 854, 762, 733, 706, 684, 668.

MS (EI, 70 eV): *m/z* (%) = 131 (100), 103 (38).
HRMS (EI): *m/z* calc. for [$C_{19}H_{25}NO_2$]: 259.1936; found 260.2008 (M+H).

(*E*)-3-(4-Methoxyphenyl)-1-(trans-octahydroquinolin-1(2H)-yl)prop-2-en-1-one (13p)

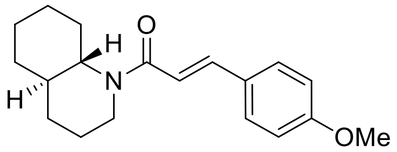

Following **TP6**, *trans*-octahydroisoquinoline-2(1H)-carbaldehyde (**4a**) (167 mg, 1.00 mmol, 1.0 equiv), $ZnCl_2$ (500 µL, 0.500 mmol, 1.0 equiv) and Et_3N (56.0 mg, 0.500 mmol, 1.0 equiv) were mixed in THF (2.0 mL) and a freshly prepared solution of TMPLi (1.10 mmol, 1.1 equiv) was dropwise added at 15 °C followed by Pd(dppf)Cl_2 (36.6 mg, 0.050 µmol, 0.05 equiv), (E)-1-(2-bromovinyl)-4-methoxybenzene (170 mg, 0.80 mmol, 0.8 equiv) and CuCN·2LiCl 1M in THF (20.0 µL, 0.020 mmol, 0.02 equiv). Thereafter, the reaction mixture was quenched with saturated $NH_4Cl_{(aq)}$ solution. After workup, the crude product was purified *via* column chromatography (pentane:ethyl acetate = 9:1 to 8:2) to give **13p** (144 mg, 0.480 mmol, 60% yield) as a colorless oil.

^{1}H-NMR (400 MHz, $CDCl_3$): δ / ppm = 7.61 (d, *J* = 15.3 Hz, 1H), 7.47 – 7.41 (m, 2H), 6.89 – 6.84 (m, 2H), 6.67 (d, *J* = 15.3 Hz, 1H), 4.13 – 4.01 (m, 1H), 3.79 (s, 3H), 3.44 – 3.35 (m, 1H), 3.20 – 3.09 (m, 1H), 2.14 – 2.06 (m, 1H), 1.88 – 1.76 (m, 2H), 1.75 – 1.66 (m, 3H), 1.65 – 1.54 (m, 2H), 1.52 – 1.39 (m, 2H), 1.38 – 1.22 (m, 2H), 1.21 – 1.02 (m, 2H).
^{13}C-NMR (101 MHz, $CDCl_3$): δ / ppm = 166.4, 160.7, 141.4, 129.2 (2C), 128.4, 116.2, 114.2 (2C), 61.8, 55.4, 38.6, 37.8, 33.1, 32.0, 26.3, 26.1, 25.55, 23.0.
IR (Diamond-ATR, neat): $\tilde{\nu}$ / cm-1 = 2925, 2855, 1642, 1593, 1575, 1510, 1459, 1424, 1413, 1360, 1304, 1286, 1250, 1233, 1219, 1171, 1134, 1112, 1030, 1009, 979, 824, 778, 729.
MS (EI, 70 eV): *m/z* (%) = 167 (10), 166 (100), 161 (28), 138 (93), 133 (10), 86 (14), 81 (17).
HRMS (EI): *m/z* calc. for [$C_{19}H_{25}NO_2$]: 299.1885; found 299.1879.

4-((*Z*)-3-Oxo-3-((1R,3r,5S)-3-((triethylsilyl)oxy)-8-azabicyclo[3.2.1]octan-8-yl)prop-1-en-1-yl)benzonitrile (13q)

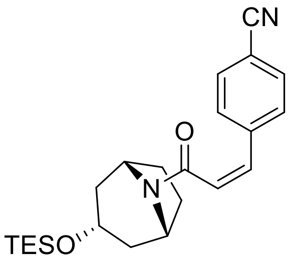

Following **TP6**, (1R,3r,5S)-3-((triethylsilyl)oxy)-8-azabicyclo[3.2.1]octane-8-carbaldehyde (**4a**) (269 mg, 1.00 mmol, 1.0 equiv), $ZnCl_2$ (500 µL, 0.500 mmol, 1.0 equiv) and Et_3N (56.0 mg, 0.500 mmol, 1.0 equiv) were mixed in THF (2.0 mL) and a freshly prepared solution of TMPLi (1.10 mmol, 1.1 equiv) was dropwise added at 15 °C followed by Pd(dppf)Cl_2 (36.6 mg, 0.050 µmol, 0.05 equiv), (*Z*)-4-(2-bromovinyl)benzonitrile (166 mg, 0.80 mmol, 0.8 equiv) and CuCN·2LiCl 1M in THF (20.0 µL, 0.020 mmol, 0.02 equiv). Thereafter, the reaction mixture was quenched with saturated $NH_4Cl_{(aq)}$ solution. After workup, the crude product was purified *via* column chromatography (pentane:ethyl acetate = 65:35) to give **13q** (196 mg, 0.496 mmol, 62% yield) as a colorless oil.

^{1}H-NMR (400 MHz, $CDCl_3$): δ / ppm = 7.63 – 7.47 (m, 4H), 6.61 (d, *J* = 12.5 Hz, 1H), 6.18 (d, *J* = 12.5 Hz, 1H), 4.73 – 4.59 (m, 1H), 4.00 – 3.94 (m, 1H), 3.92 – 3.87 (m, 1H), 2.28 – 2.19

(m, 1H), 2.17 – 2.09 (m, 1H), 2.03 – 1.95 (m, 1H), 1.77 – 1.61 (m, 2H), 1.61 – 1.48 (m, 2H), 1.45 – 1.33 (m, 1H), 0.93 – 0.84 (m, 9H), 0.55 – 0.46 (m, 6H).
^{13}C-NMR (101 MHz, $CDCl_3$): δ / ppm = 162.6, 140.1, 132.1 (2C), 132.0, 129.2 (2C), 126.8, 118.6, 111.8, 65.0, 55.4, 50.7, 41.1, 39.3, 28.4, 27.1, 6.9, 4.7.
IR (Diamond-ATR, neat): $\tilde{\nu}$ / cm-1 = 2952, 2912, 2875, 2227, 1612, 1505, 1453, 1410, 1370, 1322, 1313, 1238, 1223, 1169, 1085, 1051, 1004, 963, 923, 875, 845, 782, 724.
MS (EI, 70 eV): *m/z* (%) = 368 (17), 167 (74), 298 (41), 212 (15), 182 (18), 181 (10), 157 (10), 157 (10), 156 (98), 129 (16), 128 (100), 110 (51), 108 (10), 103 (17), 101 (10), 91 (18), 87 (18), 82 (12), 80 (10), 75 (38), 68 (14), 59 (10).
HRMS (EI): *m/z* calc. for [$C_{23}H_{32}N_2O_2Si$]: 396.2233; found 397.2313 (M+H).

N,*N*-Dibutyl-3-oxocyclohex-1-enecarboxamide (13r)

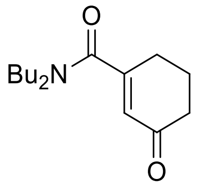

Following modified **TP5**, *N,N*-dibutylformamide (**4a**) (314 mg, 2.00 mmol, 2.0 equiv), $ZnCl_2$ (1.00 mL, 1.00 mmol, 1.0 equiv) and Et_3N (101 mg, 1.00 mmol, 1.0 equiv) were mixed in THF (4.0 mL) and a freshly prepared solution of TMPLi (2.20 mmol, 2.2 equiv) was dropwise added at 15 °C. After cooling to −78 °C, CuCN·2LiCl (1.00 mL, 1.00 mmol, 1.0 equiv) and 3-iodocyclohex-2-enone (222 mg, 1.00 mmol, 1.0 equiv). Thereafter, the reaction mixture was quenched with saturated $NH_4Cl_{(aq)}$ solution. After workup, the crude product was purified *via* column chromatography (pentane:ethyl acetate = 9:1 to 8:2) to give **13r** (163 mg, 0.650 mmol, 65% yield) as a colorless oil.

^{1}H-NMR (400 MHz, $CDCl_3$): δ / ppm = 5.92 (t, *J* = 1.7 Hz, 1H), 3.37 (dd, *J* = 8.8, 6.4 Hz, 2H), 3.24 – 3.12 (m, 2H), 2.53 (td, *J* = 6.0, 1.8 Hz, 2H), 2.48 – 2.40 (m, 2H), 2.16 – 2.07 (m, 2H), 1.61 – 1.44 (m, 4H), 1.38 – 1.29 (m, 2H), 1.28 – 1.19 (m, 2H), 0.91 (dt, *J* = 14.2, 7.3 Hz, 6H).
^{13}C-NMR (101 MHz, $CDCl_3$): δ / ppm = 199.0, 169.6, 156.7, 126.0, 48.3, 44.0, 37.5, 31.0, 29.6, 27.4, 22.7, 20.3, 20.1, 14.0, 13.8.
IR (Diamond-ATR, neat): $\tilde{\nu}$ / cm-1 = 2957, 2931, 2872, 1675, 1630, 1616, 1456, 1427, 1377, 1345, 1321, 1290, 1248, 1224, 1188, 1079, 965, 890, 732.
MS (EI, 70 eV): *m/z* (%) = 208 (17), 194 (15), 182 (10), 180 (70), 166 (14), 166 (22), 156 (20), 153 (10), 152 (17), 138 (18), 123 (68), 111 (12), 95 (100), 67 (23), 67 (11), 55 (11).
HRMS (EI): *m/z* calc. for [$C_{15}H_{25}NO_2$]: 251.1885; found 251.1883.

(*Z*)-Methyl 4-(dibutylamino)-4-oxobut-2-enoate (13s)

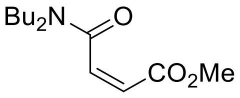

Following modified **TP5**, *N,N*-dibutylformamide (**4a**) (314 mg, 2.00 mmol, 2.0 equiv), $ZnCl_2$ (1.00 mL, 1.00 mmol, 1.0 equiv) and Et_3N (101 mg, 1.00 mmol, 1.0 equiv) were mixed in THF (4.0 mL) and a freshly prepared solution of TMPLi (2.20 mmol, 2.2 equiv) was dropwise added at 15 °C. After cooling to −78 °C, CuCN·2LiCl (1.00 mL, 1.00 mmol, 1.0 equiv) and (*Z*)-ethyl 3-iodoacrylate (226 mg, 1.00 mmol, 1.0 equiv). Thereafter, the reaction mixture was quenched with saturated $NH_4Cl_{(aq)}$ solution. After workup, the crude product was purified *via* column chromatography (pentane:ethyl acetate = 9:1 to 8:2) to give **13s** (171 mg, 0.670 mmol, 67% yield) as a colorless oil.

^{1}H-NMR (400 MHz, $CDCl_3$): δ / ppm = 6.53 (d, *J* = 12.0 Hz, 1H), 5.95 (dd, *J* = 12.0, 0.6 Hz, 1H), 4.18 (q, *J* = 7.1 Hz, 2H), 3.45 – 3.32 (m, 2H), 3.27 – 3.13 (m, 2H), 1.65 – 1.55 (m, 2H), 1.53 – 1.44 (m, 2H), 1.41 – 1.31 (m, 2H), 1.31 – 1.20 (m, 5H), 0.92 (dt, *J* = 15.8, 7.3 Hz, 6H).
^{13}C-NMR (101 MHz, $CDCl_3$): δ / ppm = 166.6, 164.9, 138.2, 123.0, 60.9, 48.2, 44.6, 30.8, 29.5, 20.4, 20.1, 14.0, 13.9.
IR (Diamond-ATR, neat): $\tilde{\nu}$ / cm-1 = 2959, 2932, 2873, 1724, 1628, 1457, 1430, 1379, 1296, 1209, 1176, 1143, 1031, 951, 819, 756.
MS (EI, 70 eV): *m/z* (%) = 226 (23), 210 (11), 128 (40), 127 (17), 99 (100).
HRMS (EI): *m/z* calc. for [$C_{14}H_{25}NO_3$]: 255.1834; found 256.1908 (M+H).

NMR spectra of starting materials

_N_-(2-Hydroxyethyl)-_N_-methylformamide (INT-4b1)

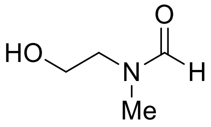

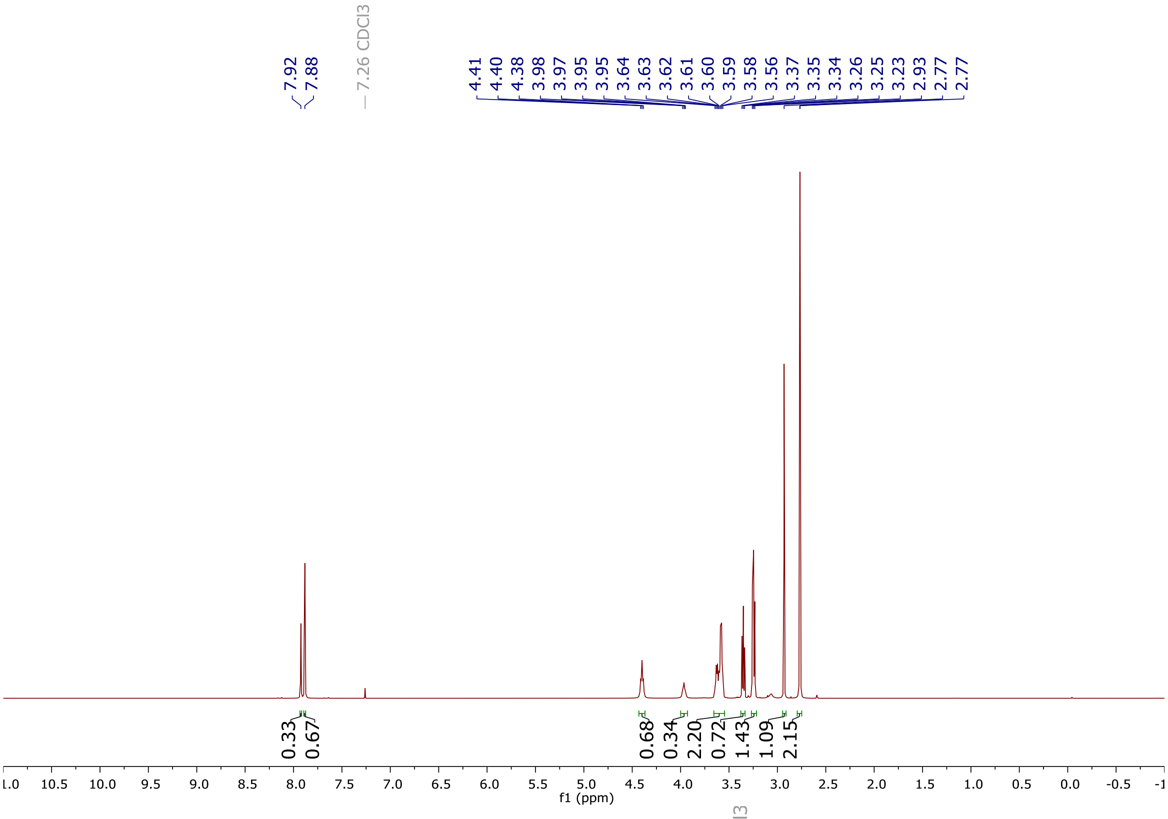

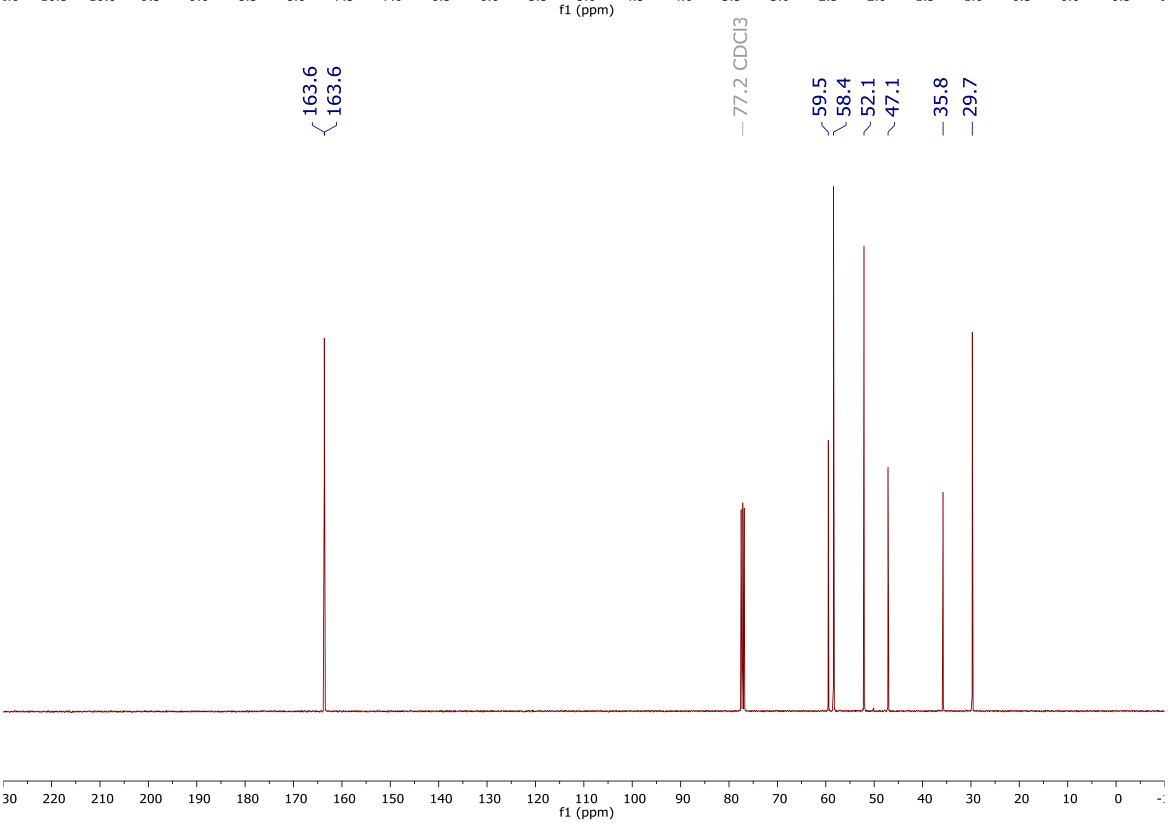

***N*-(2-((tert-Butyldimethylsilyl)oxy)ethyl)-*N*-methylformamide (4b)**

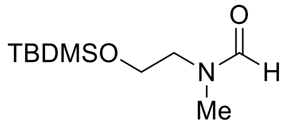

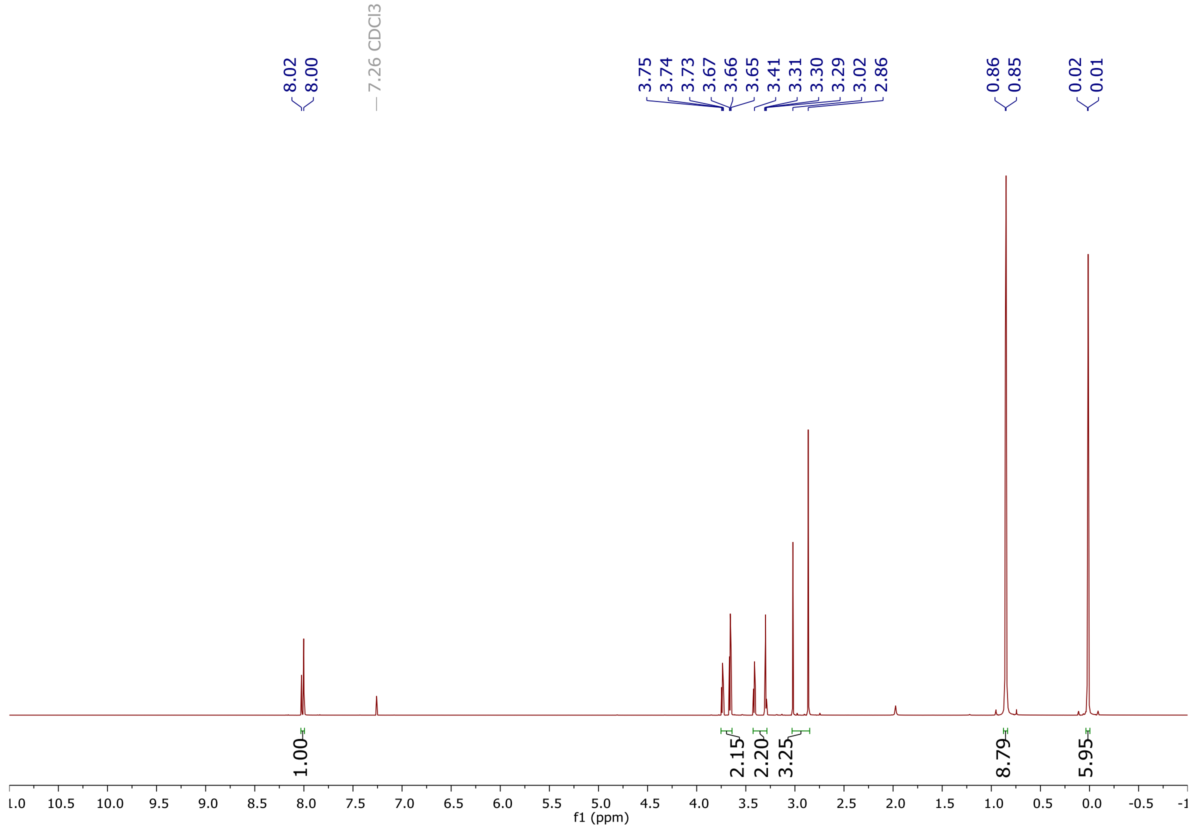

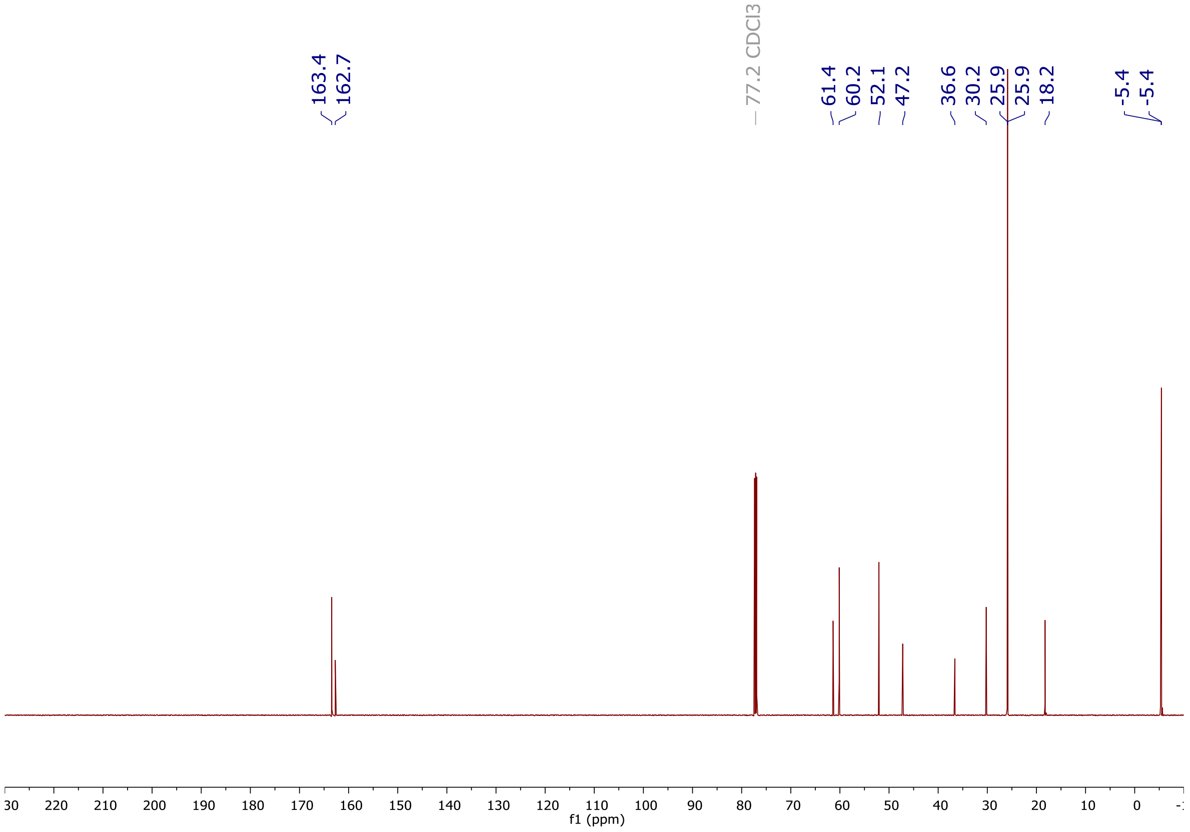

***trans*-Octahydroisoquinoline-2(1H)-carbaldehyde (4e)**

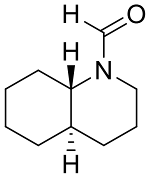

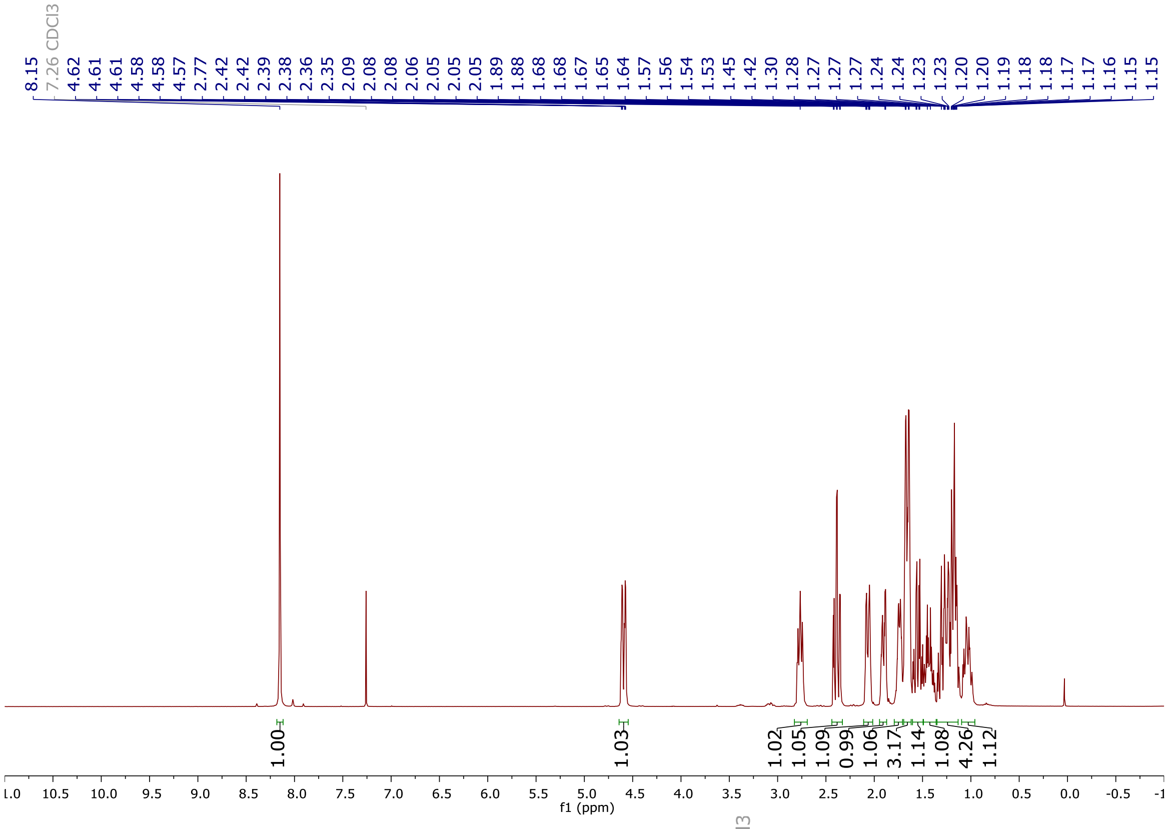

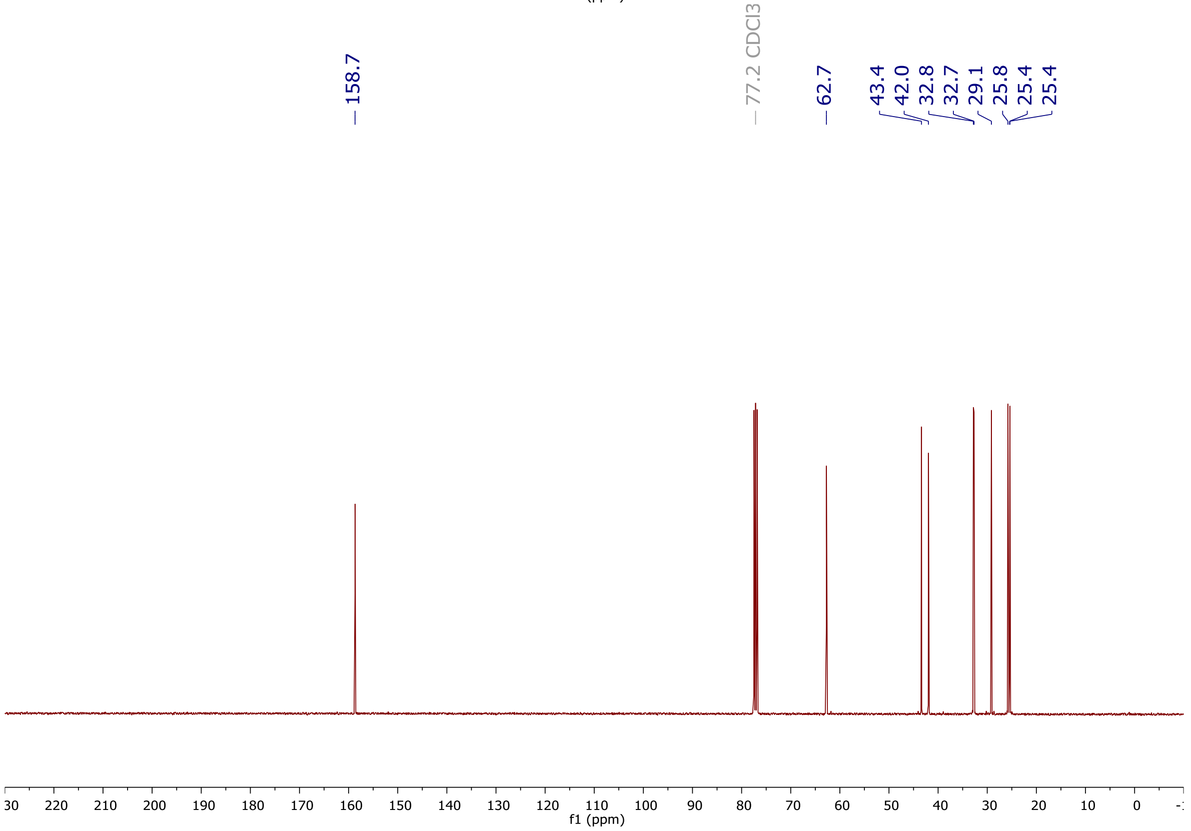

4-(3-(Trifluoromethyl)pyridin-2-yl)piperazine-1-carbaldehyde (4f)

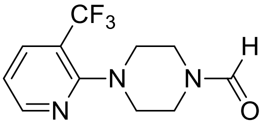

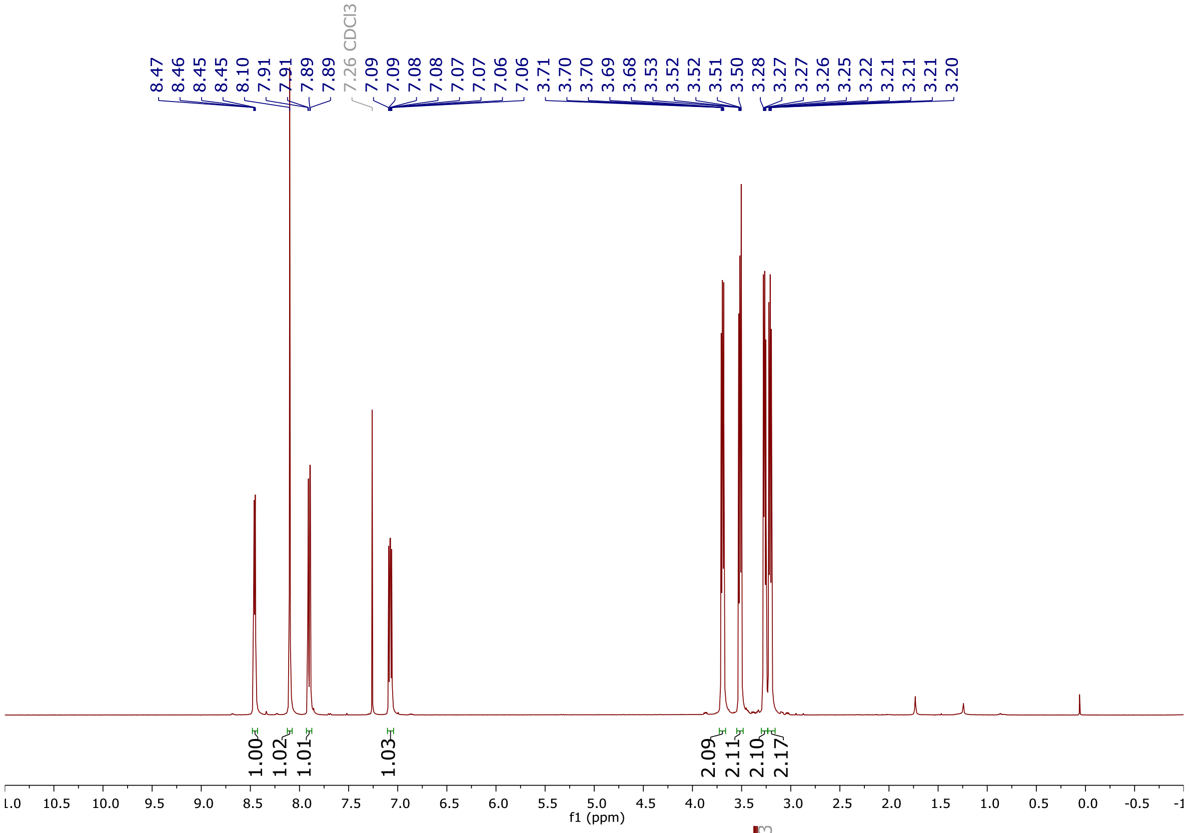

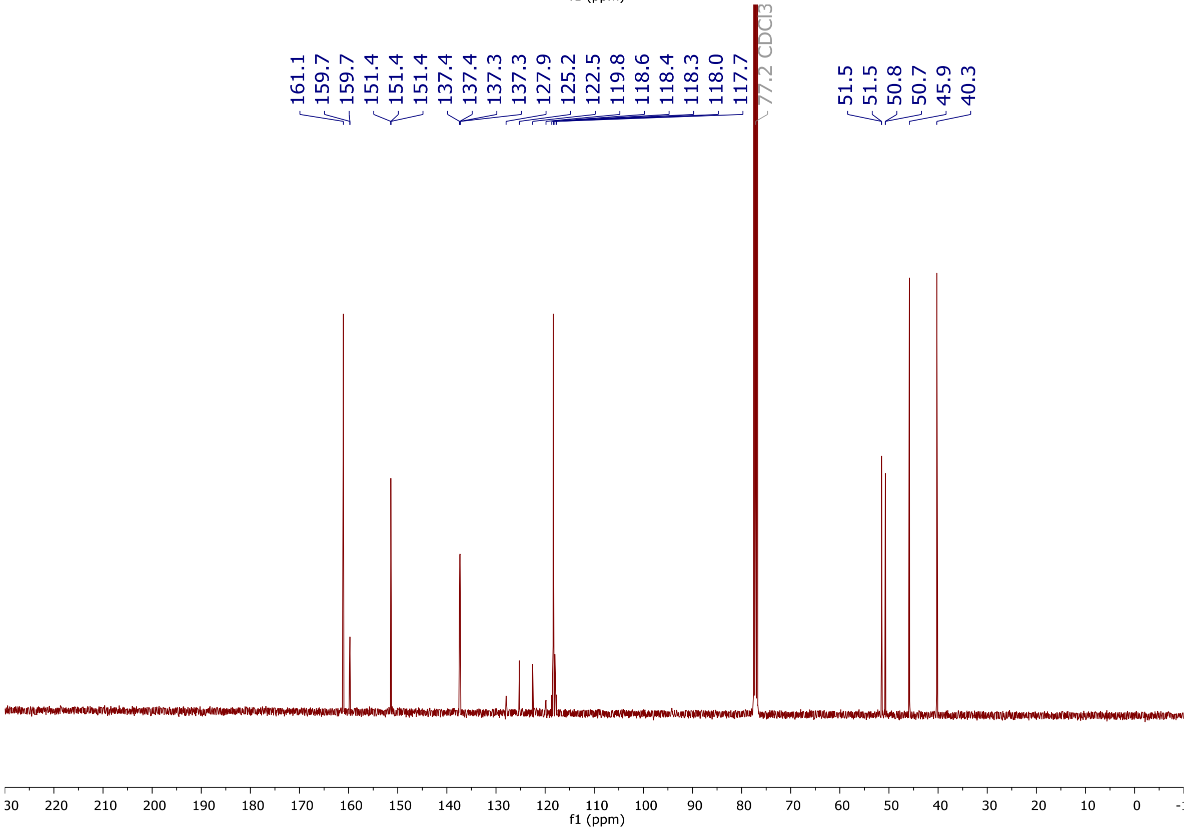

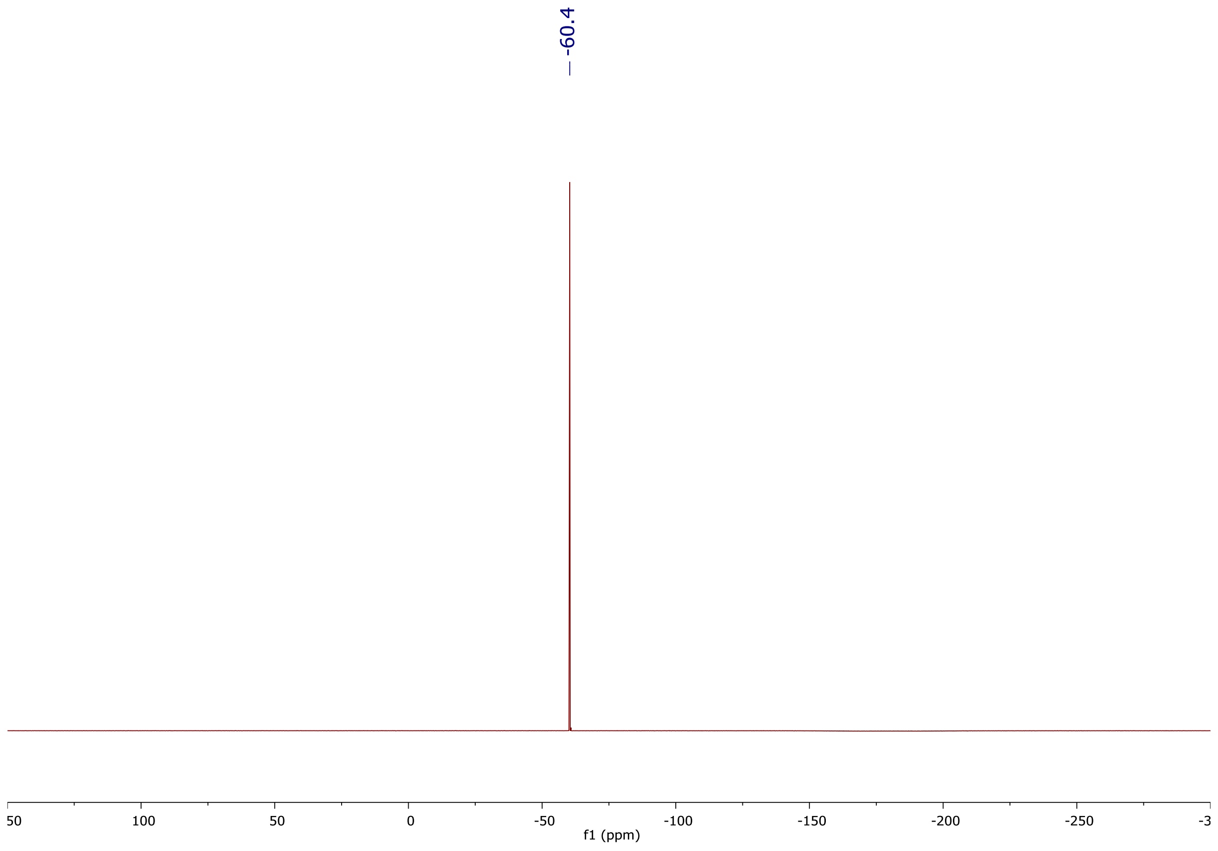
-60.4
50
100
50
0
-50
-100
-150
-200
-250
-3
f1 (ppm)

7,7,9,9-Tetramethyl-1,4-dioxa-8-azaspiro[4.5]decane-8-carbaldehyde (4h)

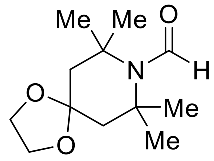

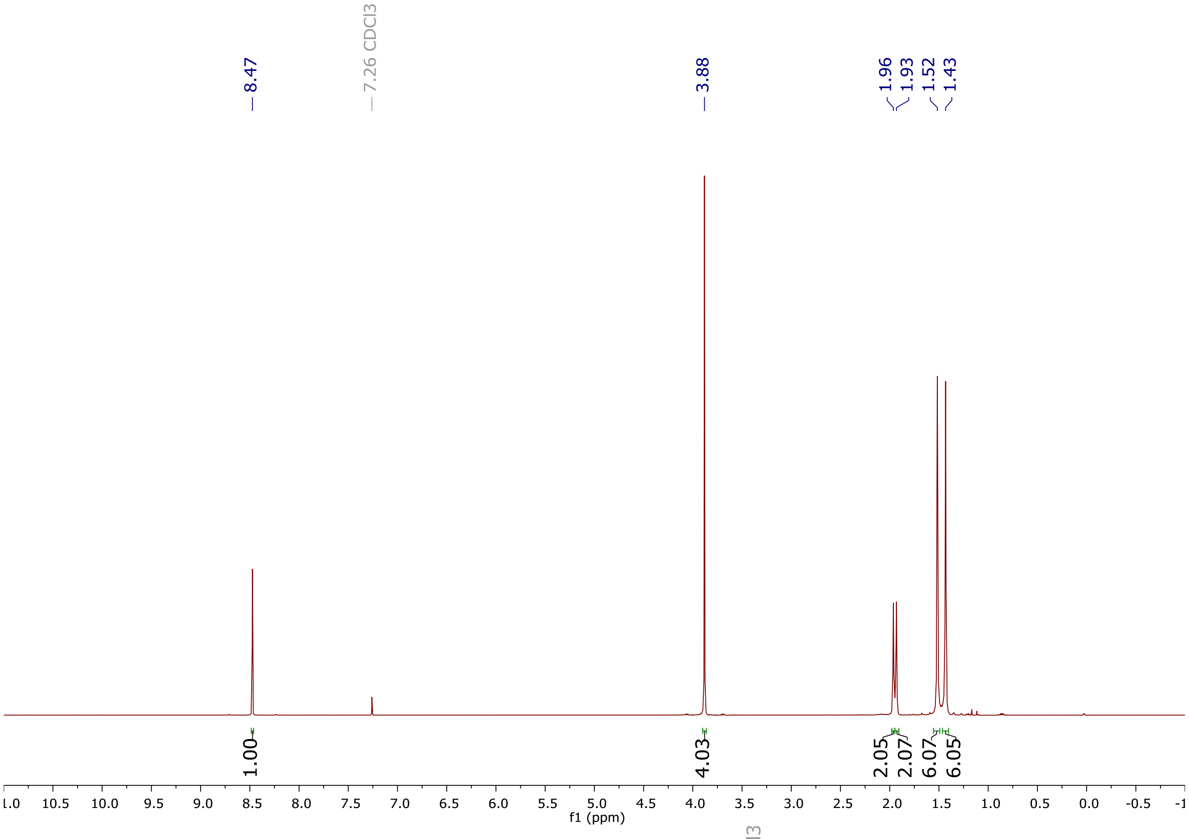

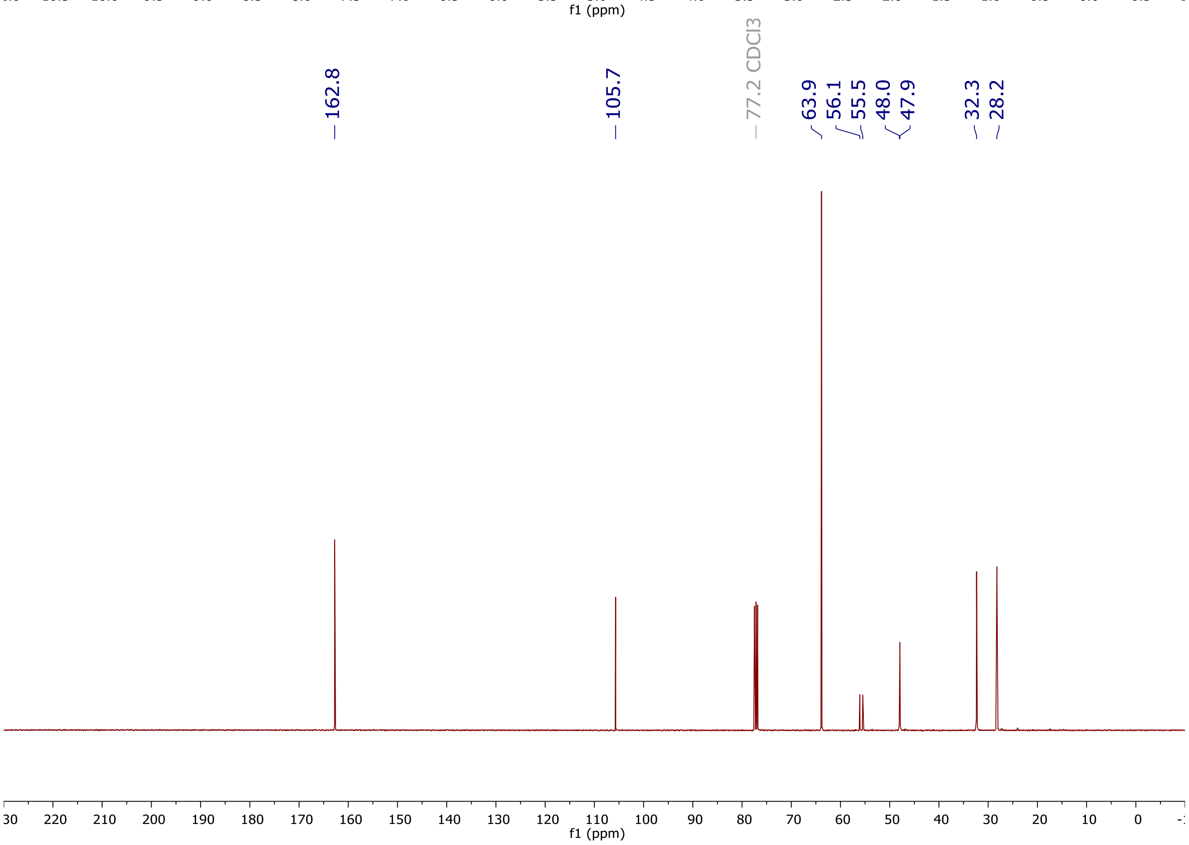

***N,N*-Dibutyl[^{13}C]formamide (4j)**

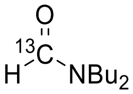

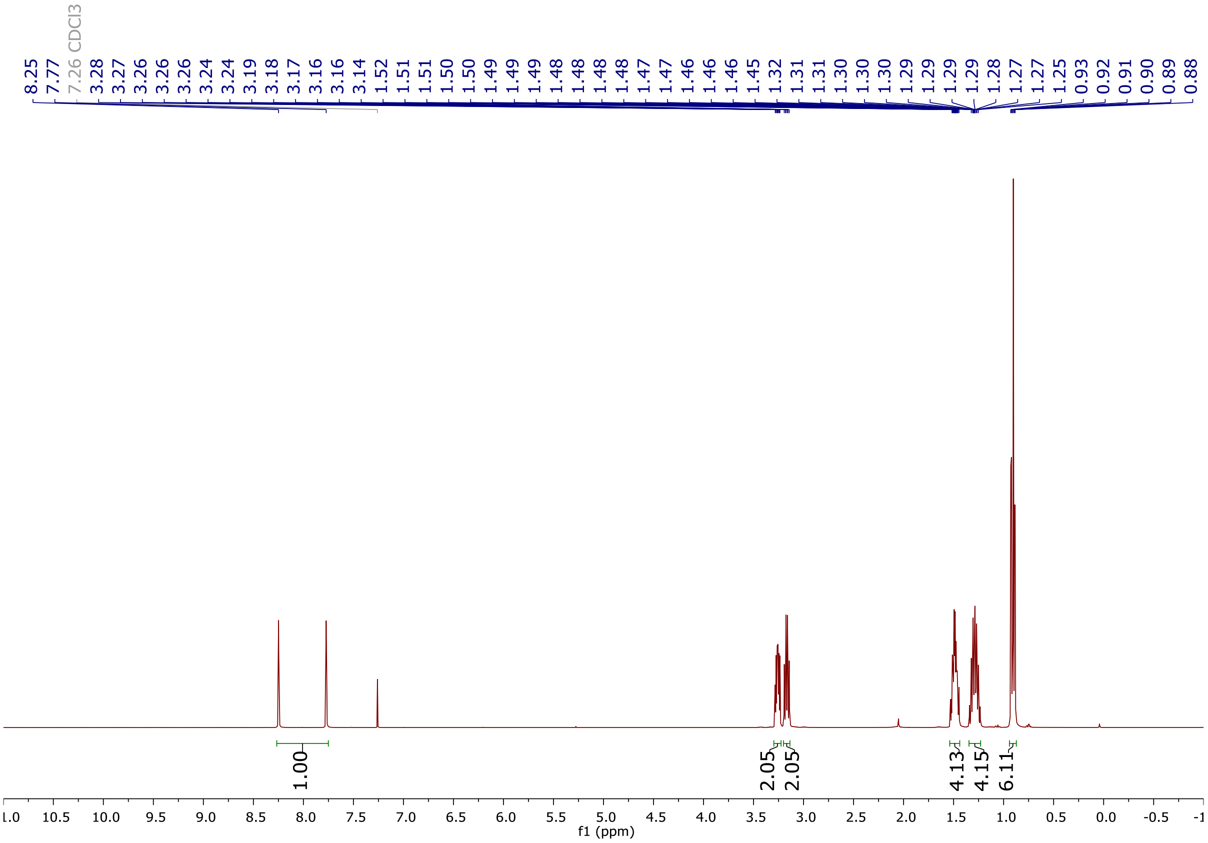

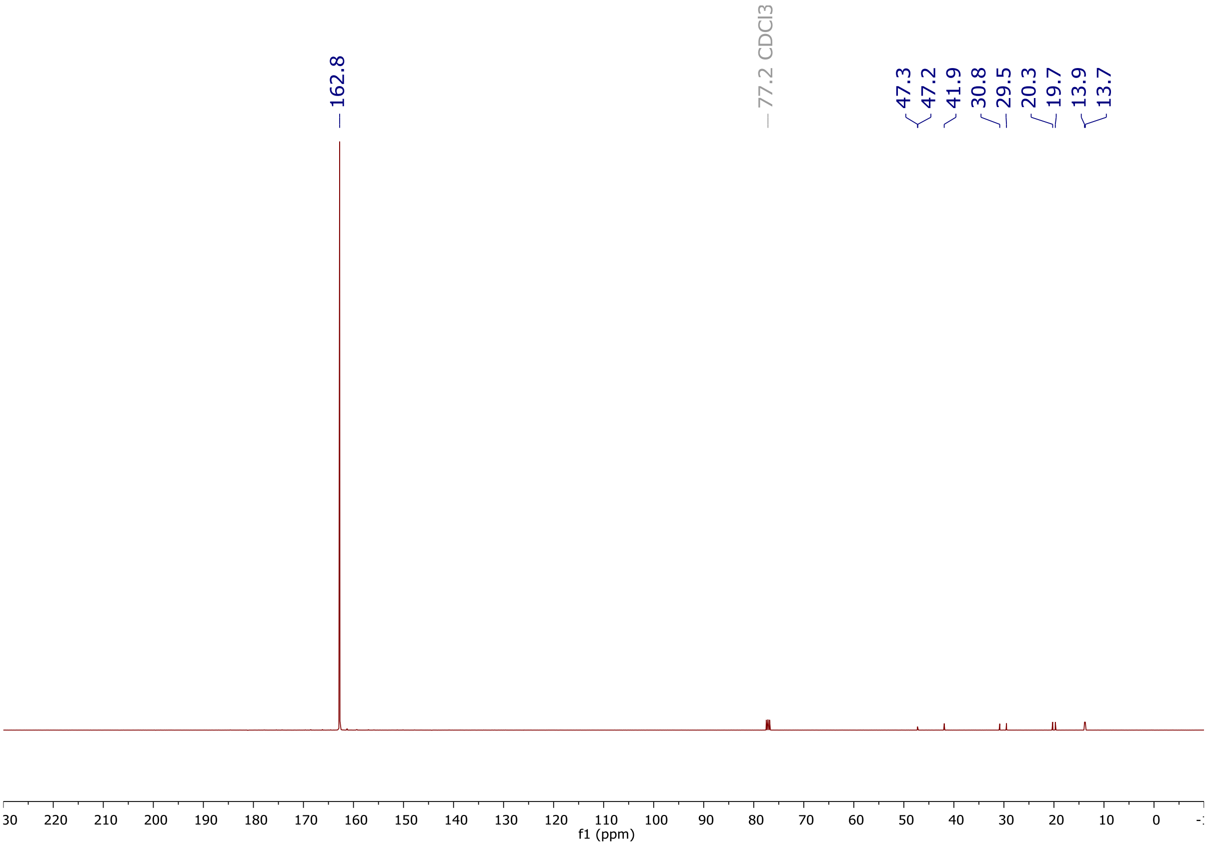

***N*-(2-Bromoethyl)-*N*-methylformamide (INT-4k1)**

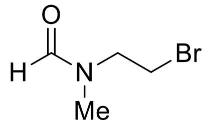

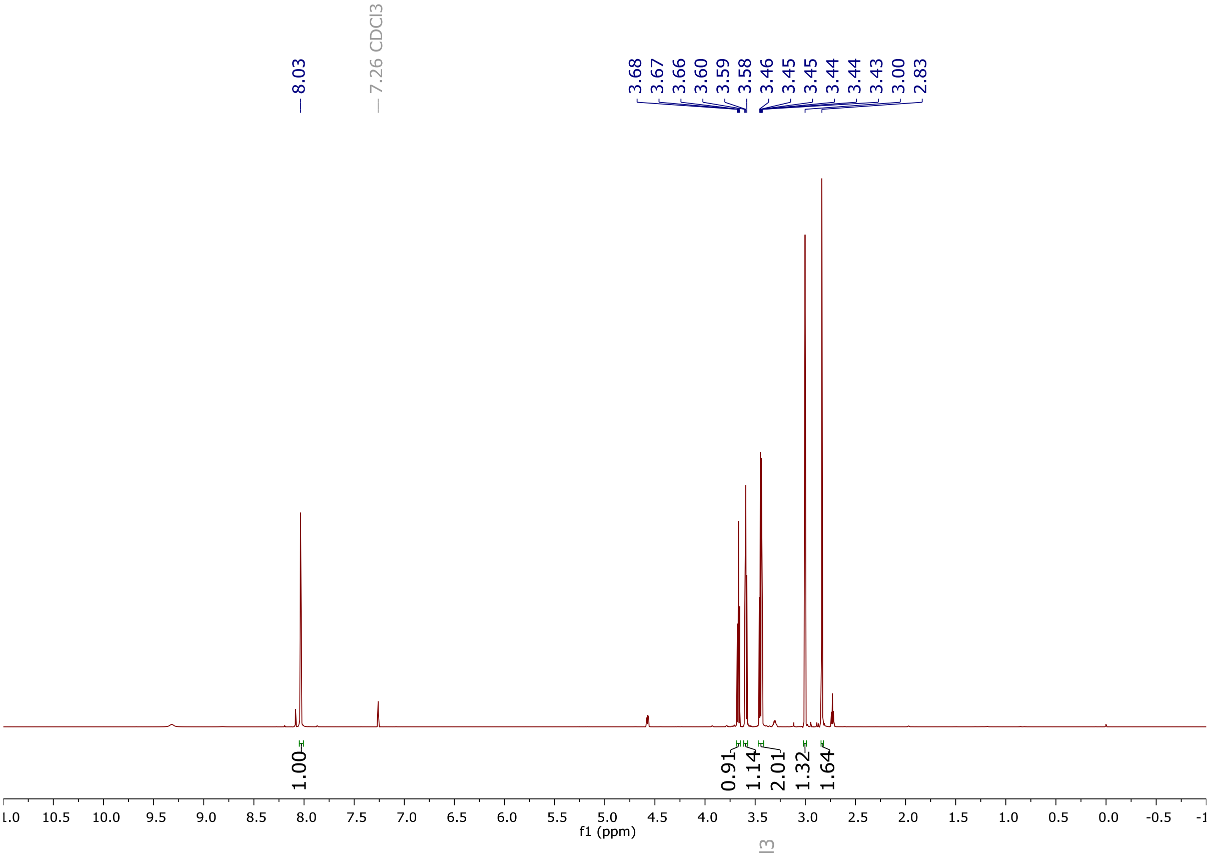

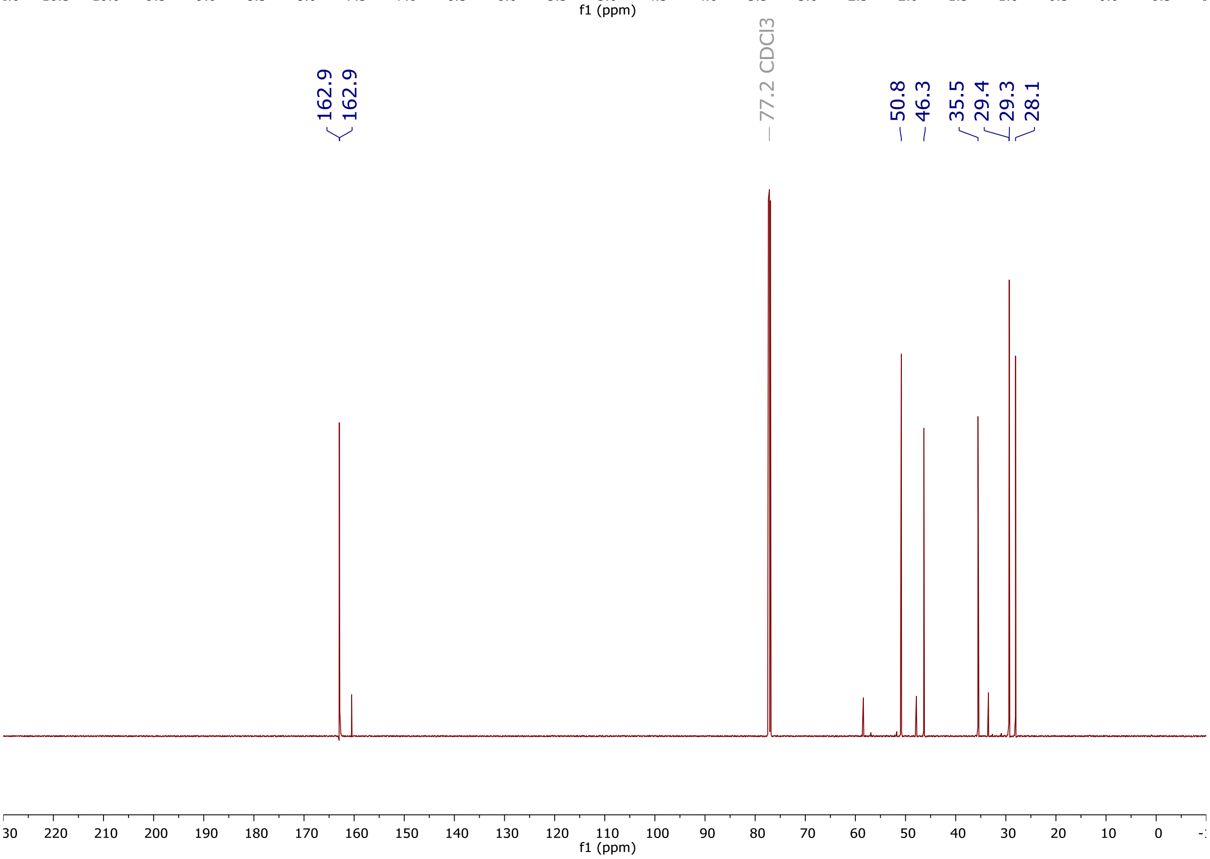

Ethyl 4-(2-(*N*-methylformamido)ethoxy)benzoate (4k)

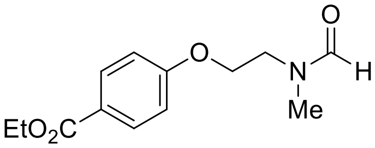

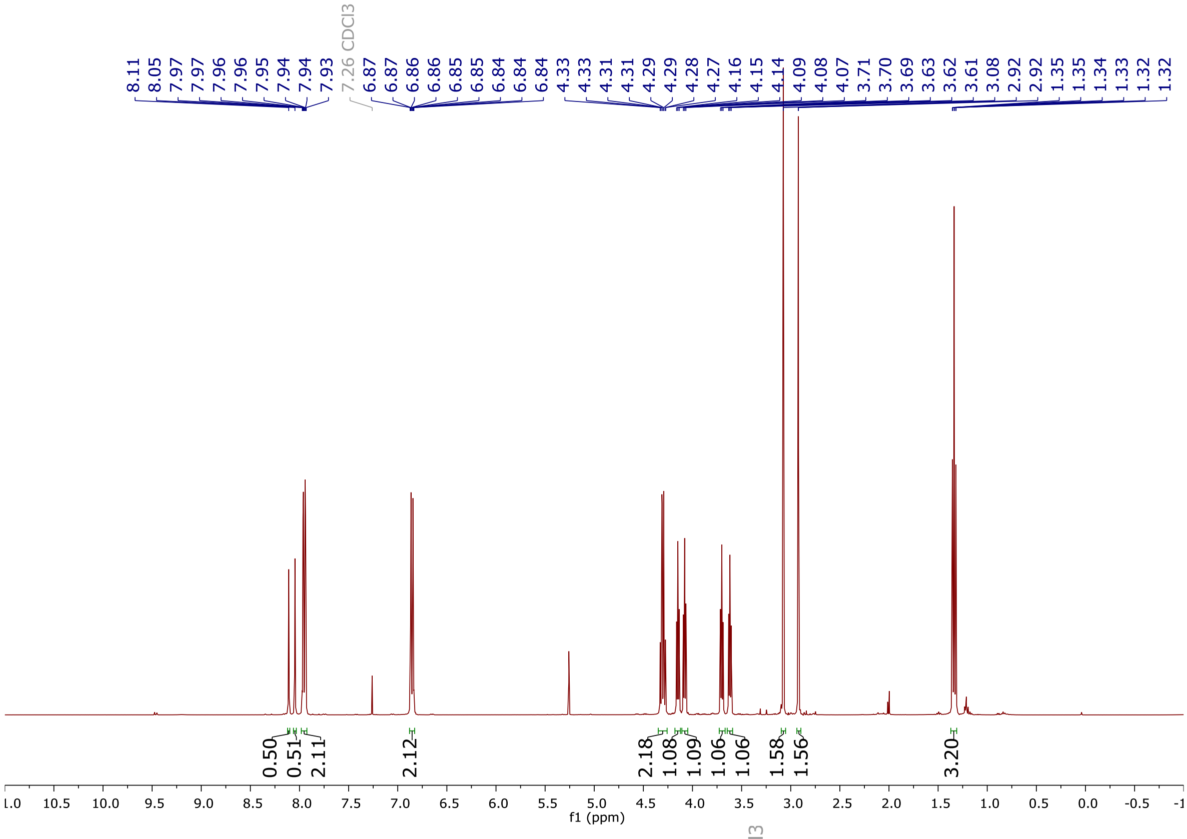

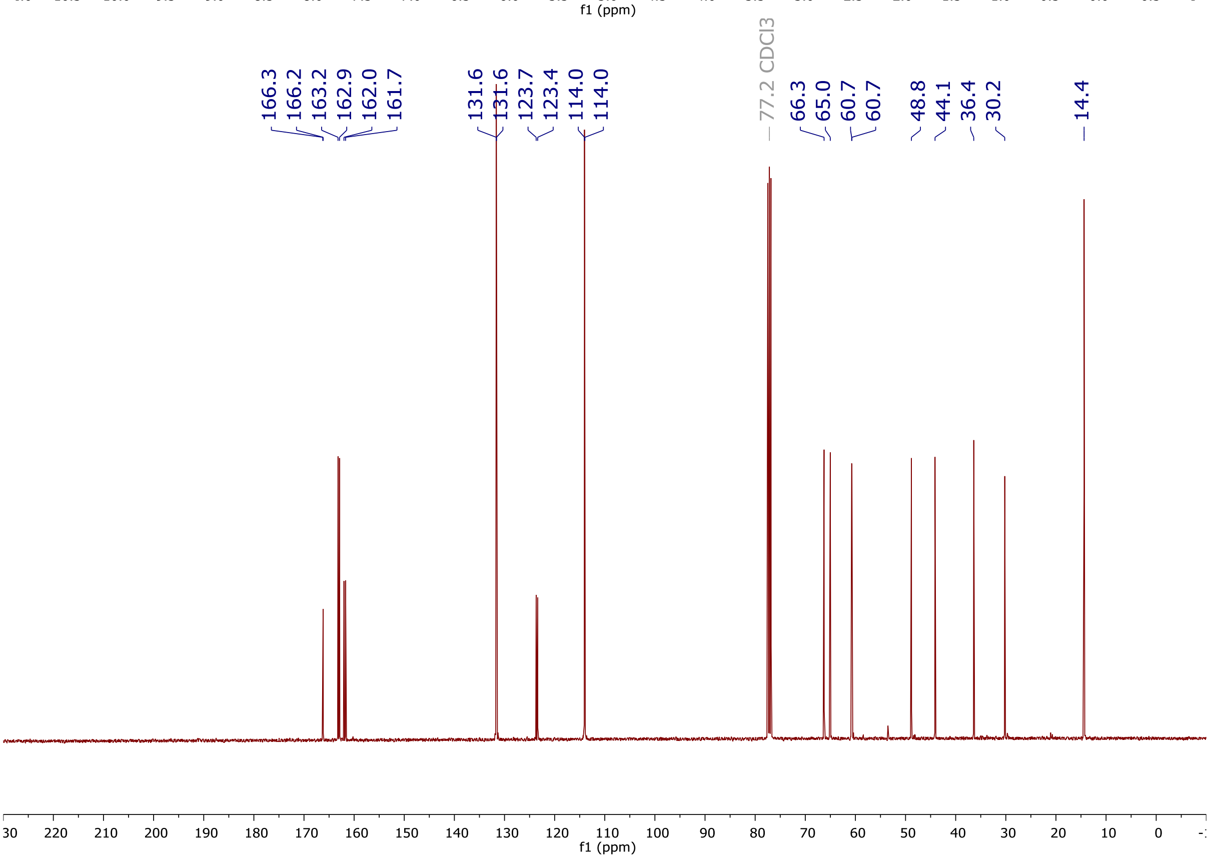

N-(2-Chloroethyl)-N-methylformamide (INT4l-1)

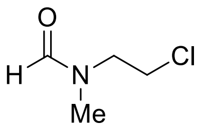

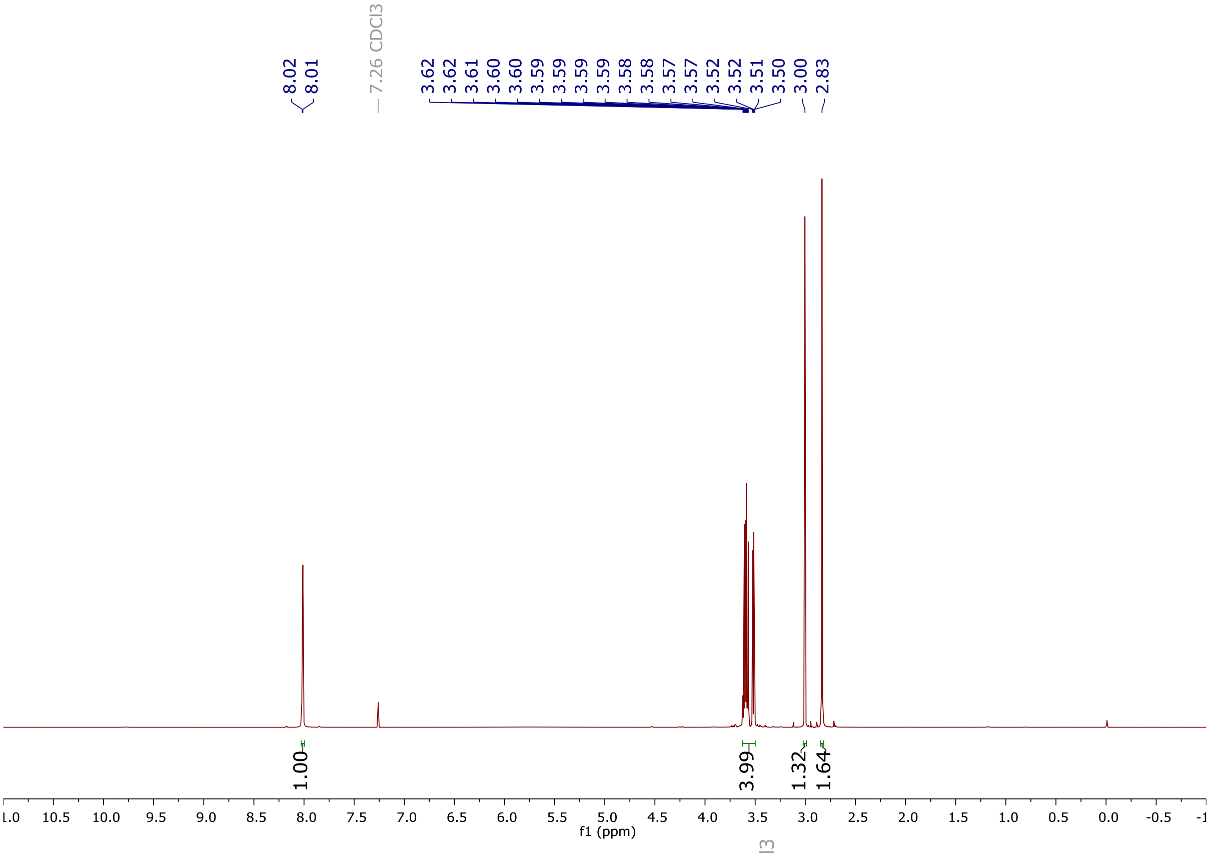

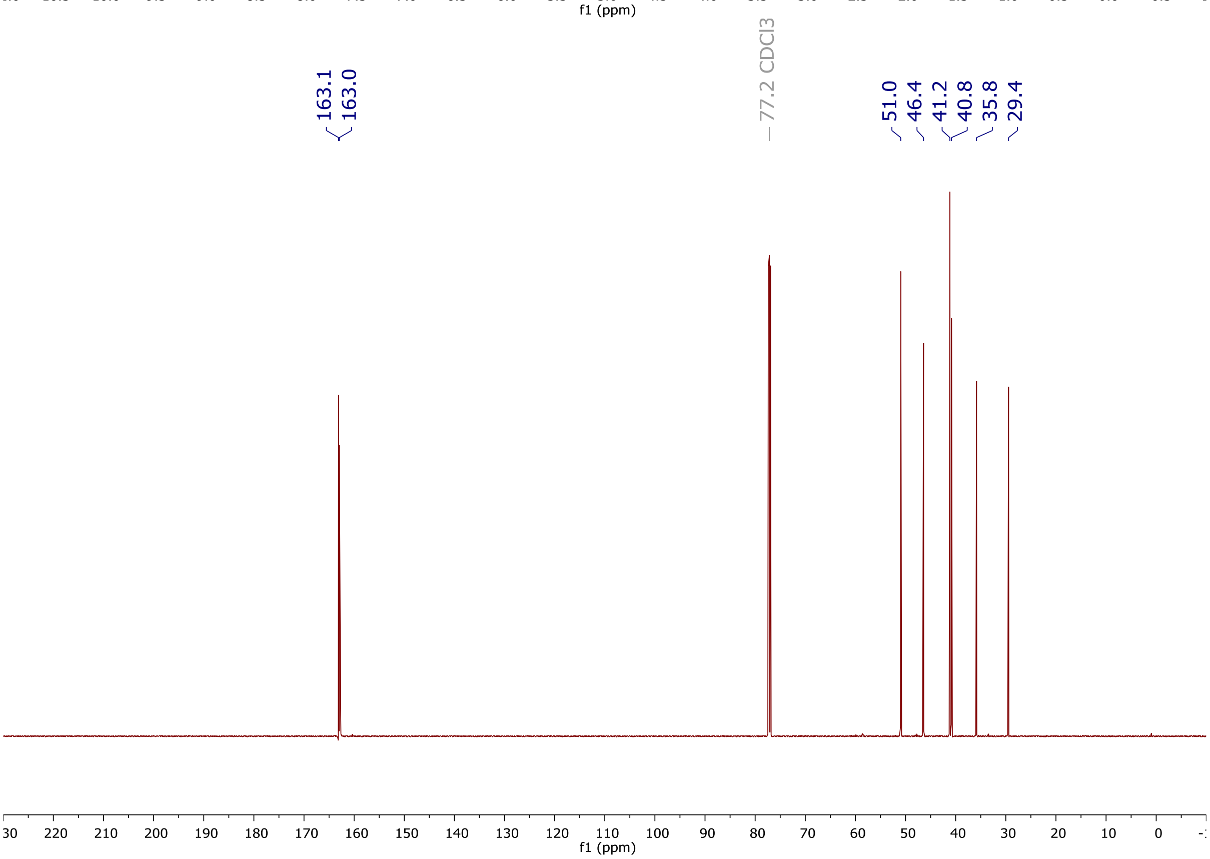

***N*-(2-(2-Benzoylphenoxy)ethyl)-*N*-methylformamide (4l)**

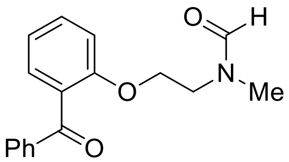

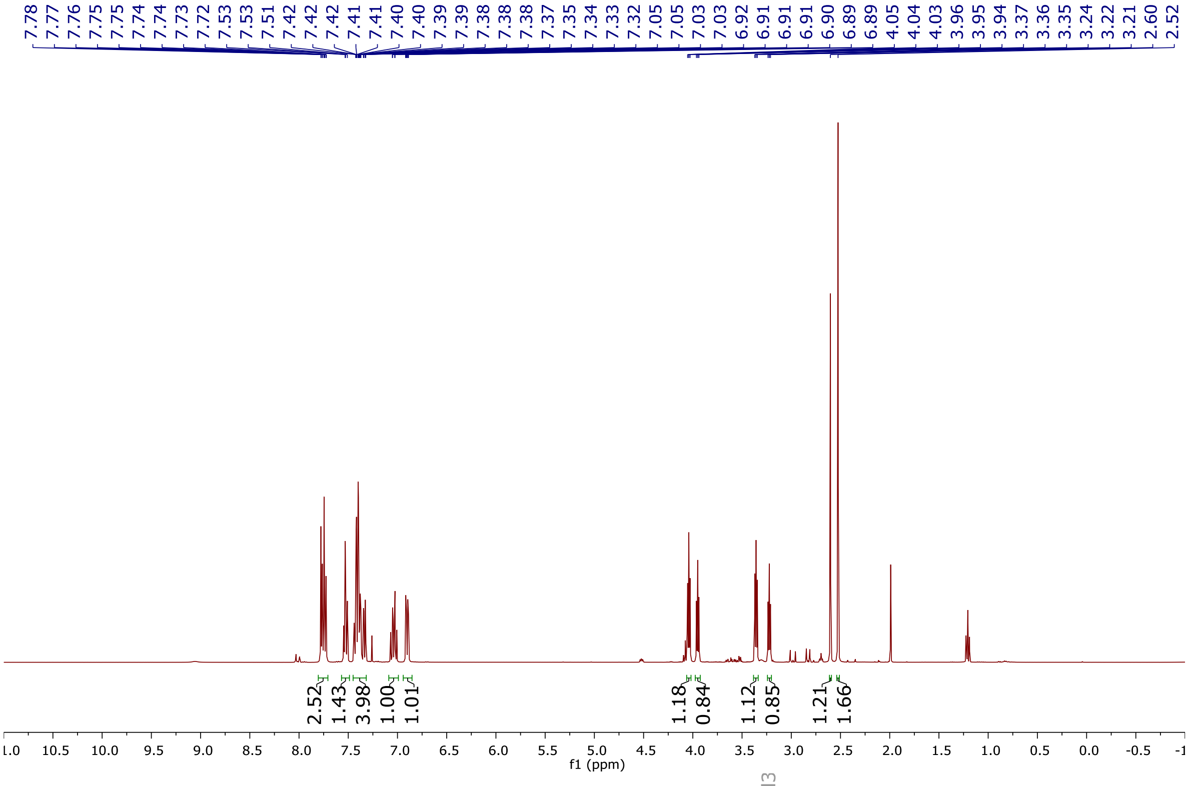

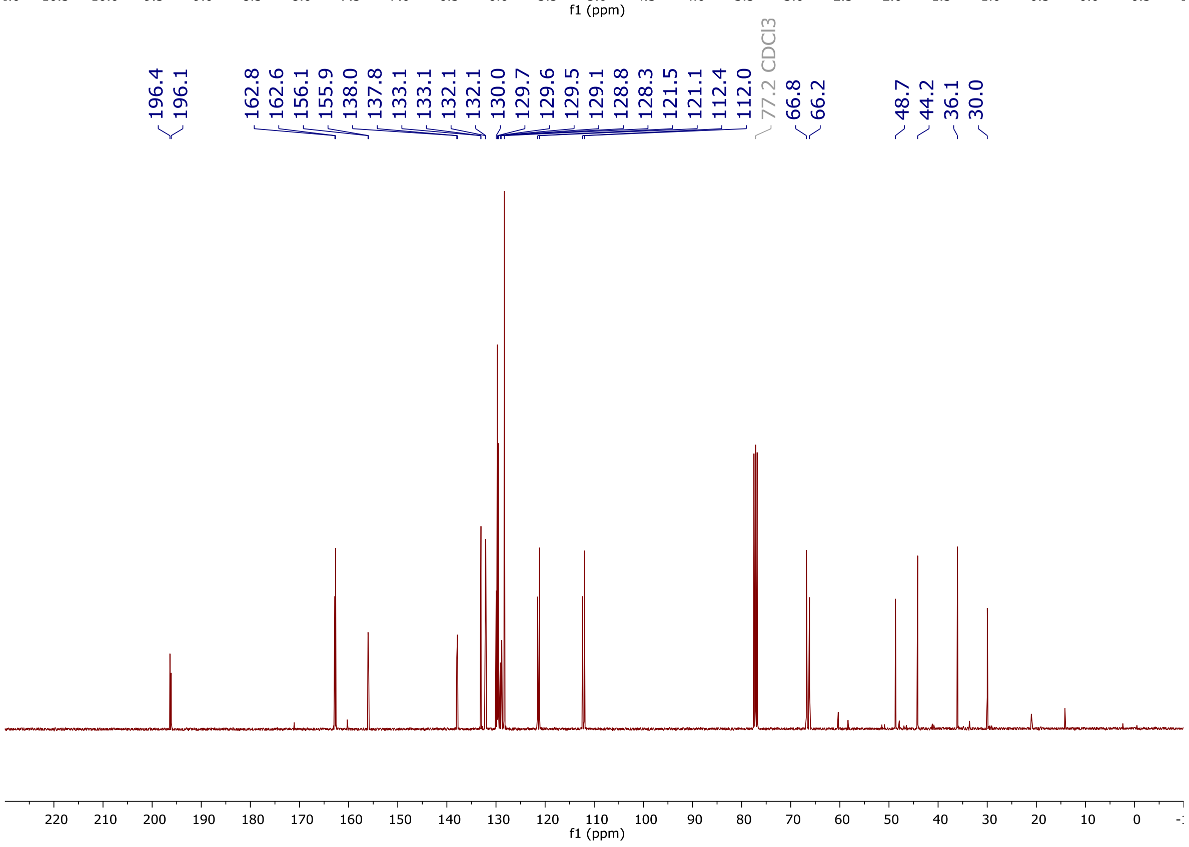

_N_-(3-(1,3-Dioxoisoindolin-2-yl)propyl)-_N_-isopropylformamide (4m)

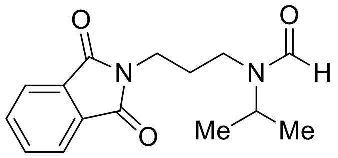

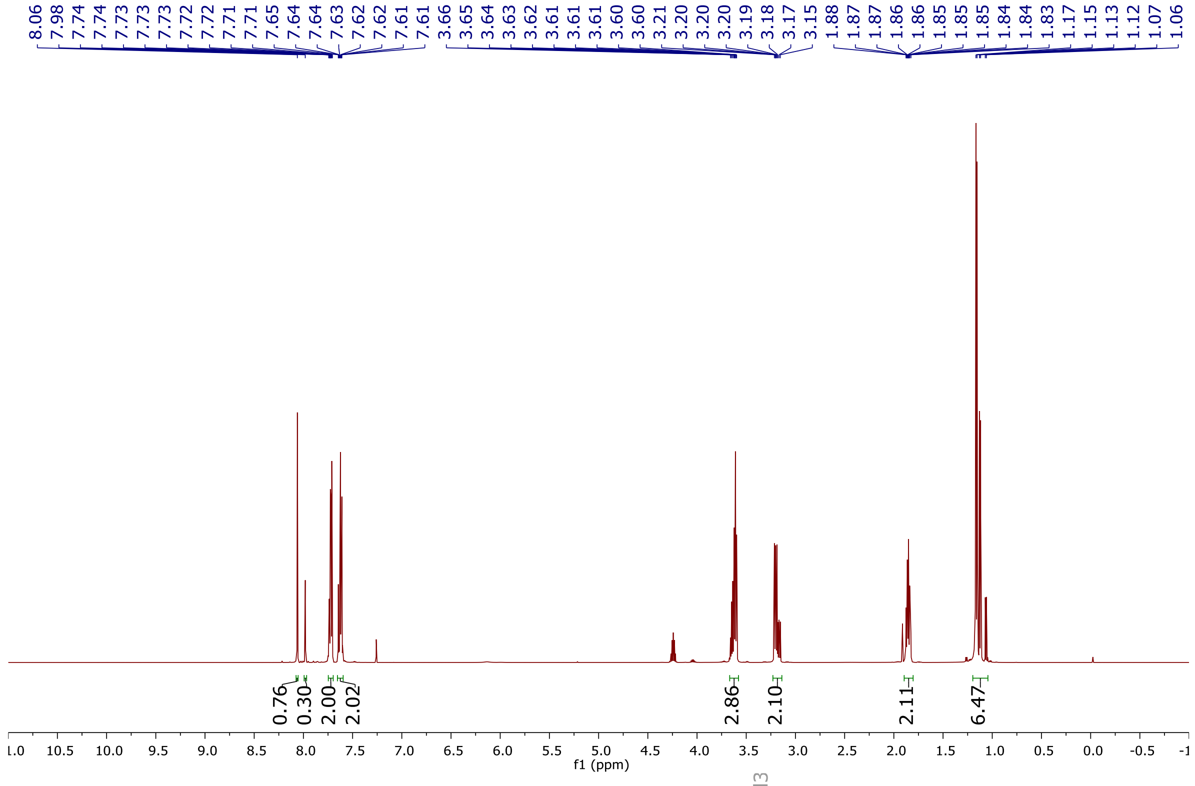

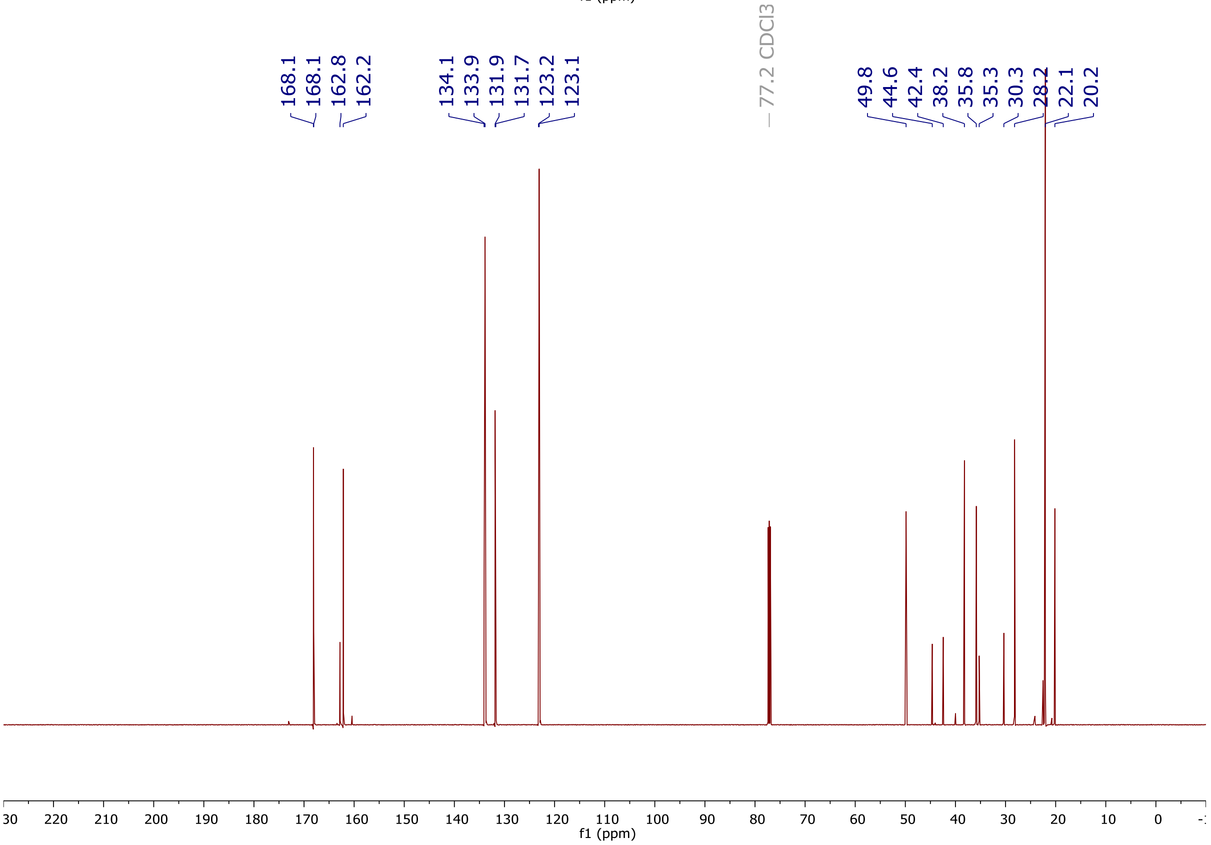

_N_-Methyl-_N_-(2-(4-nitrophenoxy)ethyl)formamide (4n)

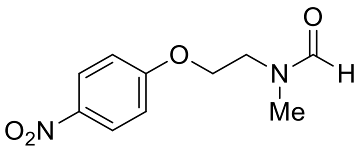

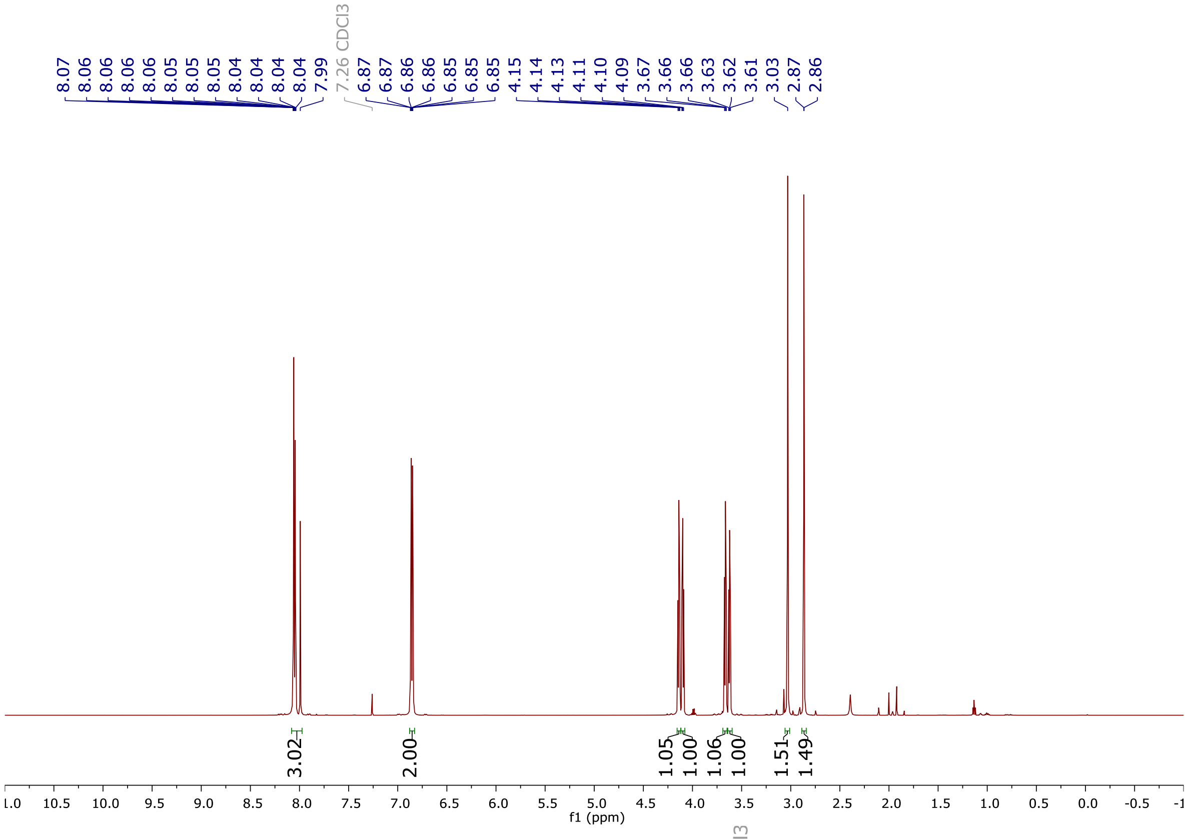

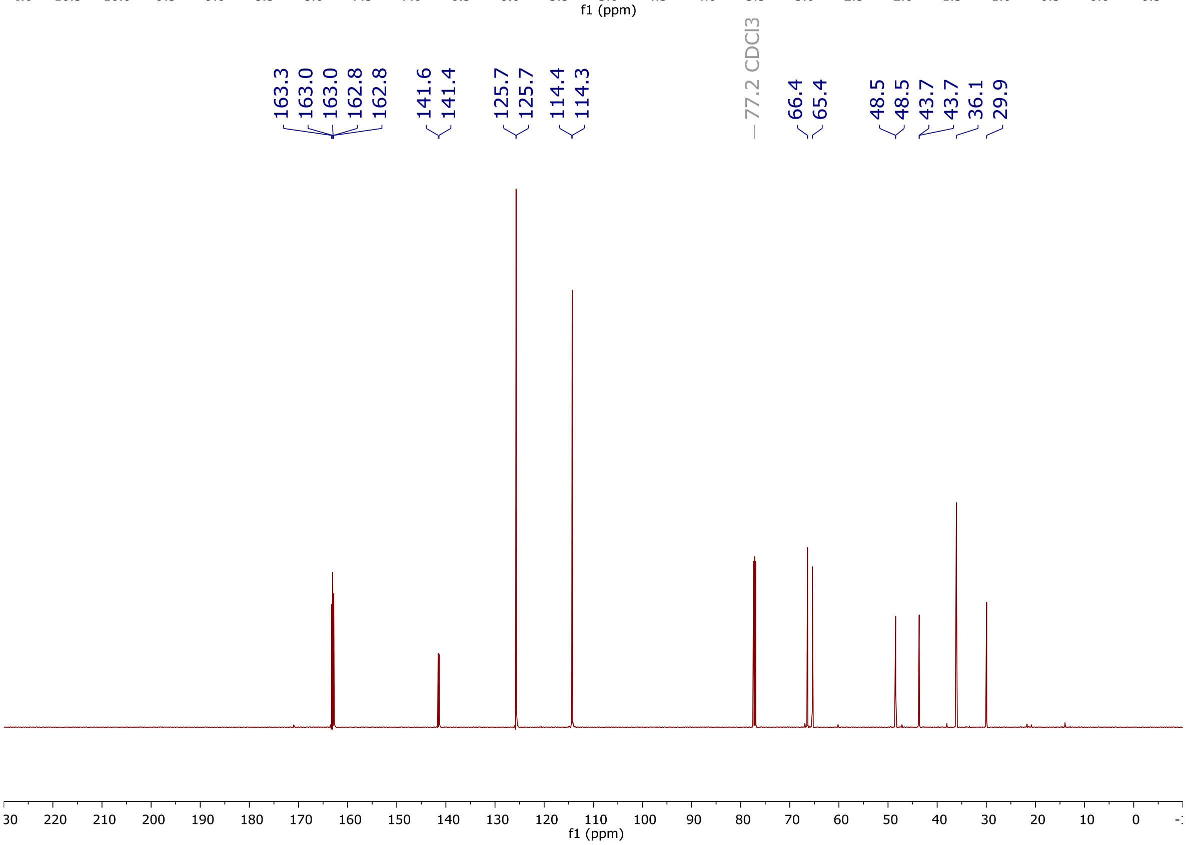

4-(4-Formylpiperazin-1-yl)benzonitrile (4p)

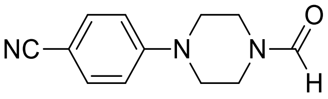

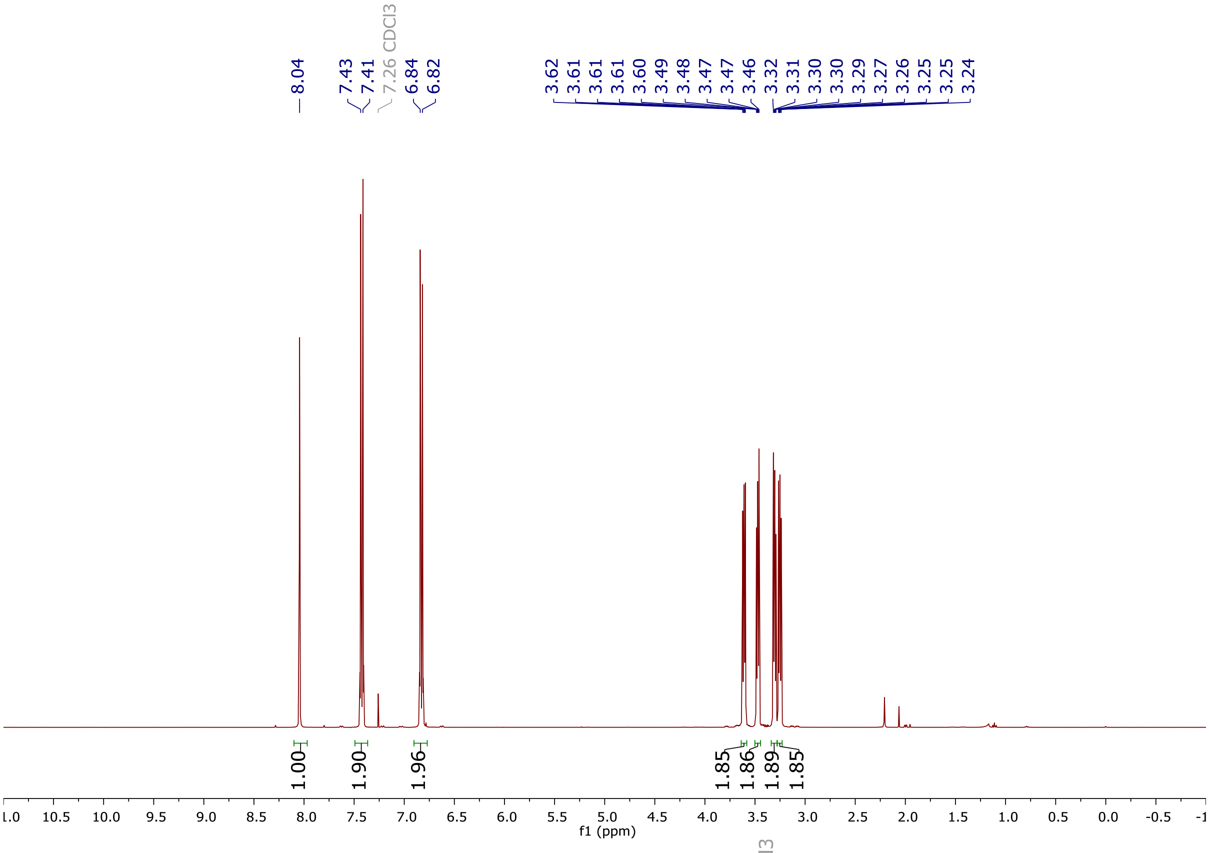

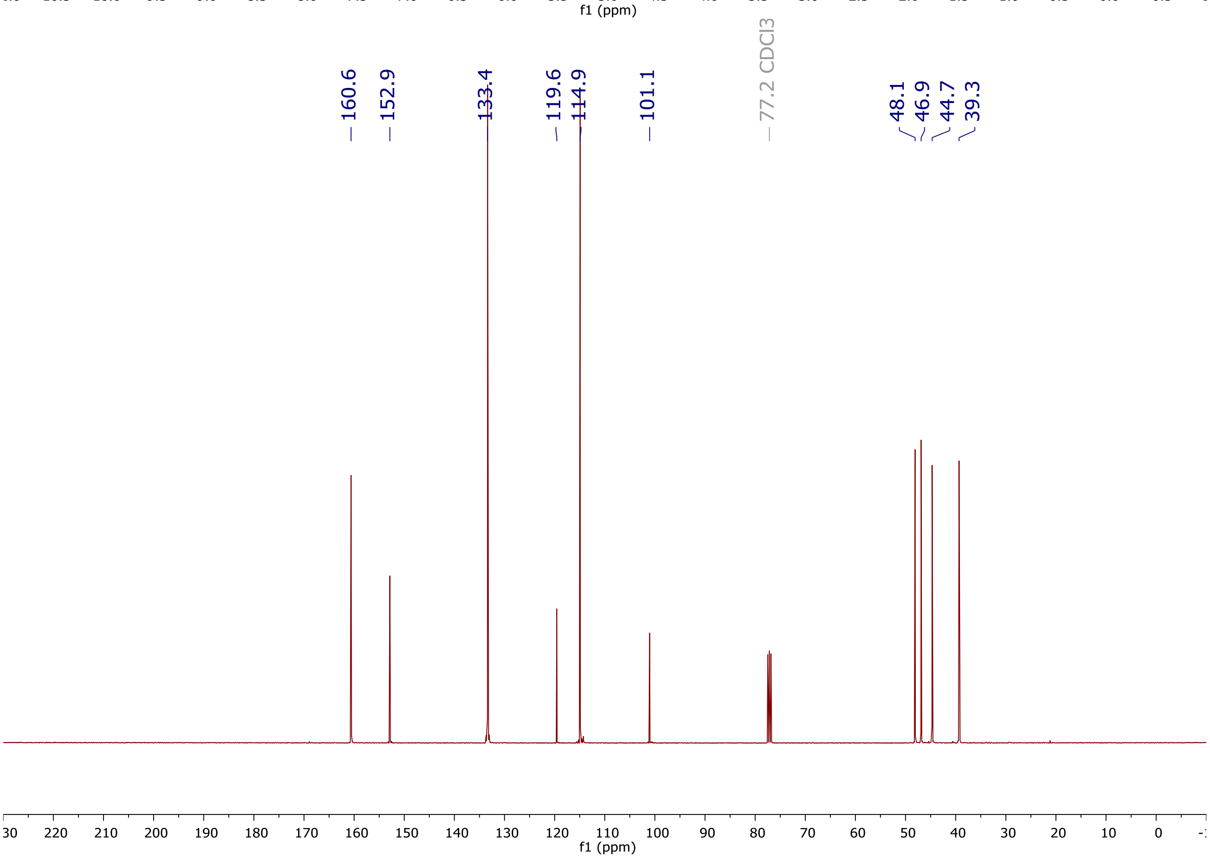

(1*R*,3*r*,5*S*)-3-Hydroxy-8-azabicyclo[3.2.1]octane-8-carbaldehyde (INT-4q1)

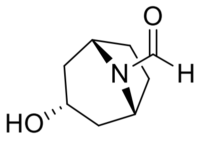

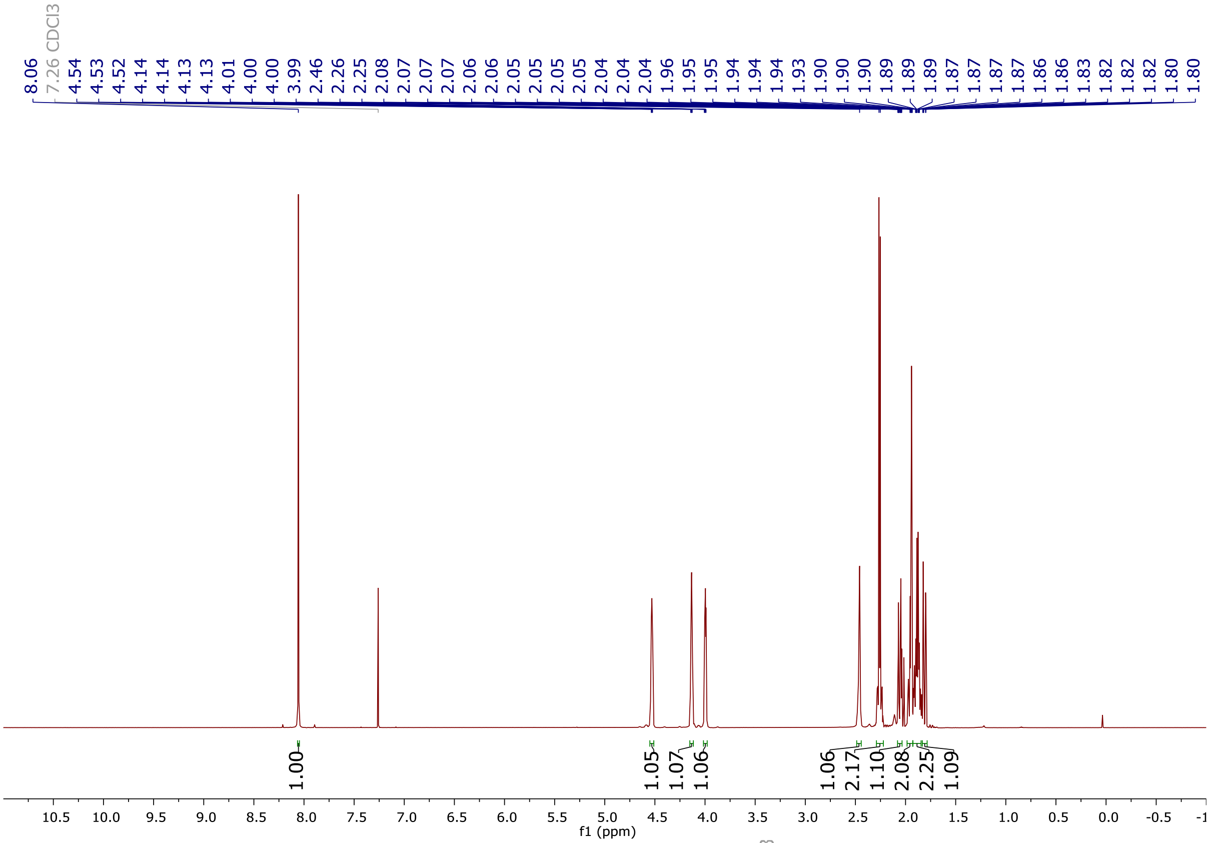

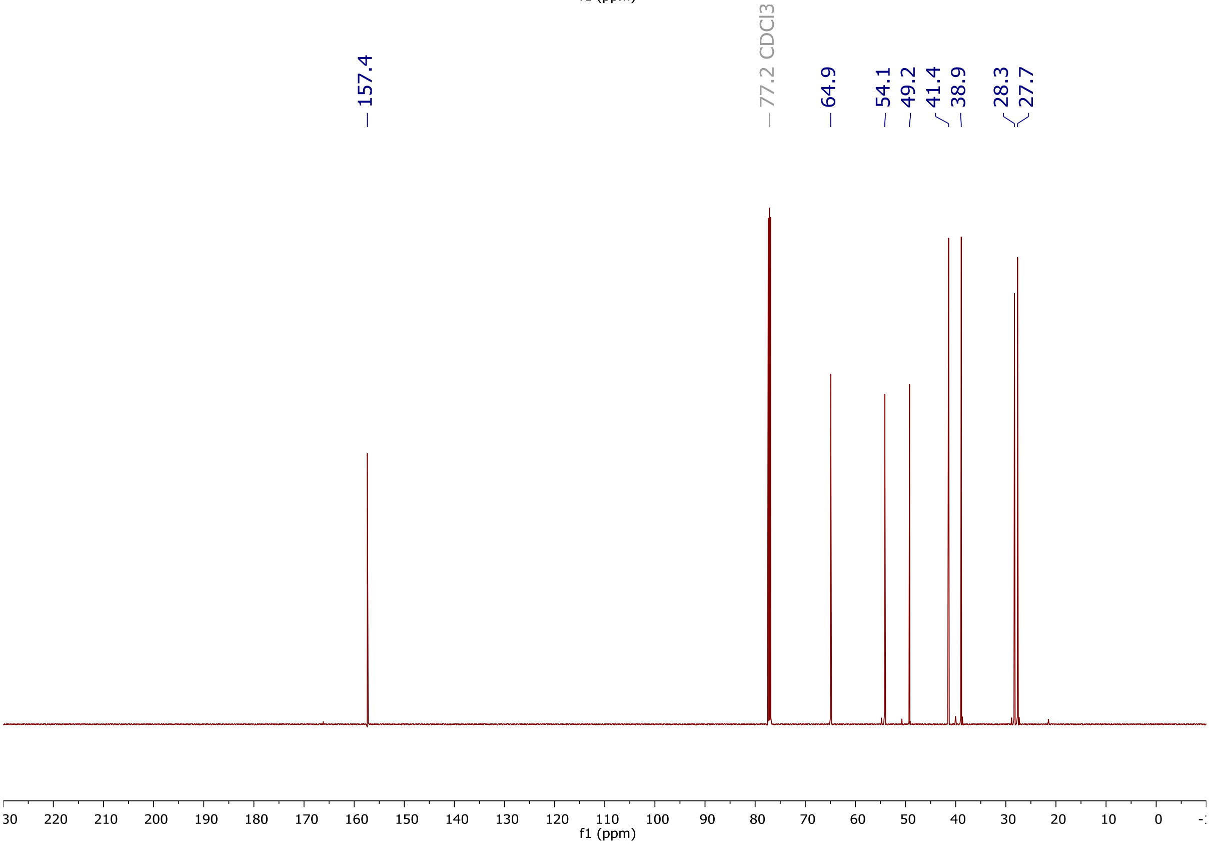

(*1R,3r,5S*)-3-((Triethylsilyl)oxy)-8-azabicyclo[3.2.1]octane-8-carbaldehyde (4q)

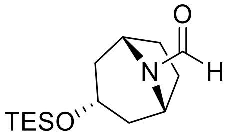

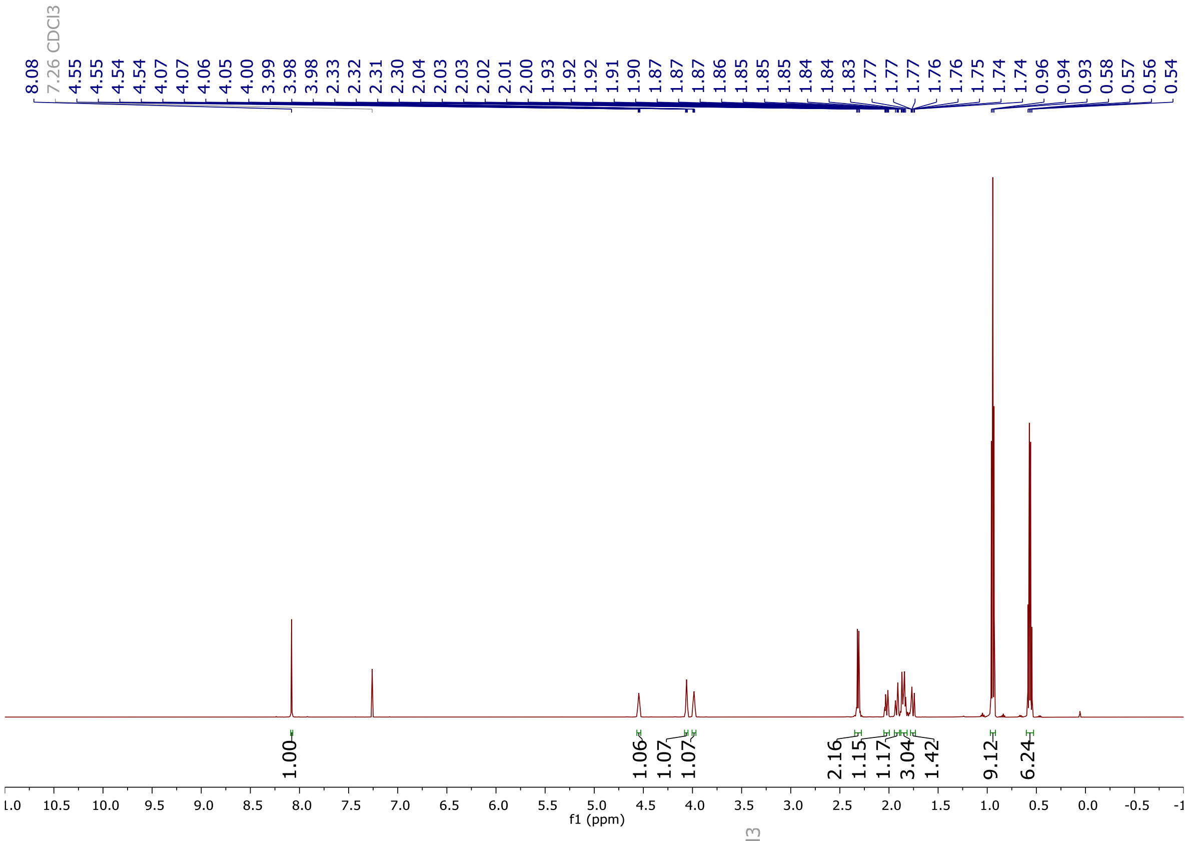

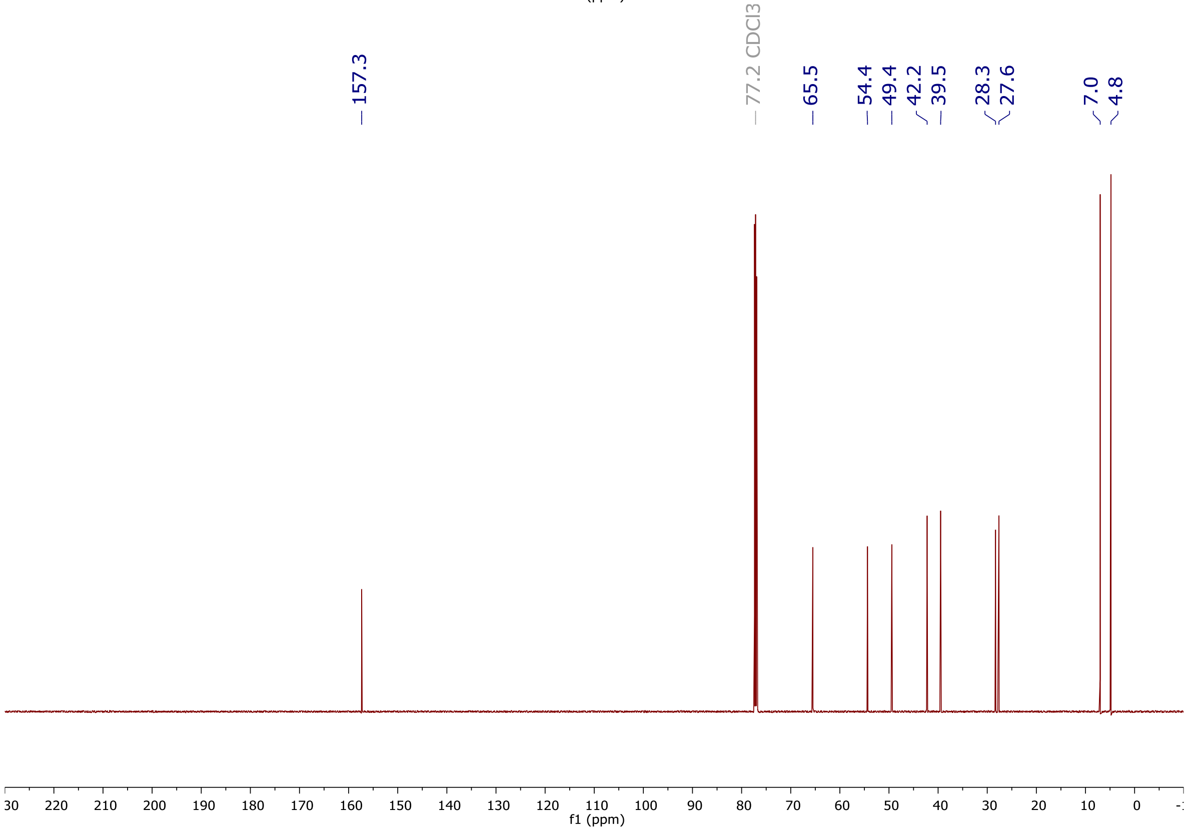

NMR spectra of products

***N,N*-Dibutylbut-3-enamide (8a)**

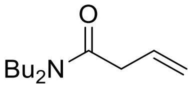

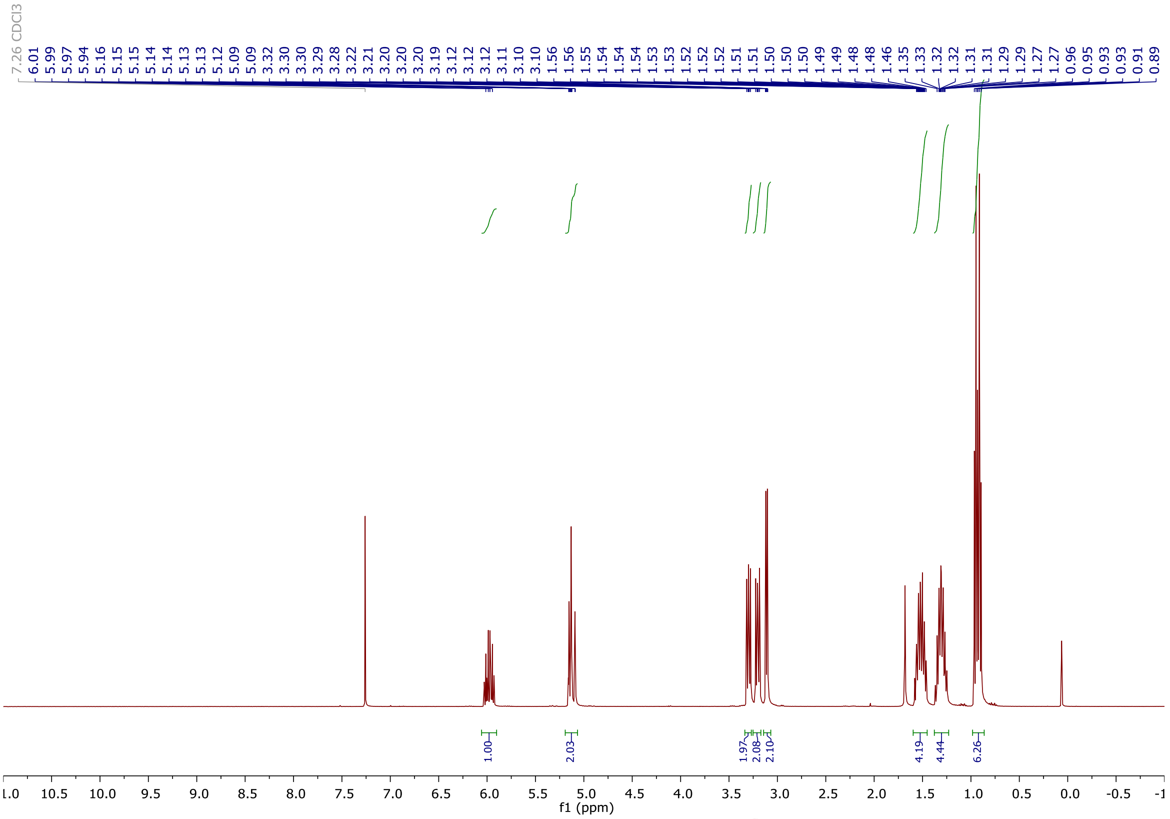

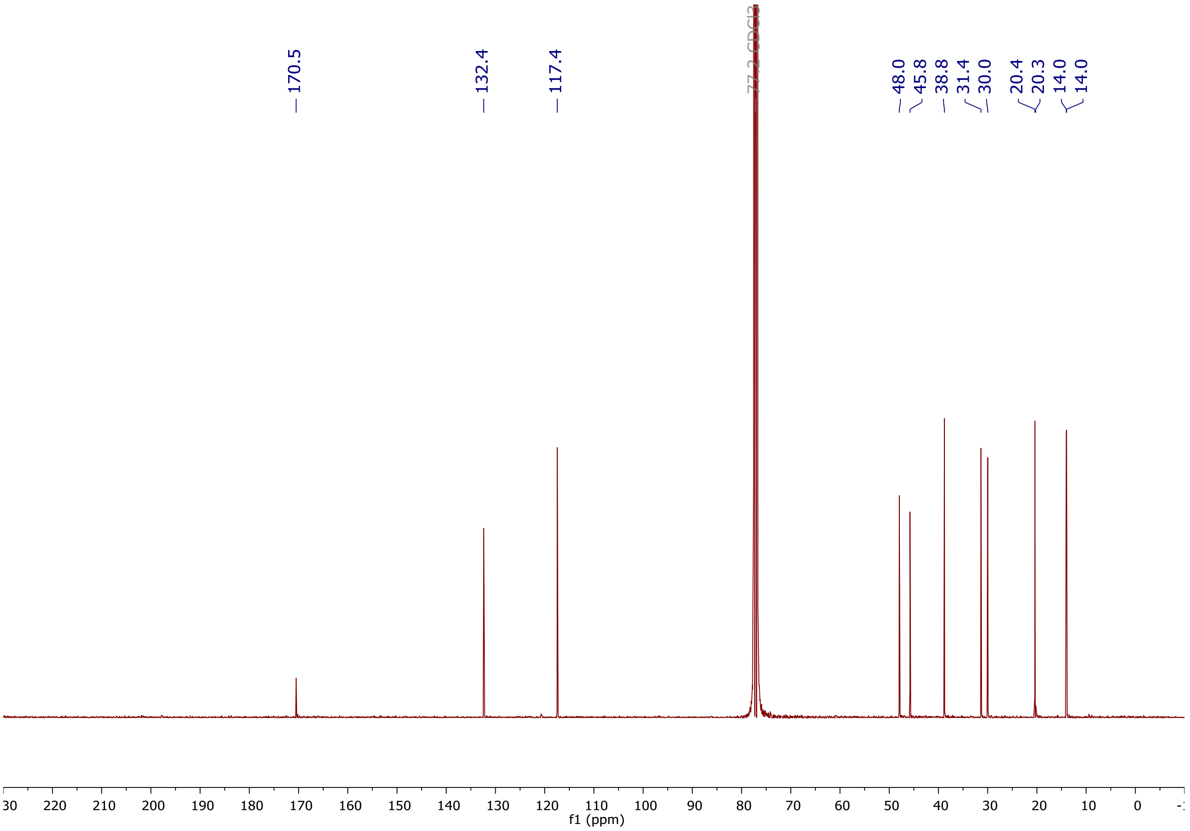

***N*-(2-((Tert-butyldimethylsilyl)oxy)ethyl)-*N*-methylbut-3-enamide (8b)**

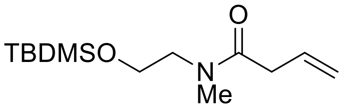

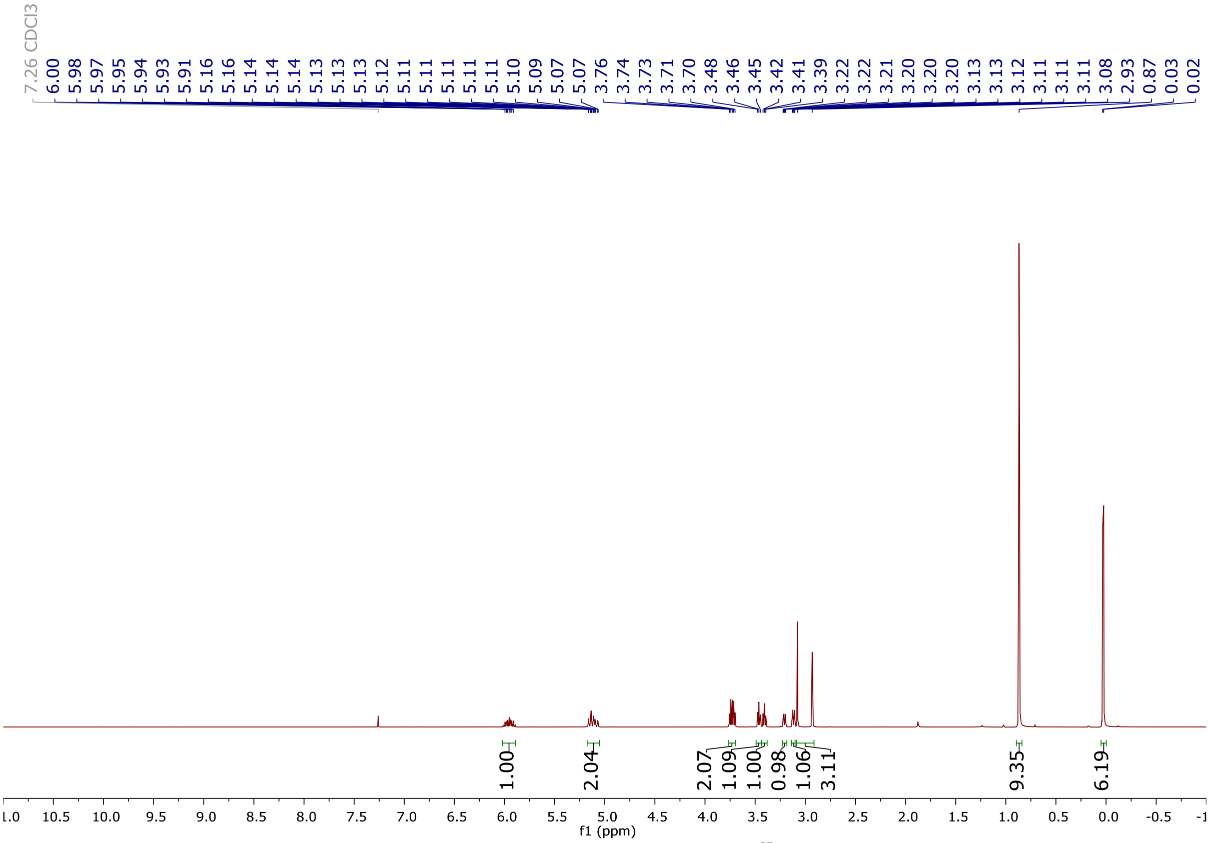

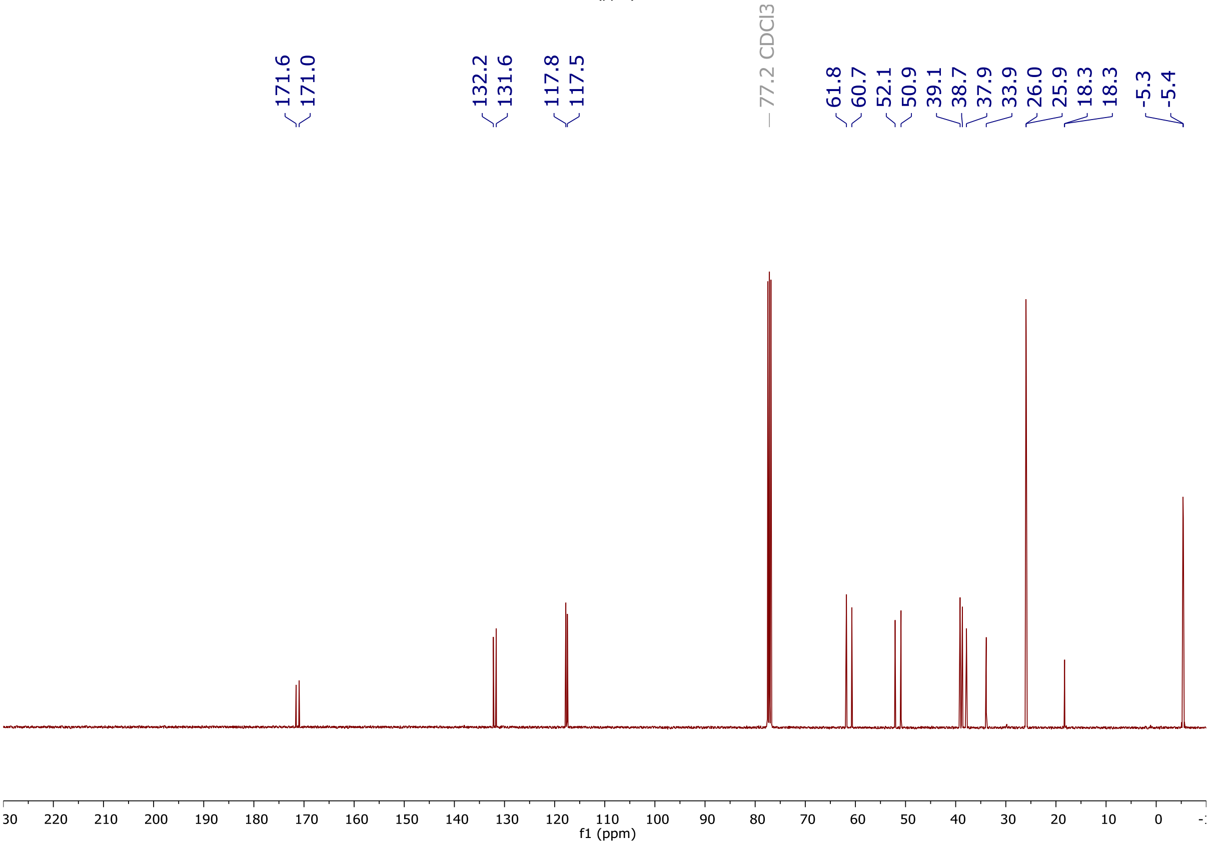

1-Morpholinobut-3-en-1-one (8c)

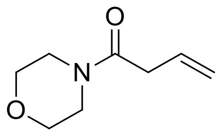

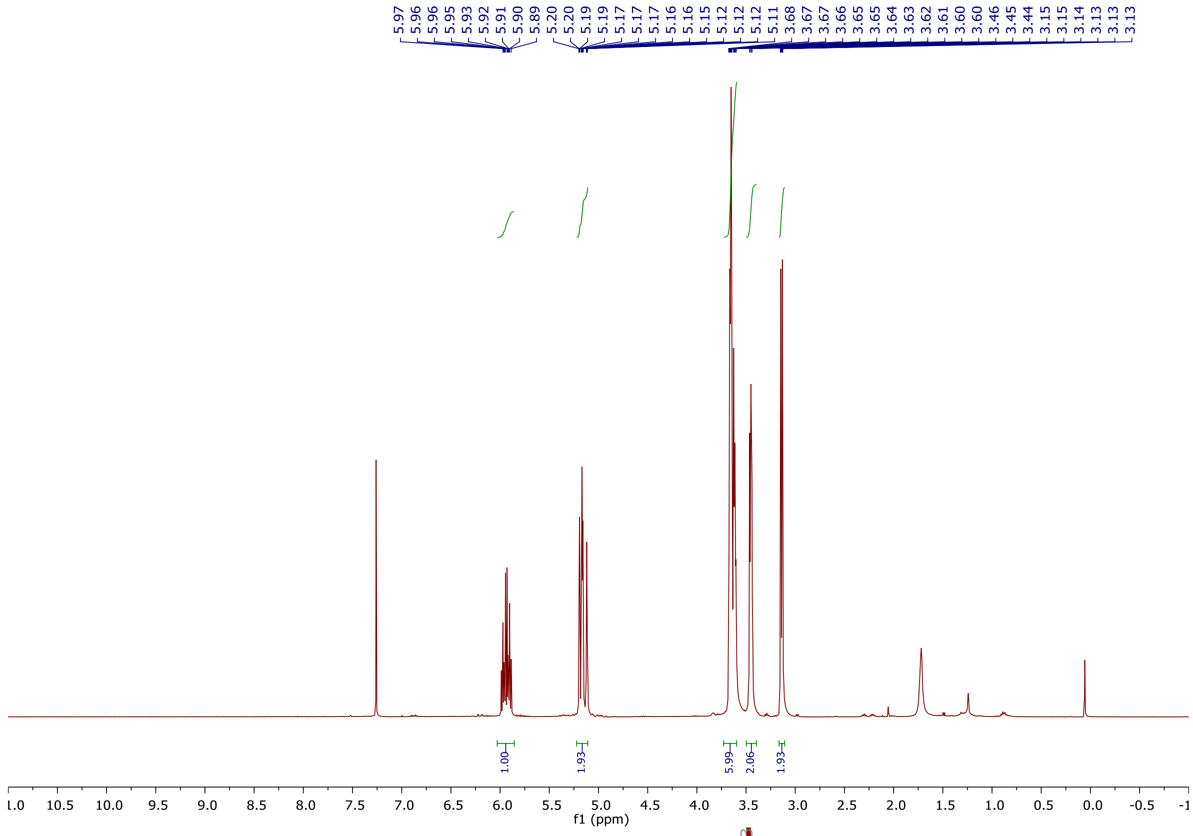

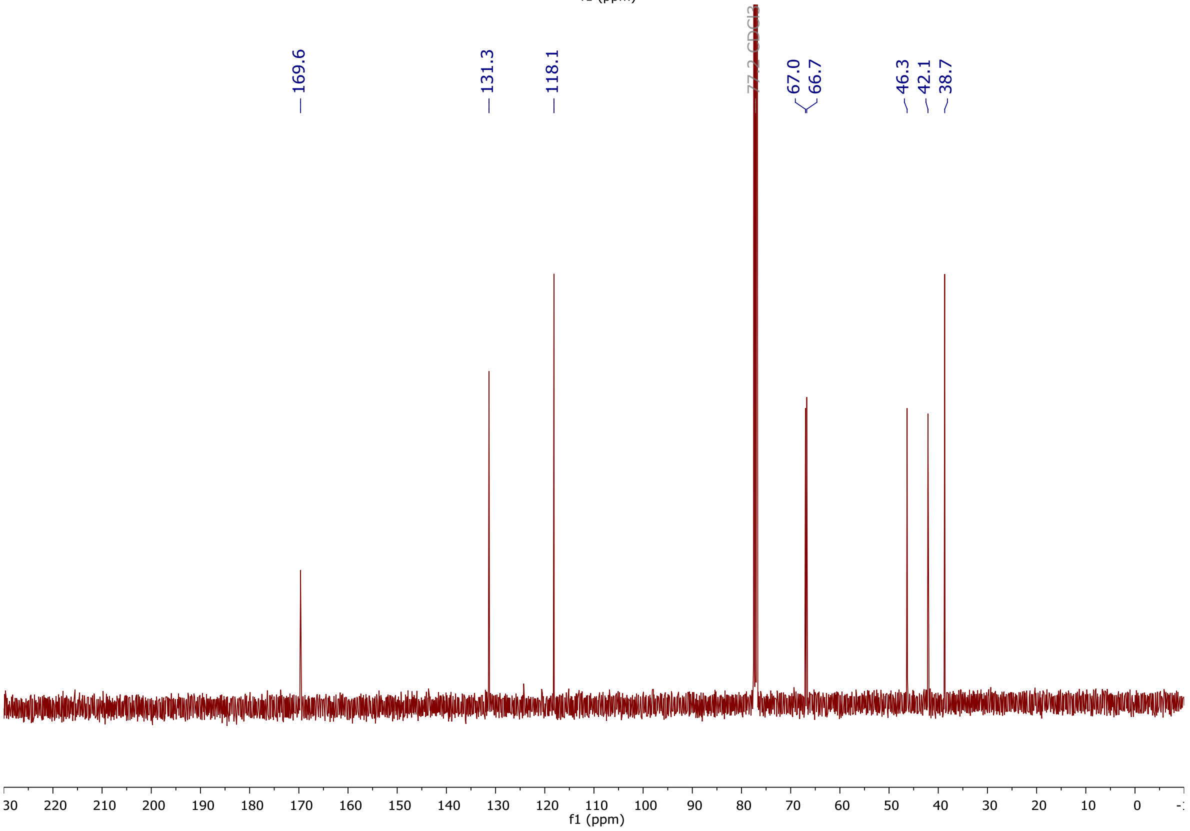

1-(4-Phenylpiperazin-1-yl)but-3-en-1-one (8d)

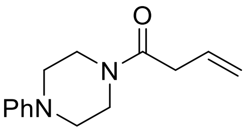

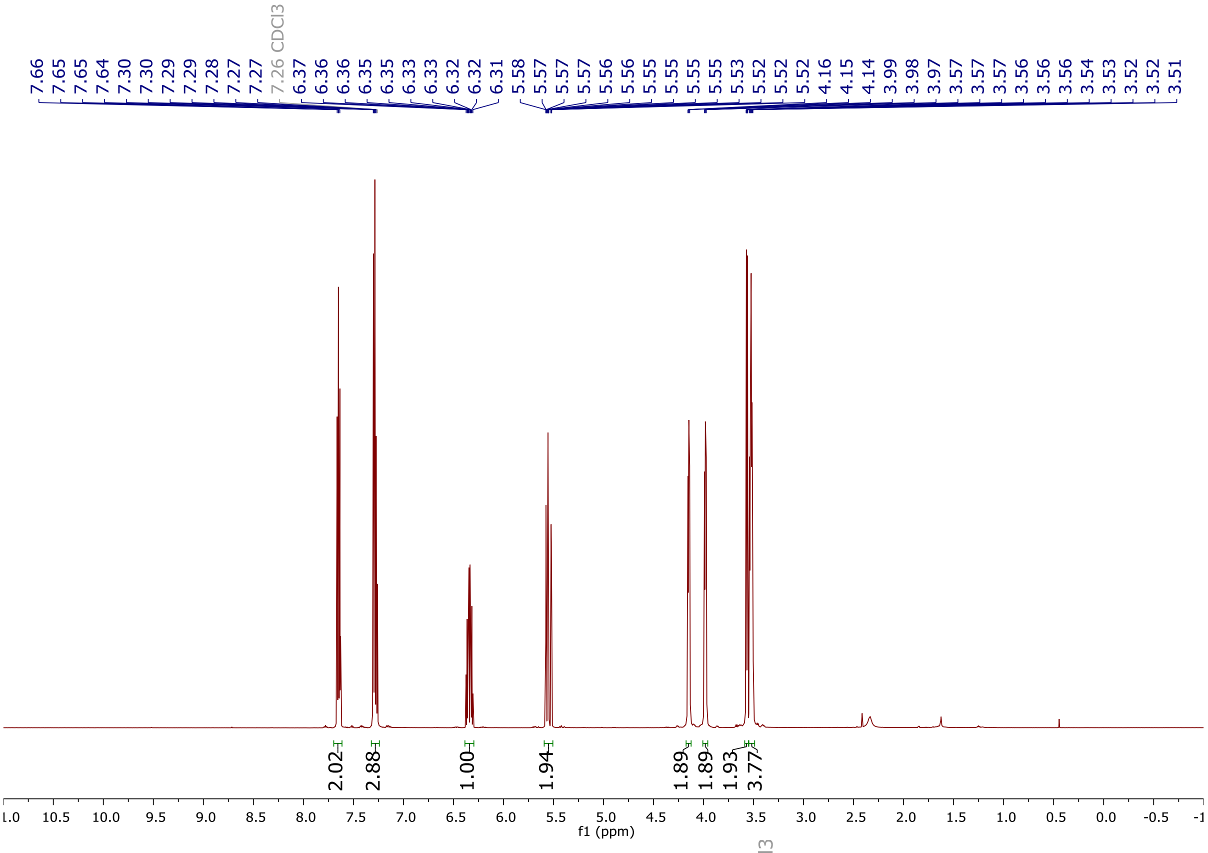

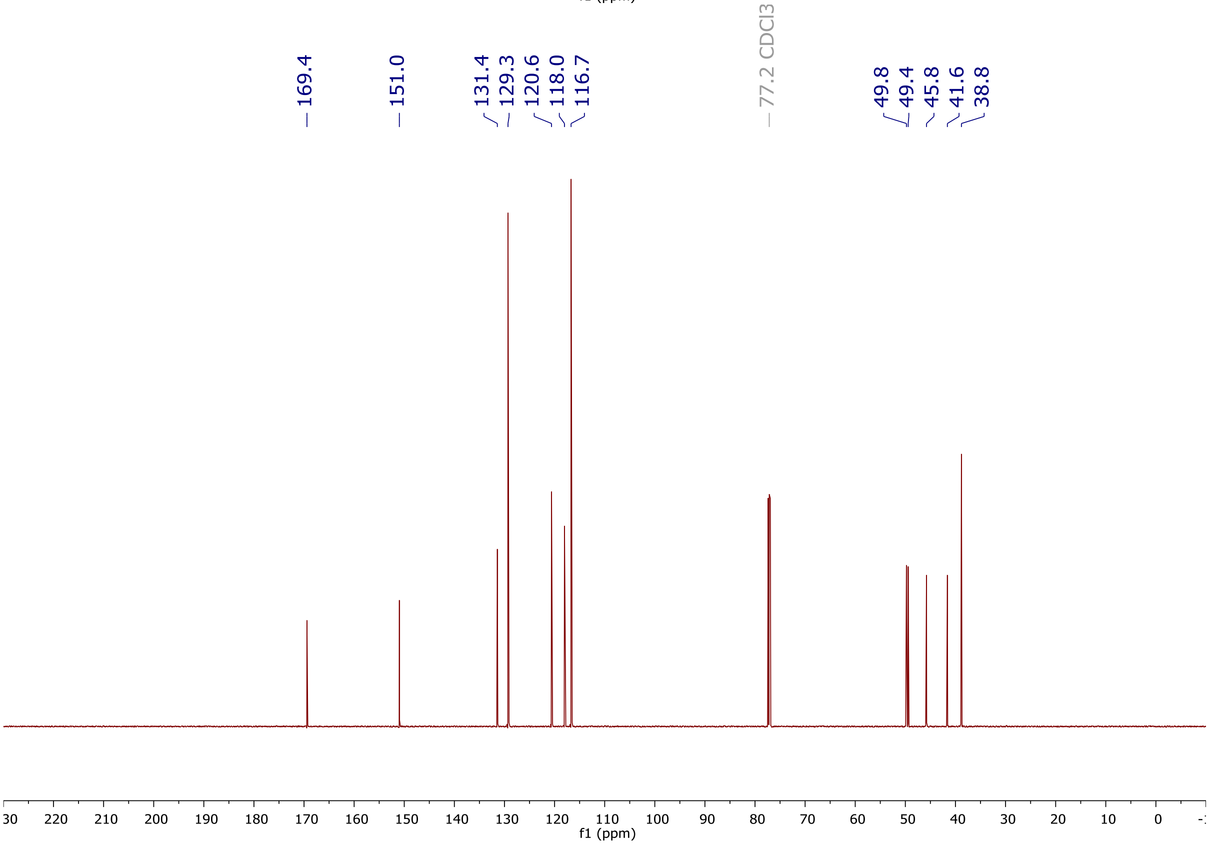

1-((trans)-Octahydroquinolin-2(1H)-yl)but-3-en-1-one (8e)

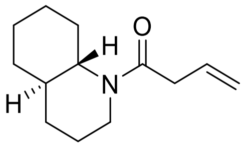

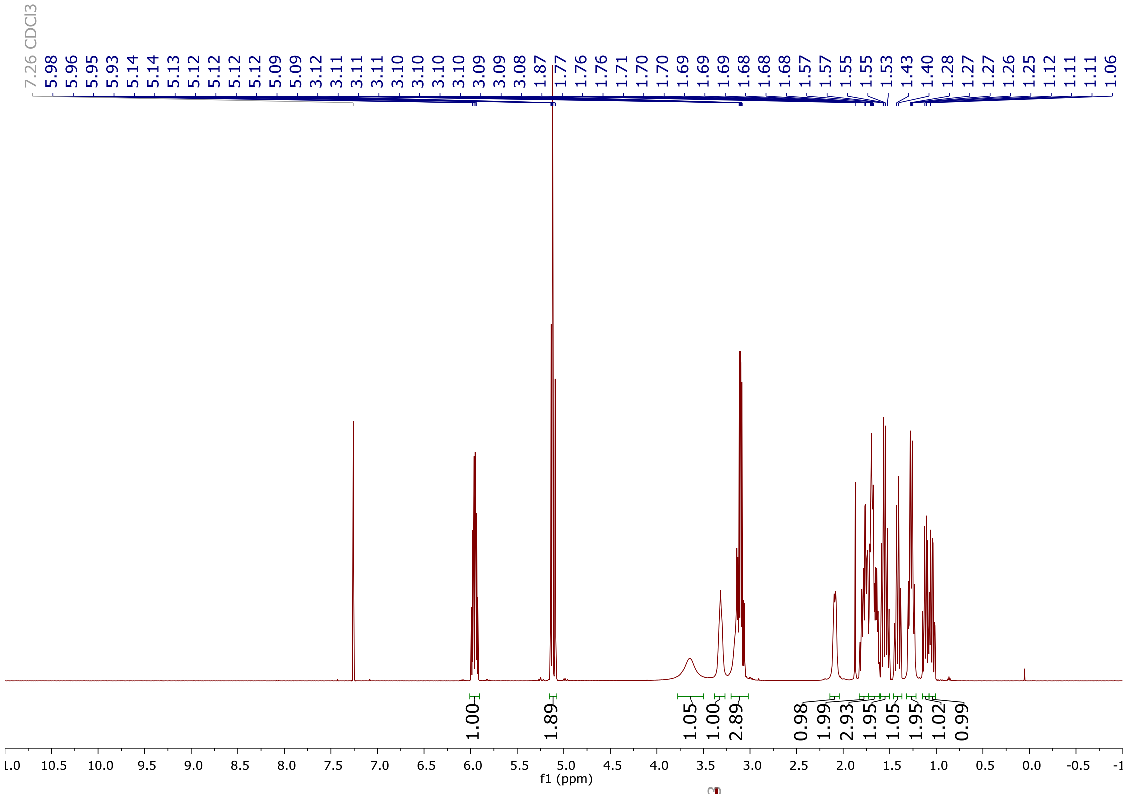

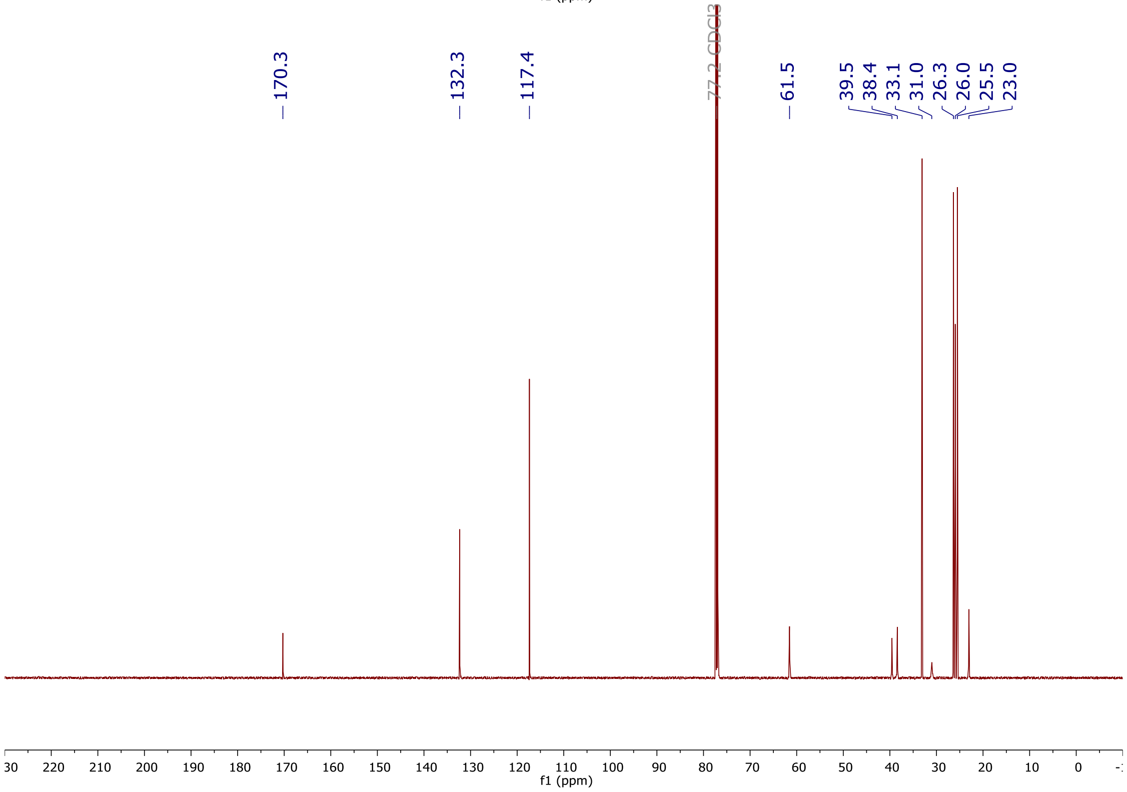

1-(4-(3-(Trifluoromethyl)pyridin-2-yl)piperazin-1-yl)but-3-en-1-one (8f)

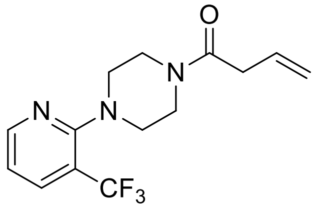

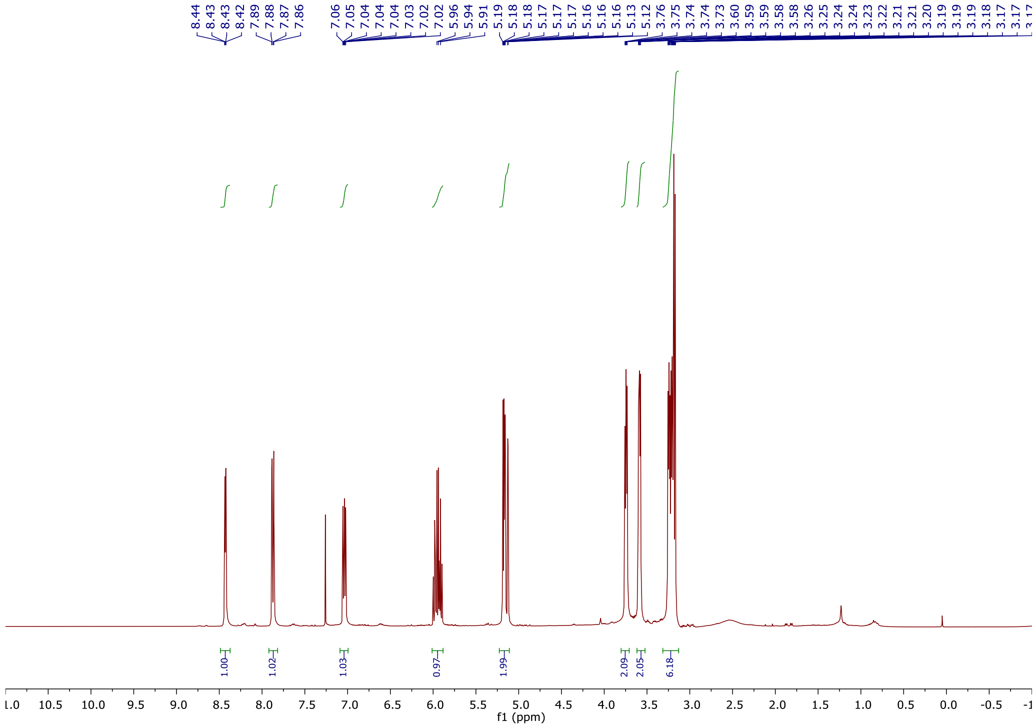

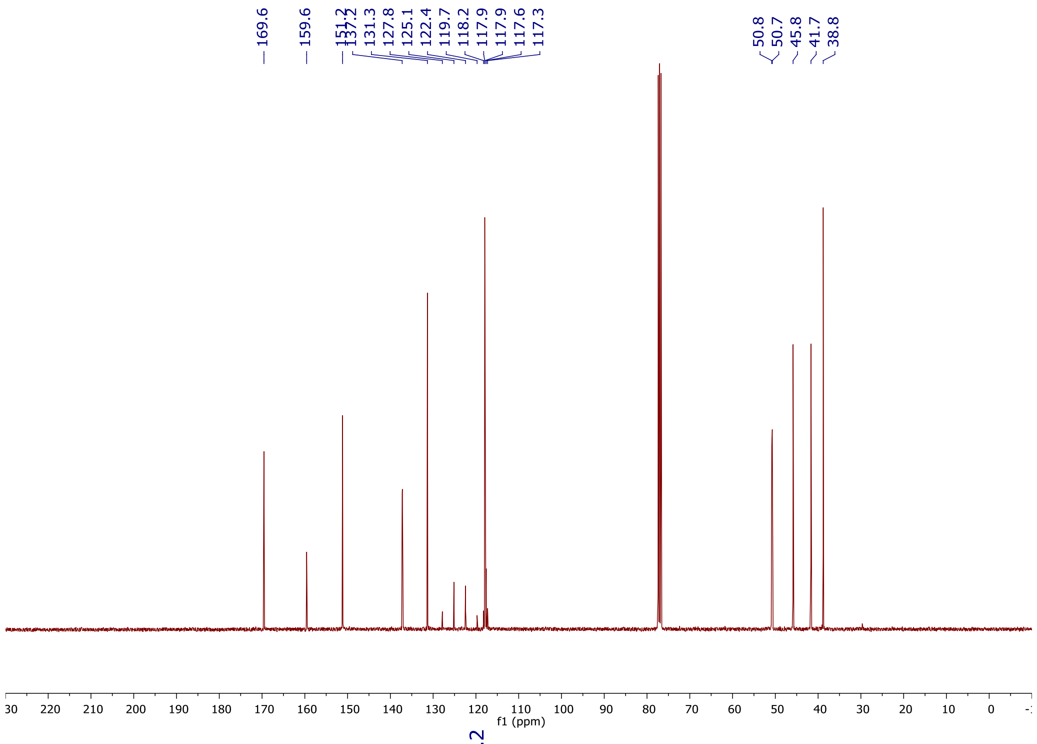
169.6
159.6
151.2
137.2
131.3
127.8
125.1
122.4
119.7
118.2
117.9
117.9
117.6
117.3
50.8
50.7
45.8
41.7
38.8
f1 (ppm)

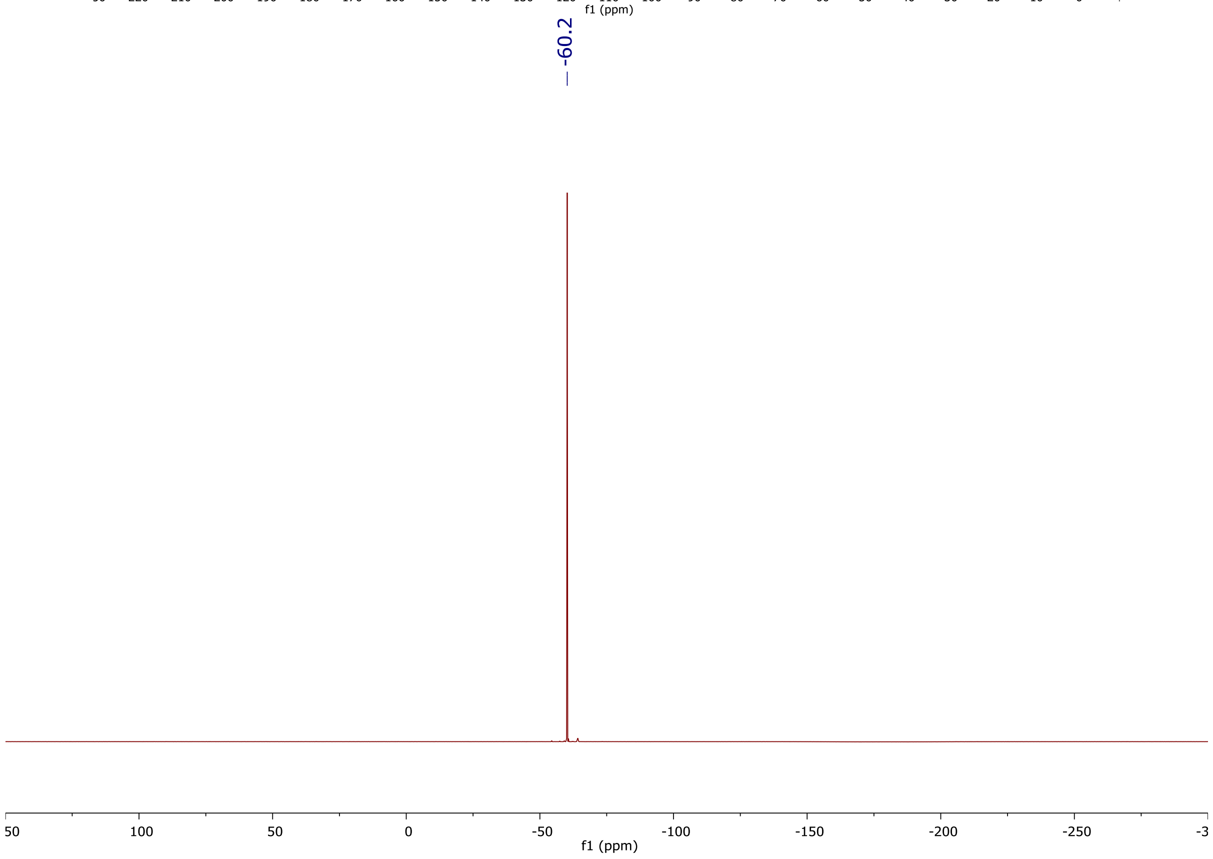
-60.2
f1 (ppm)

Cyclohex-2-en-1-yl(7,7,9,9-tetramethyl-1,4-dioxa-8-azaspiro[4.5]decan-8-yl)methanone (8g)

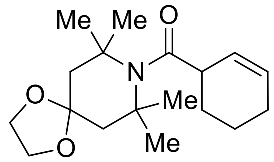

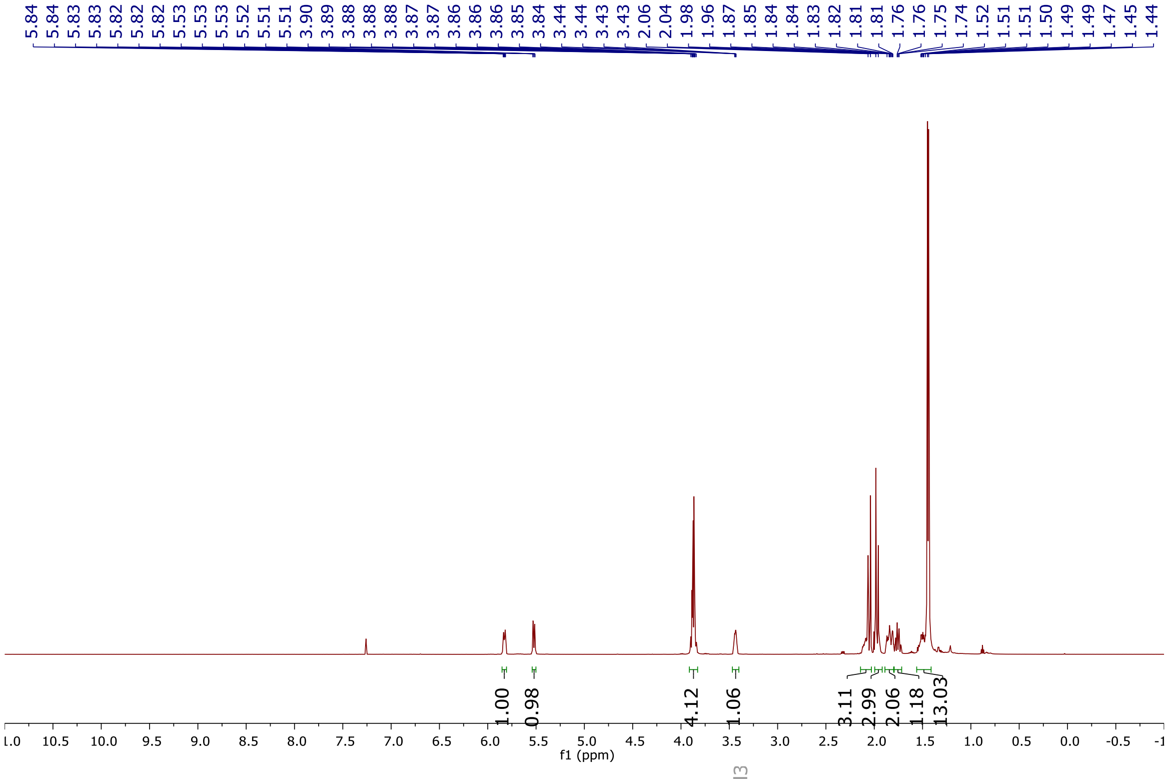

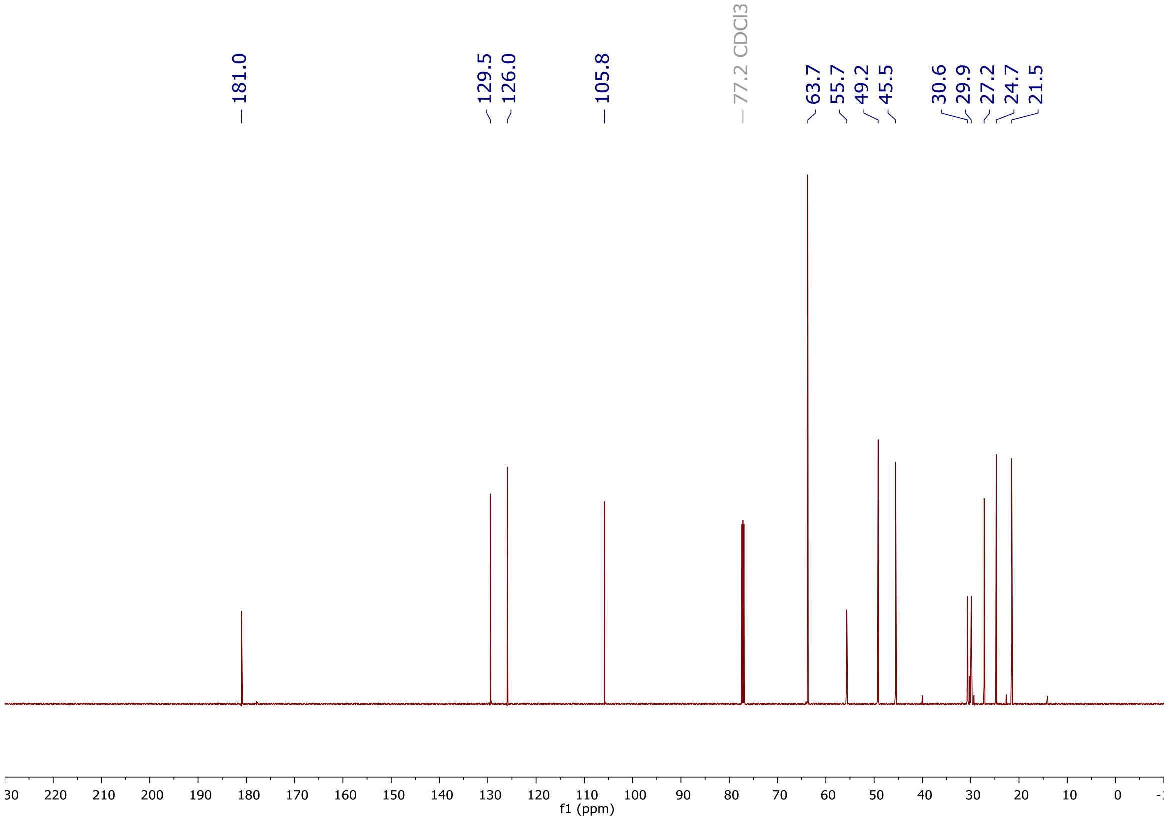

(*S*)-1-(2-(Methoxymethyl)pyrrolidin-1-yl)-4-methylpent-3-en-1-one (8h)

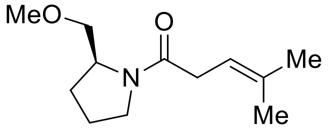

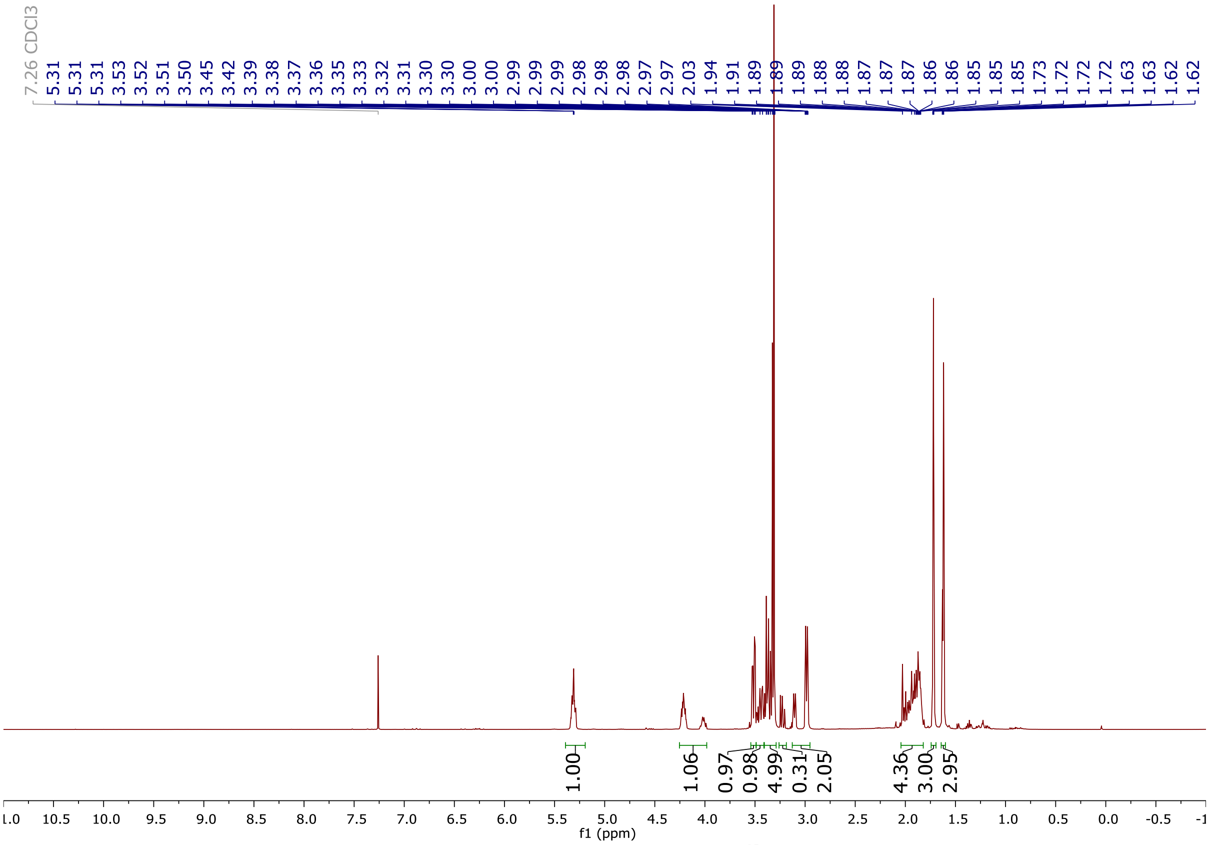

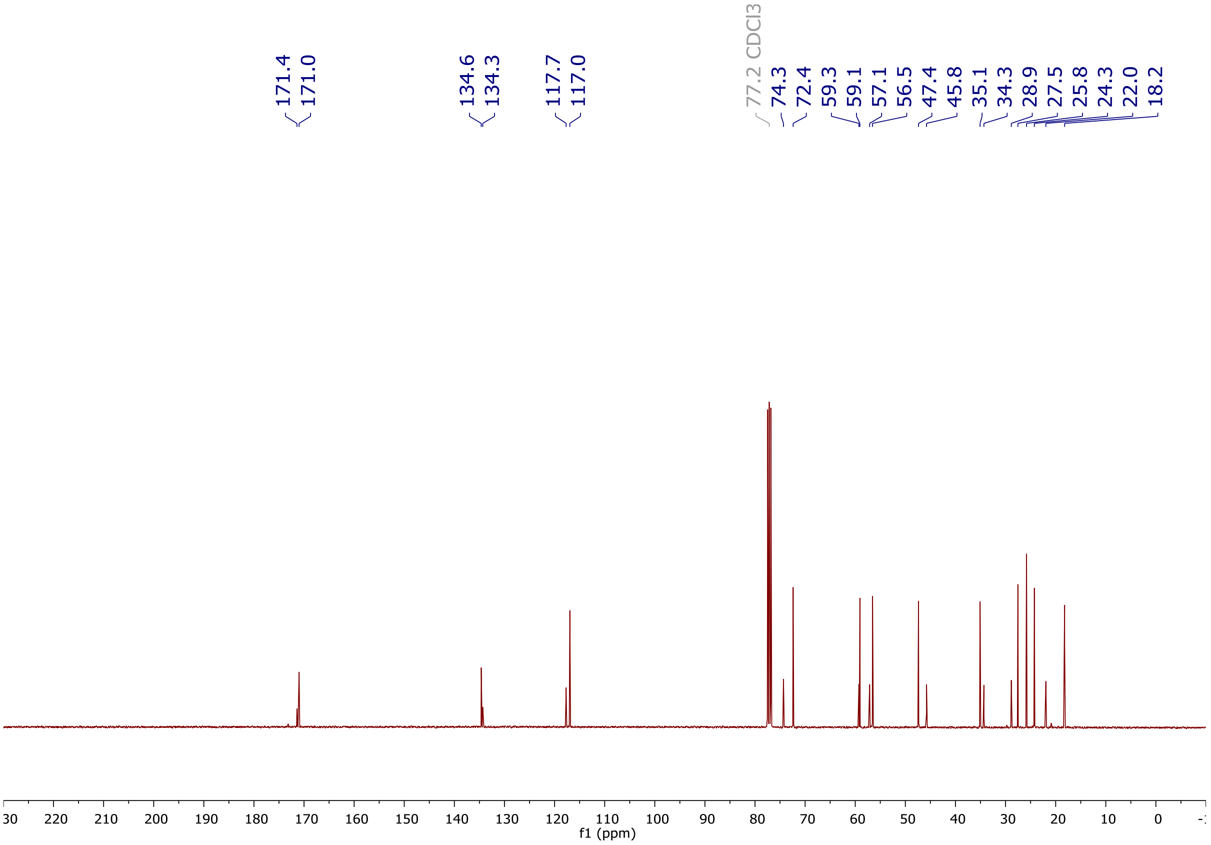

4-Methyl-1-morpholinopent-3-en-1-one (8i)

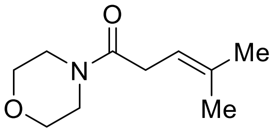

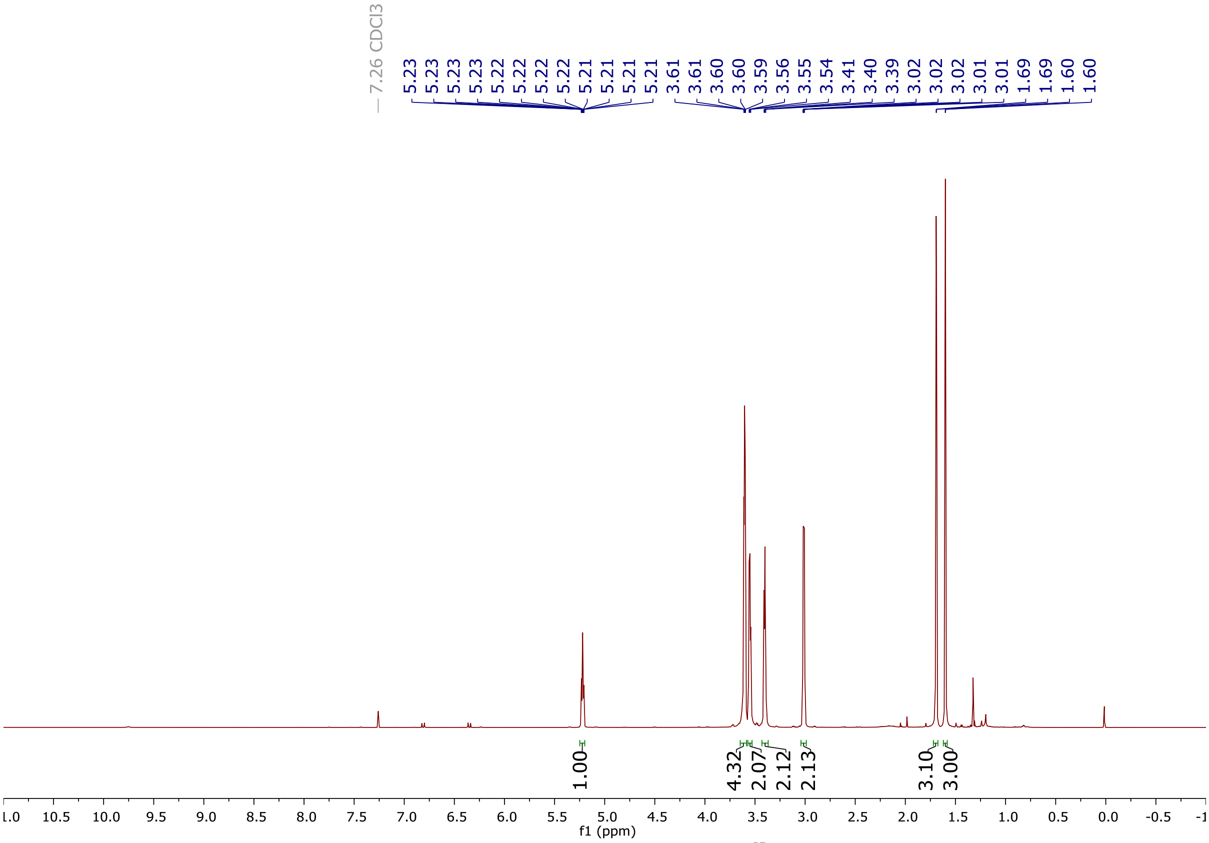

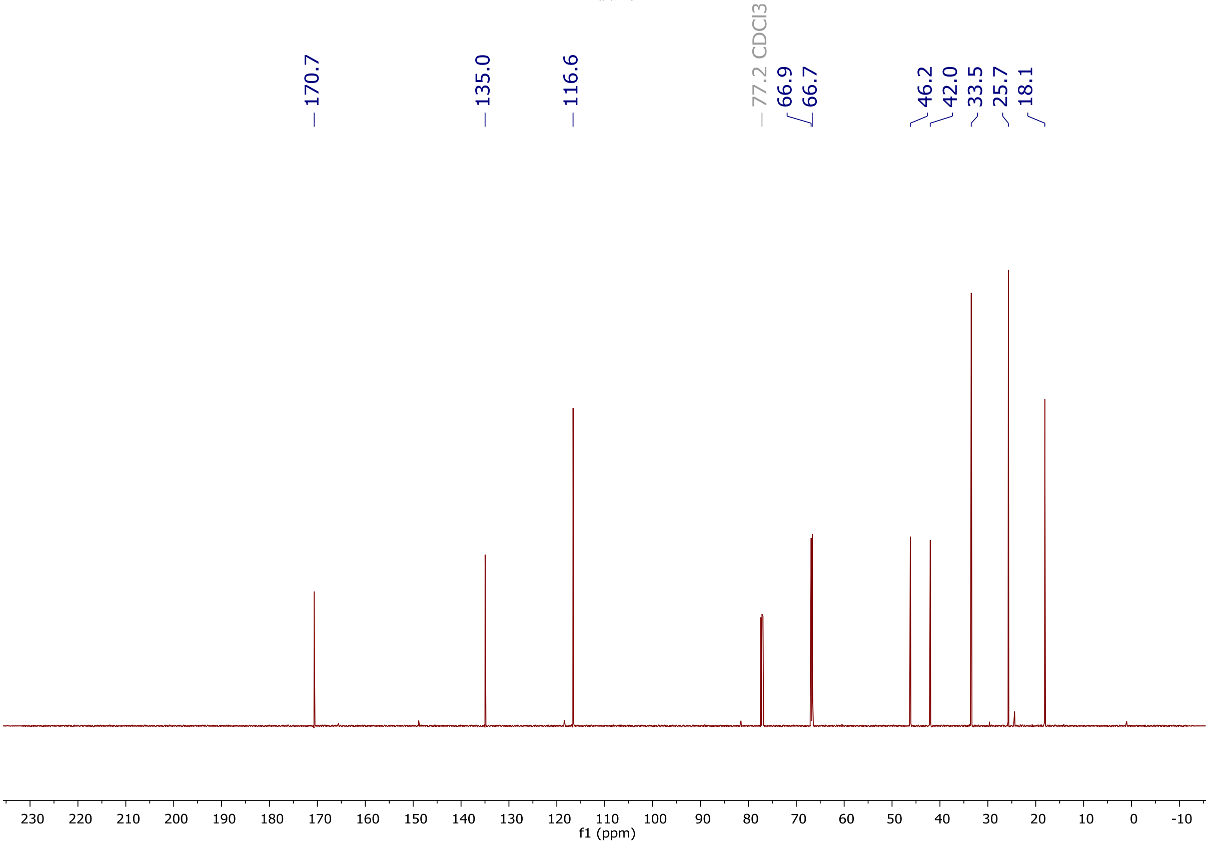

(*E*)-1-Morpholino-4-phenylbut-3-en-1-one (8j)

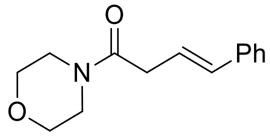

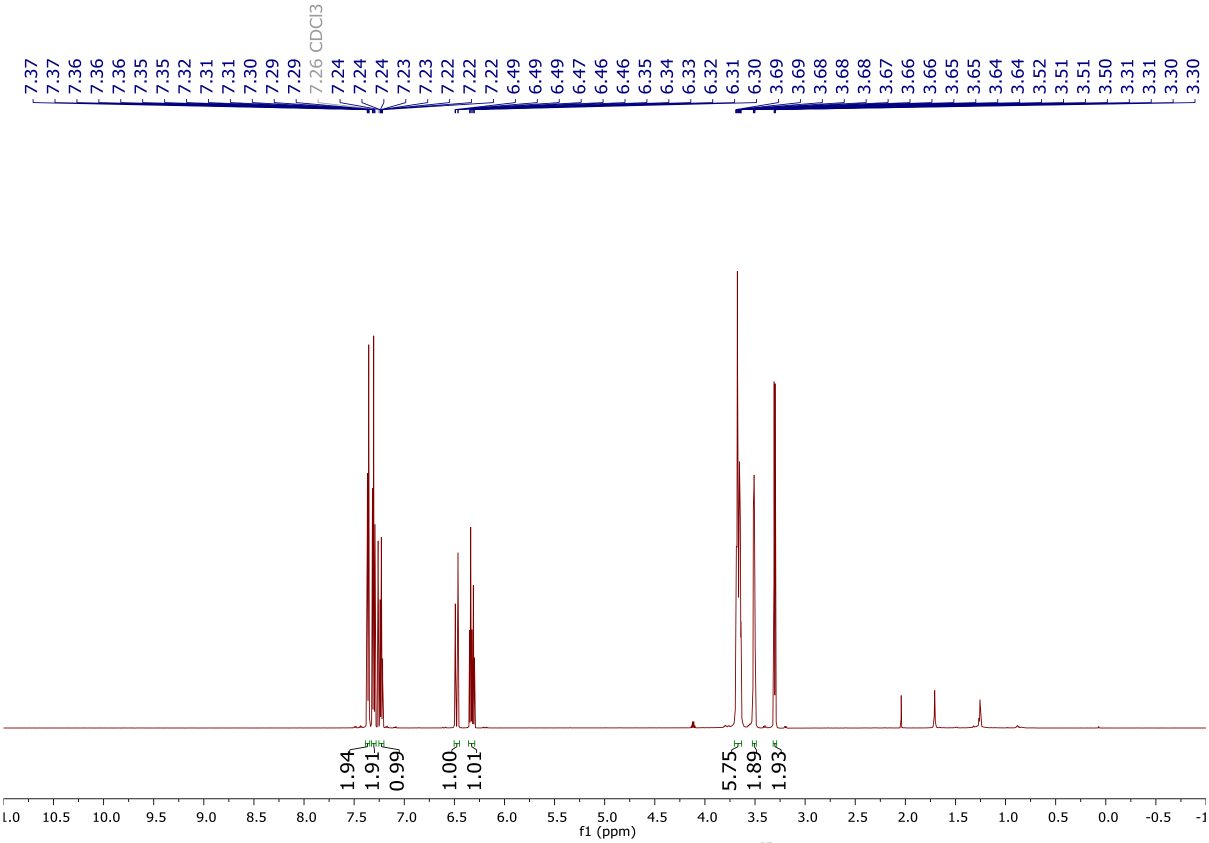

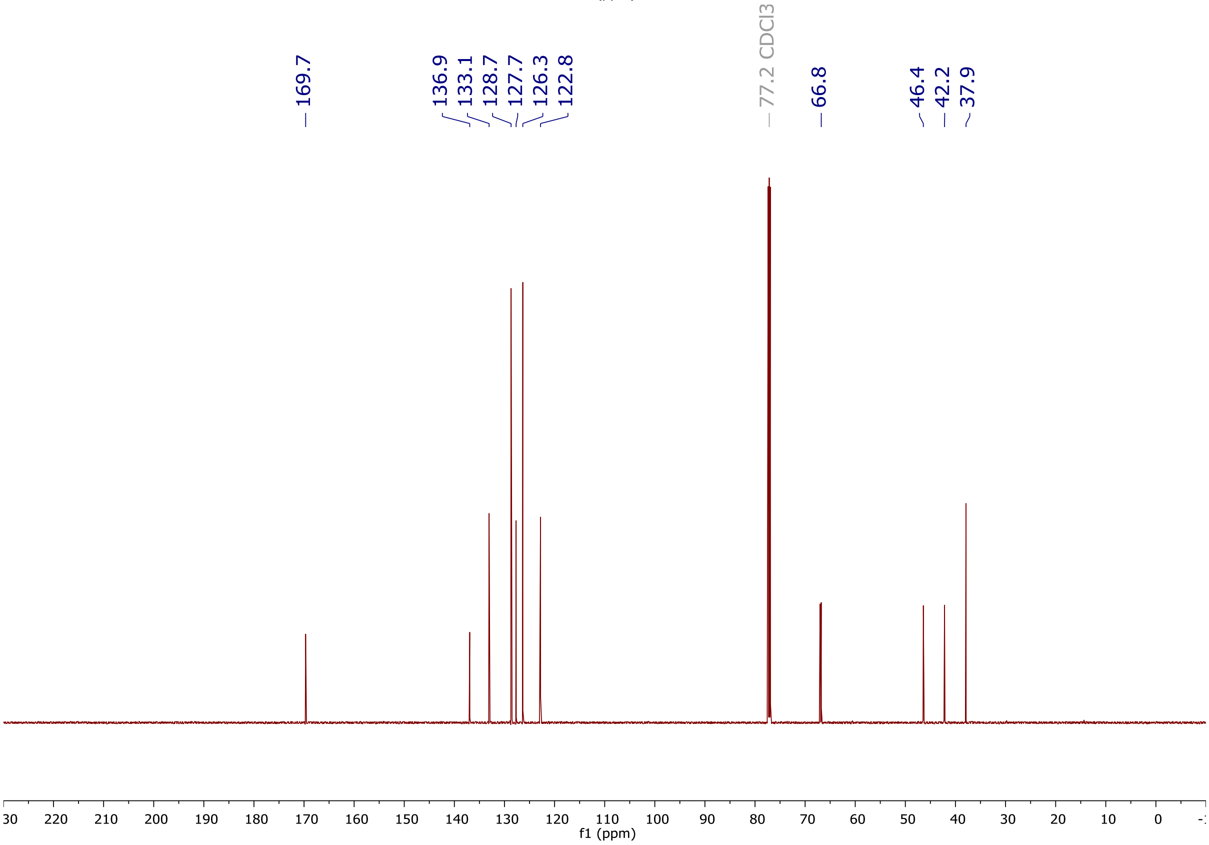

1-(Piperidin-1-yl)buta-2,3-dien-1-one (8k)

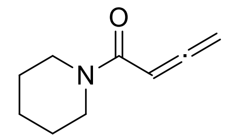

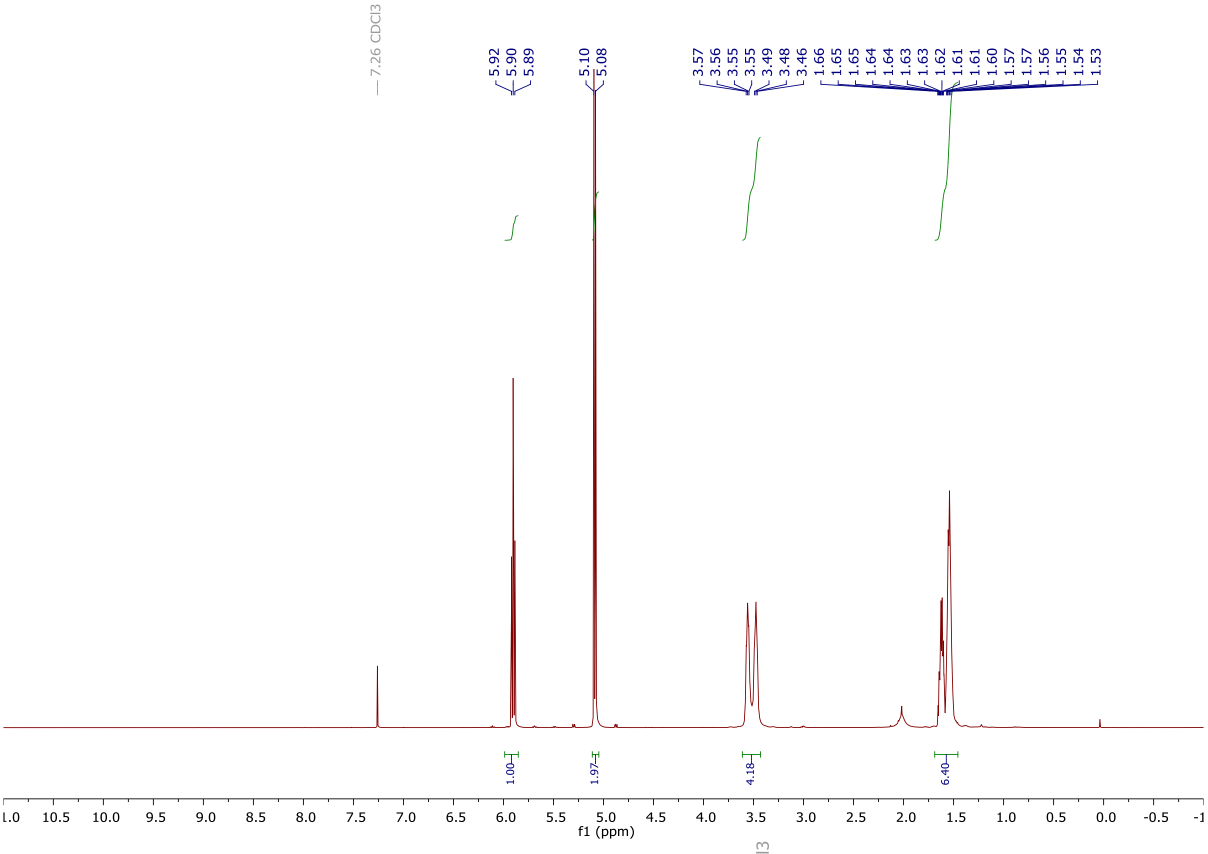

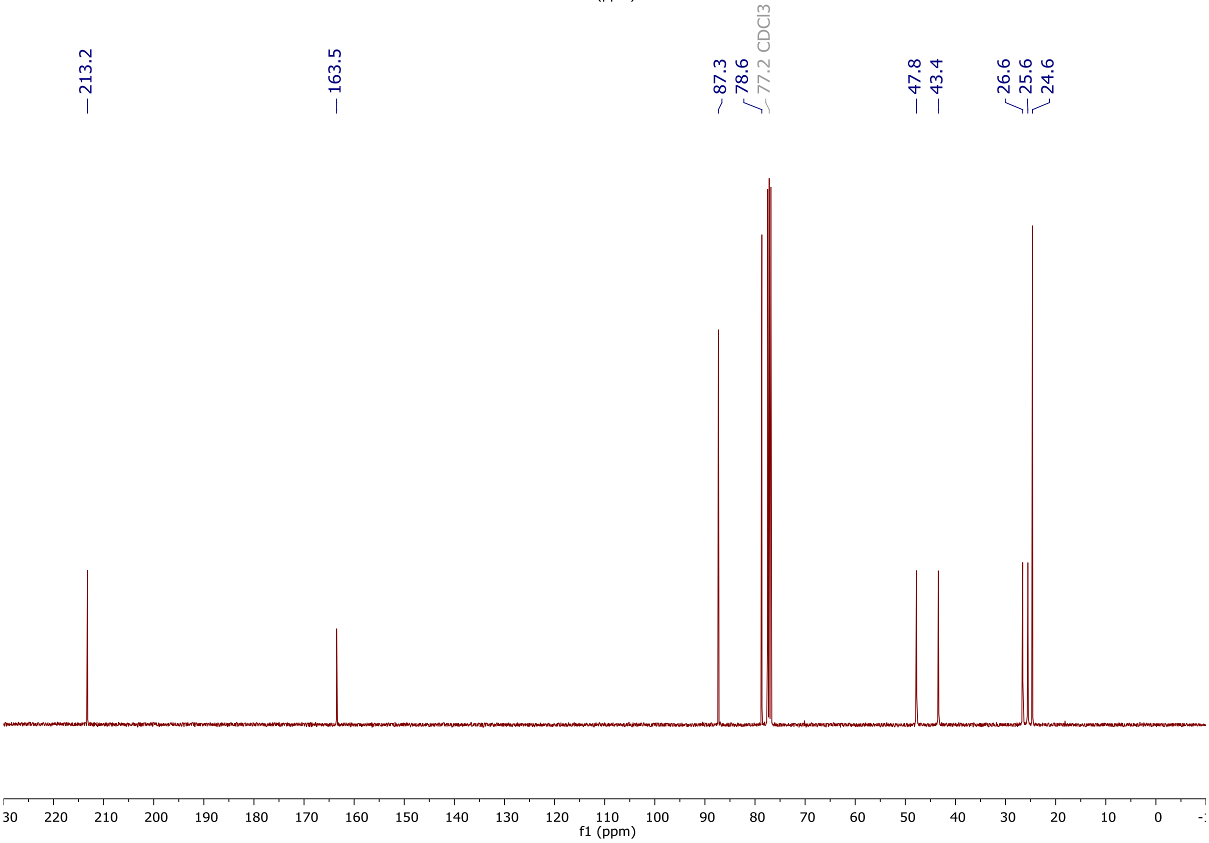

Ethyl 4-(dibutylamino)-2-methylene-4-oxobutanoate-4-[*13C*] (8l)

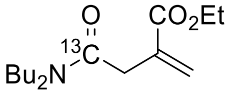

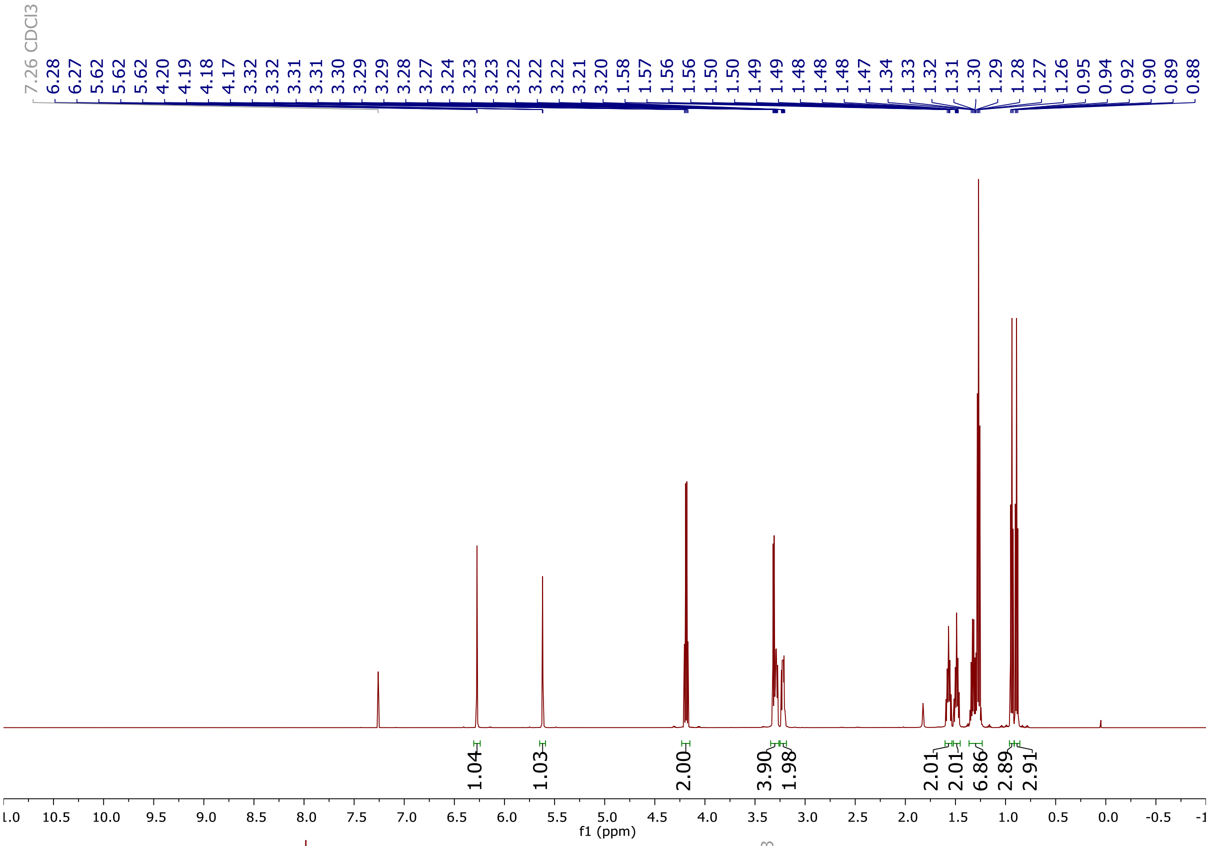

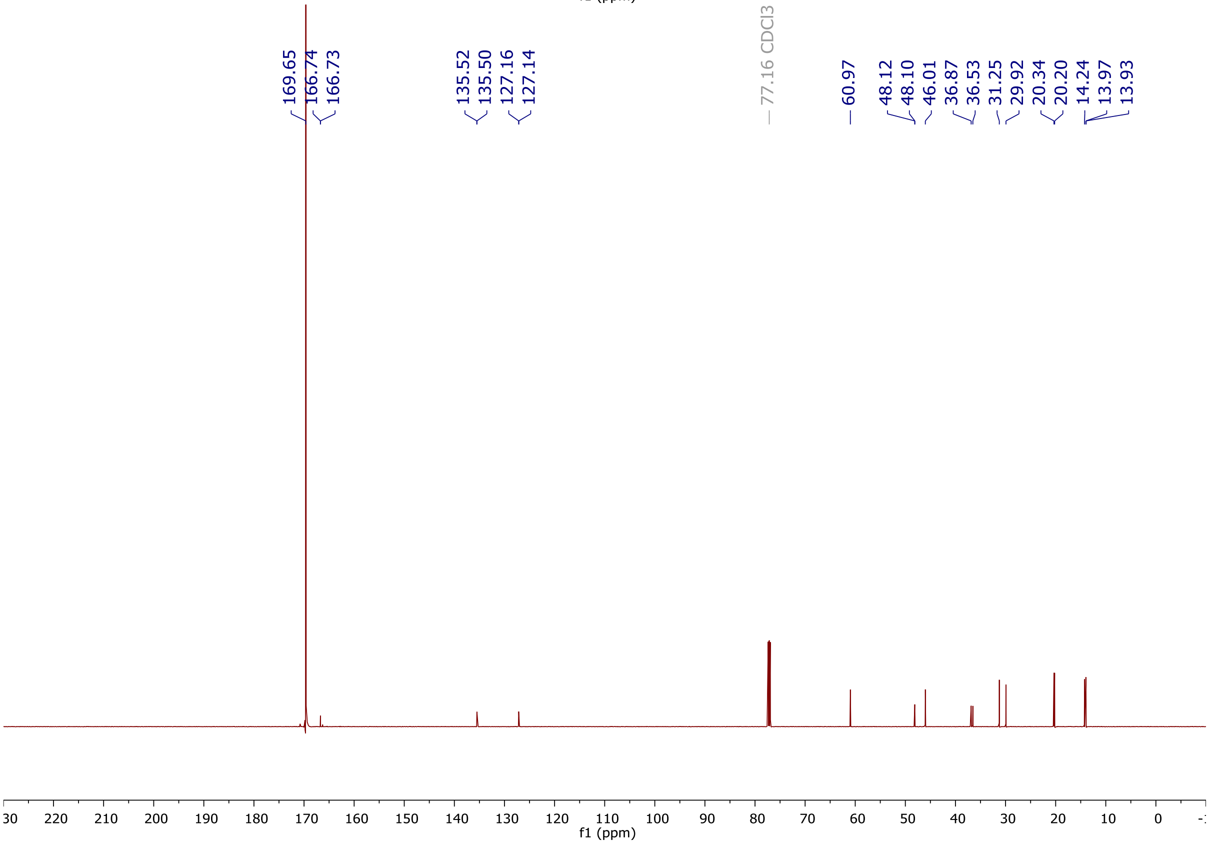

Ethyl 4-(2-(*N*-methylcyclohex-2-ene-1-carboxamido)ethoxy)benzoate (8m)

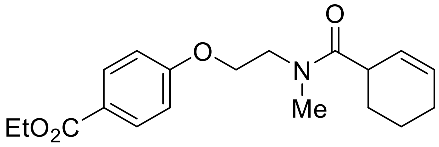

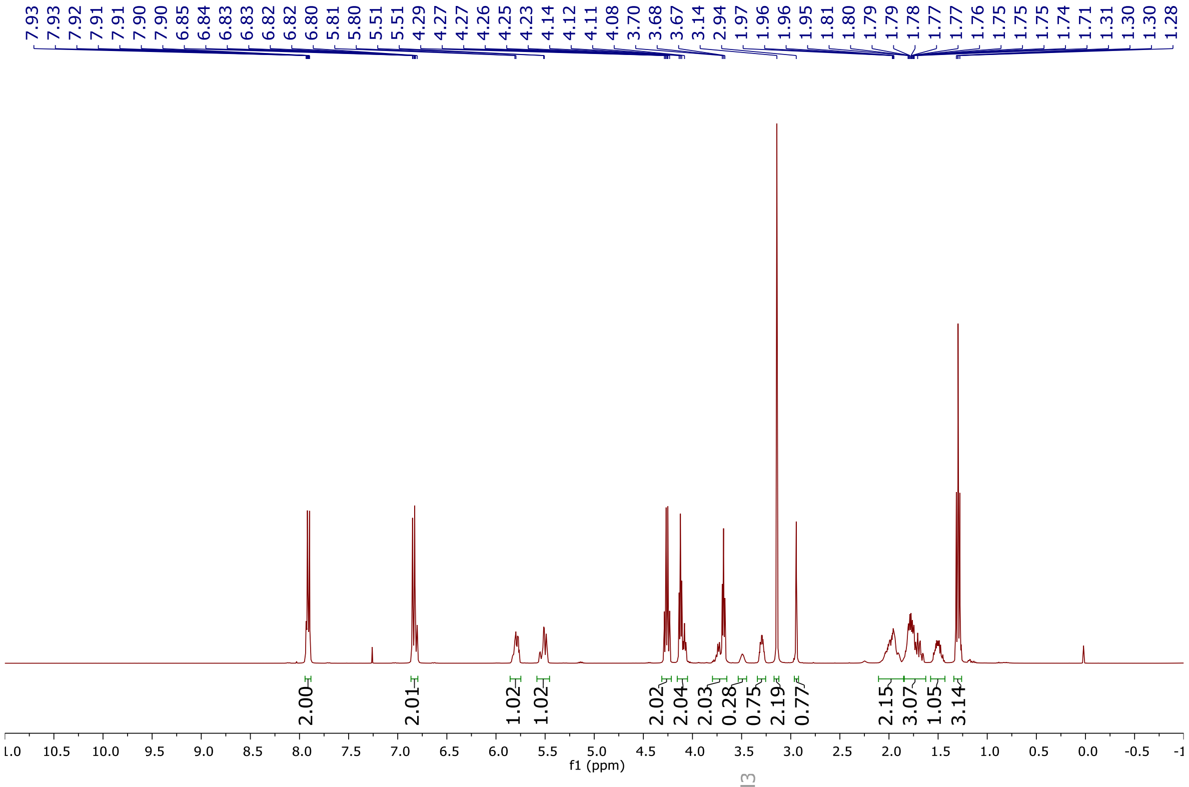

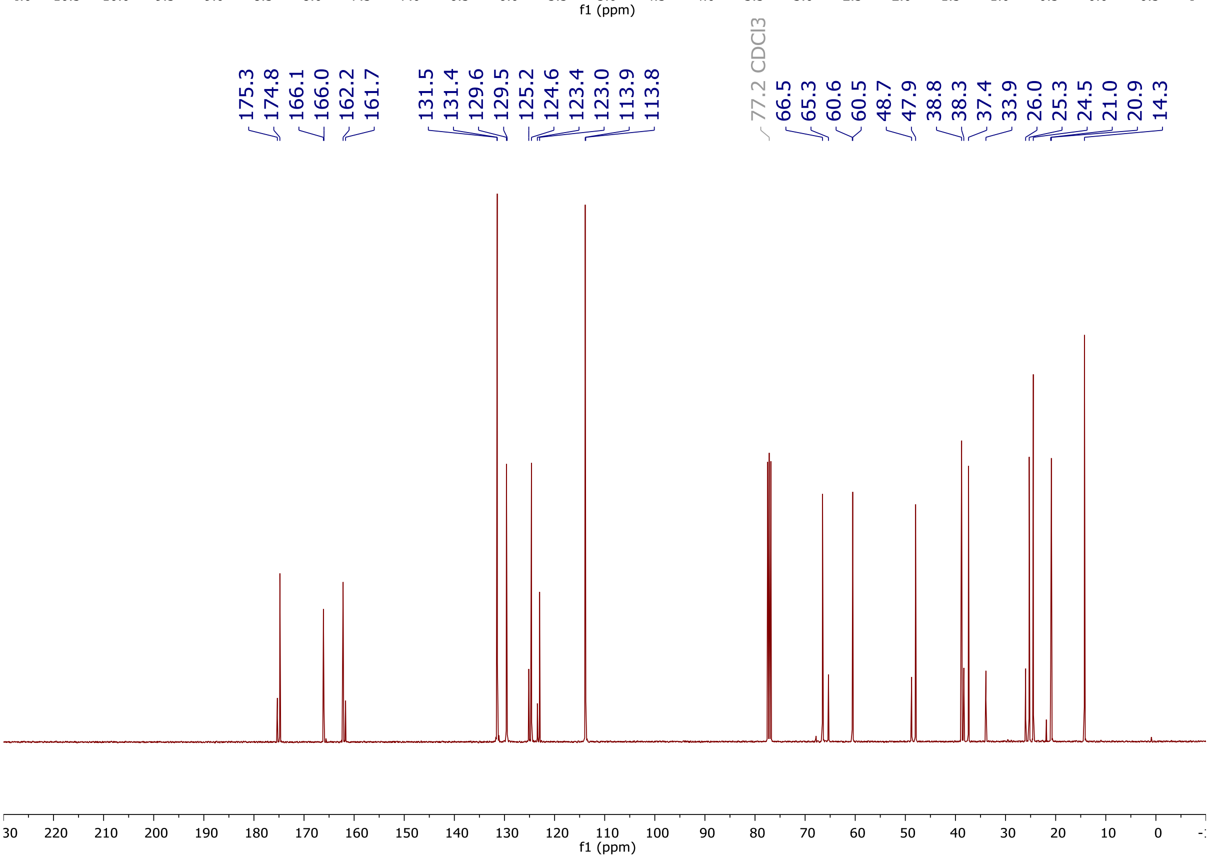

N-(2-(2-benzoylphenoxy)ethyl)-N,3-dimethylbut-3-enamide (8n)

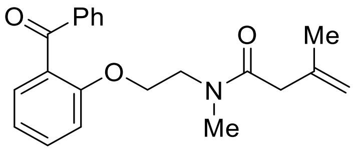

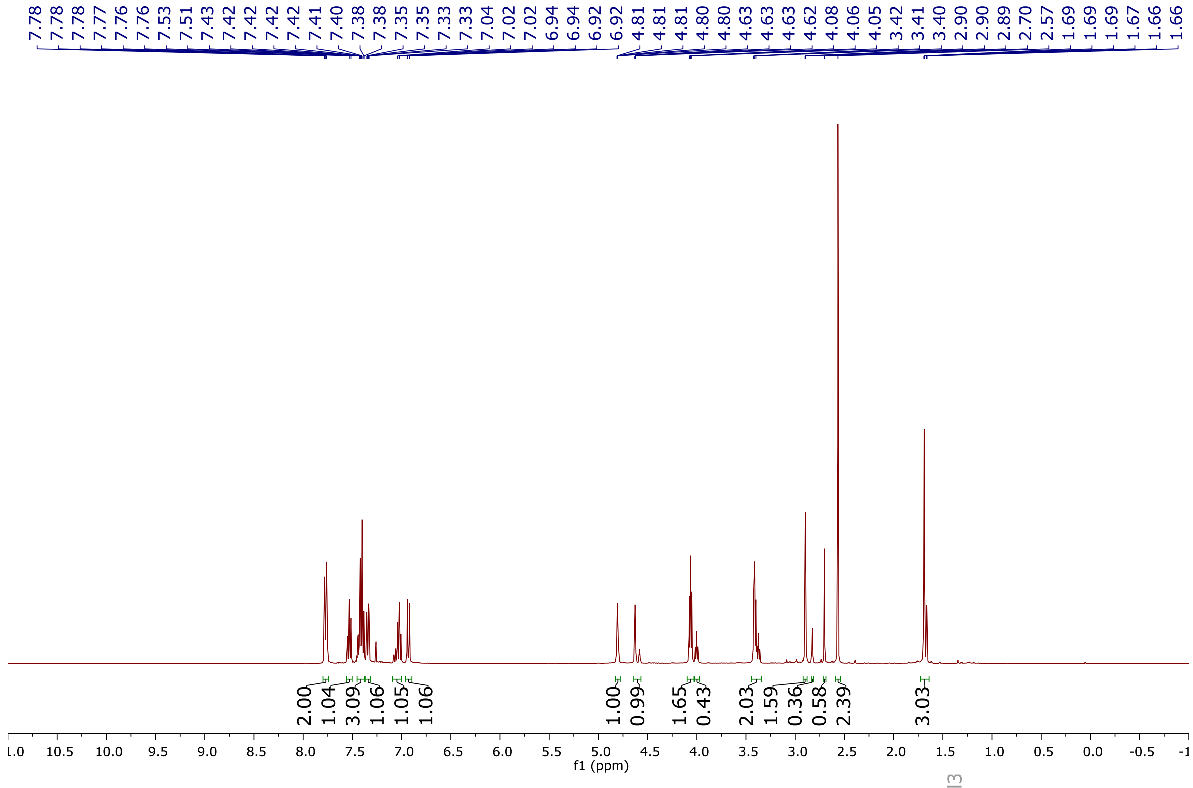

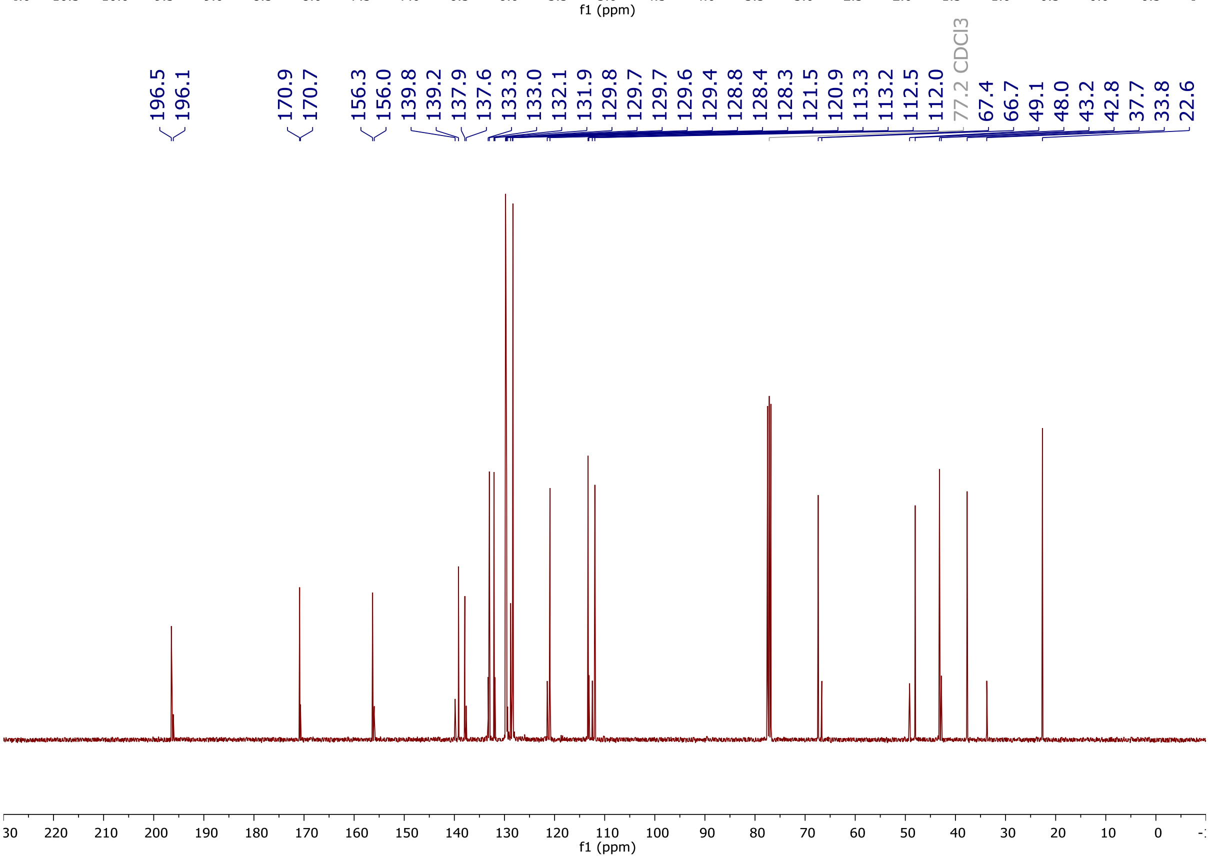

***N*-(3-(1,3-dioxoisoindolin-2-yl)propyl)-*N*-isopropyl-3-methylbut-3-enamide (8o)**

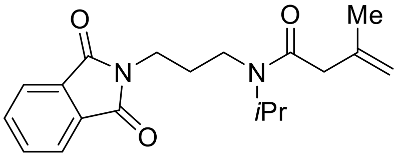

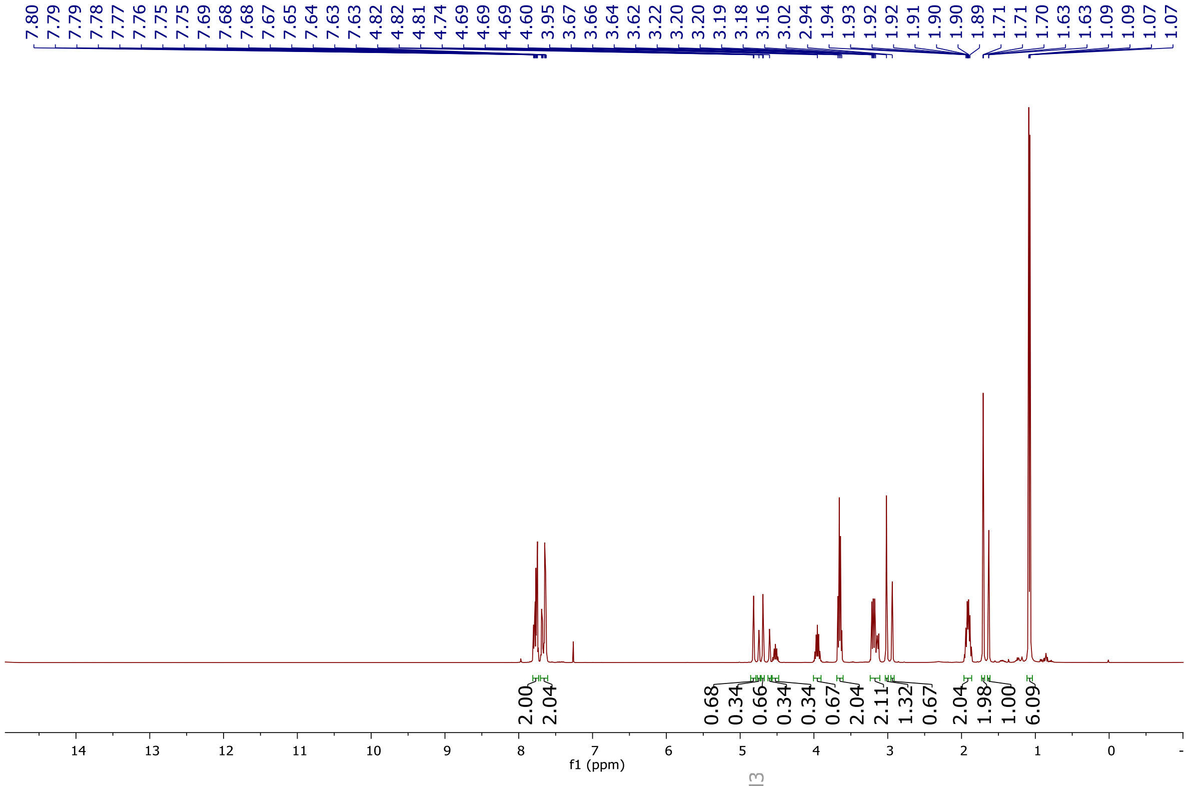

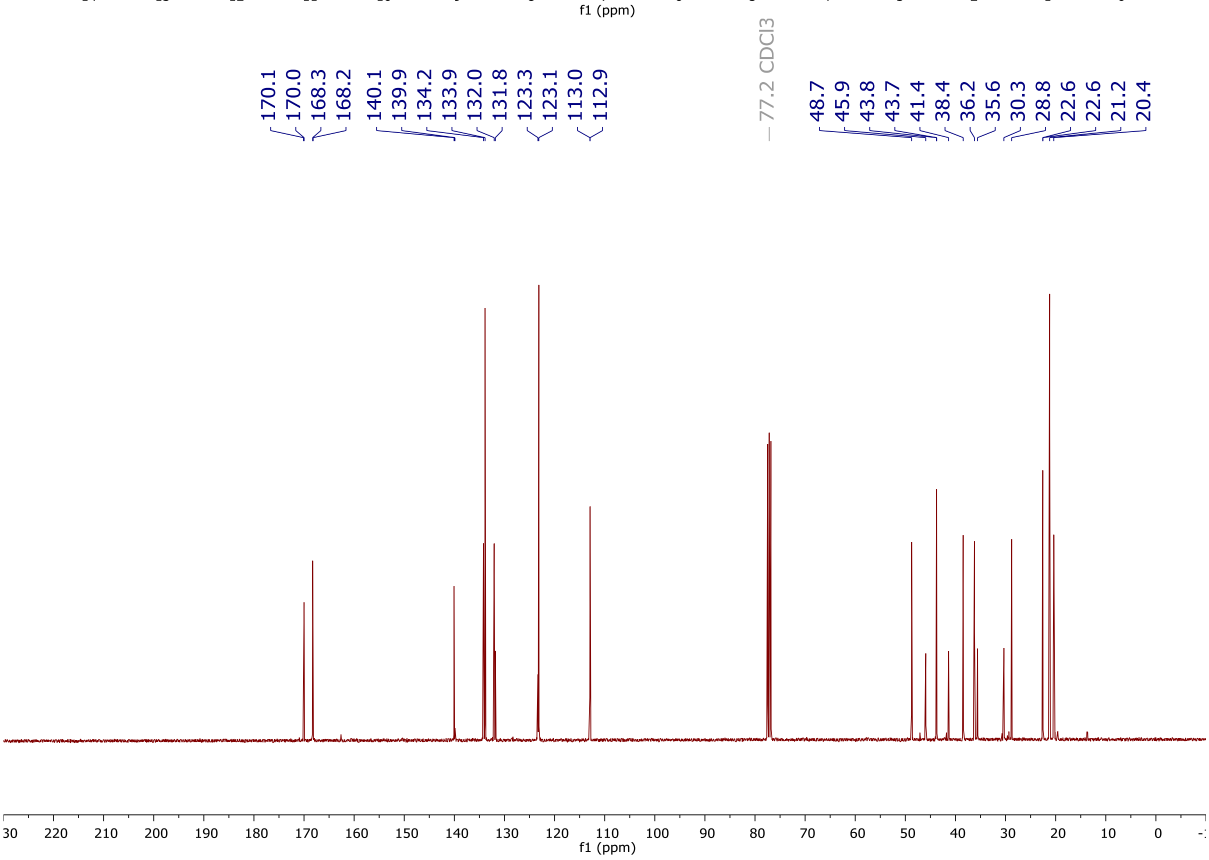

_N_-methyl-_N_-(2-(4-nitrophenoxy)ethyl)but-3-enamide (8p)

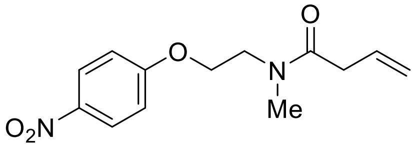

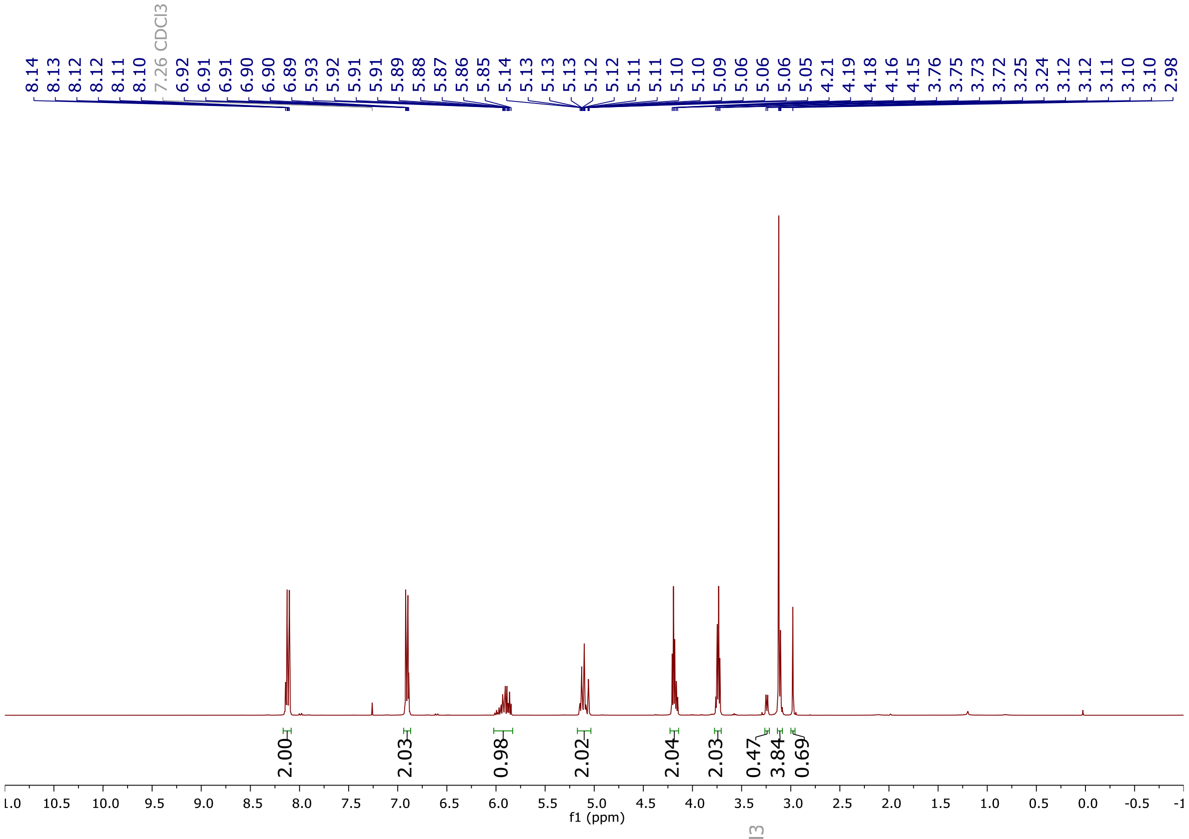

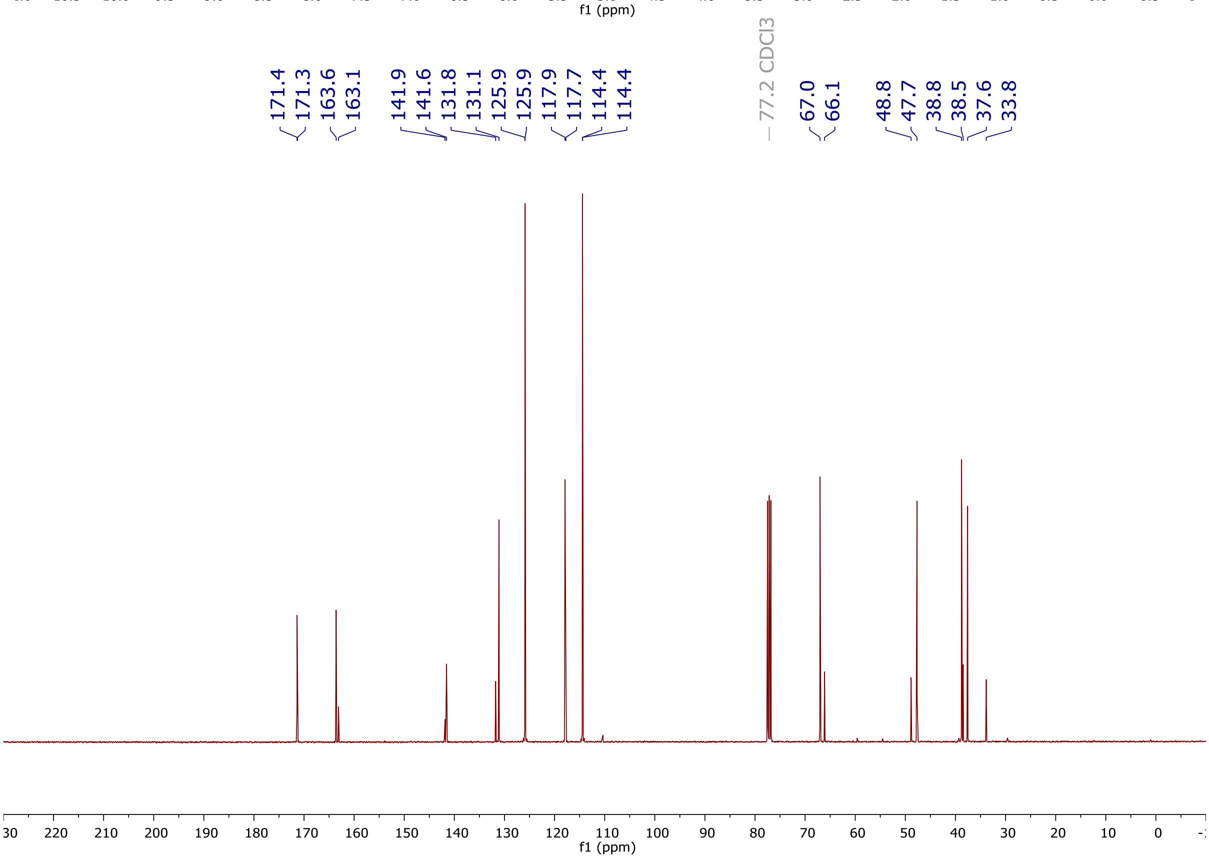

1-(4-(Pyrimidin-2-yl)piperazin-1-yl)but-3-en-1-one (8q)

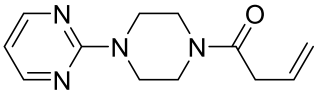

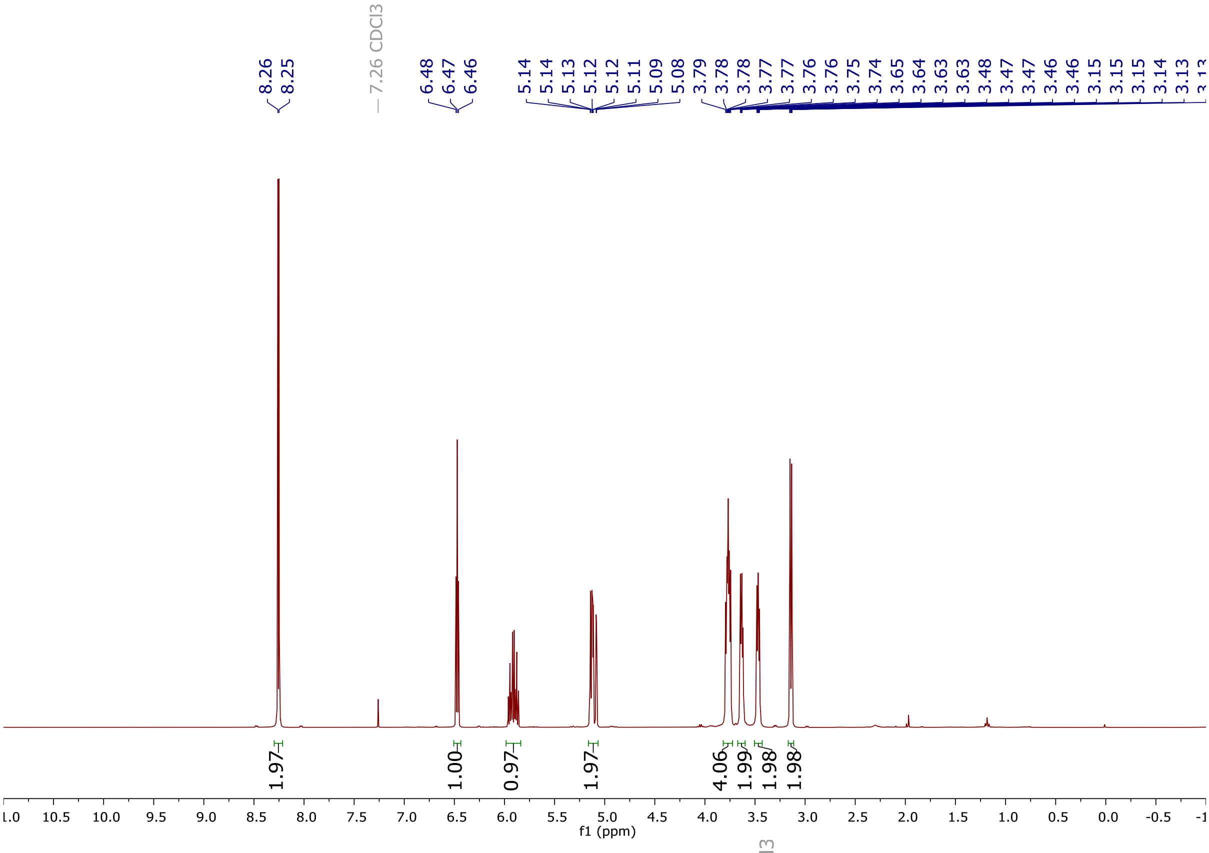

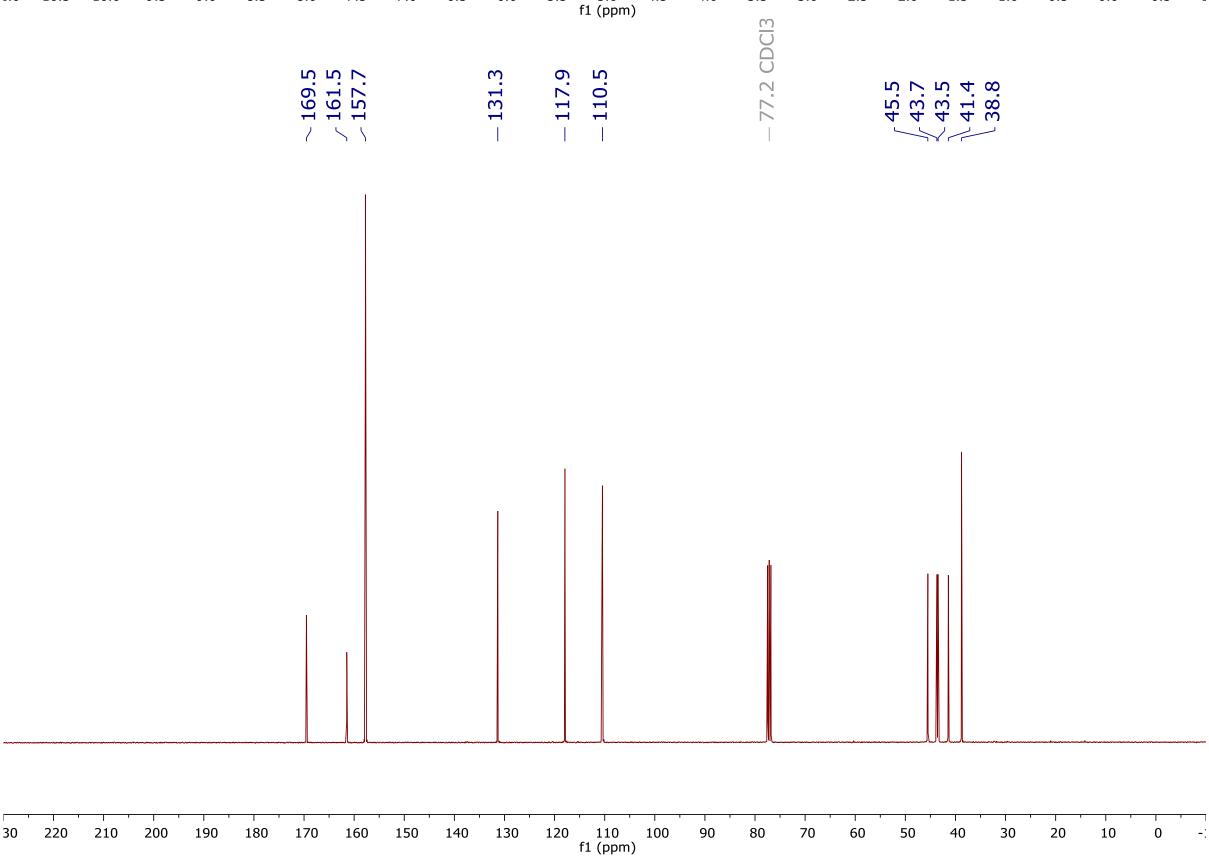

_N_,_N_-Dibutyl-2-(naphthalen-2-yl)acetamide (9a)

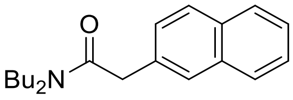

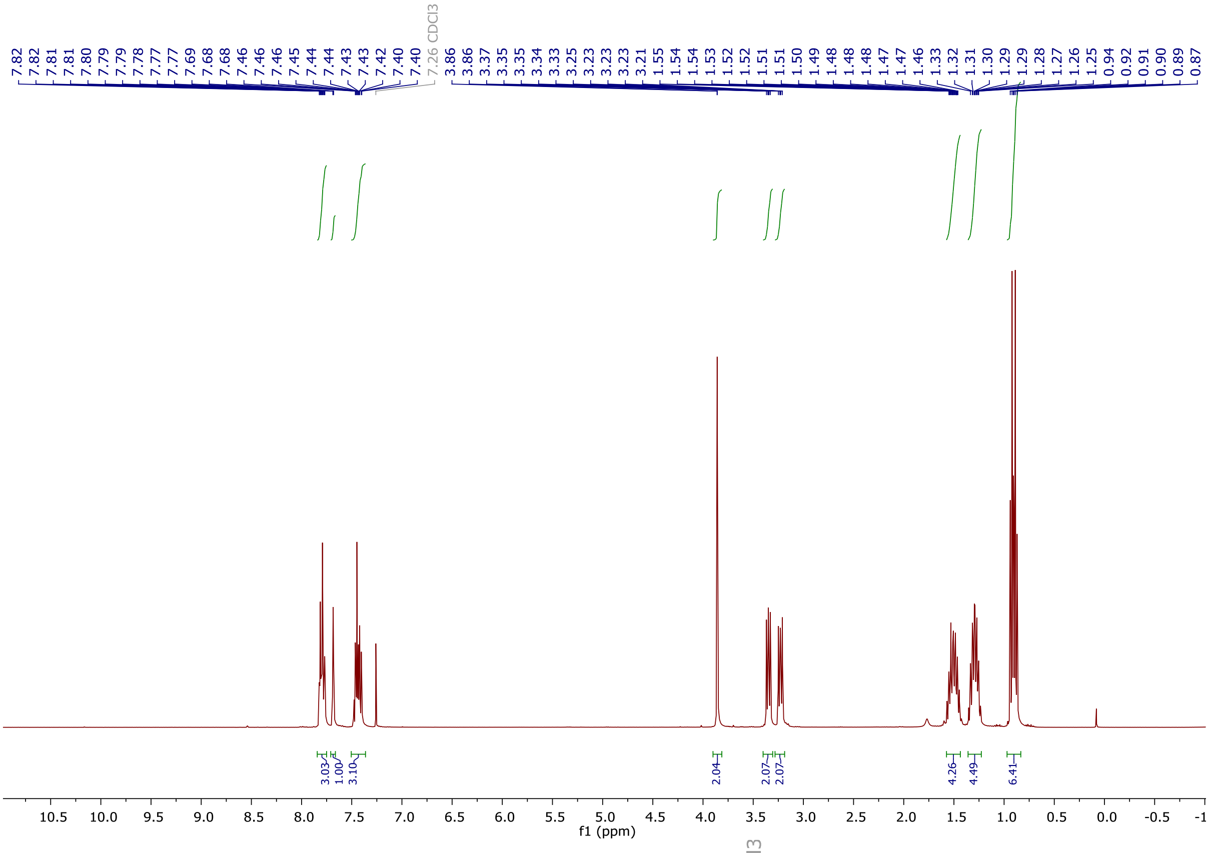

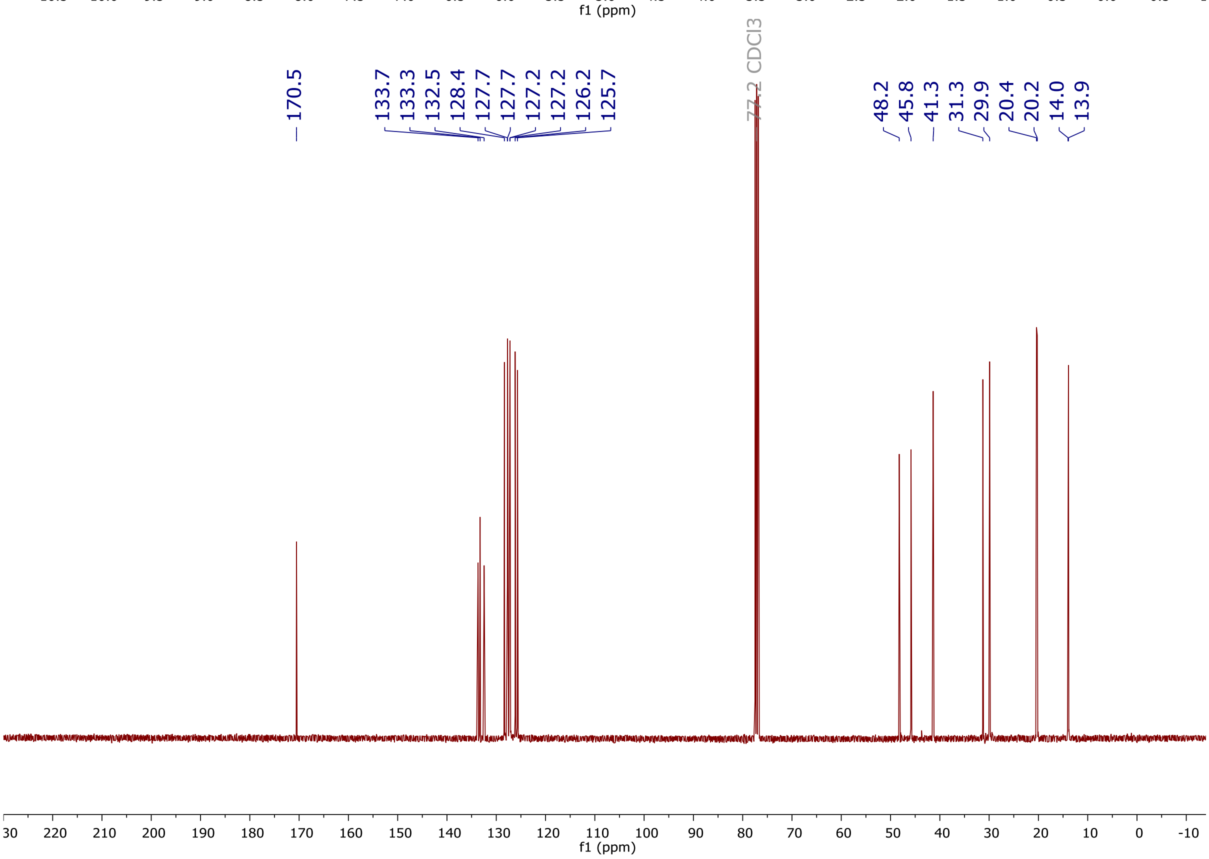

2-([1,1'-Biphenyl]-4-yl)-*N*,*N*-dibutylacetamide (9b)

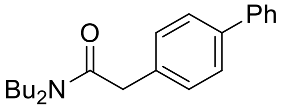

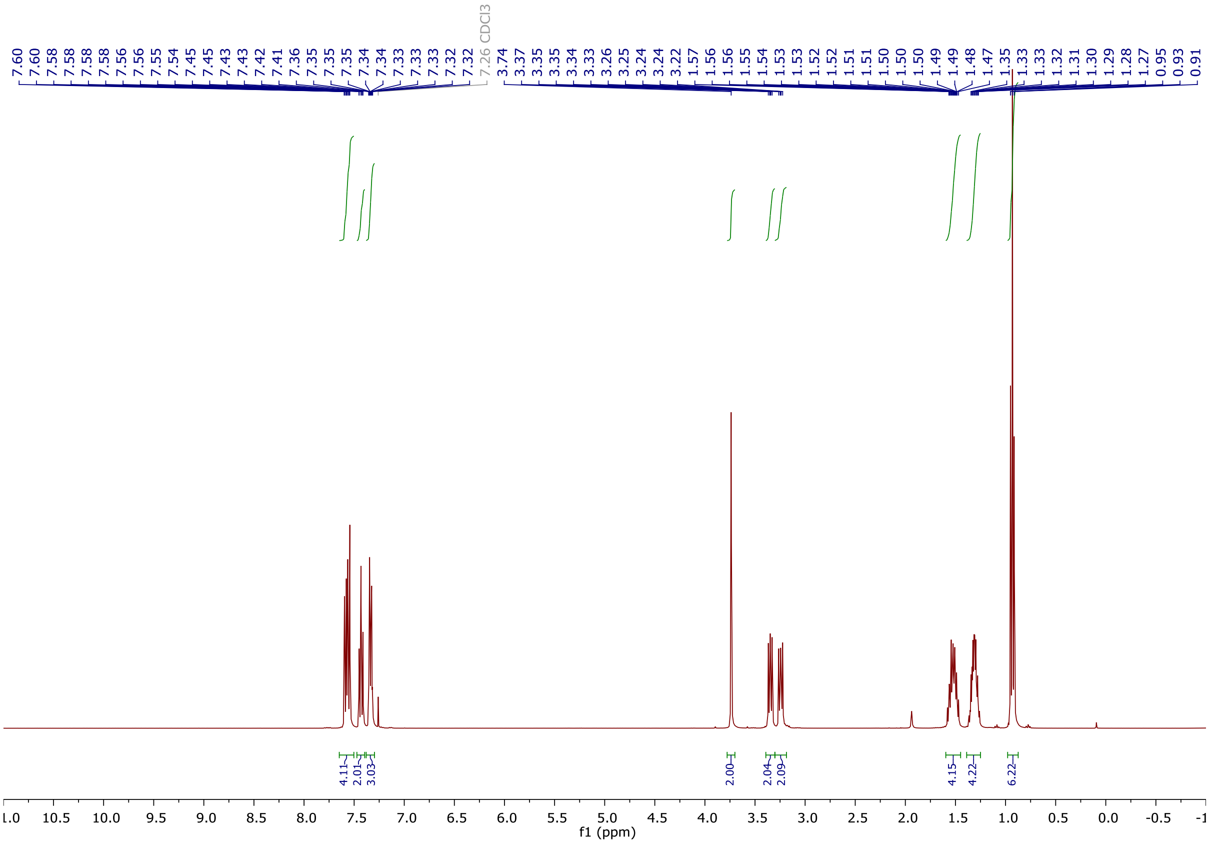

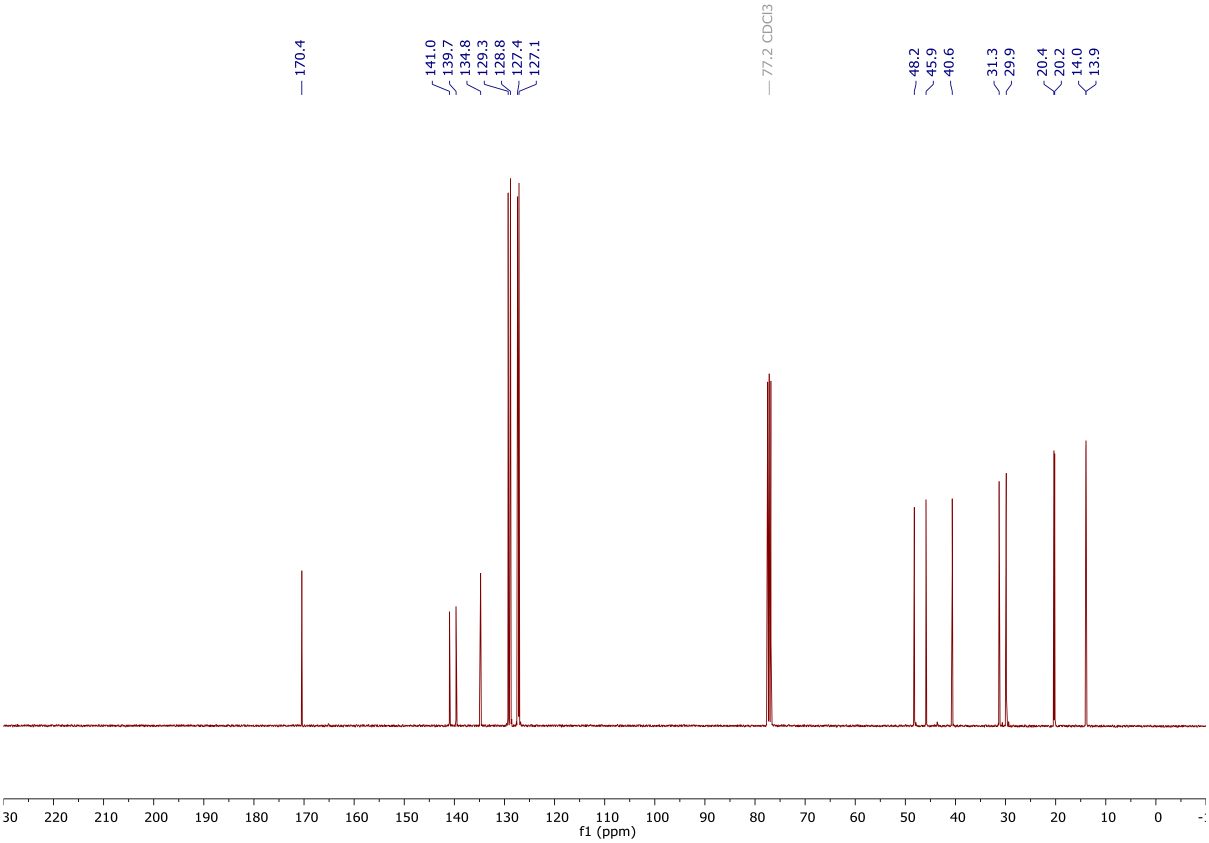

***N*,*N*-Dibutyl-2-(3-chlorophenyl)acetamide (9c)**

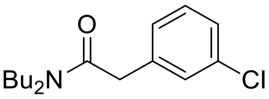

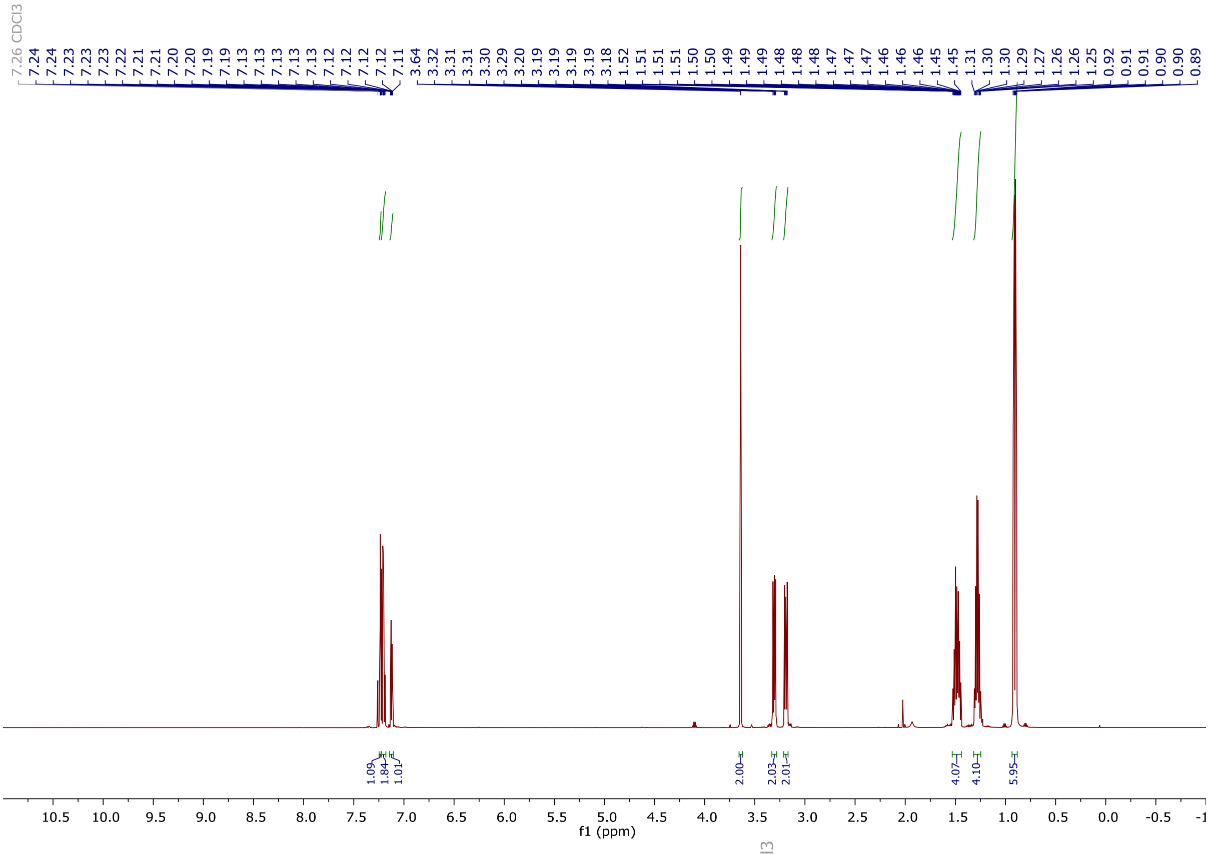

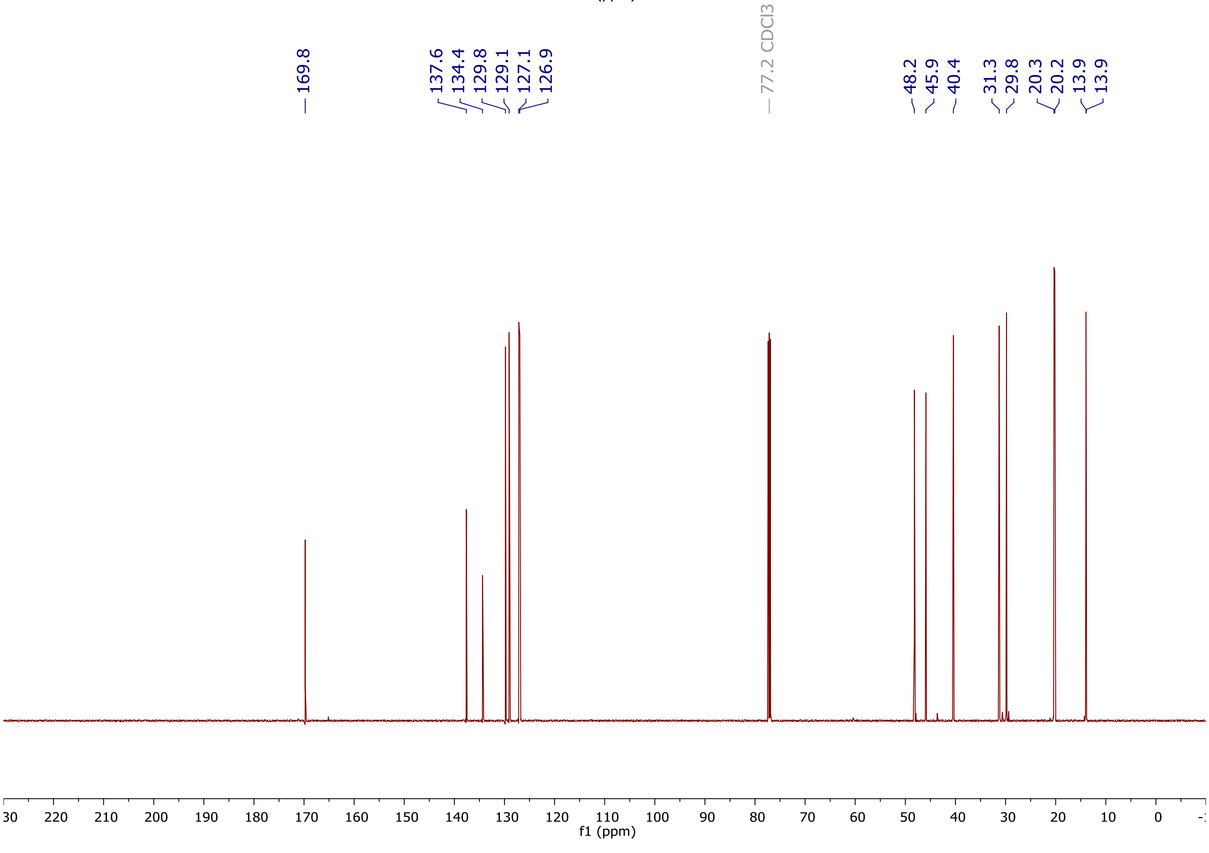

***N*,*N*-Dibutyl-2-(3-cyanophenyl)acetamide (9d)**

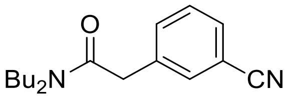

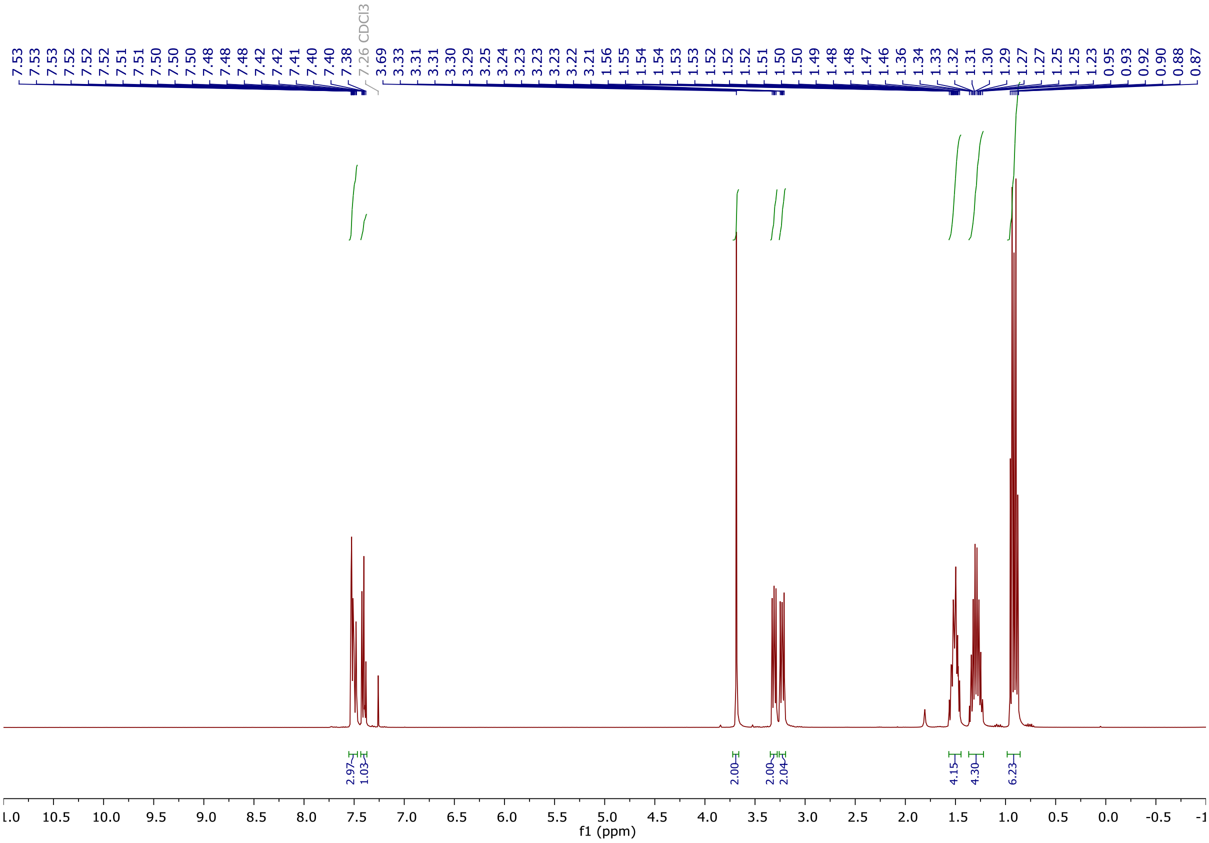

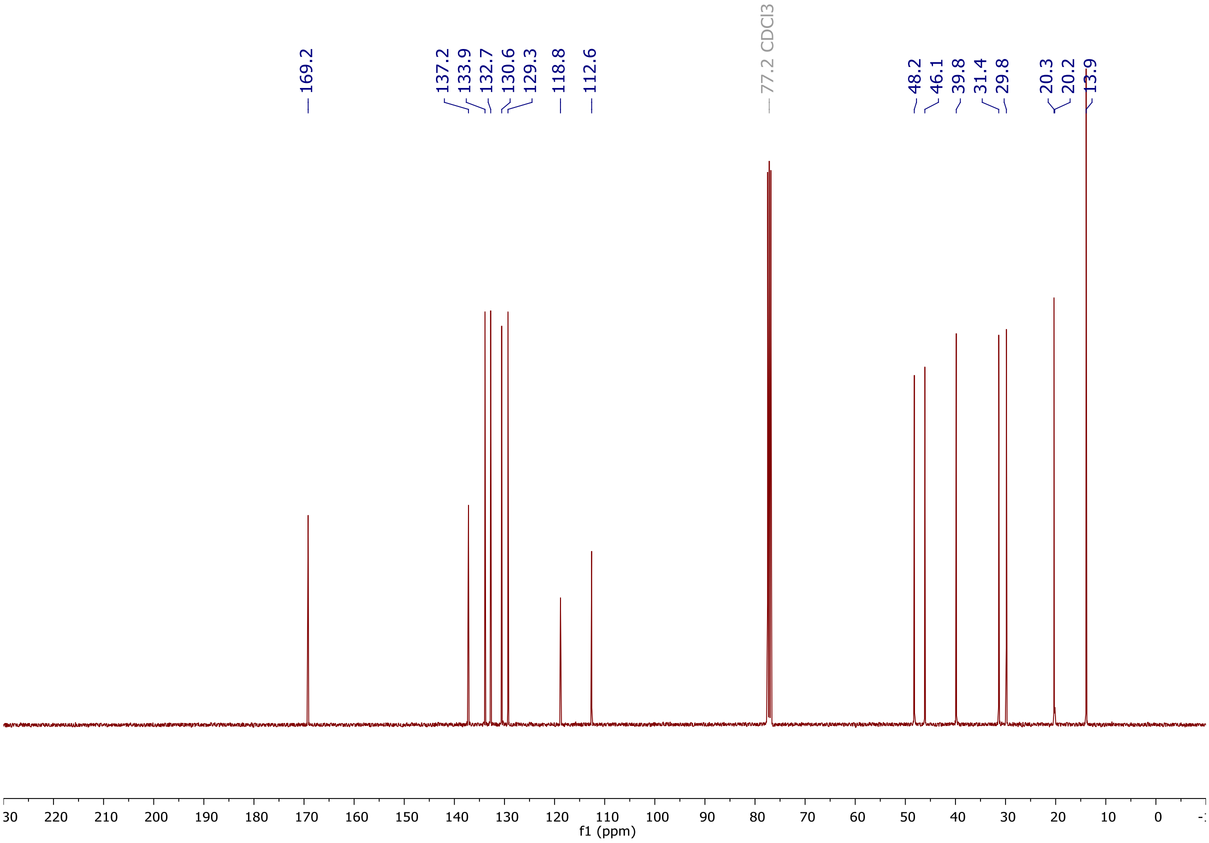

***N*,*N*-Dibutyl-2-(4-chlorophenyl)acetamide (9e)**

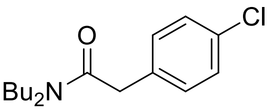

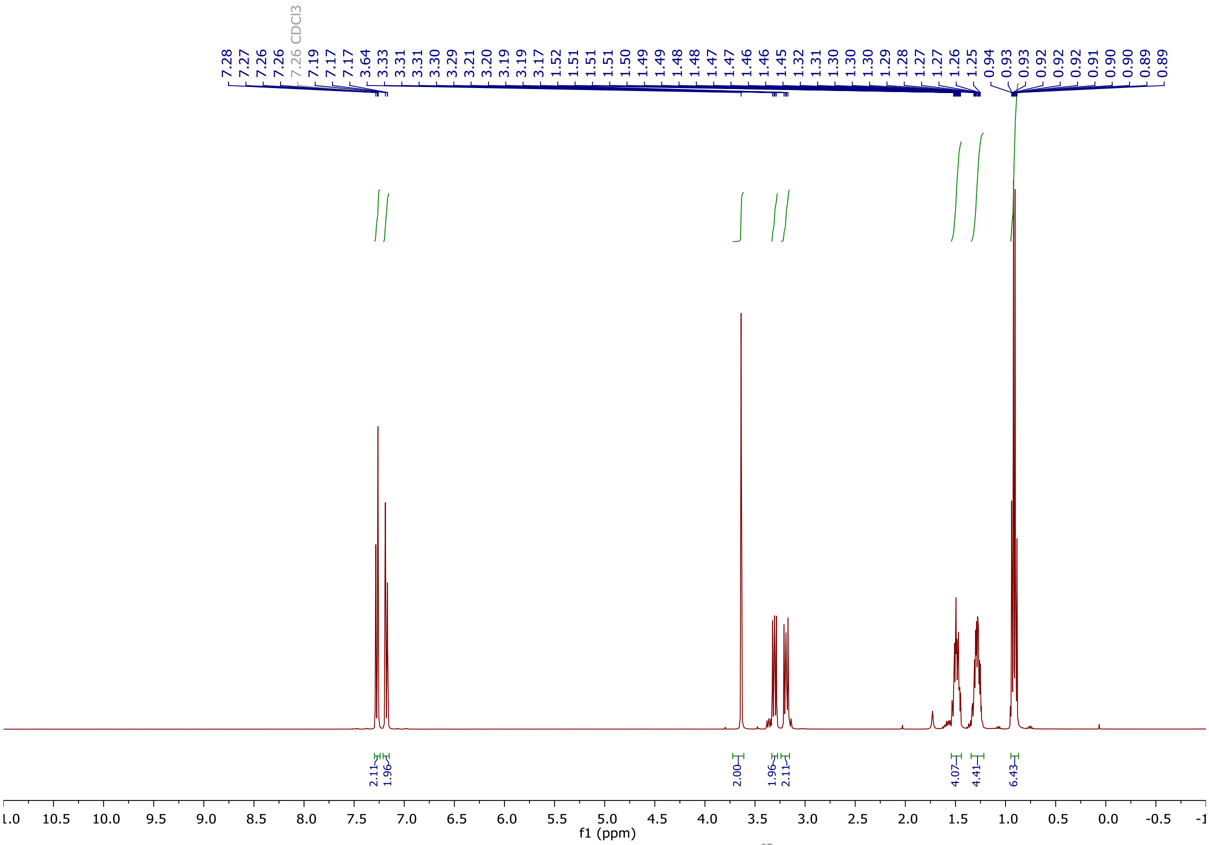

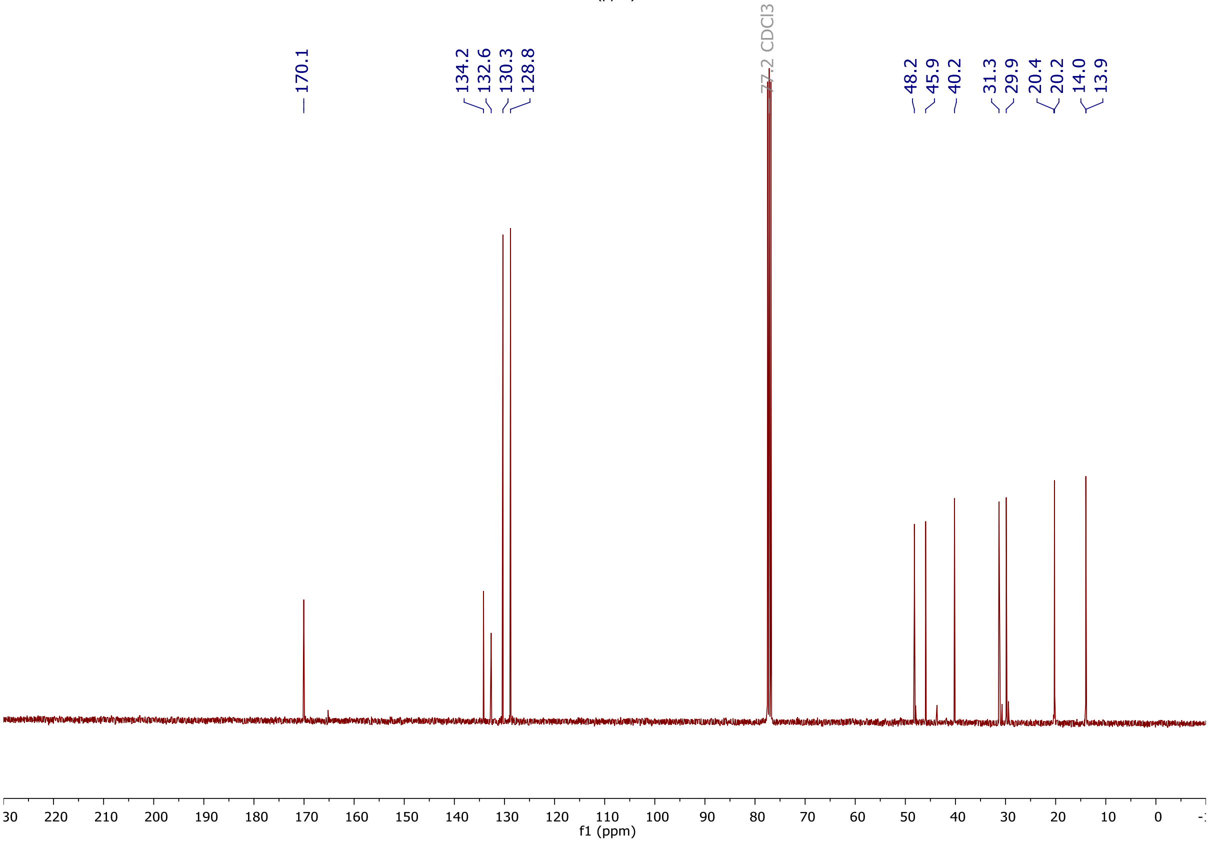

***N*,*N*-Dibutyl-2-(4-(trifluoromethyl)phenyl)acetamide (9f)**

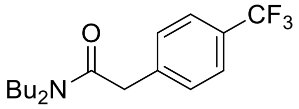

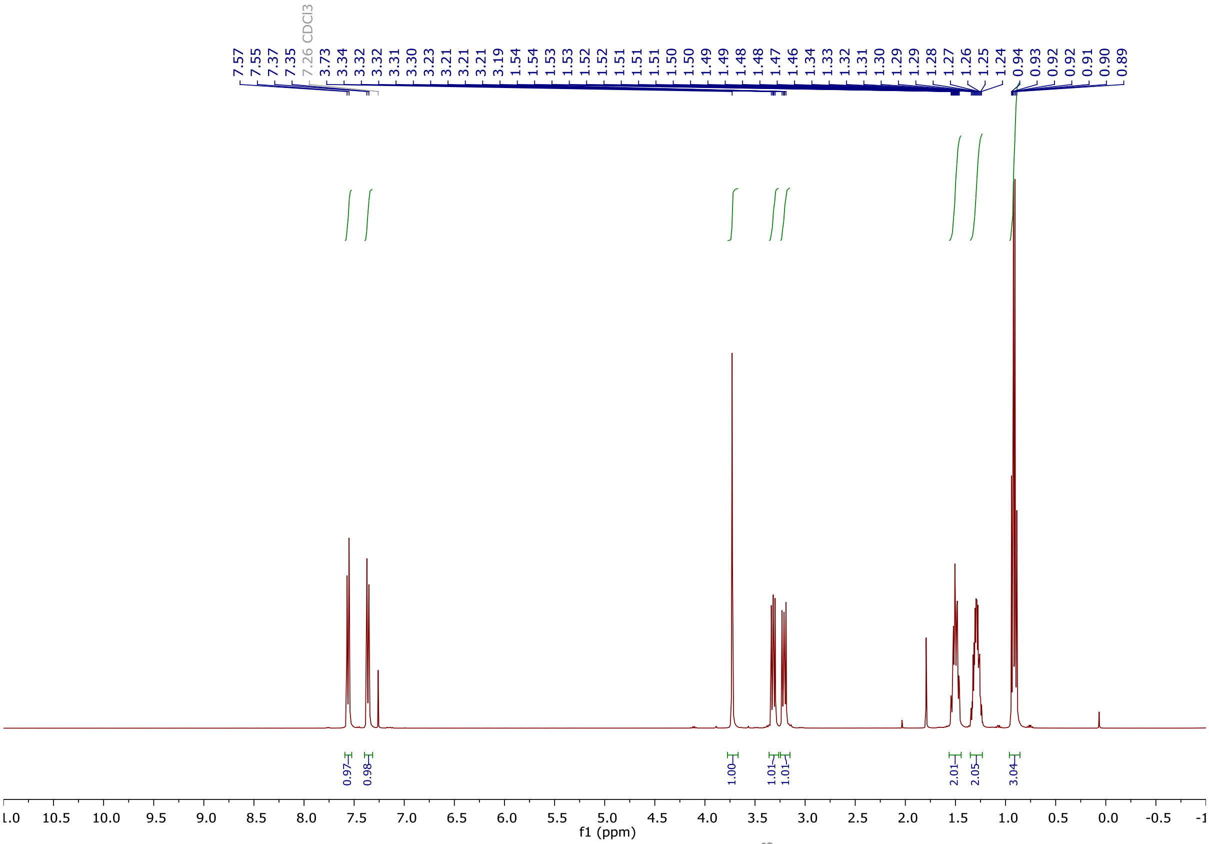

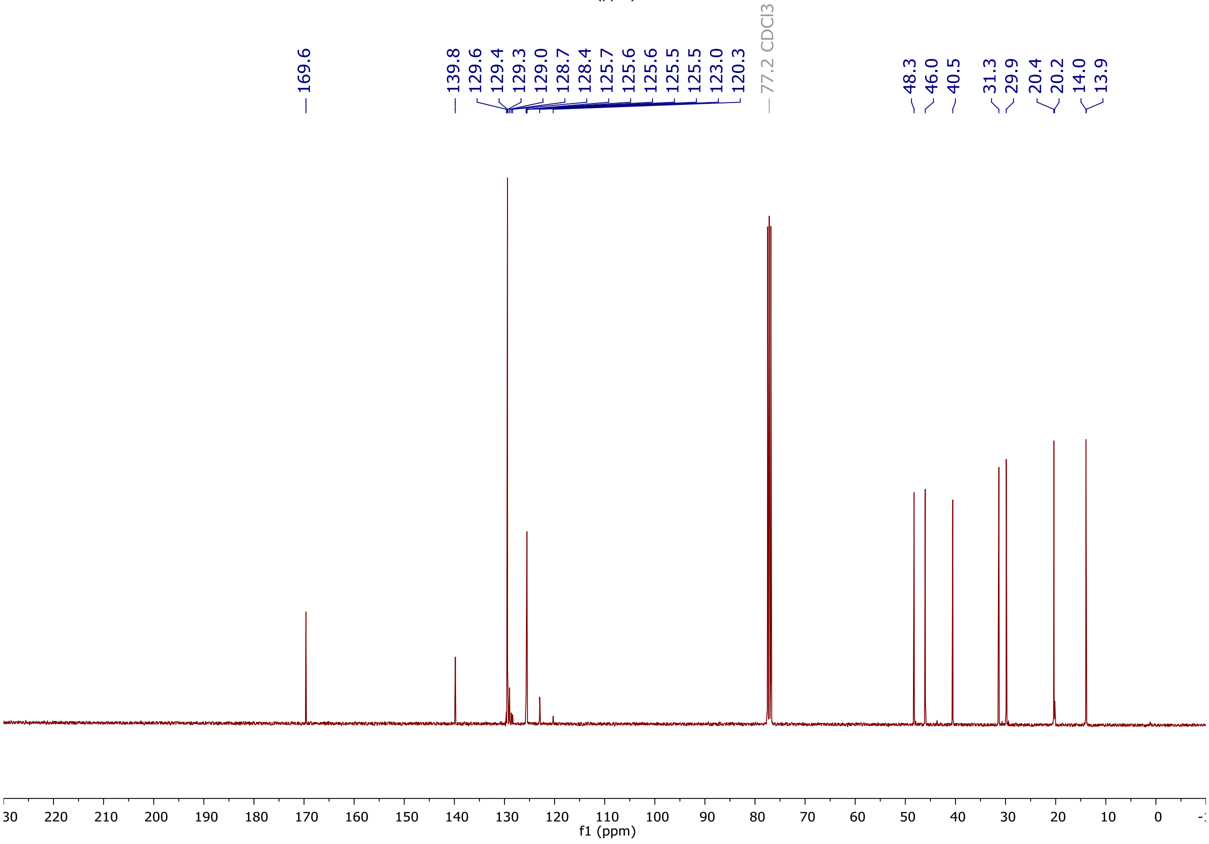

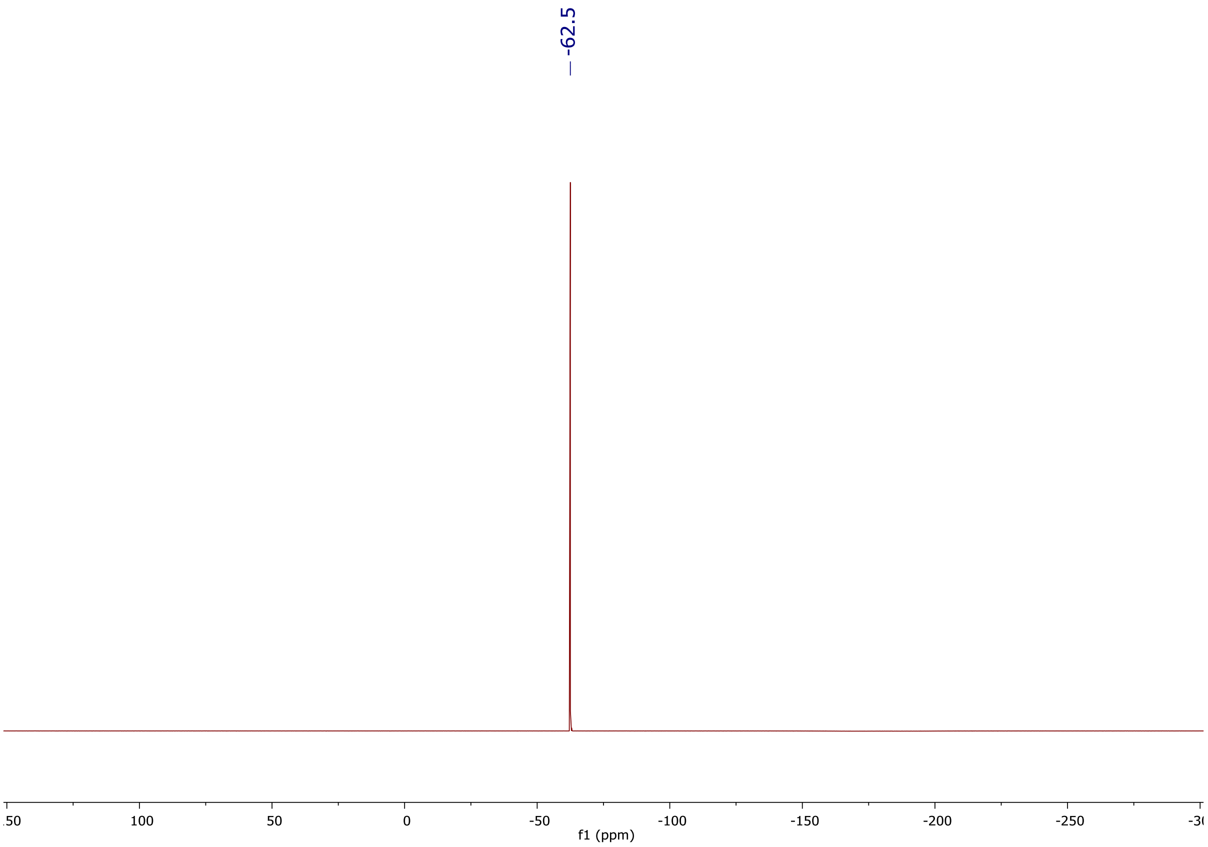
-62.5
50
100
50
0
-50
-100
-150
-200
-250
-3(
f1 (ppm)

2-(2-Bromophenyl)-*N*,*N*-dibutylacetamide (9g)

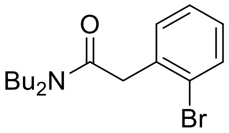

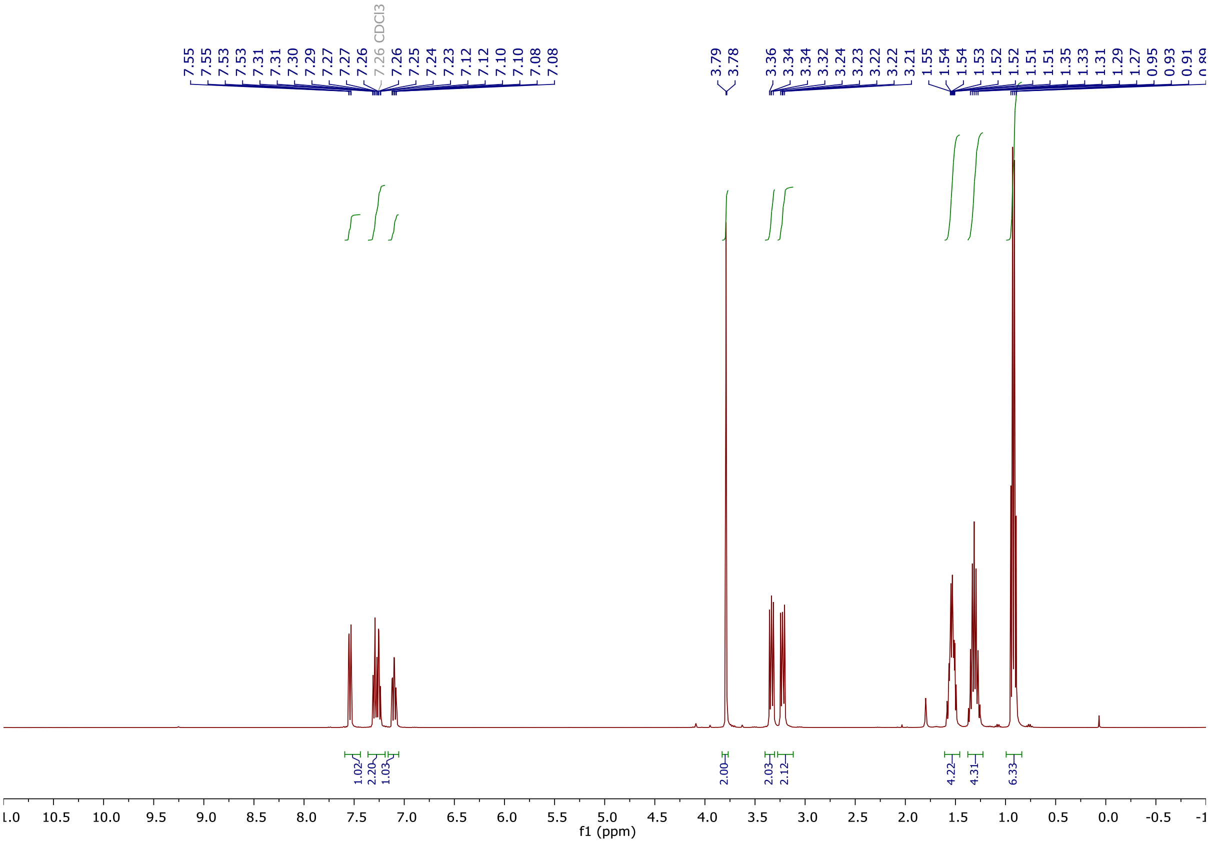

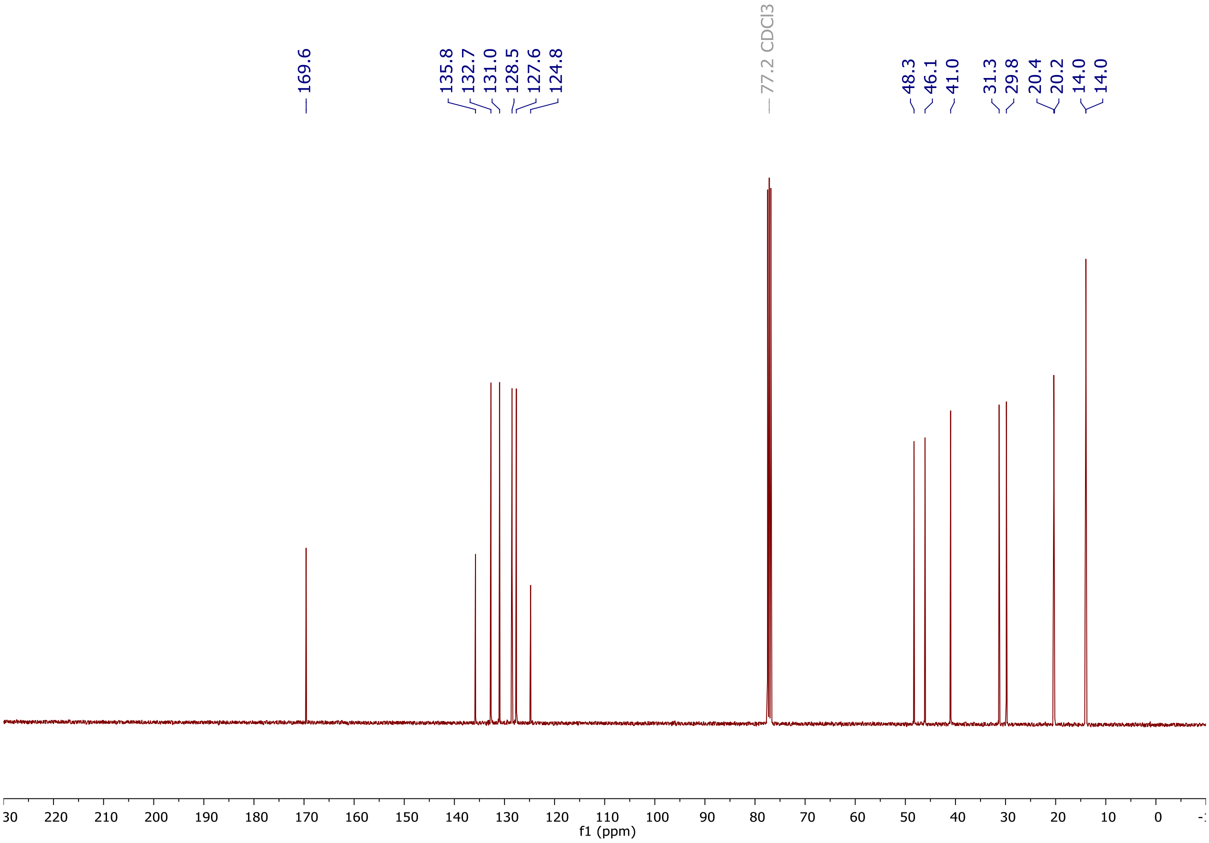

1-Morpholino-2-(naphthalen-2-yl)ethanone (9h)

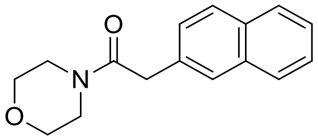

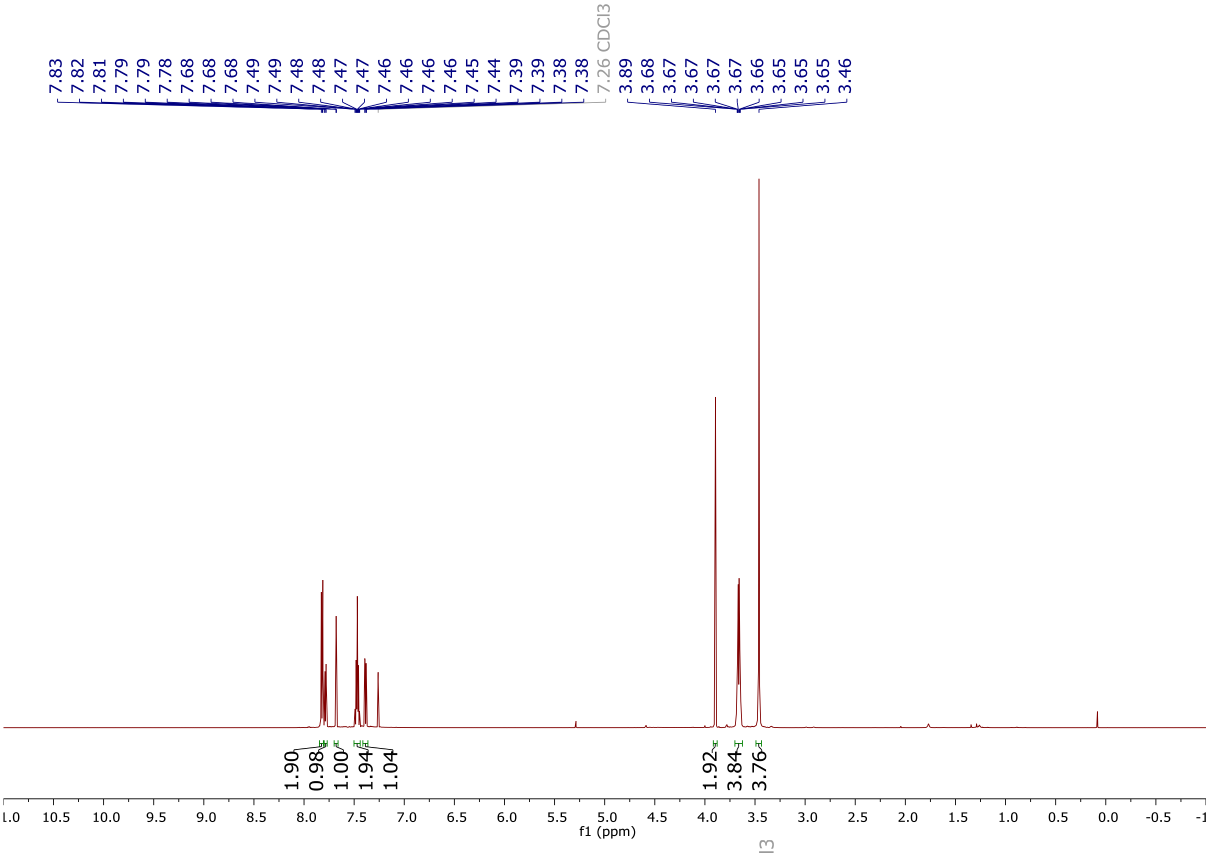

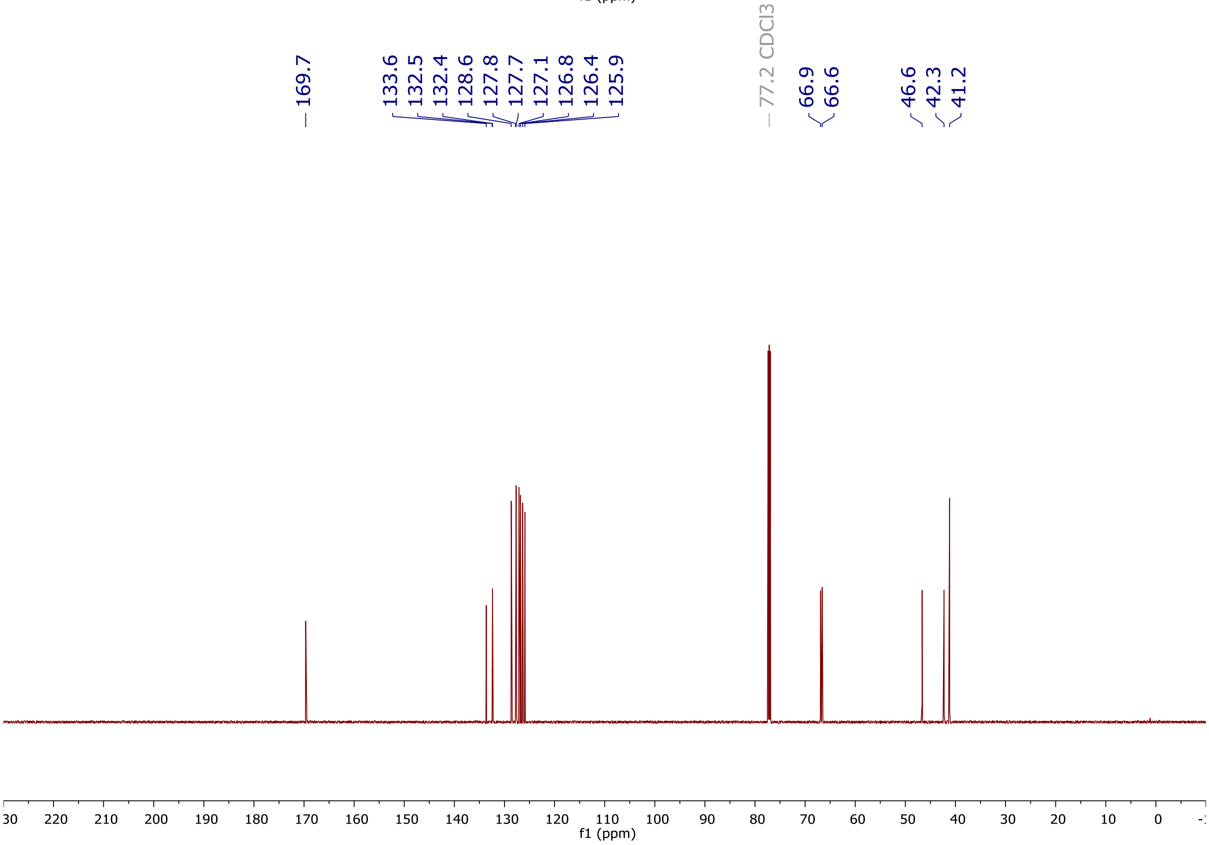

2-(Naphthalen-2-yl)-1-(4-(3-(trifluoromethyl)pyridin-2-yl)piperazin-1-yl)ethanone (9i)

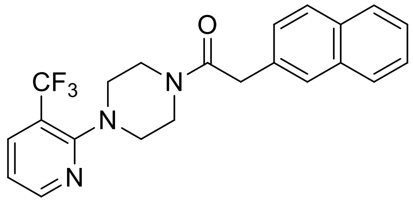

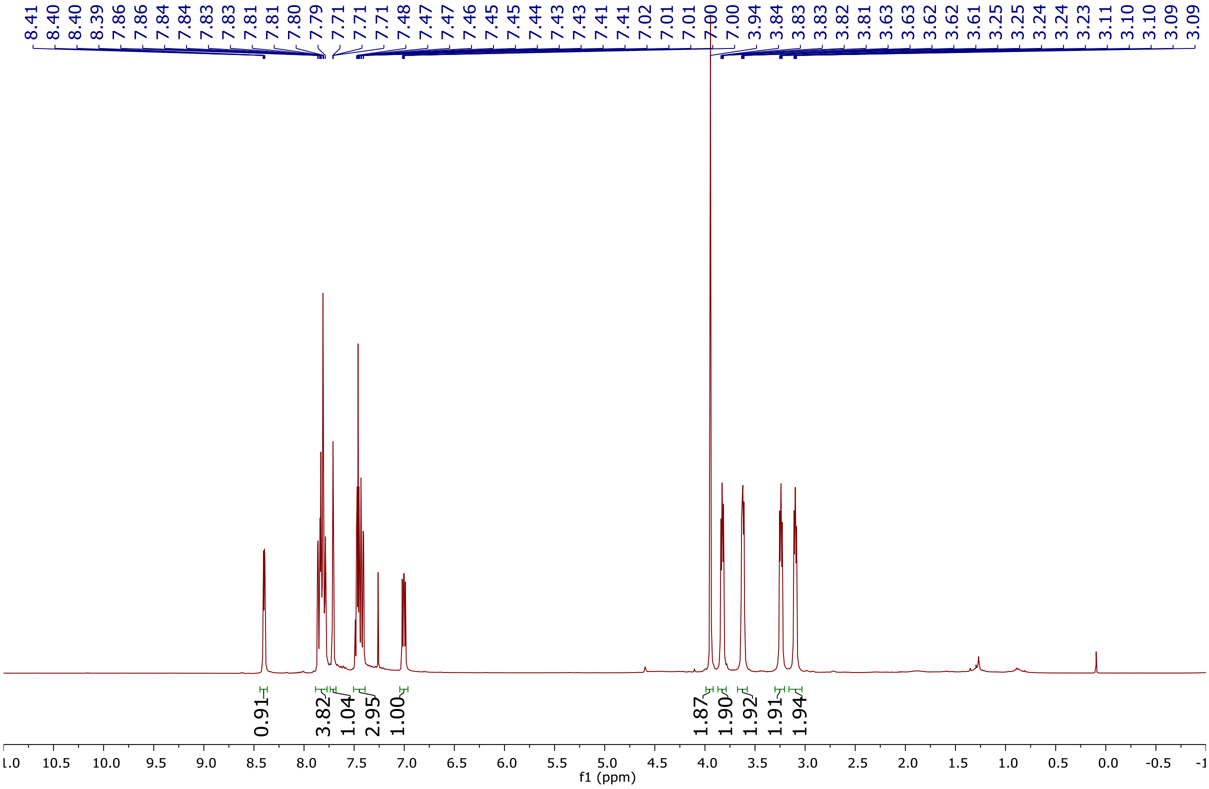

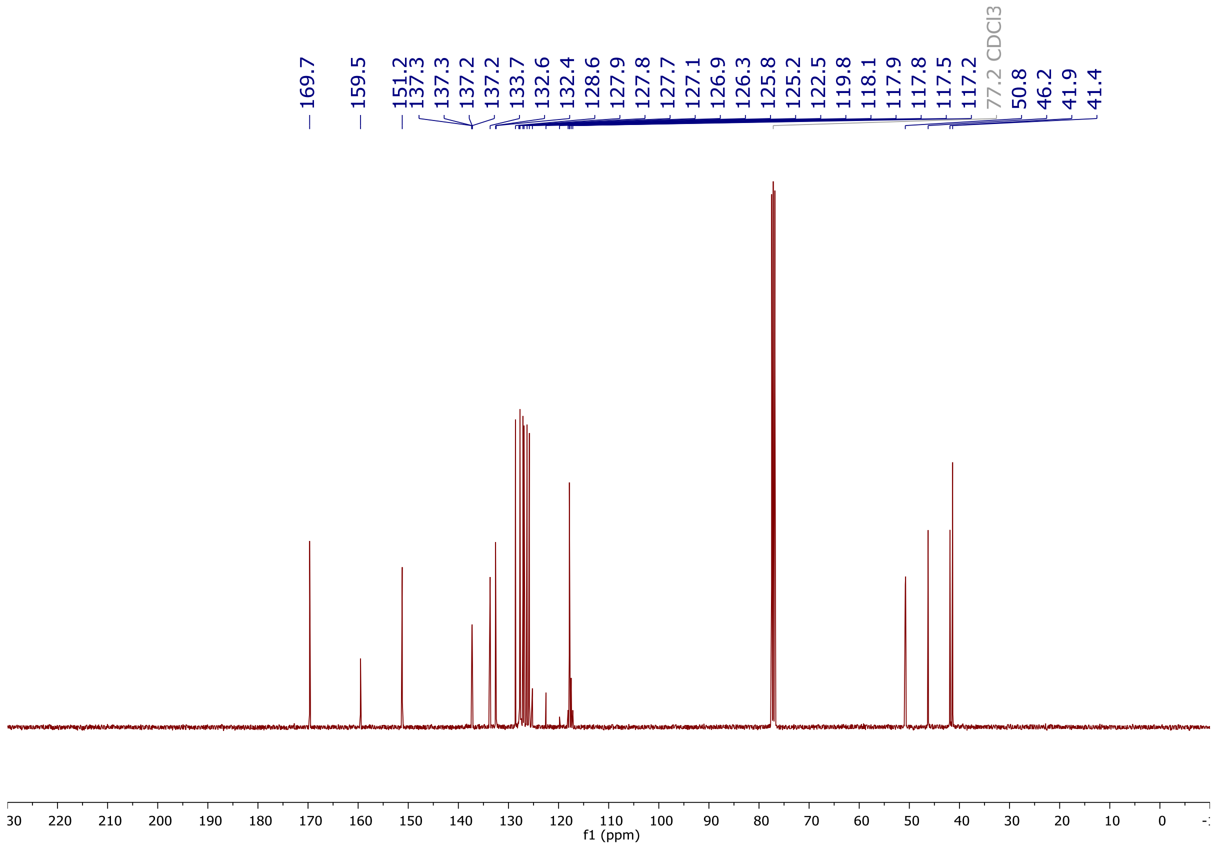
169.7
159.5
151.2
137.3
137.3
137.2
137.2
133.7
132.6
132.4
128.6
127.9
127.8
127.7
127.1
126.9
126.3
125.8
125.2
122.5
119.8
118.1
117.9
117.8
117.5
117.2
77.2 CDCl3
50.8
46.2
41.9
41.4
f1 (ppm)

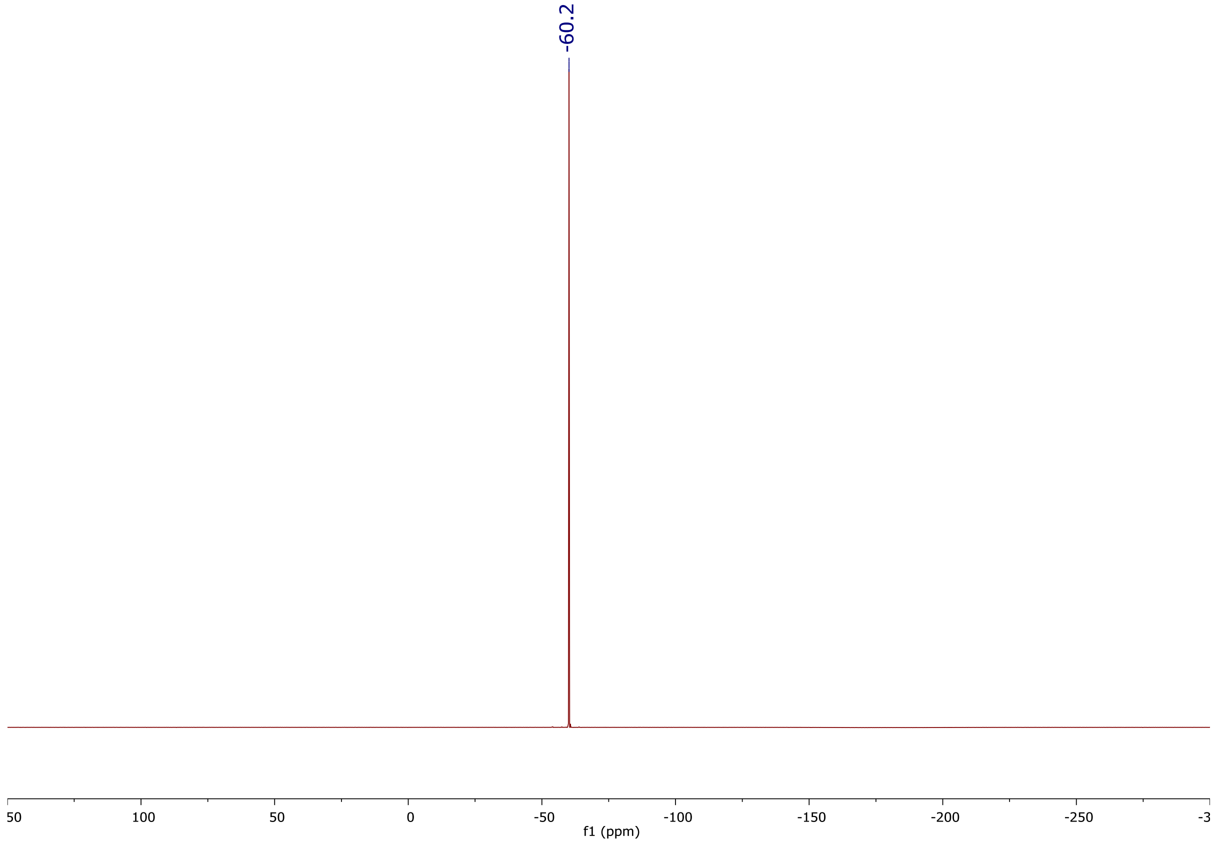
-60.2
f1 (ppm)

***N*,*N*-Dibutyl-2-hydroxy-2-phenylacetamide (10a)**

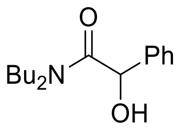

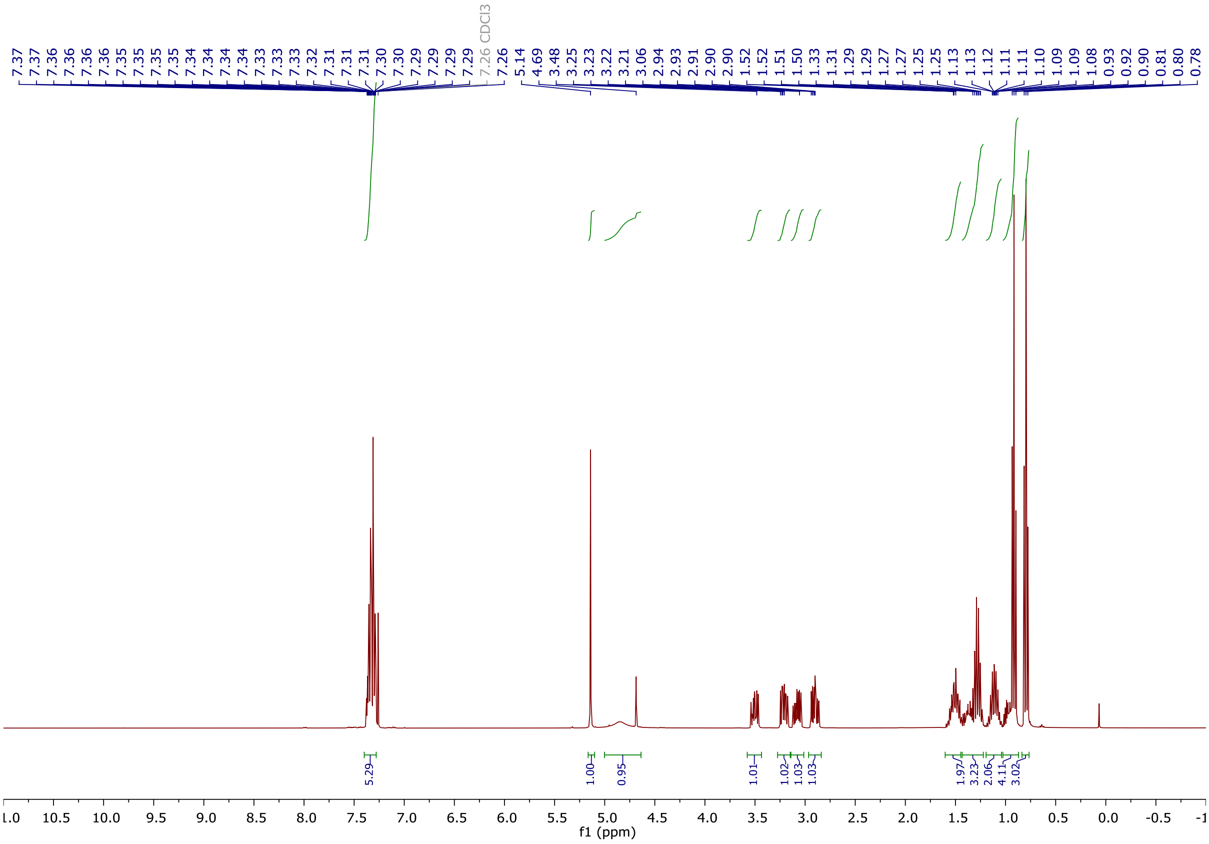

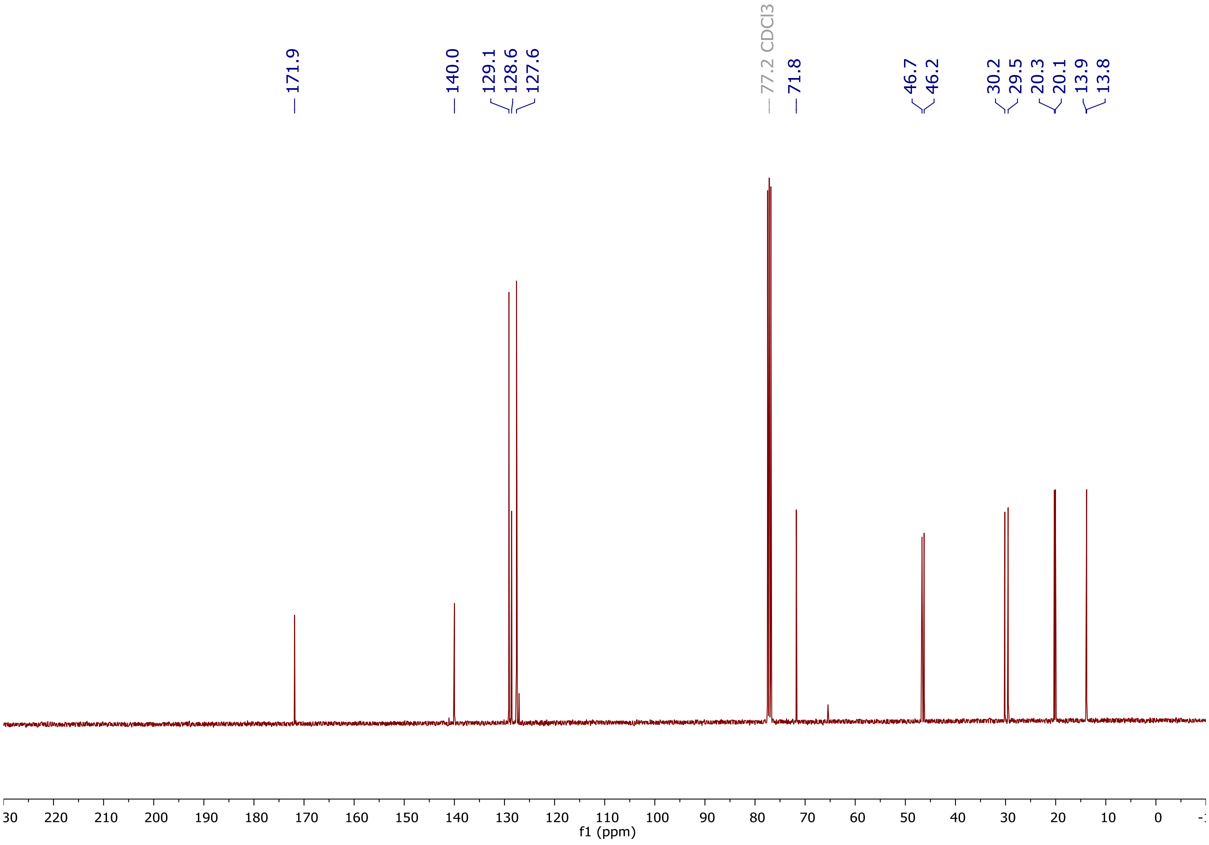

2-(2-Bromophenyl)-2-hydroxy-1-((4aR,8aS)-octahydroquinolin-2(1H)-yl)ethanone (10b)

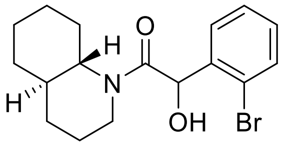

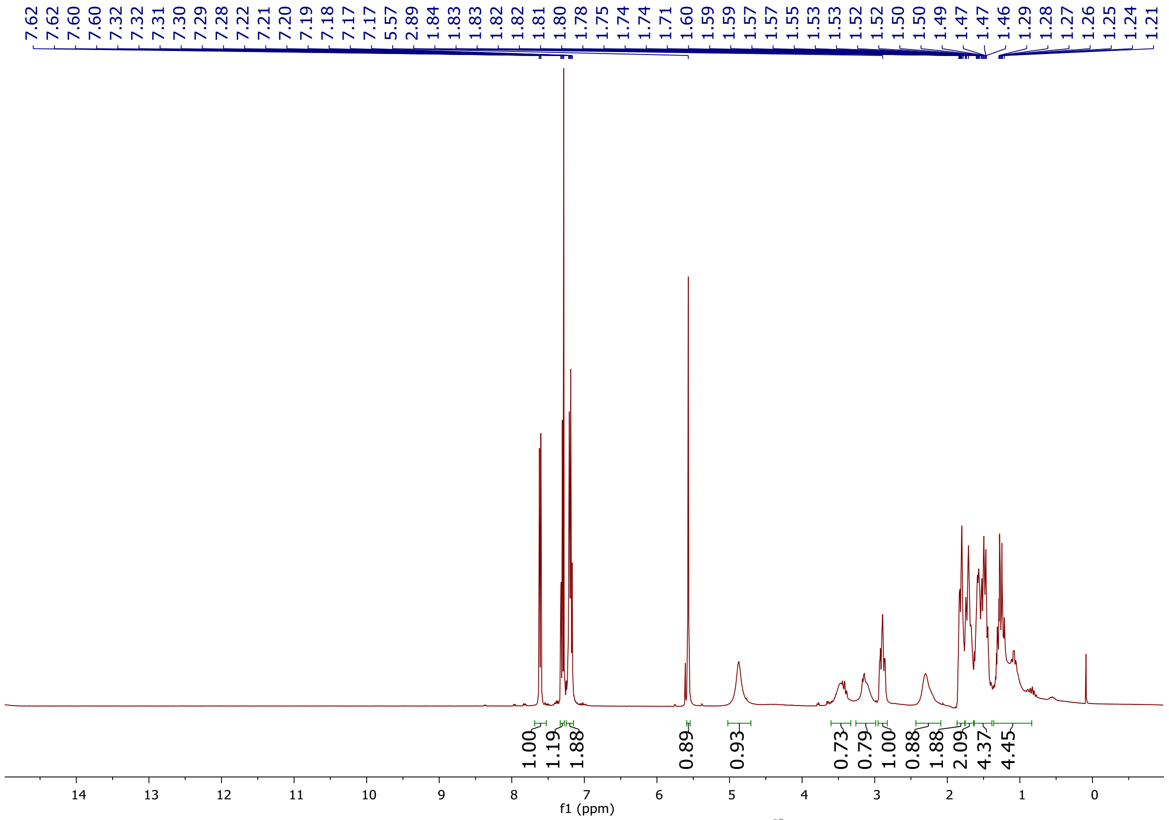

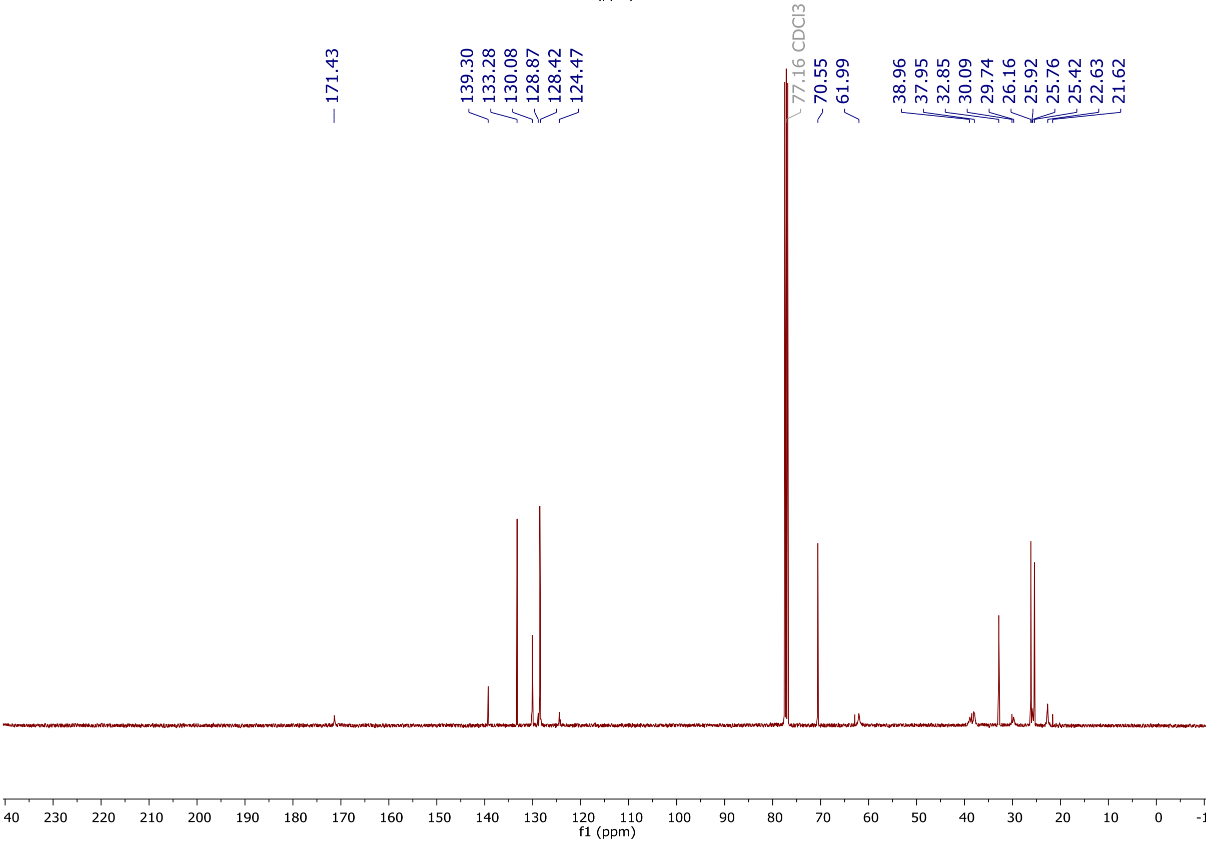

***N*,*N*-dibutyl-2-cyclopropyl-2-oxoacetamide (11a)**

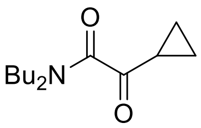

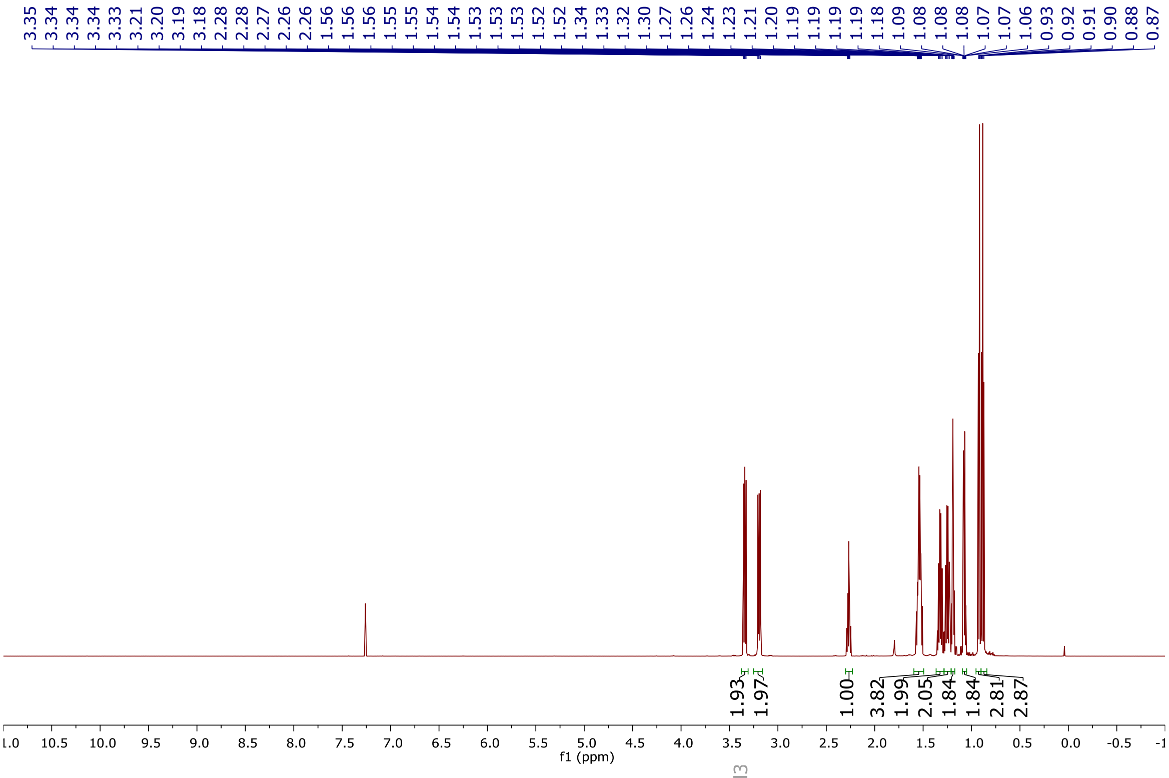

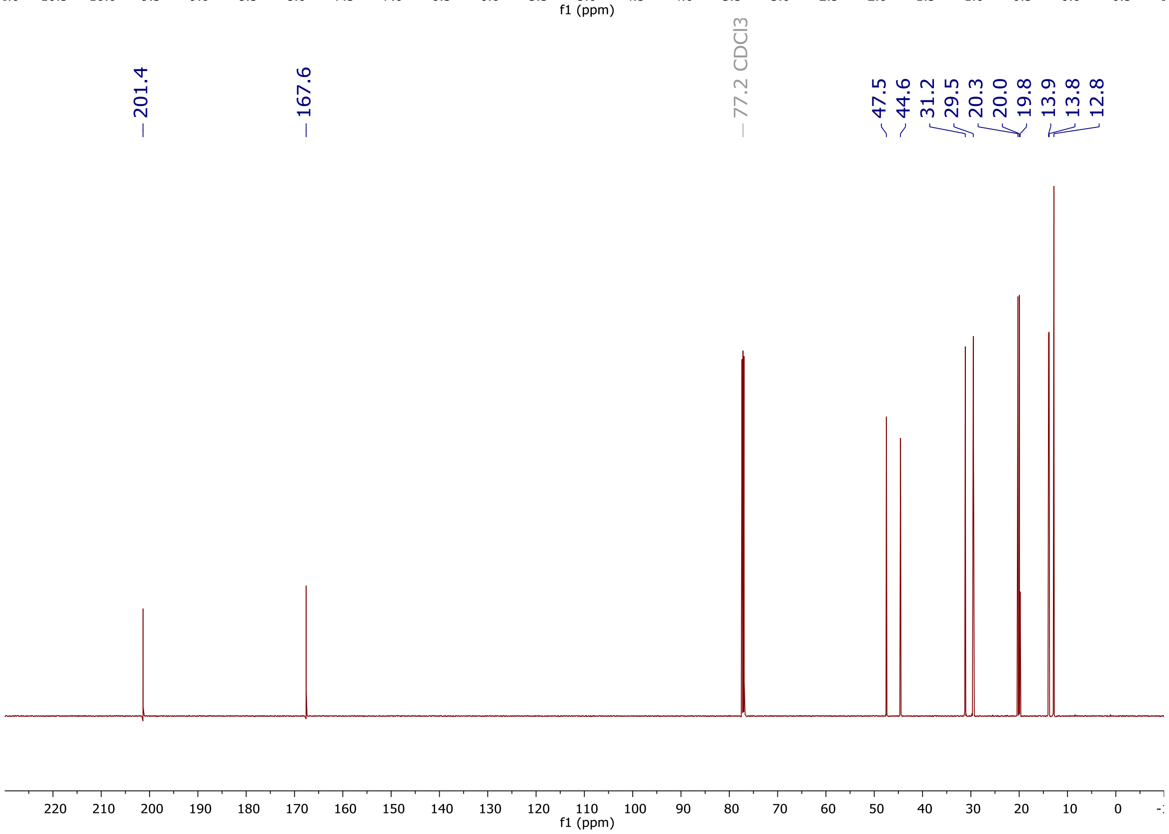

1-(4-Bromophenyl)-2-((4aR,8aS)-octahydroquinolin-2(1H)-yl)ethane-1,2-dione (11b)

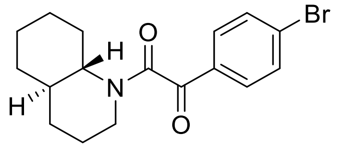

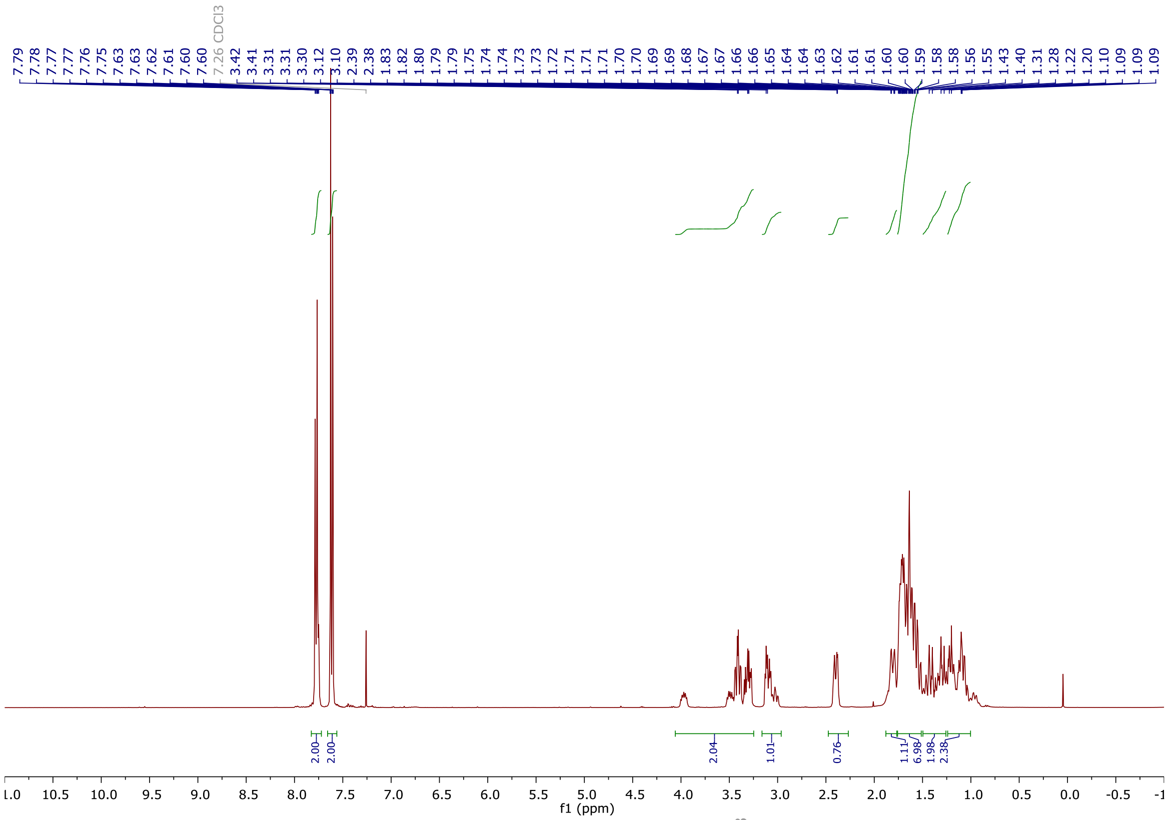

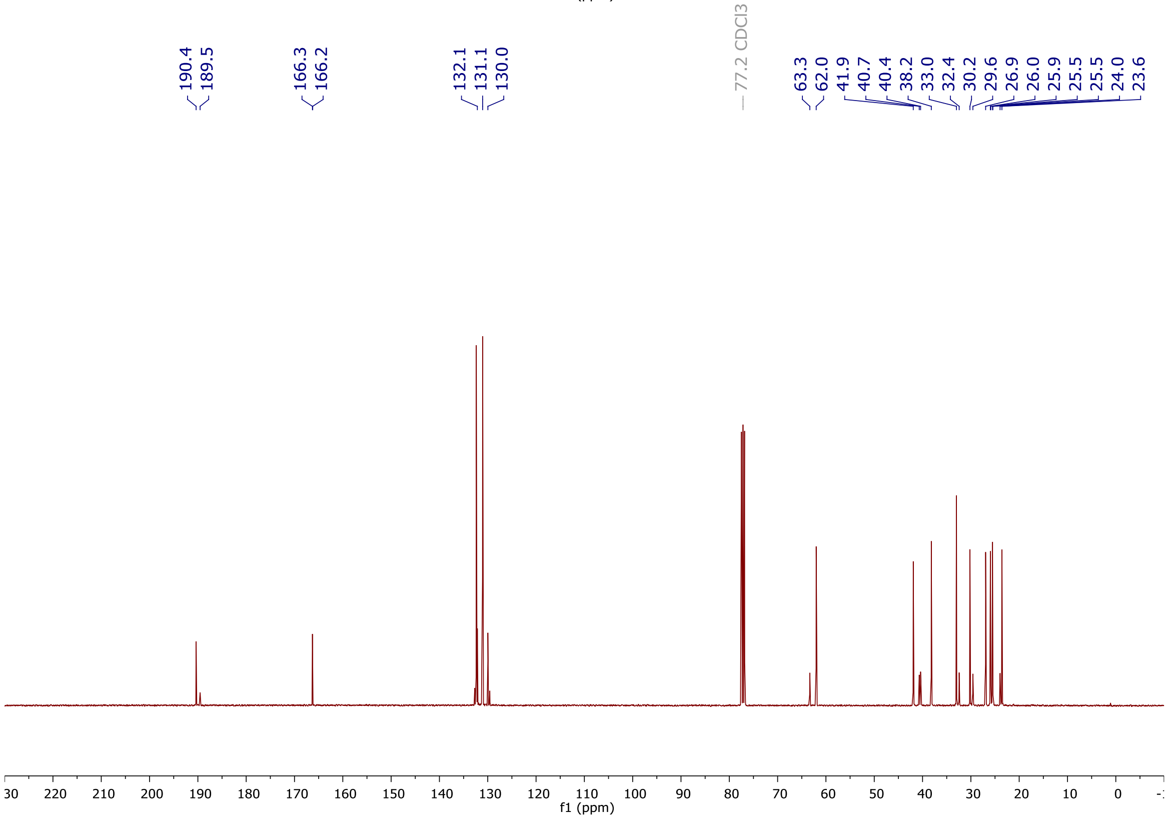

1-(4-Chlorophenyl)-2-((4aR,8aS)-octahydroquinolin-2(1H)-yl)ethane-1,2-dione (11c)

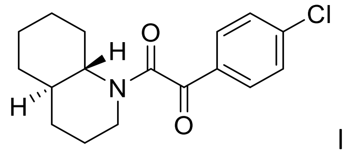

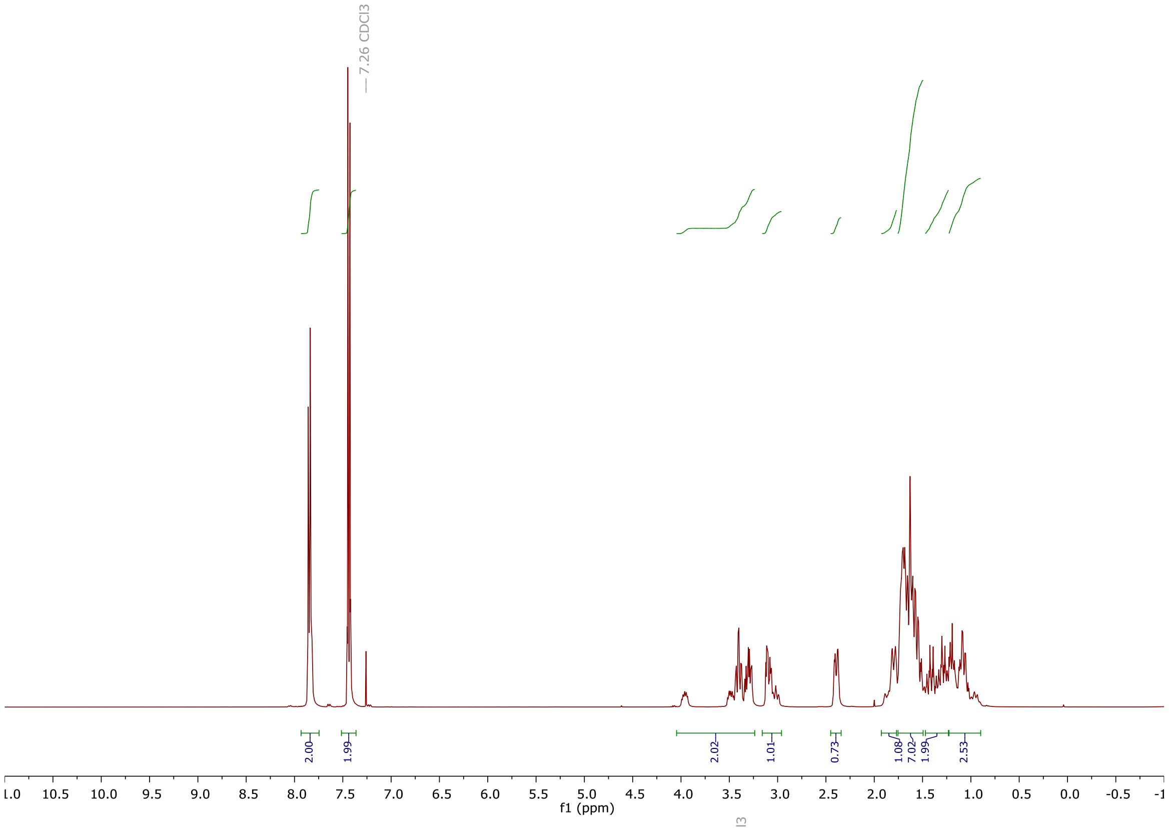

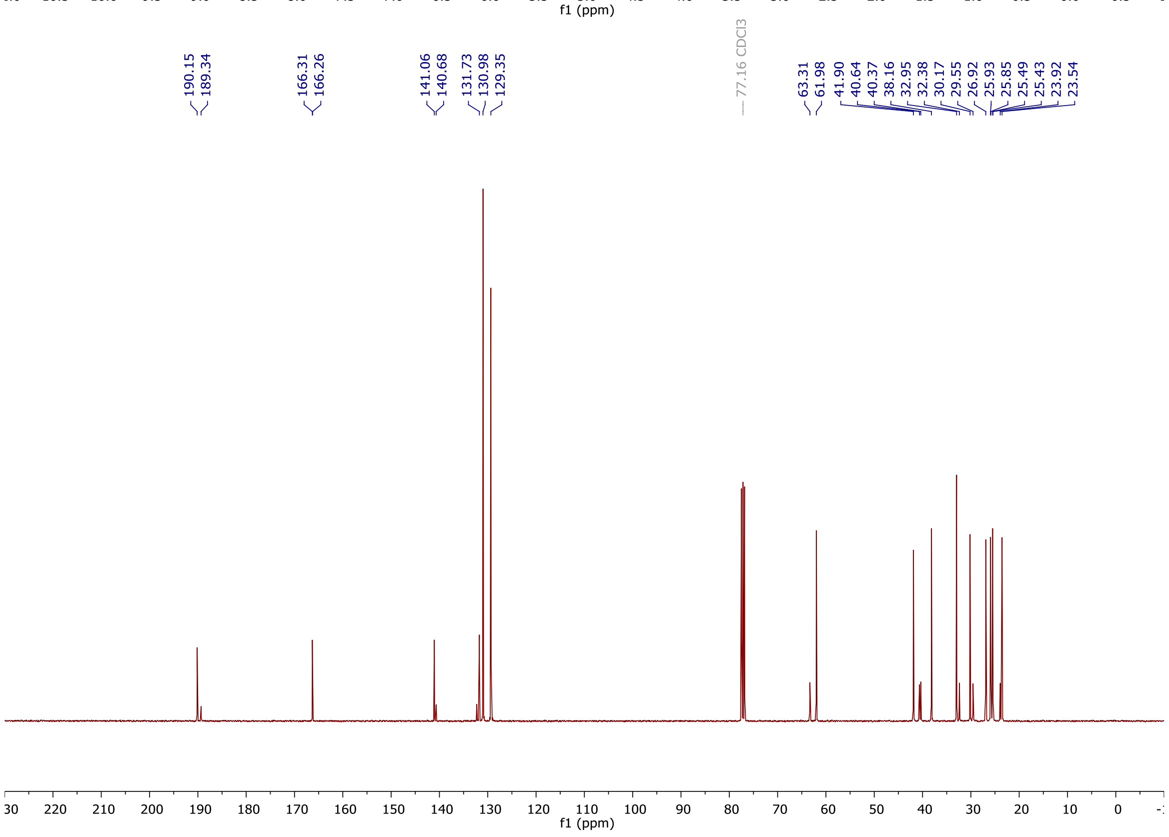

1-(2-Iodophenyl)-2-((4aR,8aS)-octahydroquinolin-2(1H)-yl)ethane-1,2-dione (11d)

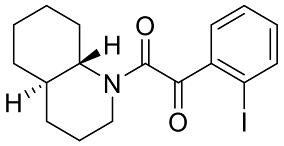

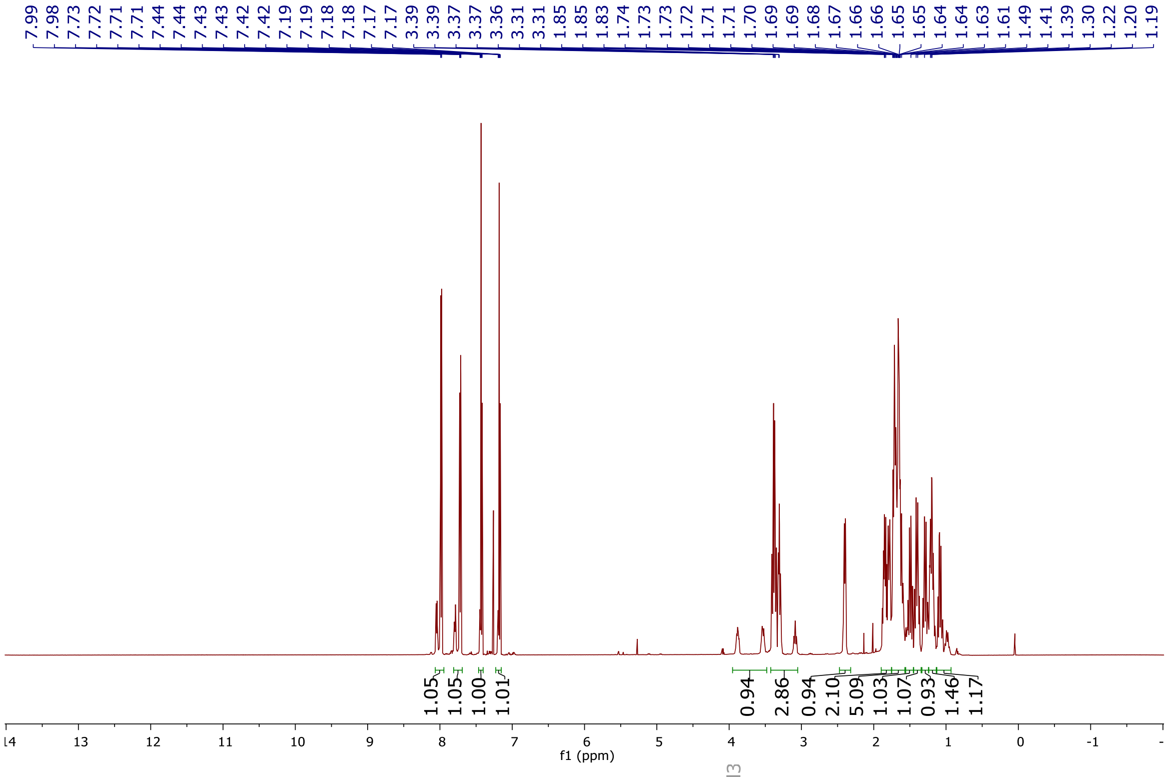

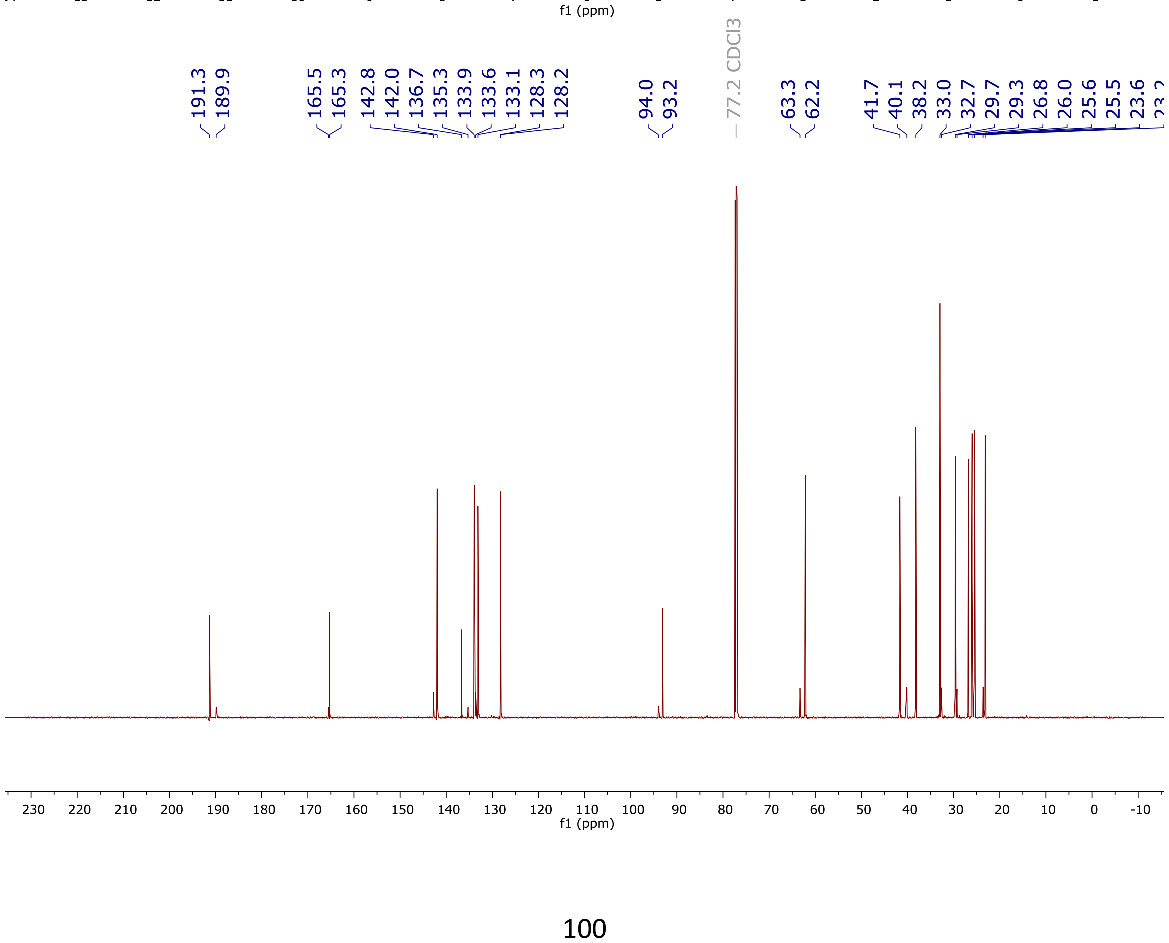

***N*,*N*-Dibutyl-1-(diphenylphosphoryl)formamide (11e)**

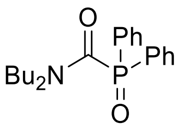

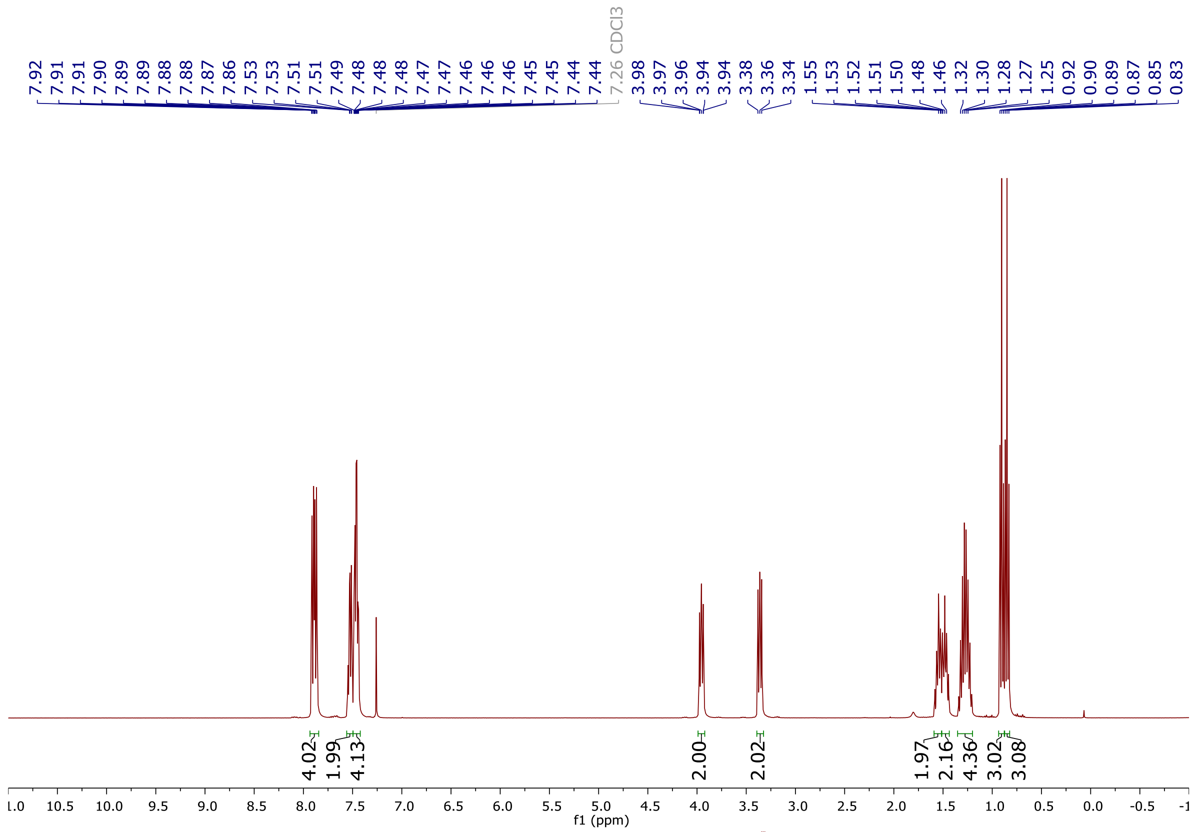

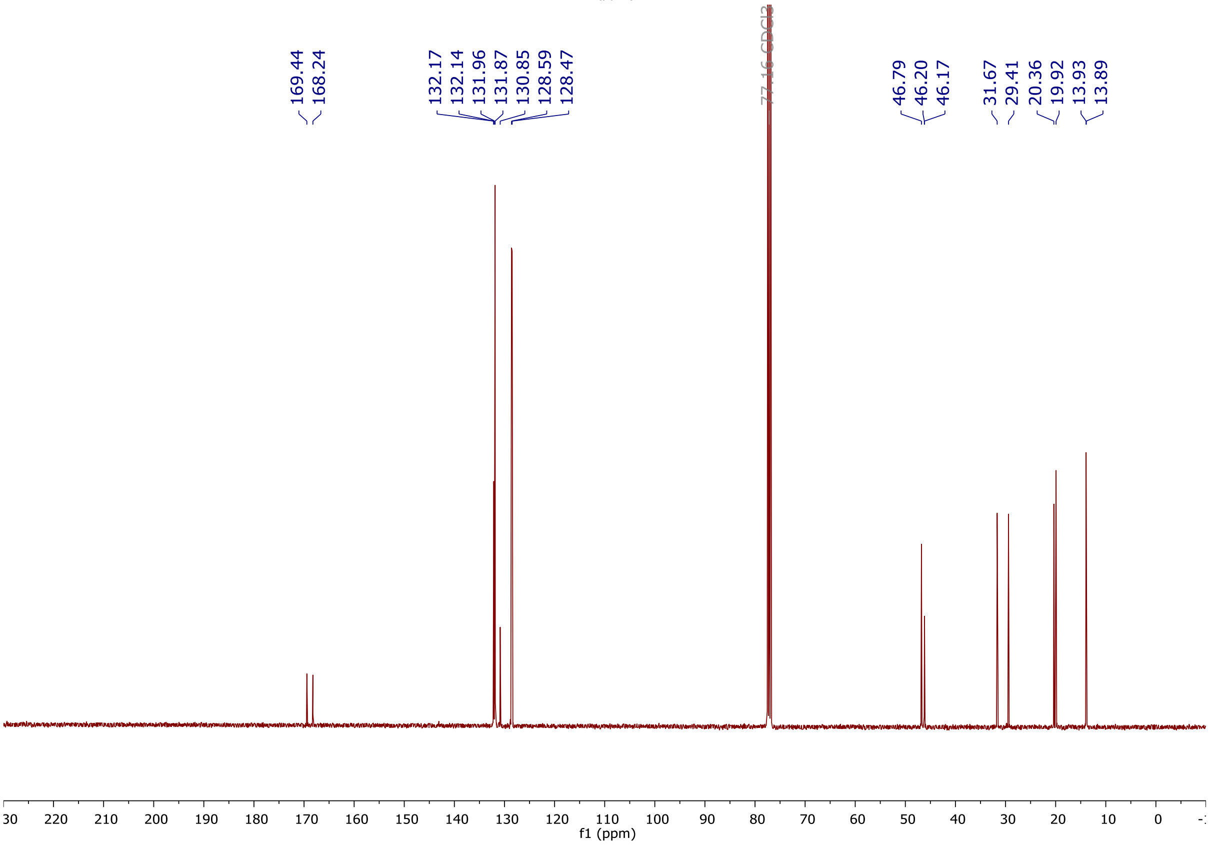

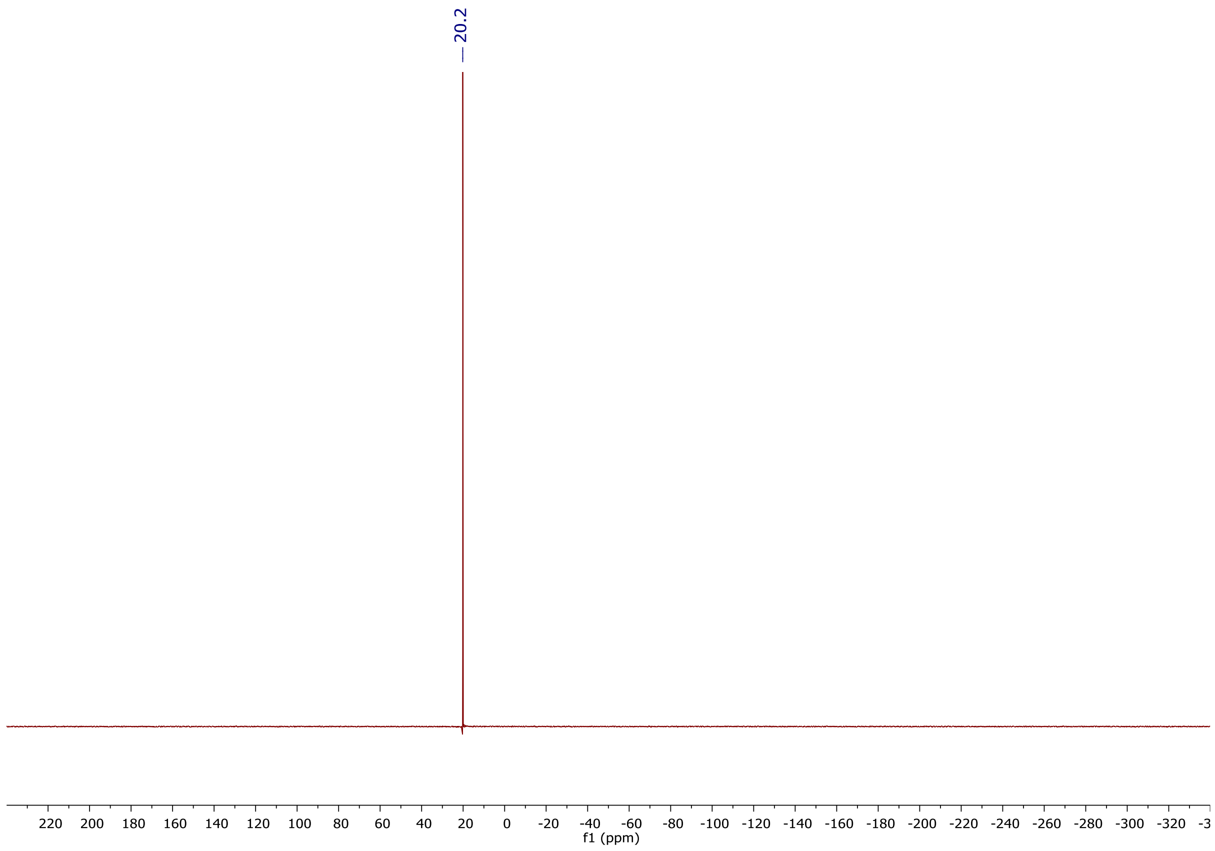
20.2
220 200 180 160 140 120 100 80 60 40 20 0 -20 -40 -60 -80 -100 -120 -140 -160 -180 -200 -220 -240 -260 -280 -300 -320 -3
f1 (ppm)

***N*,*N*-Dibutyl-3-oxocyclohexanecarboxamide (12a)**

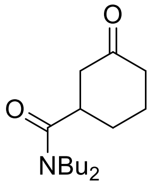

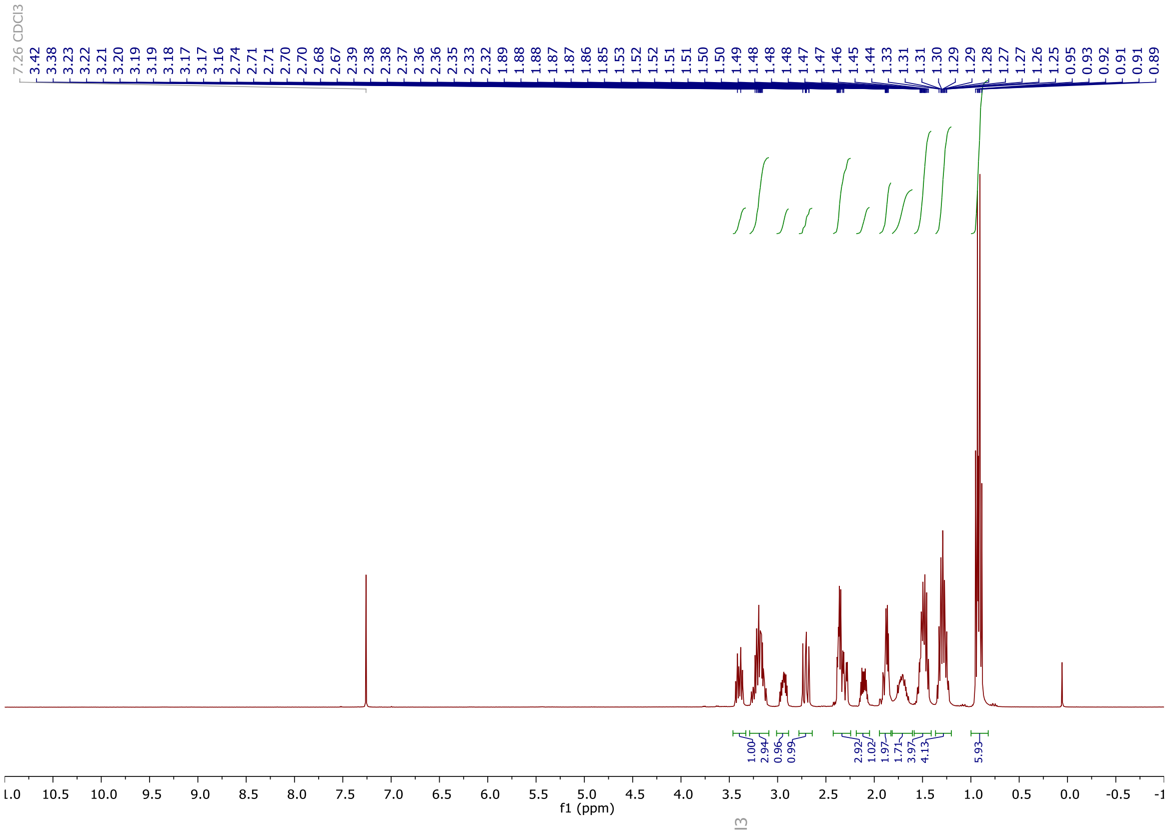

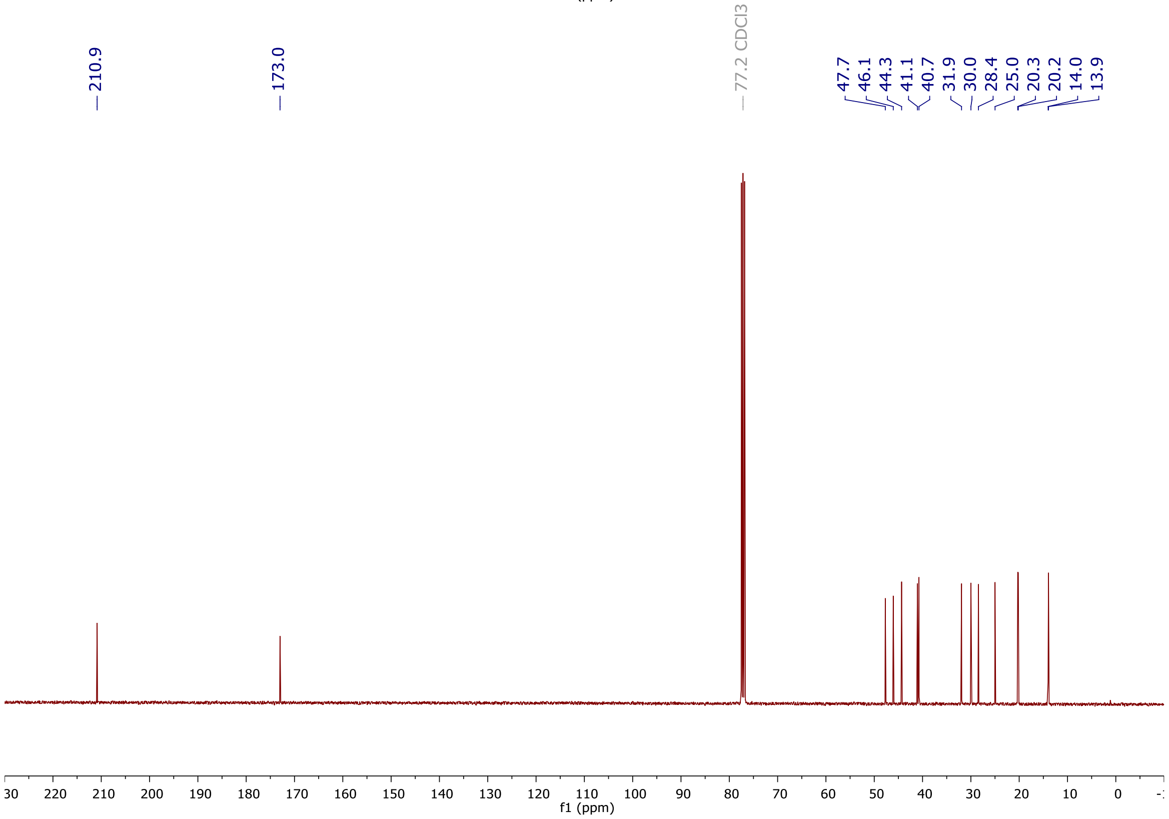

***N*,*N*-Dibutyl-3-(trimethylsilyl)benzamide (13a)**

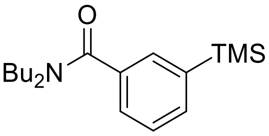

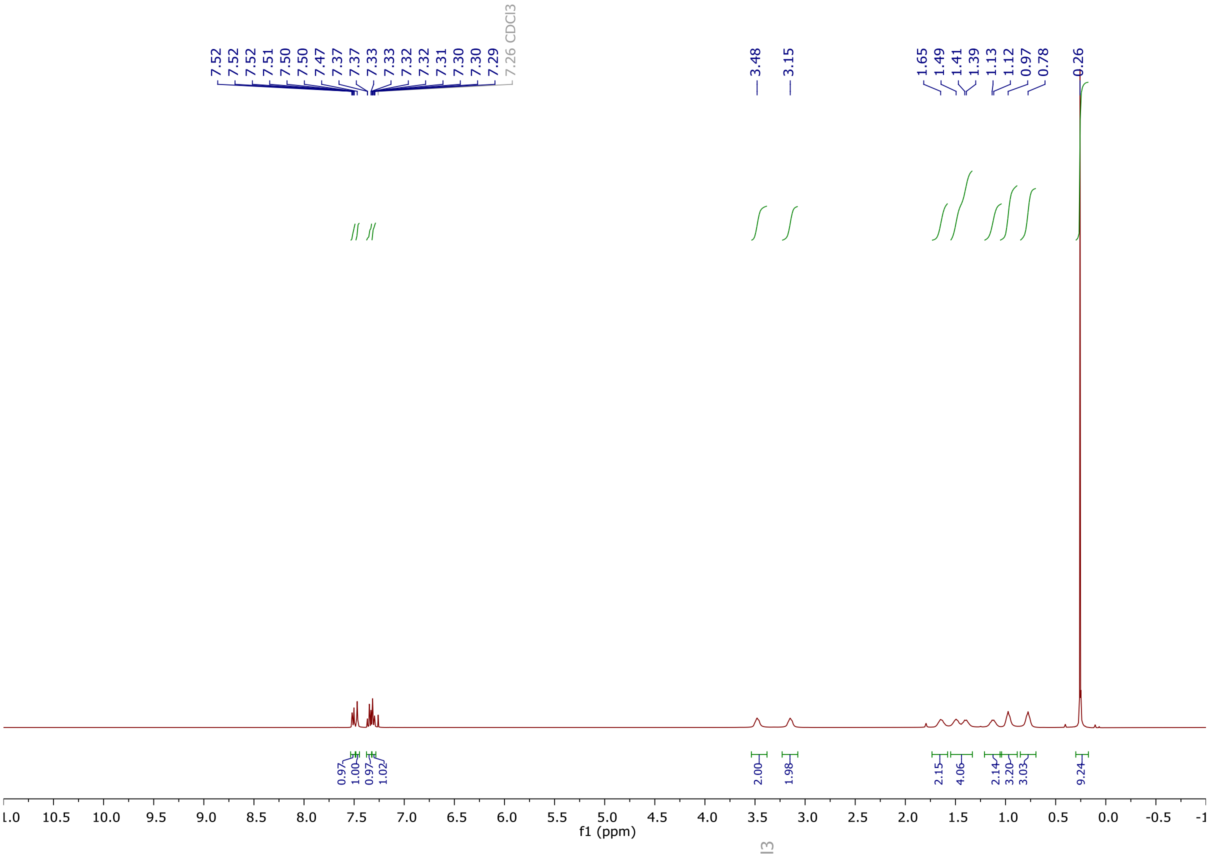

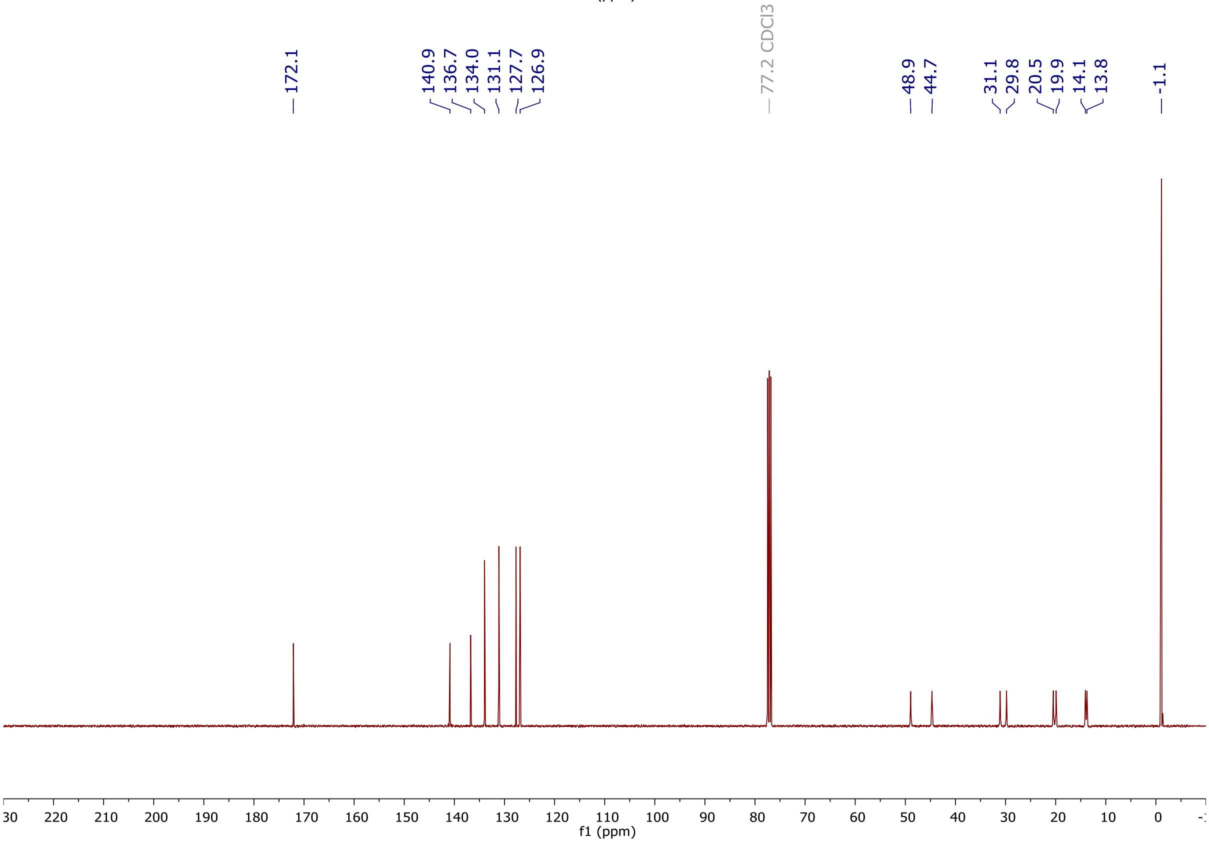

***N*,*N*-Dibutyl-3-cyanobenzamide (13b)**

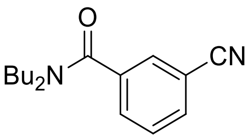

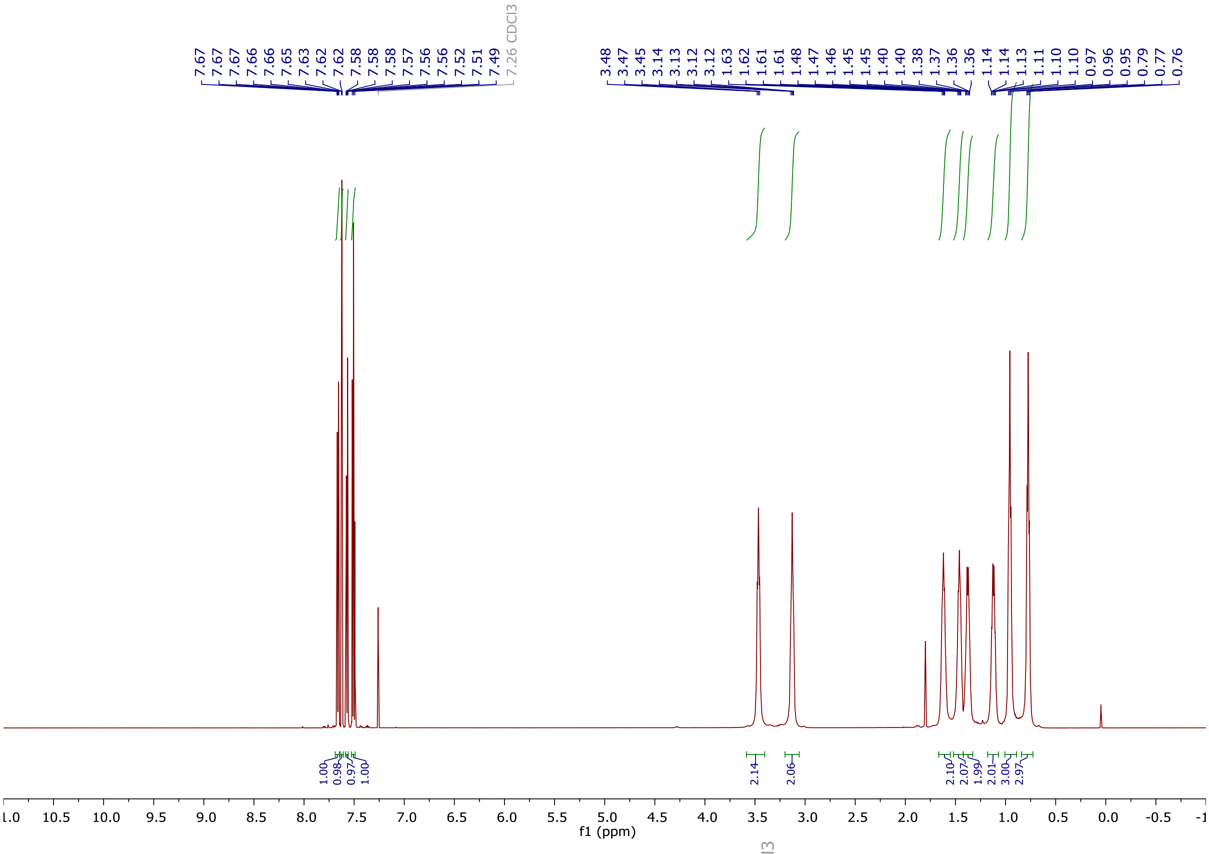

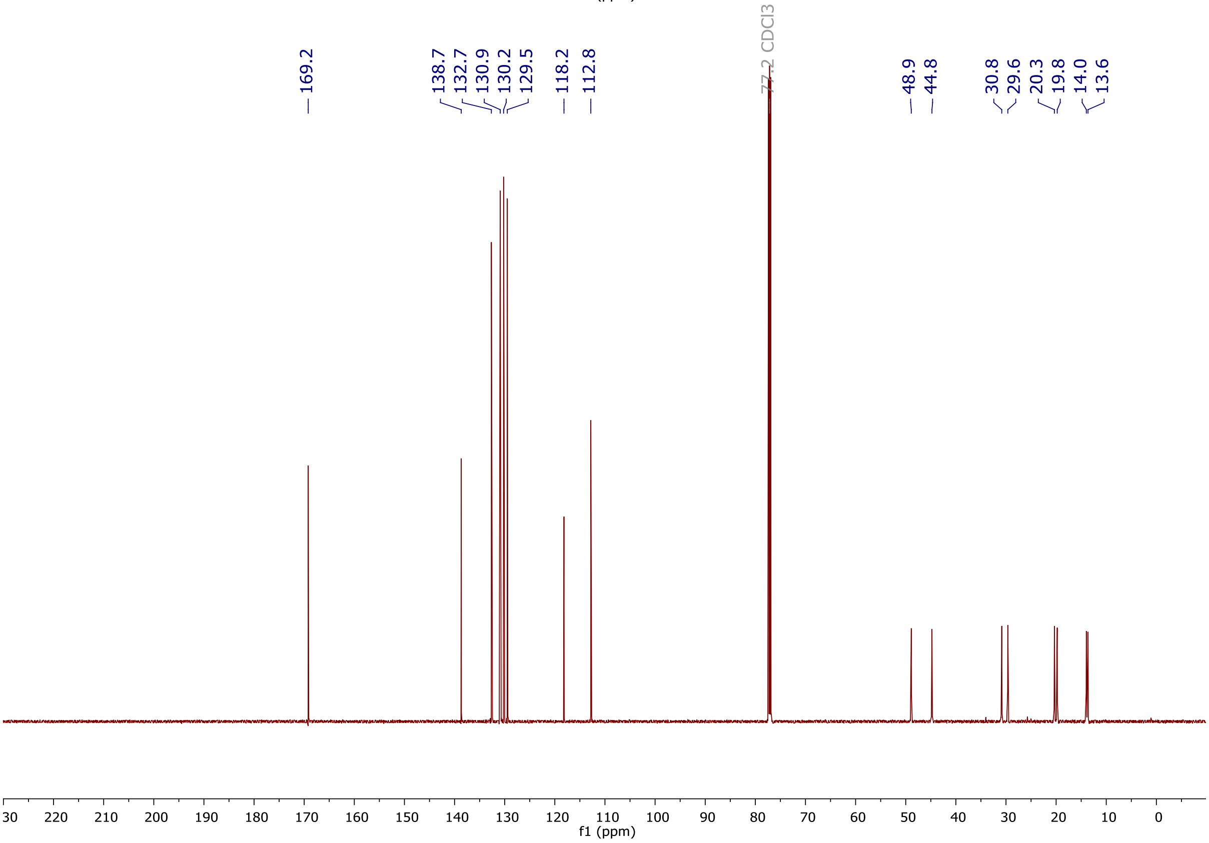

Ethyl 3-(dibutylcarbamoyl)benzoate (13c)

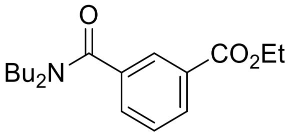

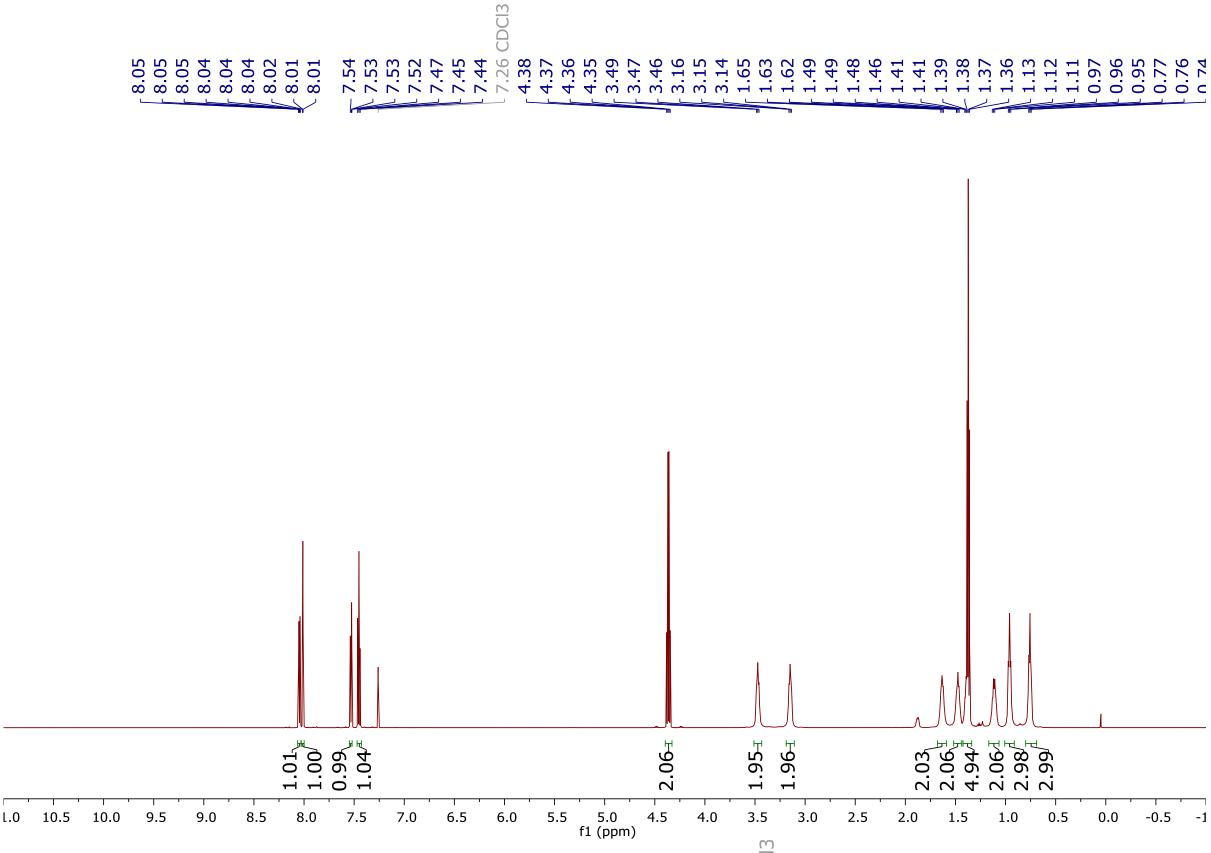

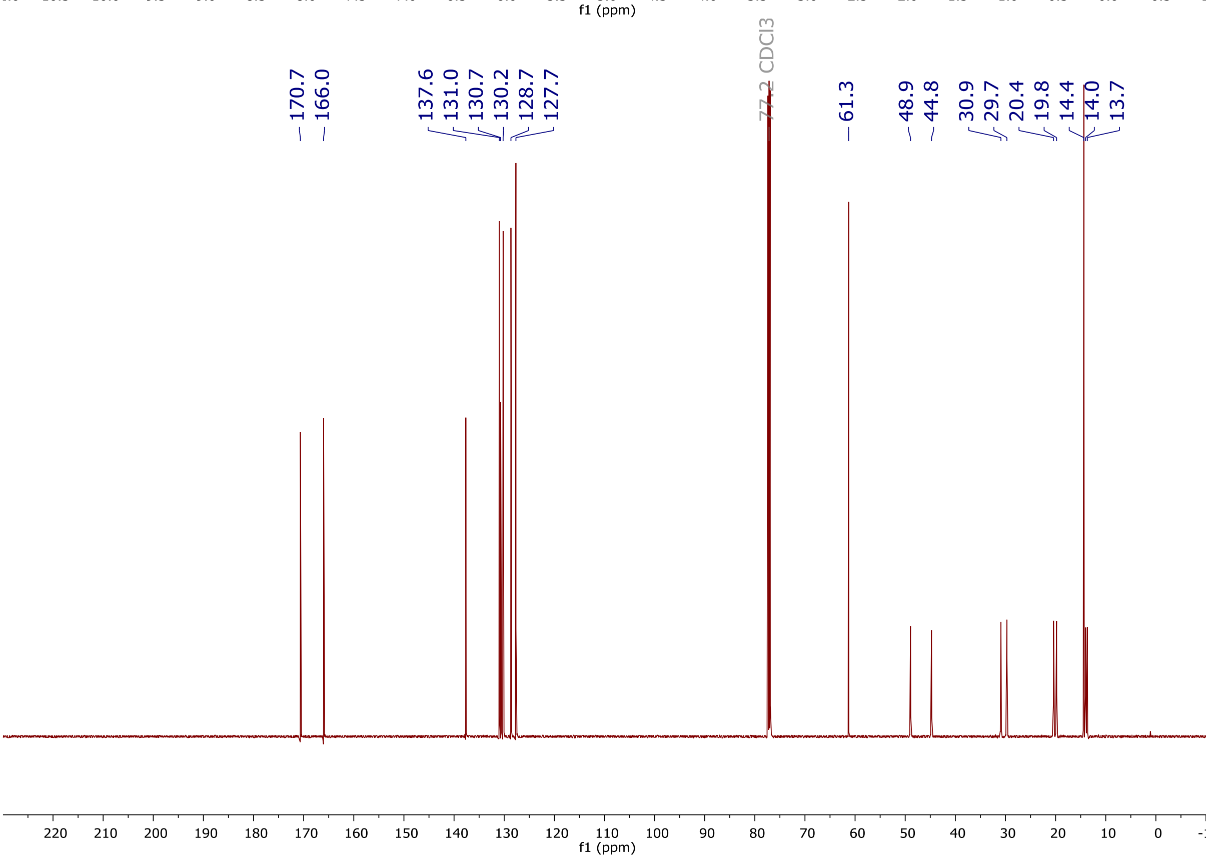

***N*,*N*-Dibutyl-4-(trifluoromethyl)benzamide (13d)**

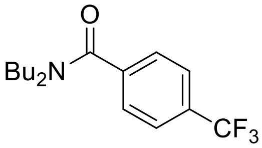

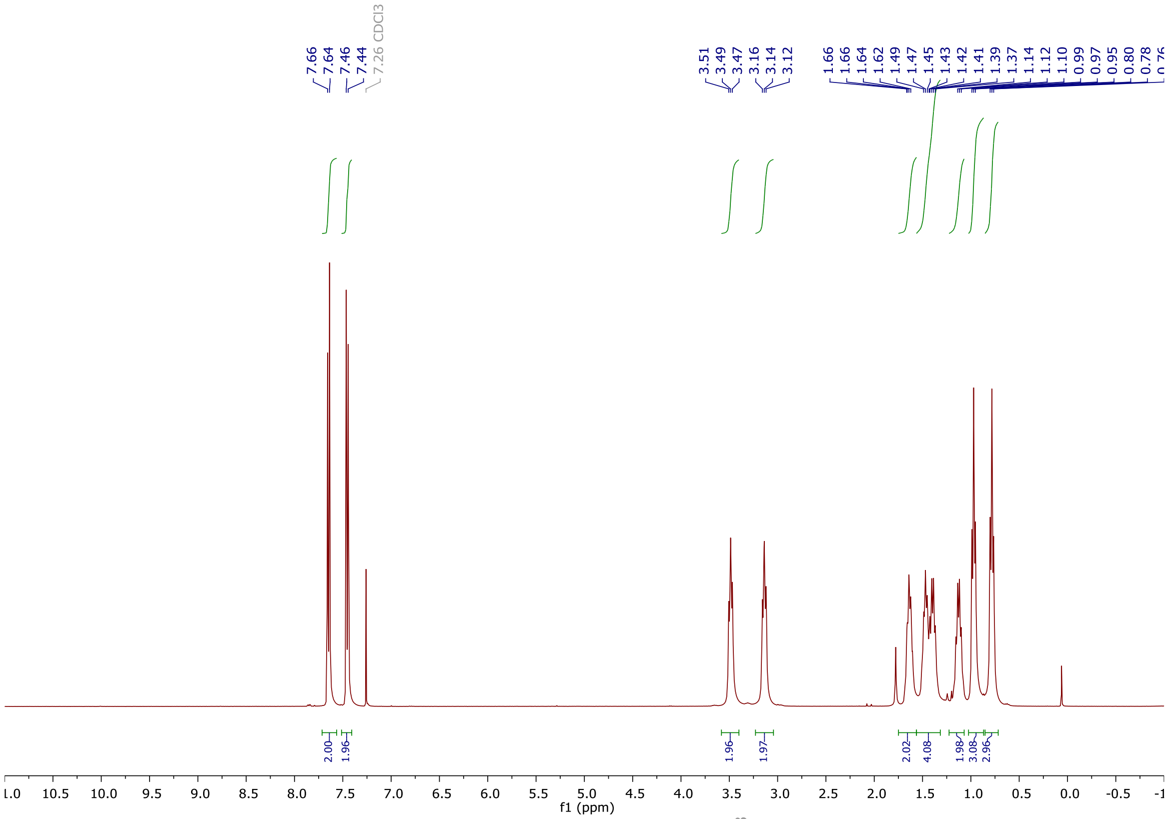

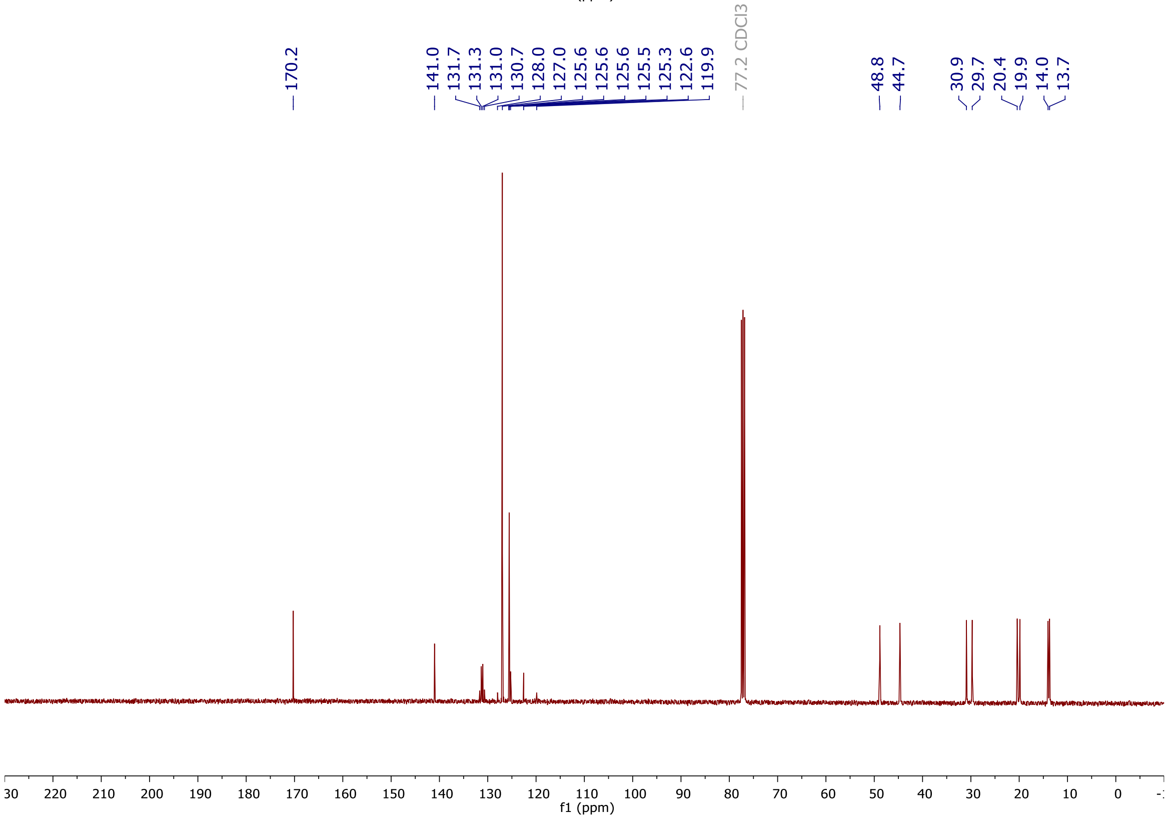

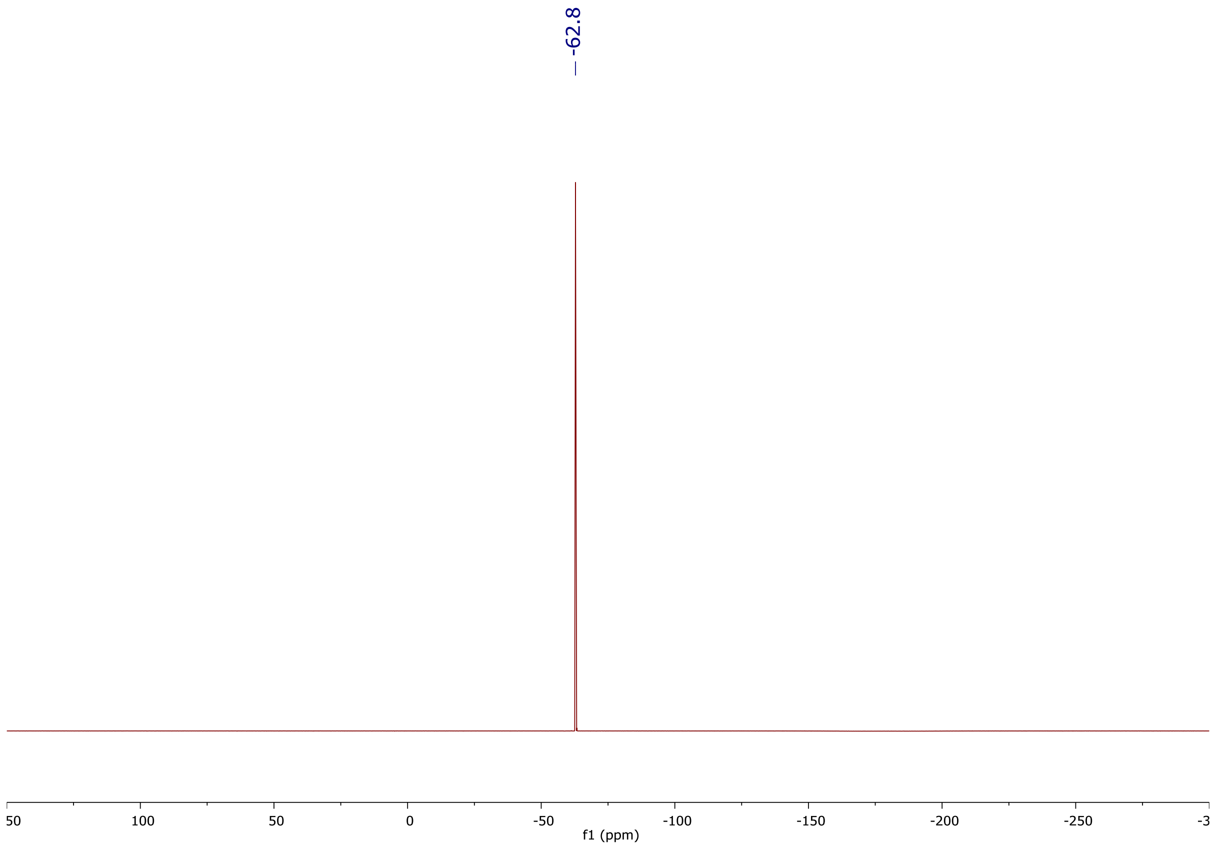
-62.8
50
100
50
0
-50
-100
-150
-200
-250
-3
f1 (ppm)

***N*,*N*-Dibutyl-4-(trifluoromethoxy)benzamide (13e)**

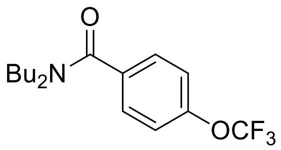

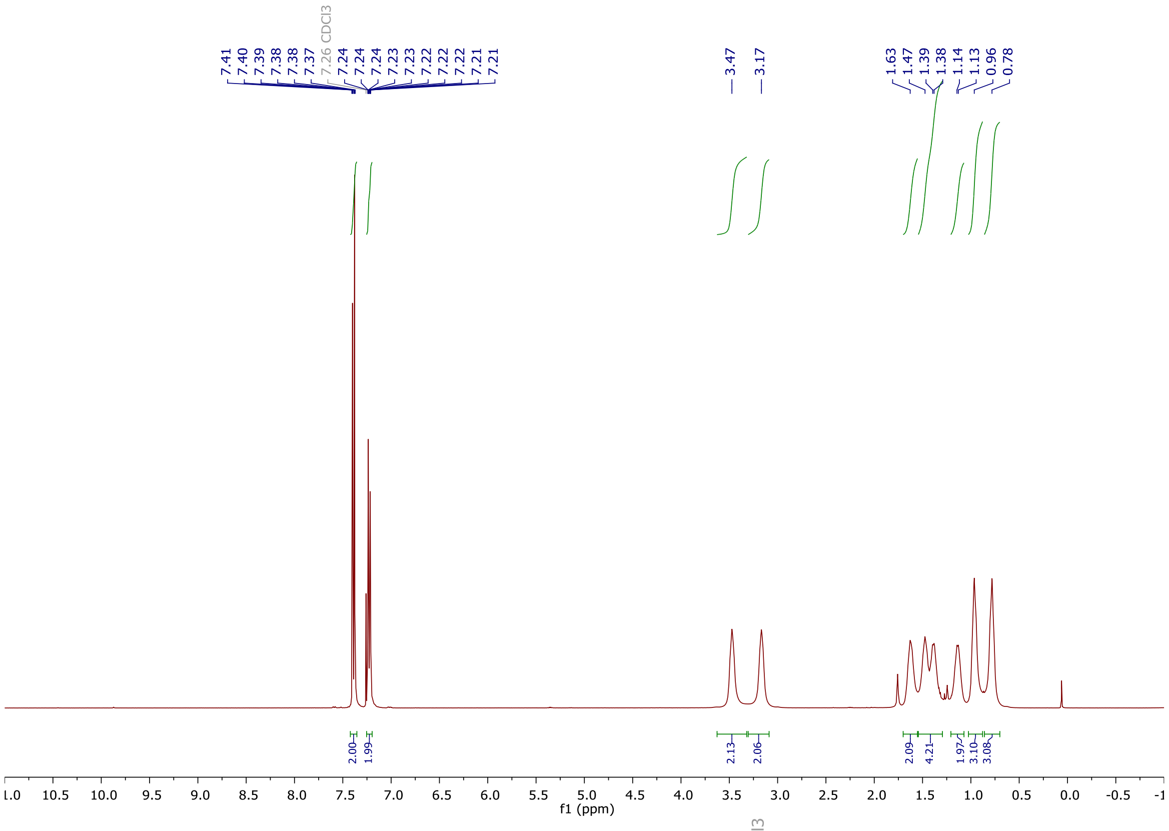

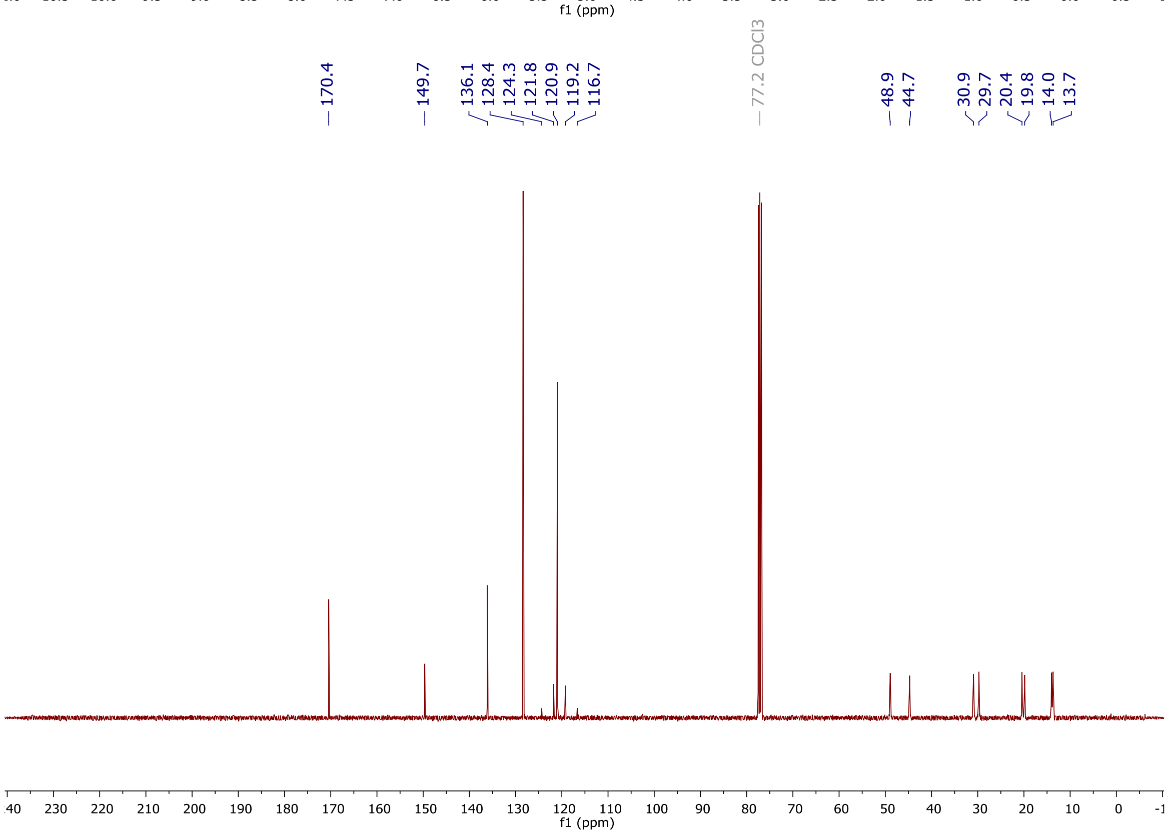

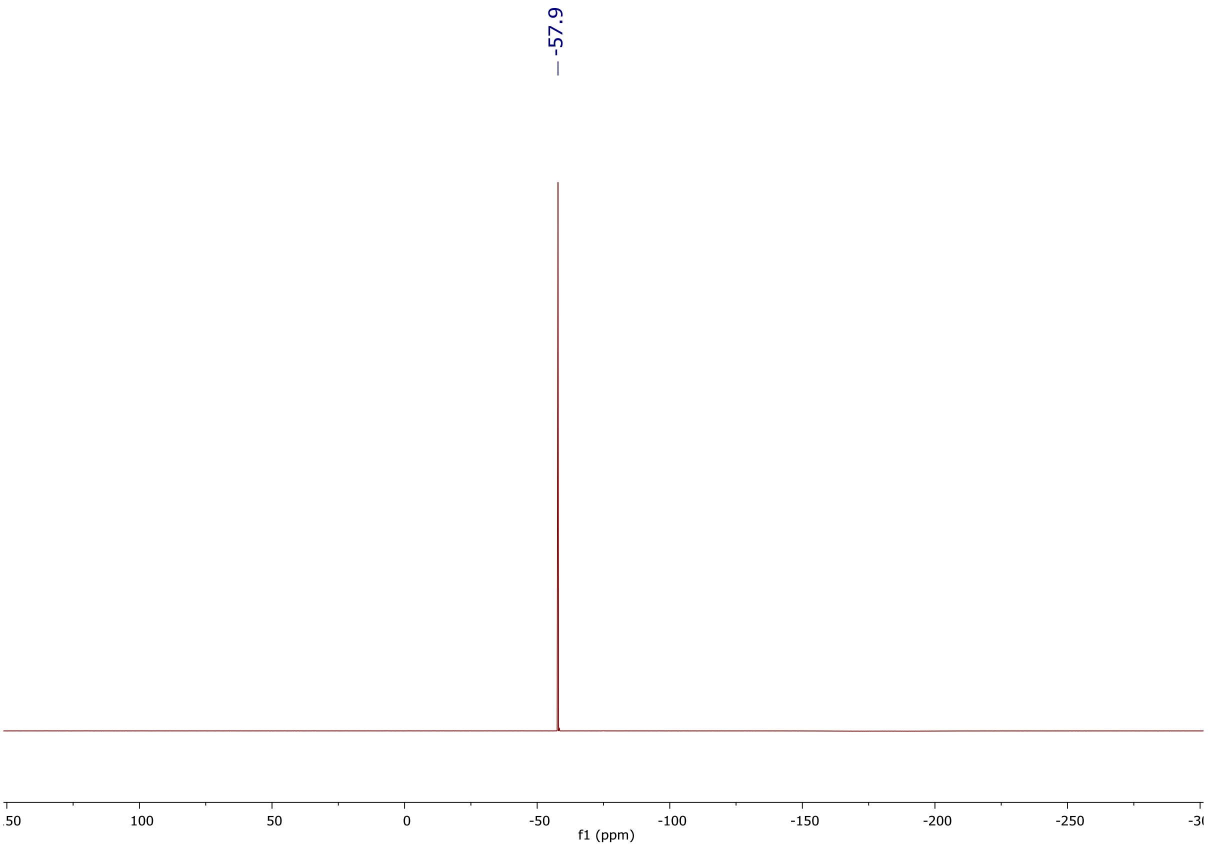
-57.9
50
100
50
0
-50
-100
-150
-200
-250
-30
f1 (ppm)

_N_,_N_-Dibutyl-4-methoxybenzamide (13f)

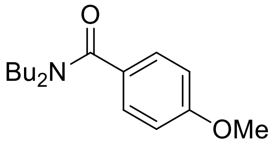

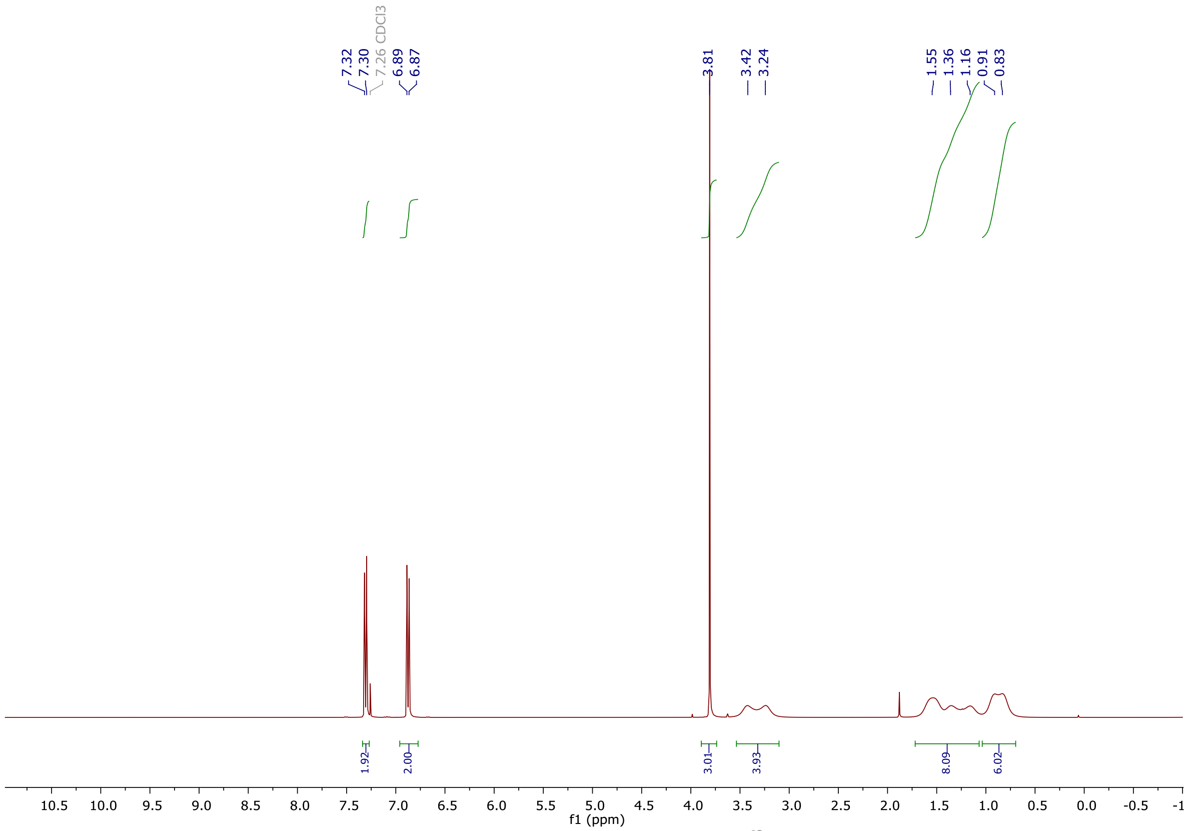

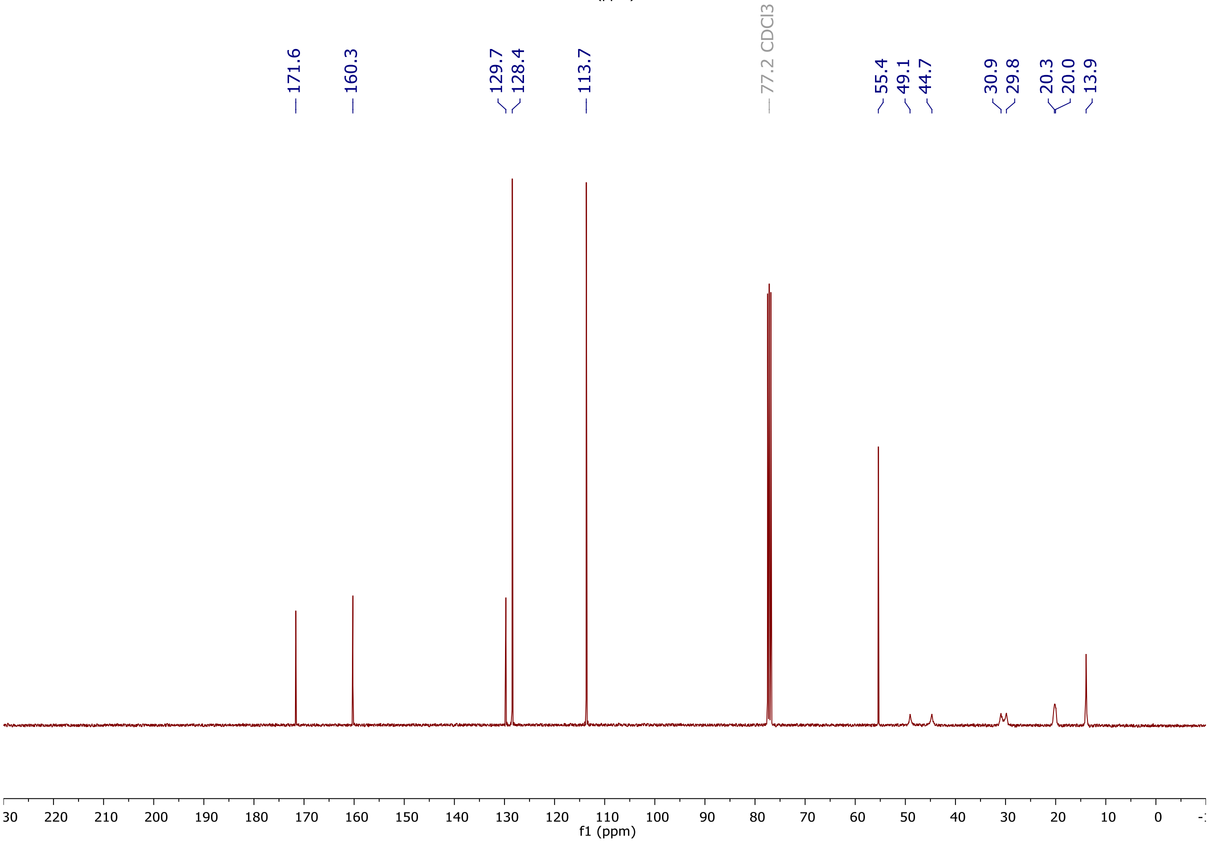

Methyl 6-(dibutylcarbamoyl)-2-naphthoate (13g)

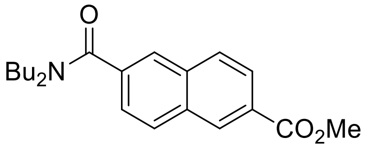

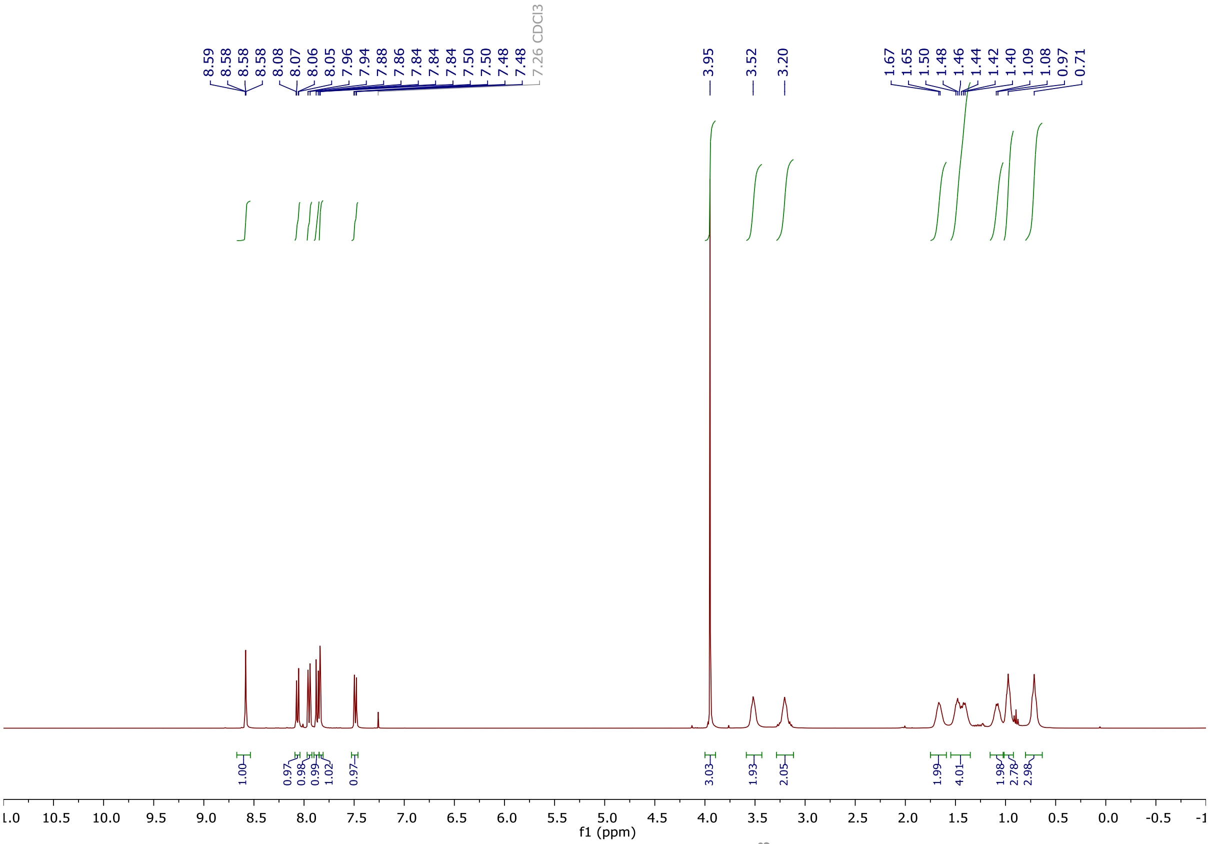

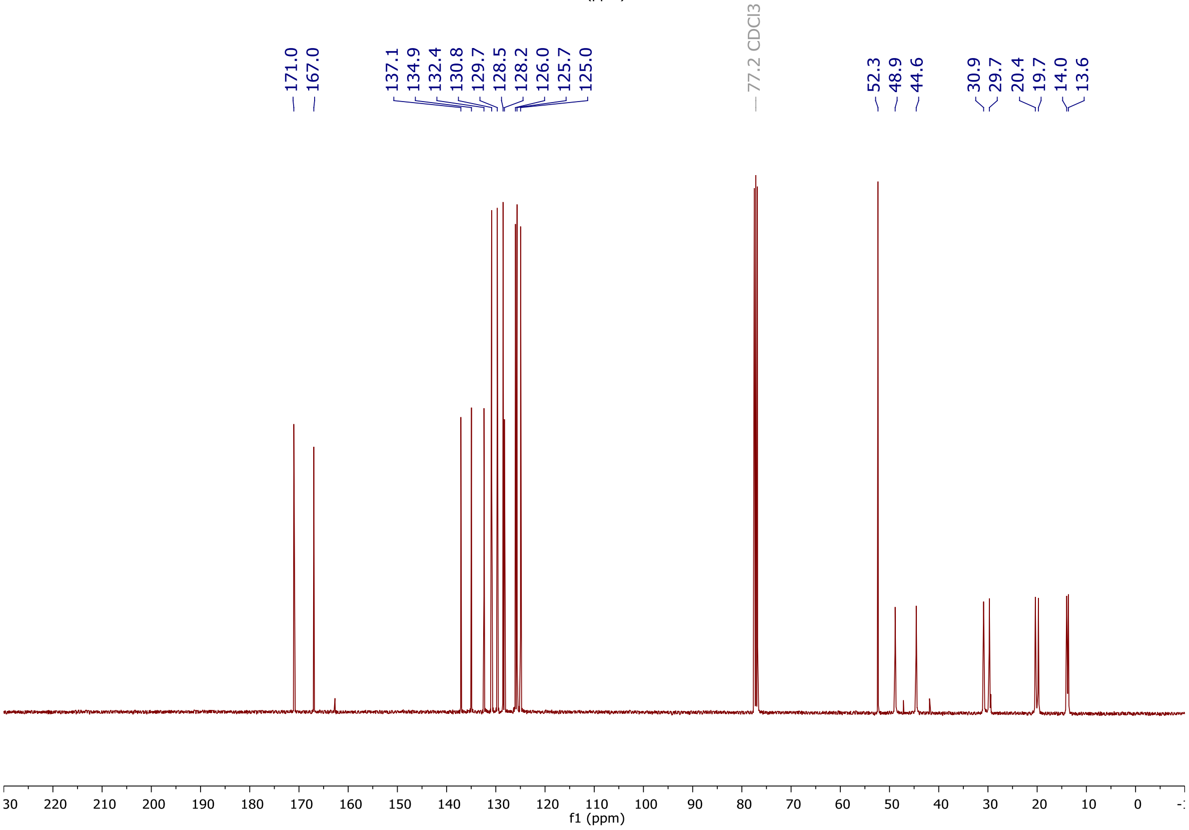

N,N-Dibutyl-2-methyl-1-naphthamide (13h)

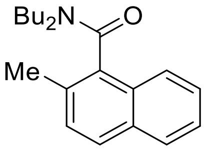

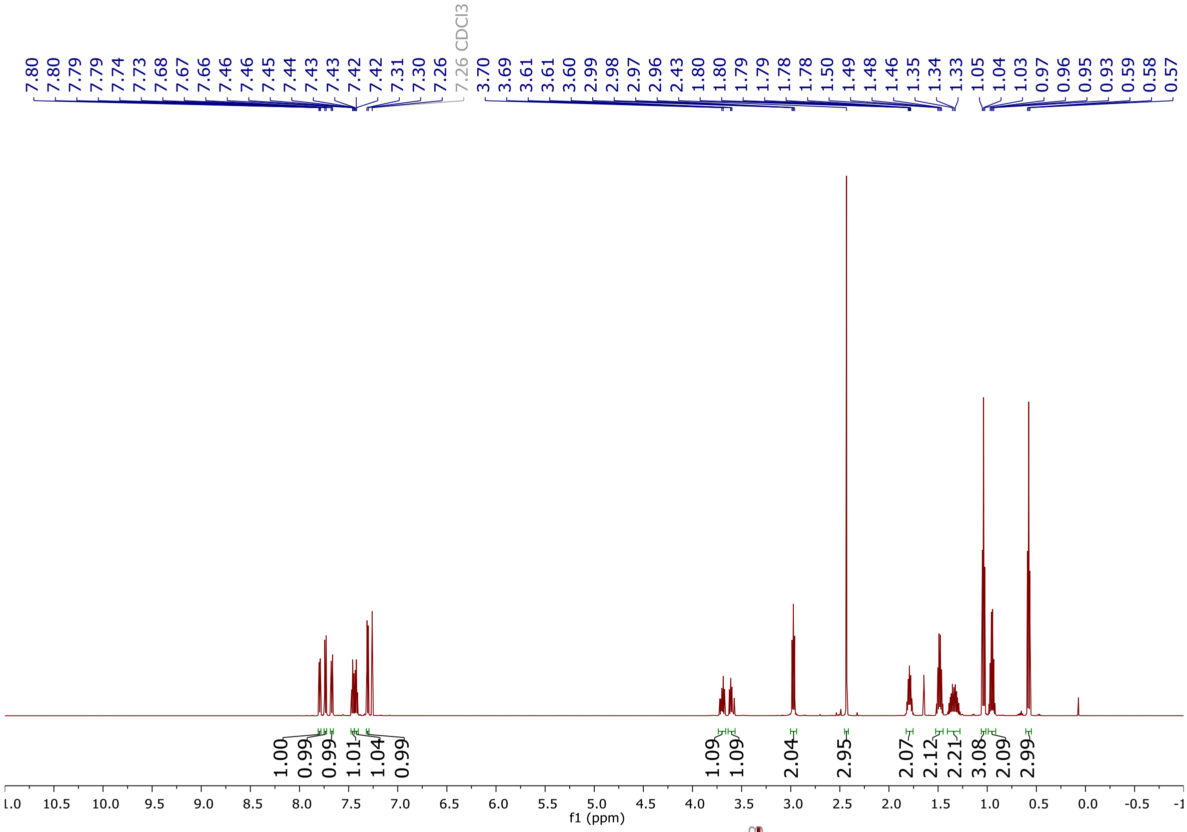

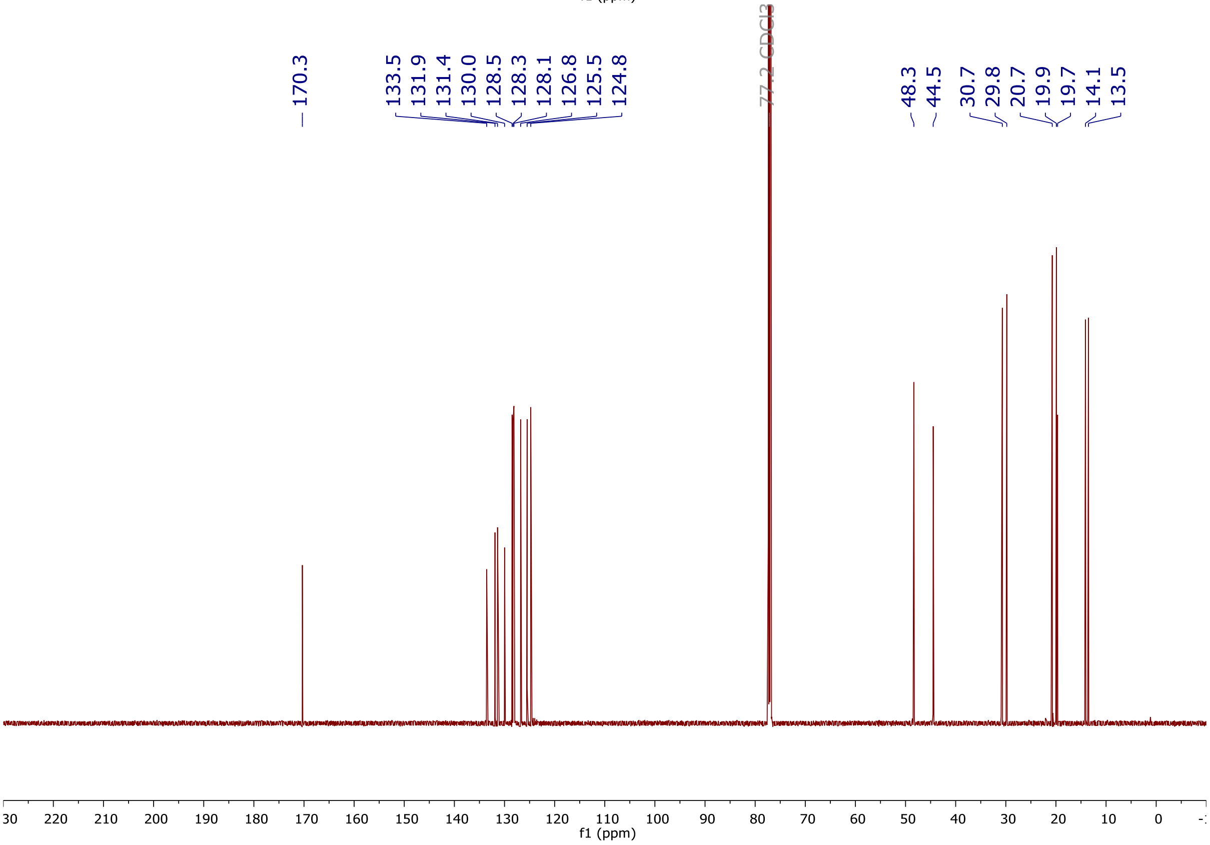

***N*,*N*-Dibutylquinoline-2-carboxamide (13i)**

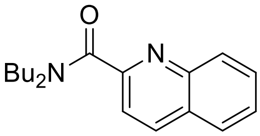

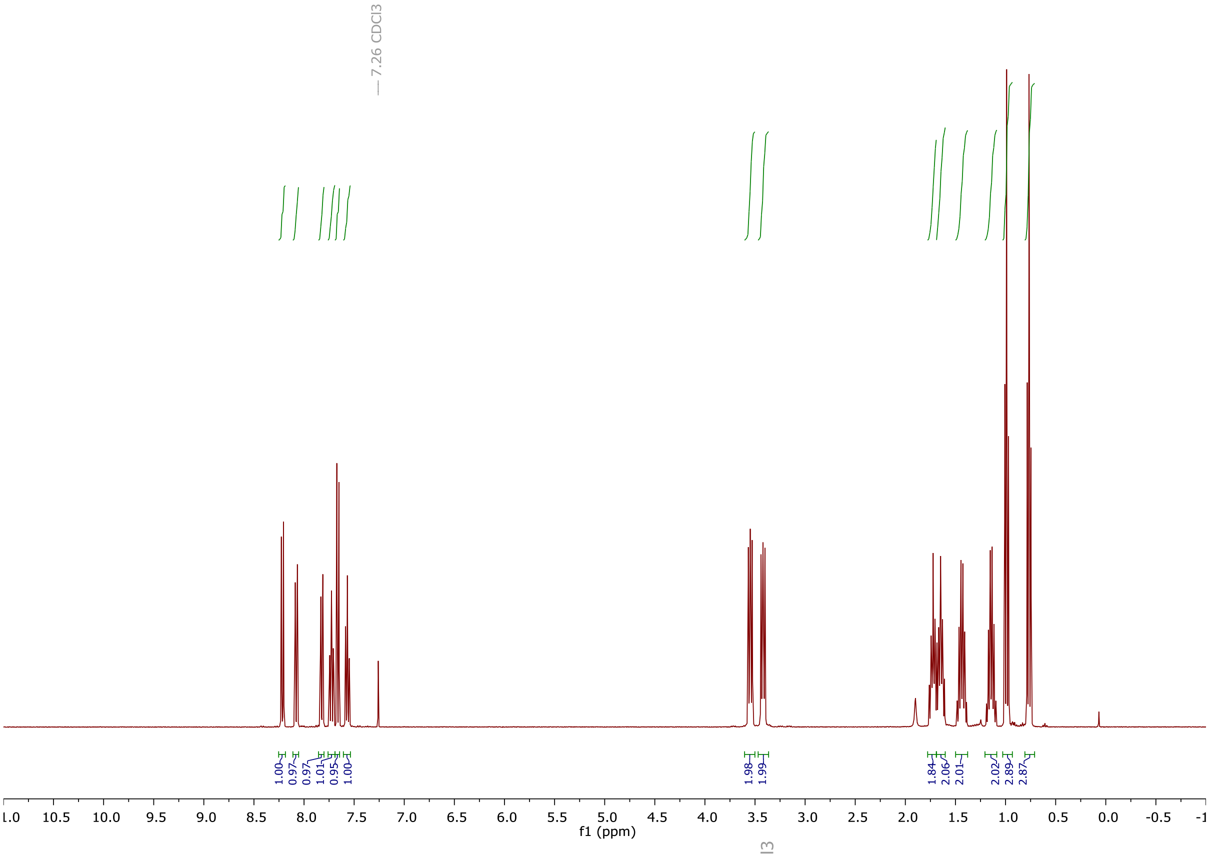

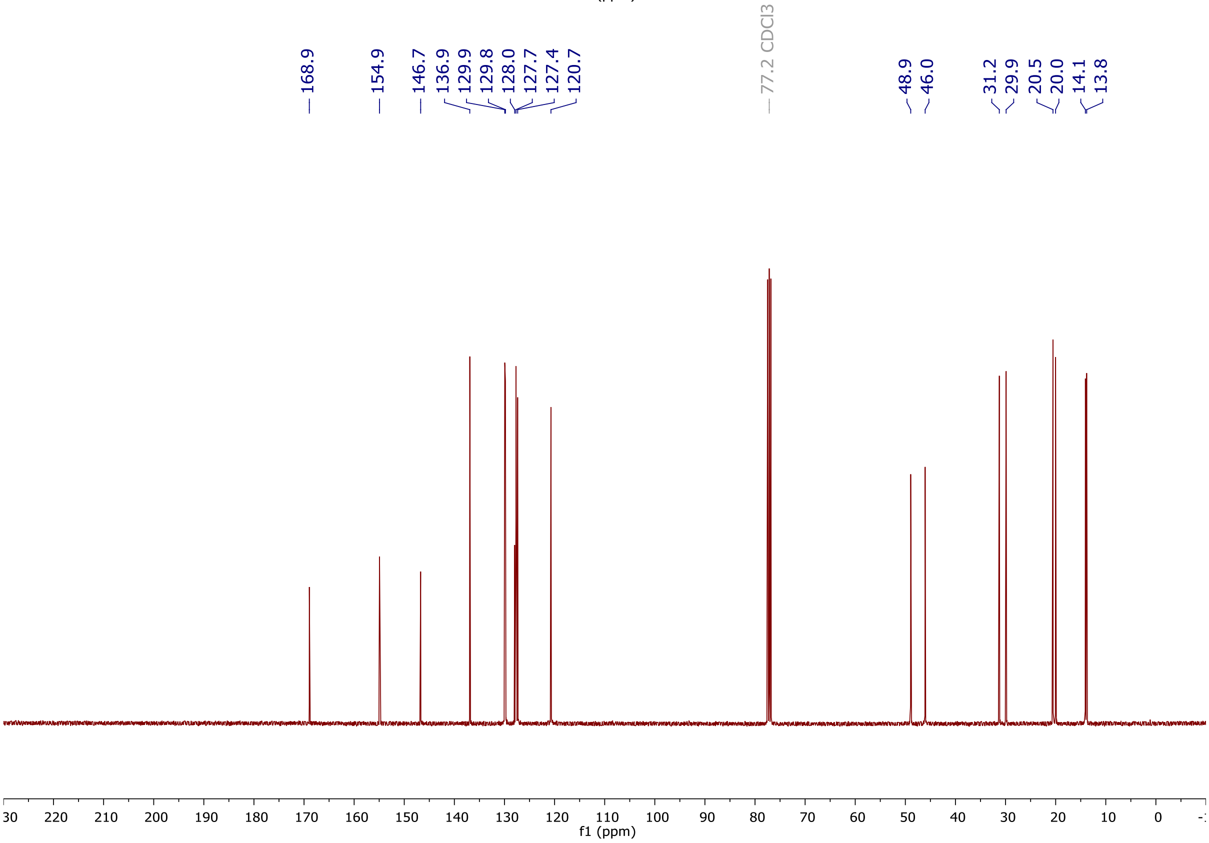

N,N-Dibutyl-6-methoxypicolinamide (13j)

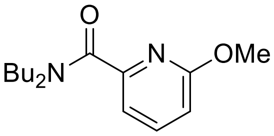

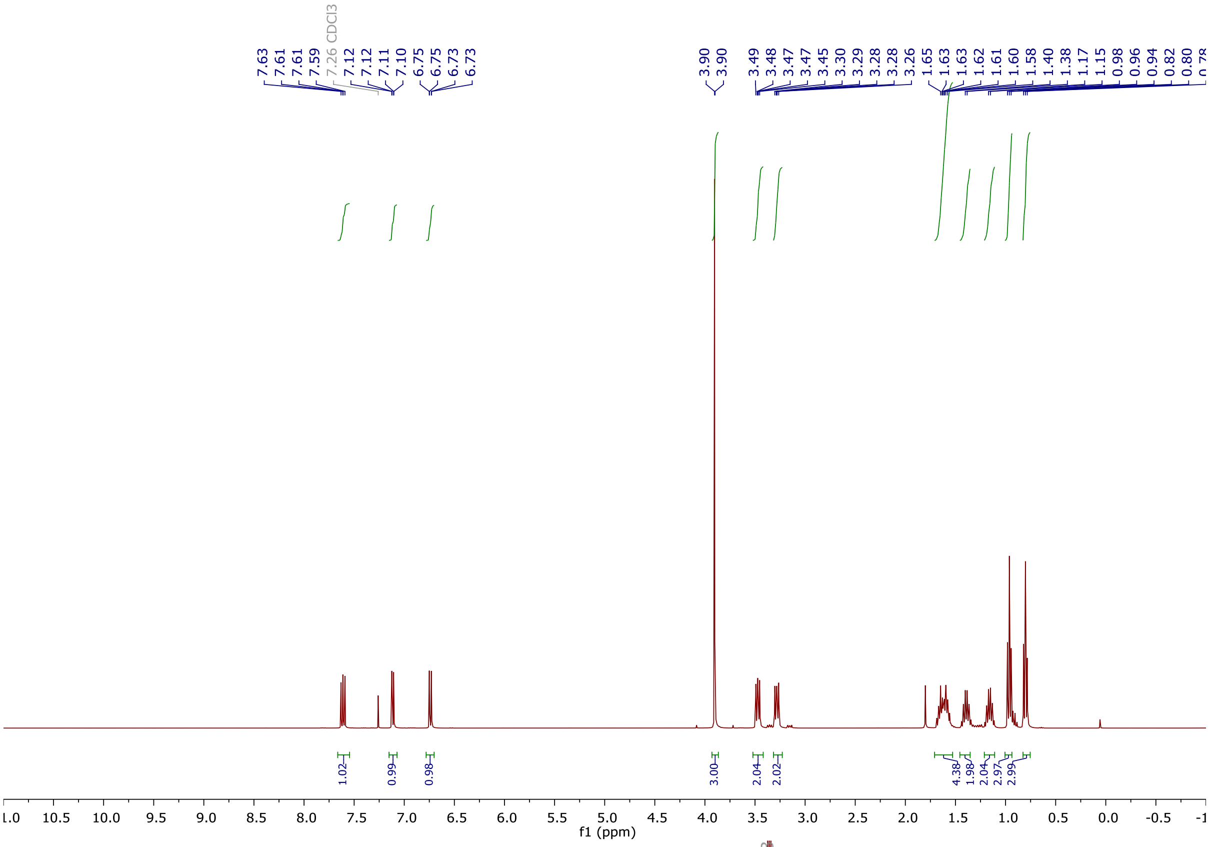

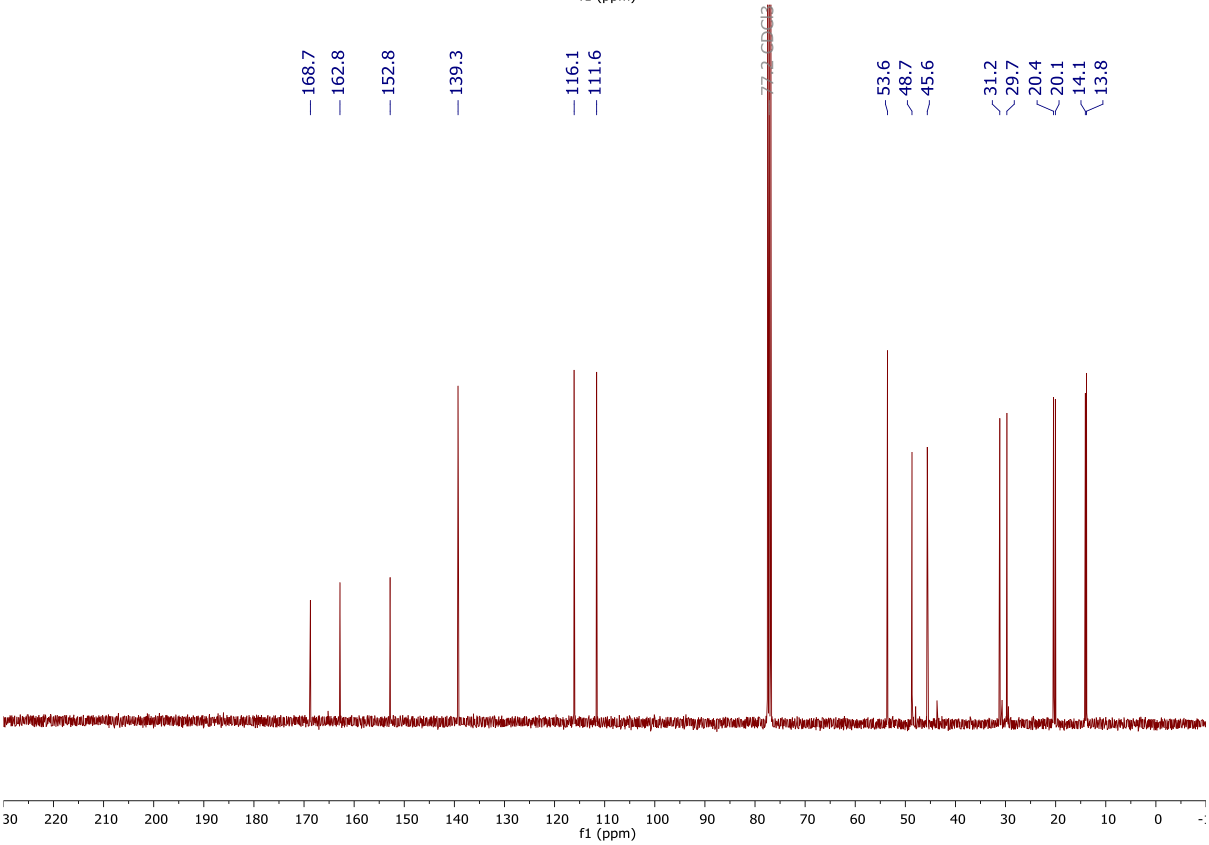

_N_,_N_-Dibutyl-3-((5-(4-fluorophenyl)thiophen-2-yl)methyl)-4-methylbenzamide (13k)

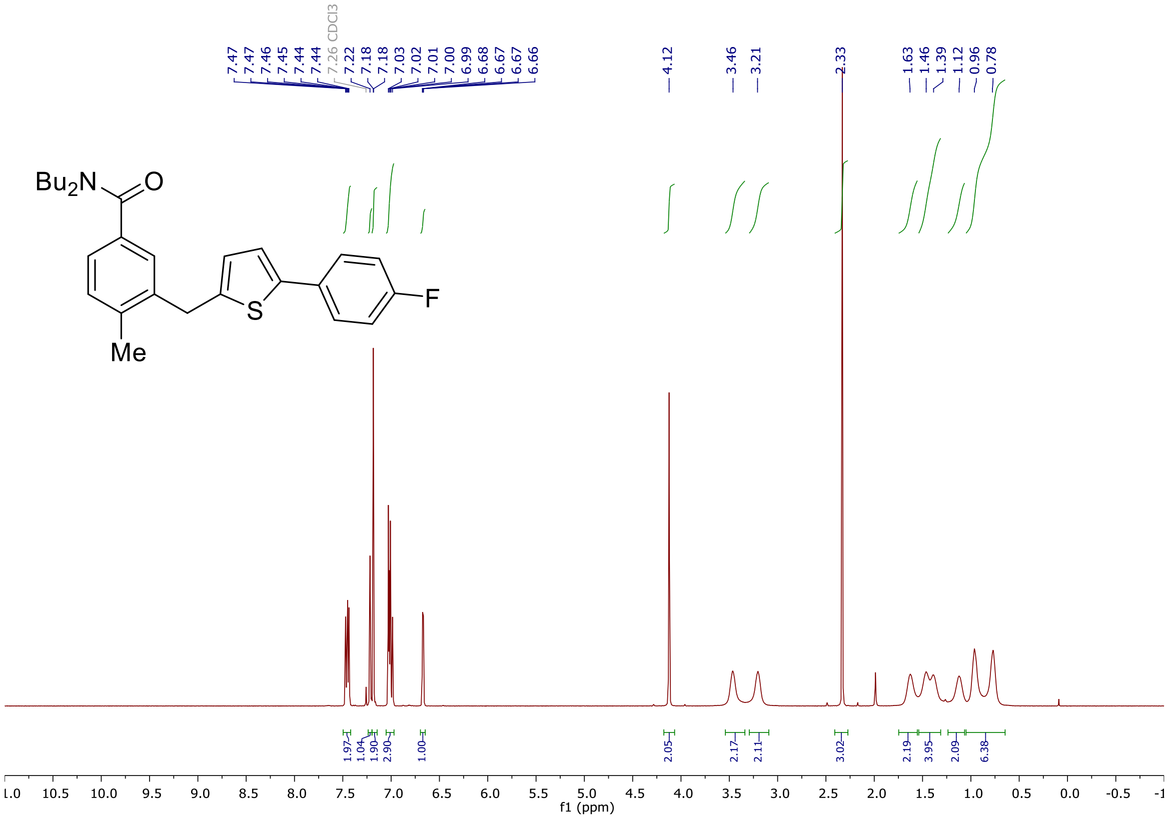

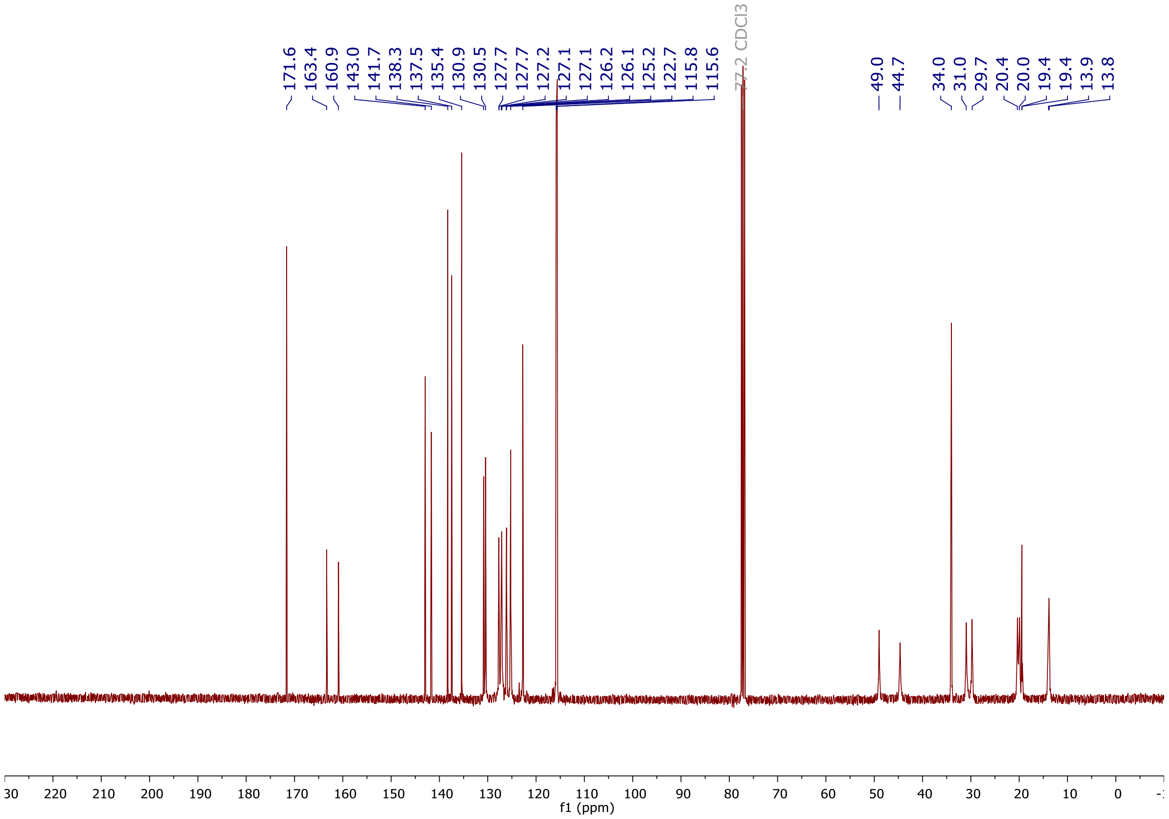

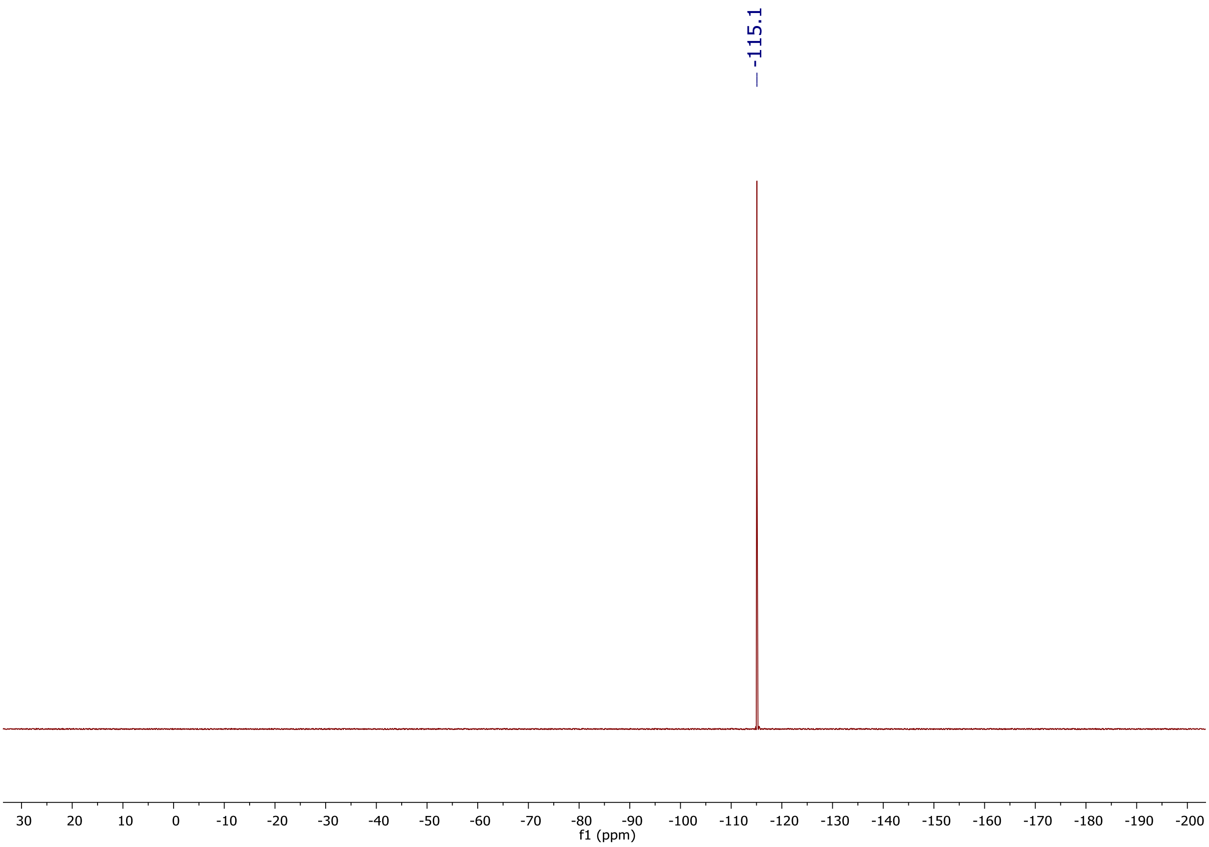

-115.1
30 20 10 0 -10 -20 -30 -40 -50 -60 -70 -80 -90 -100 -110 -120 -130 -140 -150 -160 -170 -180 -190 -200
f1 (ppm)

3-(4-(4-Cyanophenyl)piperazine-1-carbonyl)benzonitrile (13l)

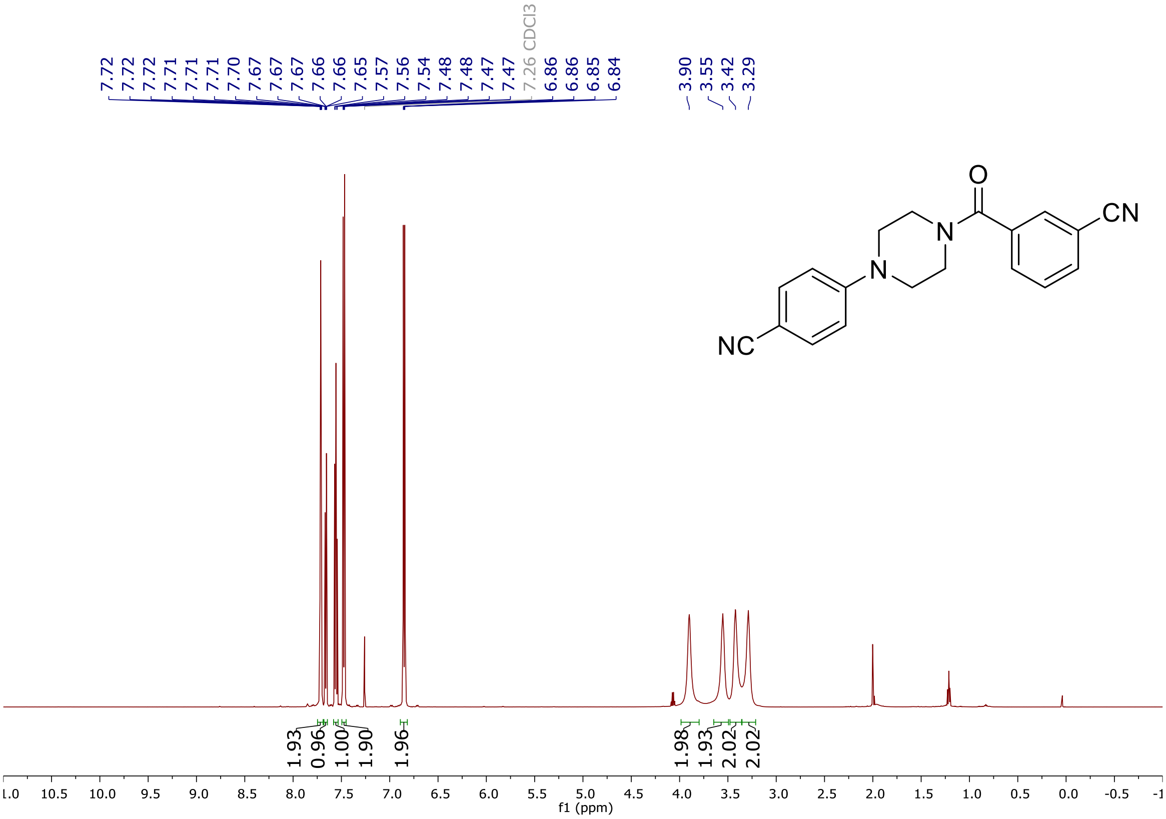

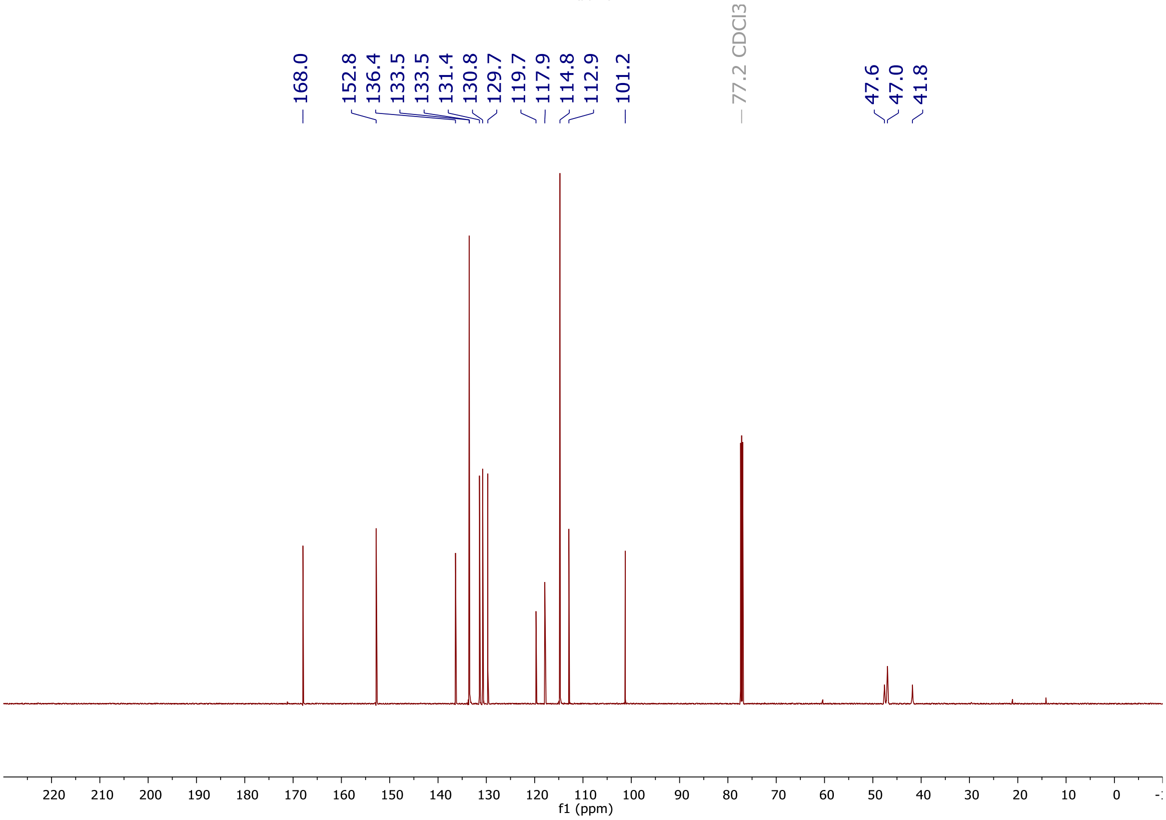

(4-(3-(Trifluoromethyl)pyridin-2-yl)piperazin-1-yl)(3-(trimethylsilyl)phenyl)methanone (13m)

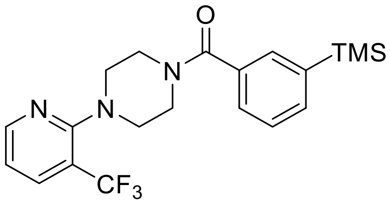

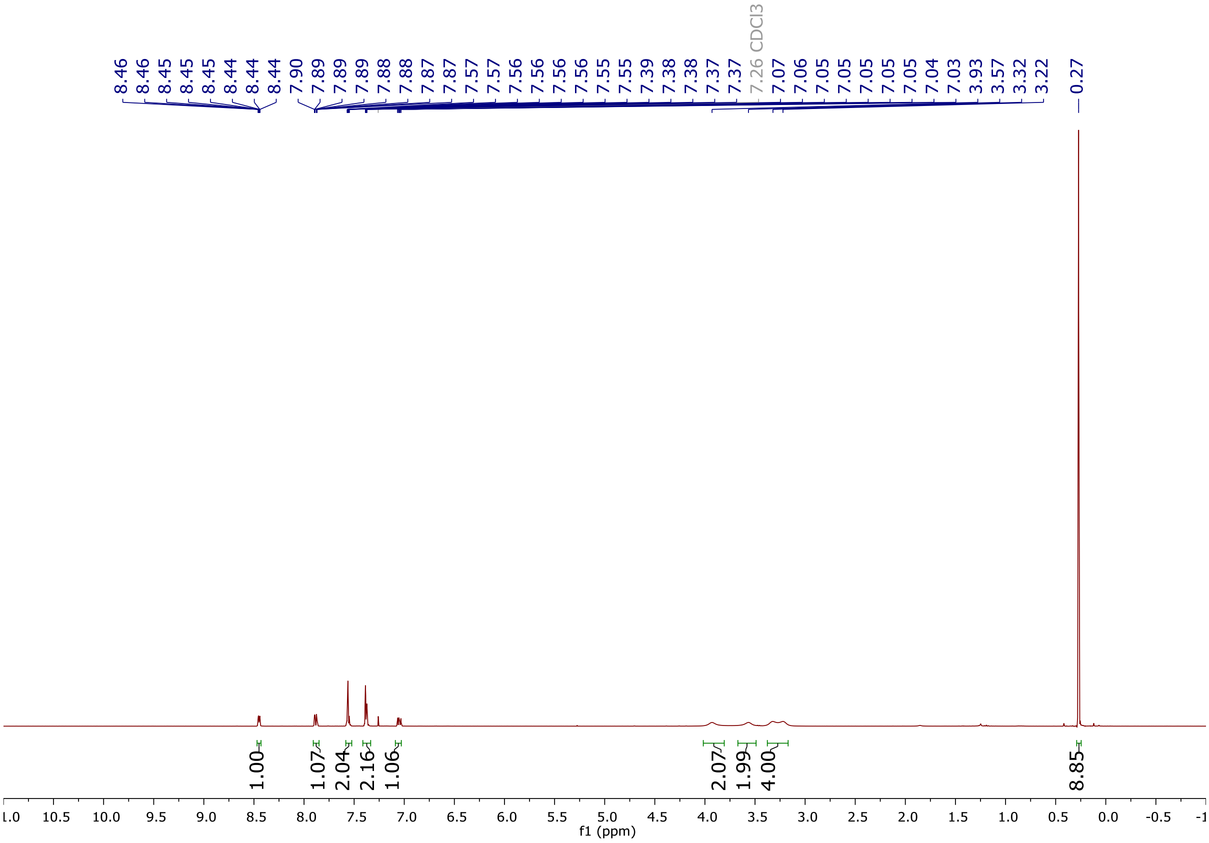

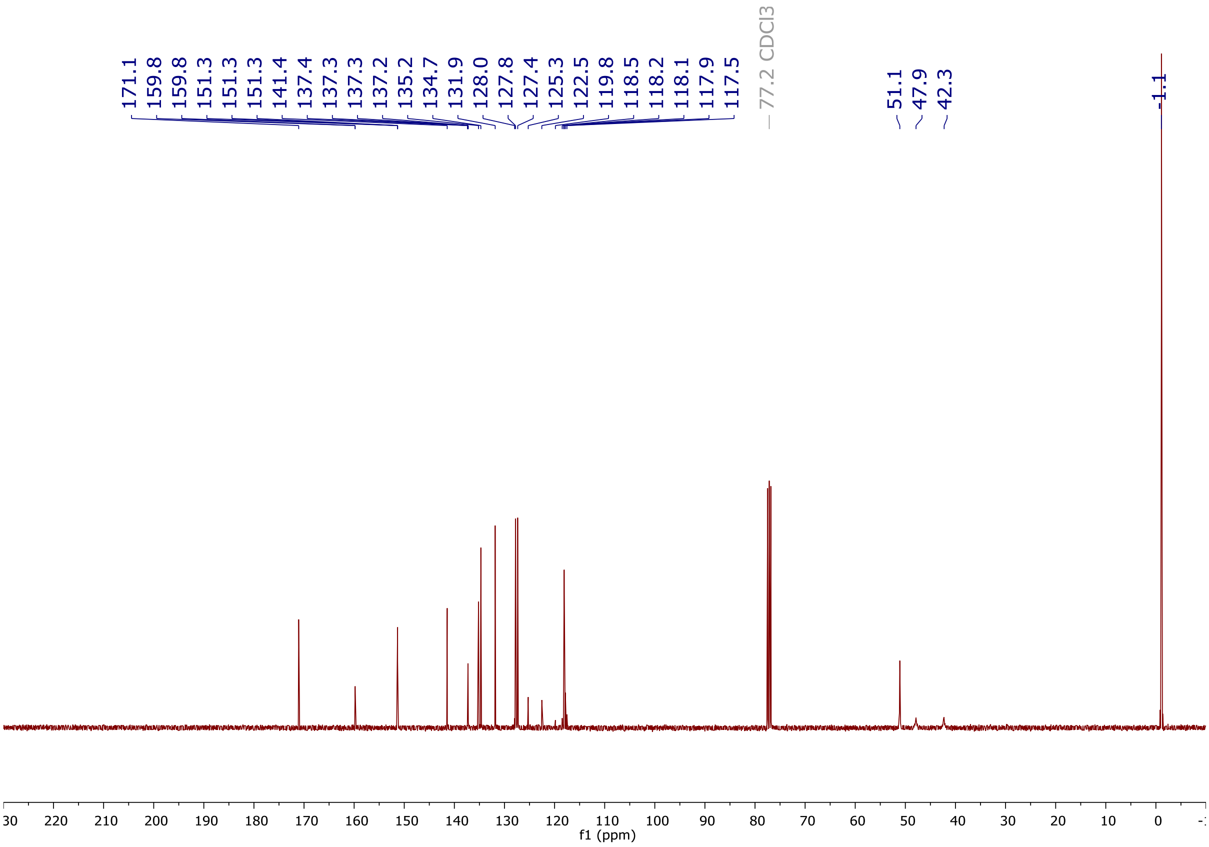
171.1
159.8
159.8
151.3
151.3
151.3
141.4
137.4
137.3
137.3
137.2
135.2
134.7
131.9
128.0
127.8
127.4
125.3
122.5
119.8
118.5
118.2
118.1
117.9
117.5
77.2 CDCl3
51.1
47.9
42.3
-1.1
f1 (ppm)

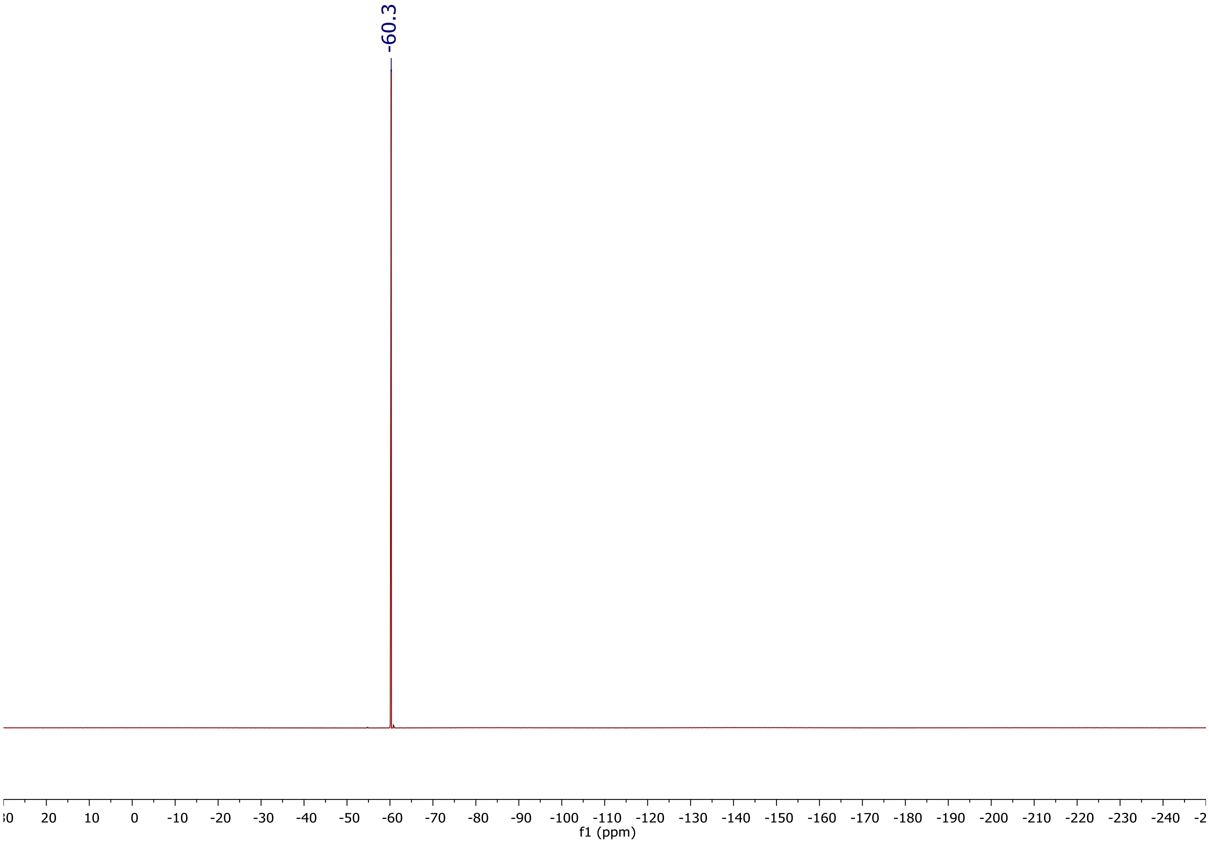
-60.3
f1 (ppm)

_N_-(3-(1,3-Dioxoisoindolin-2-yl)propyl)-_N_-isopropyl-6-methoxypicolinamide **(13n)**

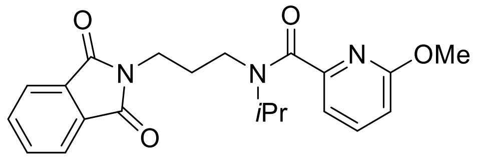

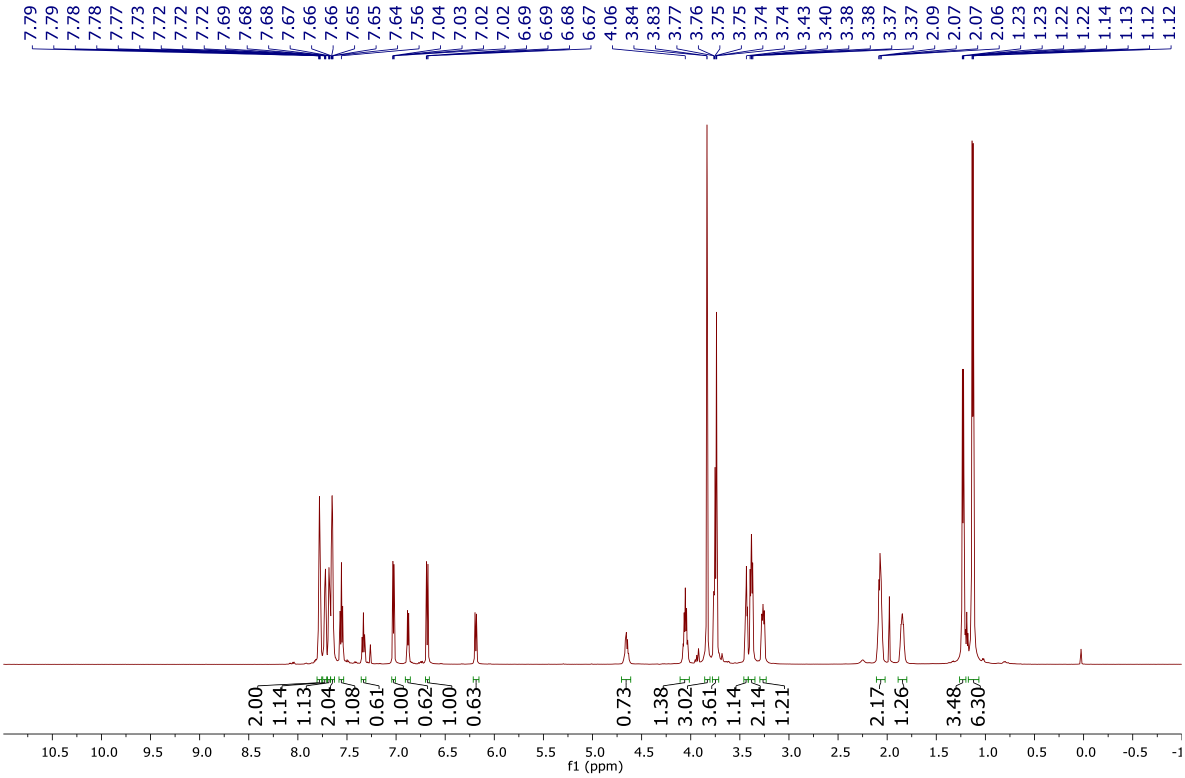

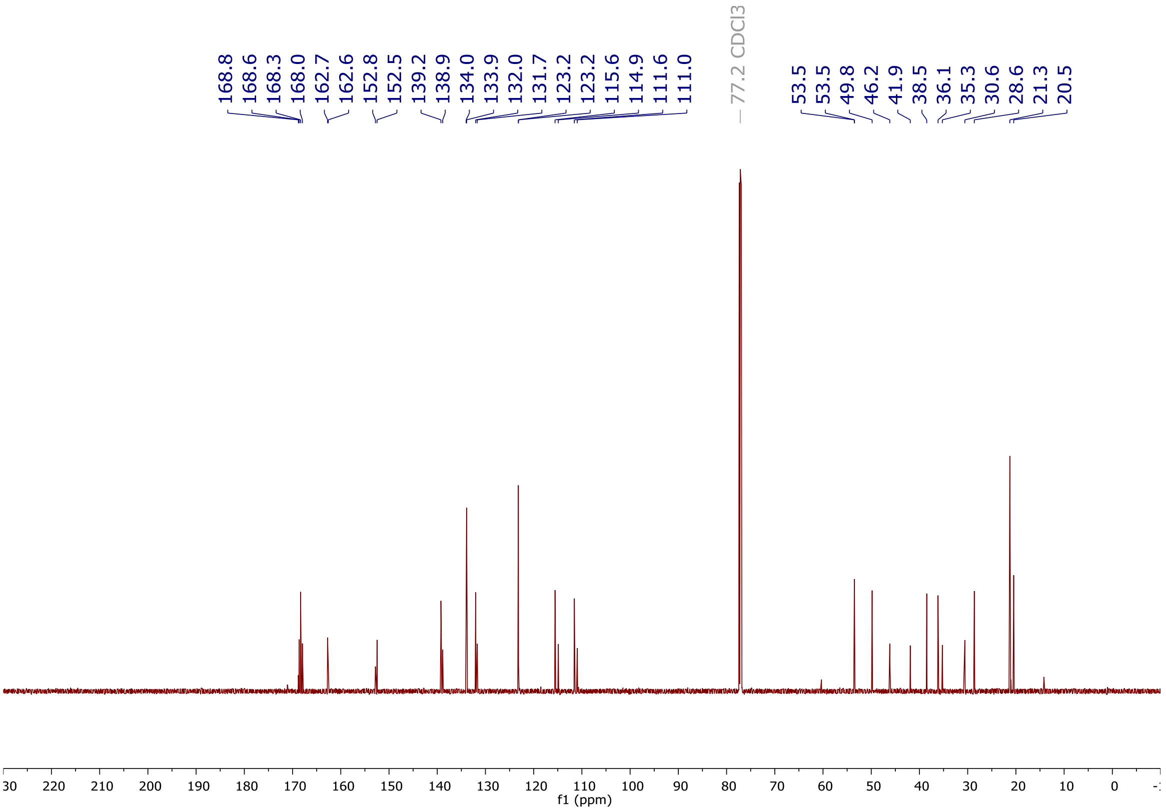

***N*,*N*-Dibutylcinnamamide (13o)**

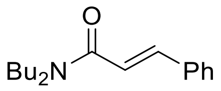

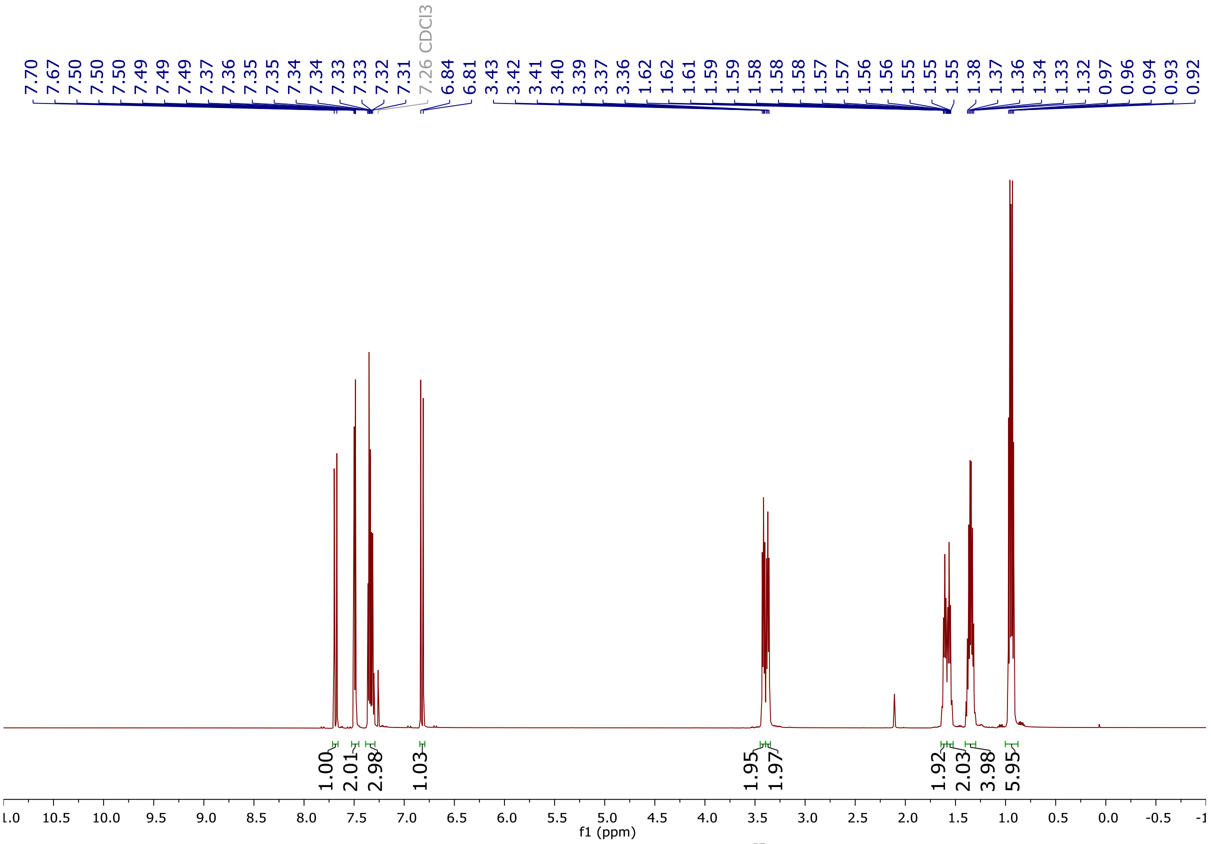

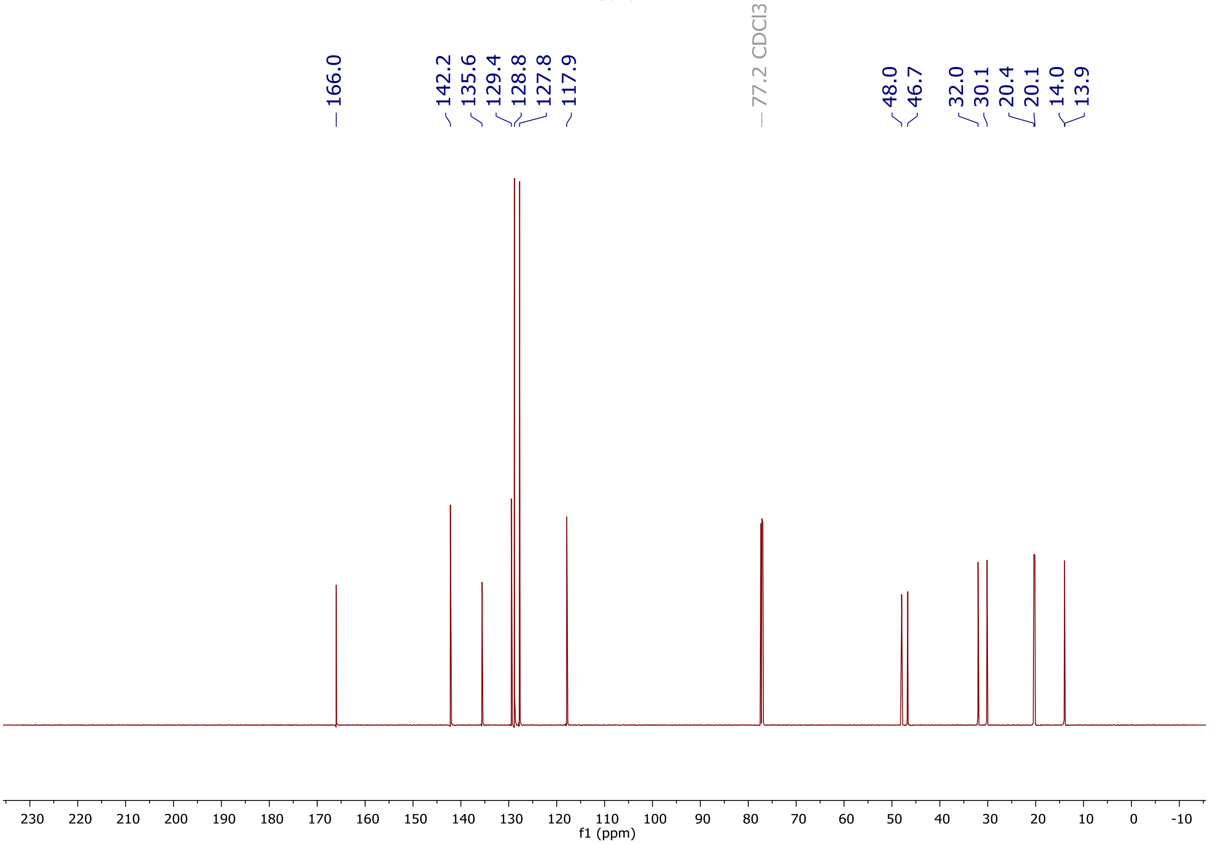

(*E*)-3-(4-Methoxyphenyl)-1-(*trans*-octahydroquinolin-2(1H)-yl)prop-2-en-1-one (13p)

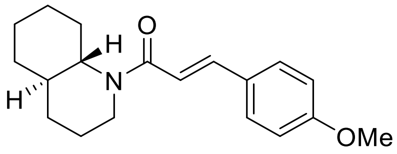

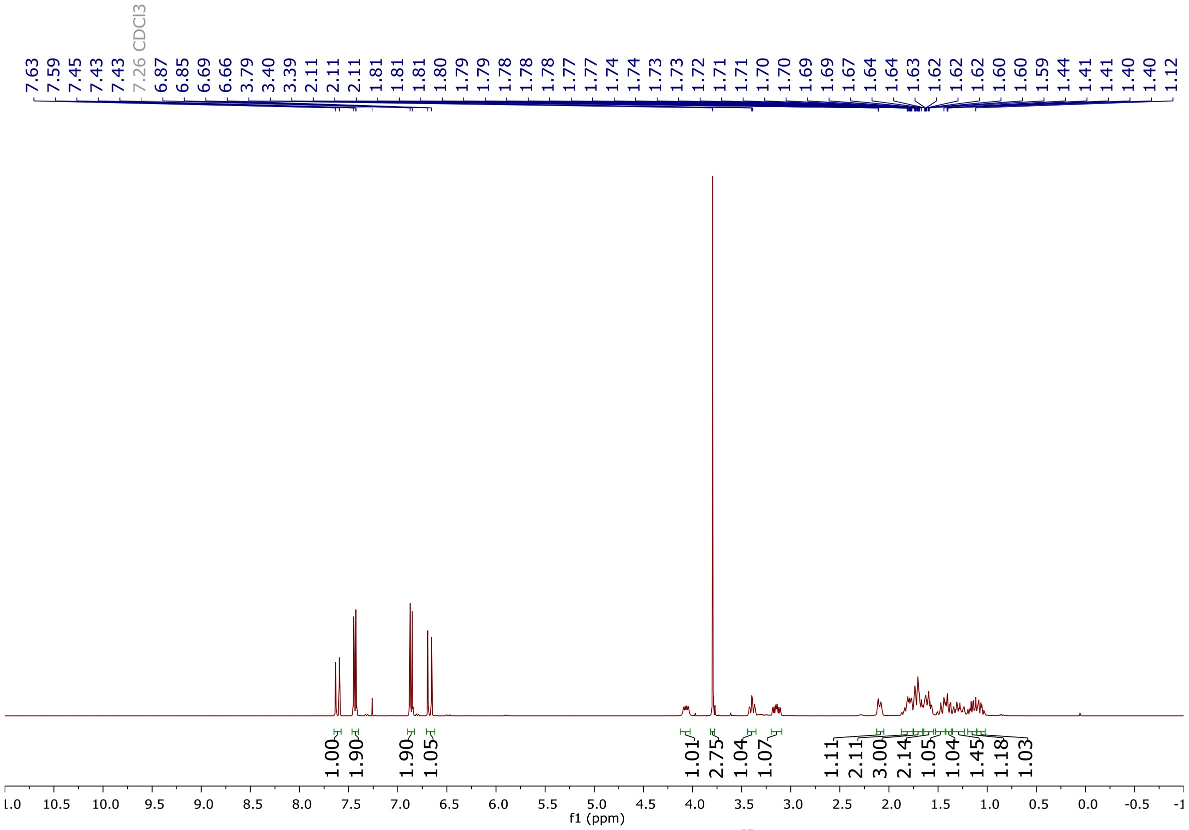

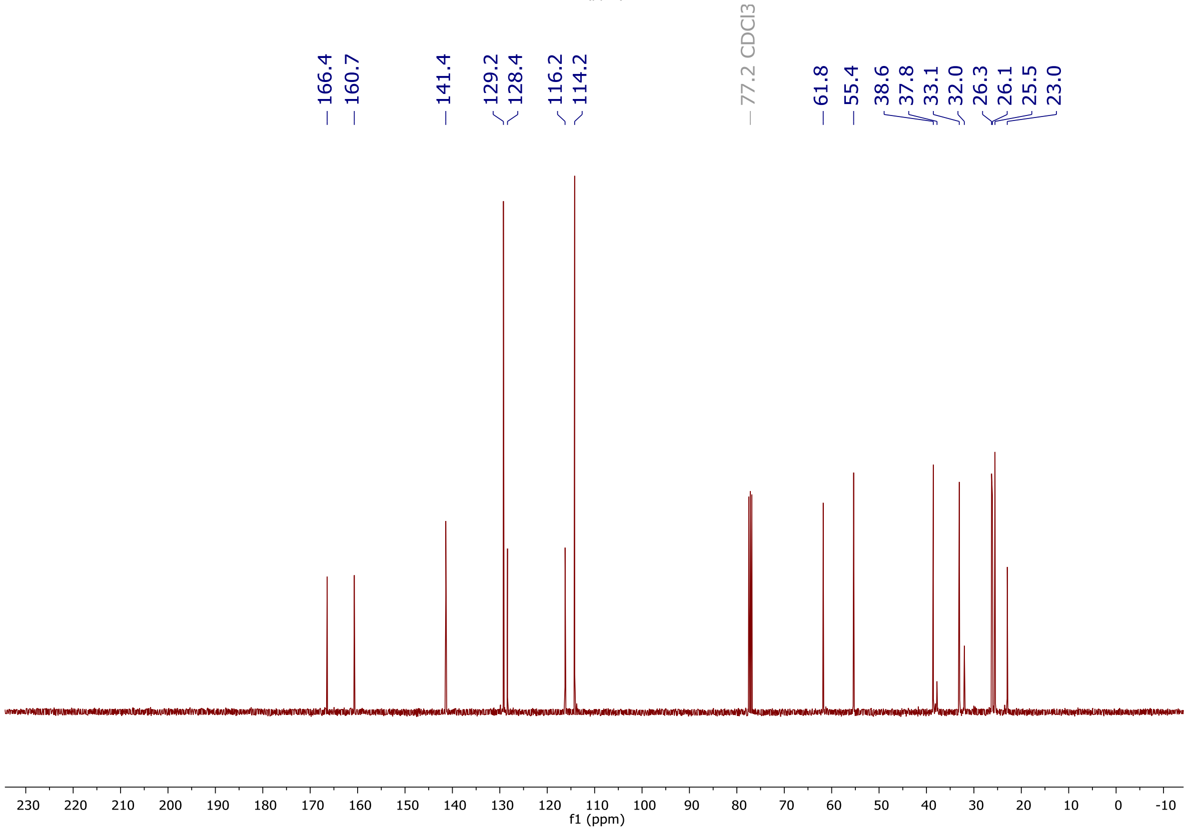

4-((*Z*)-3-Oxo-3-((*1R,3r,5S*)-3-((triethylsilyl)oxy)-8-azabicyclo[3.2.1]octan-8-yl)prop-1-en-1-yl)benzonitrile (13q)

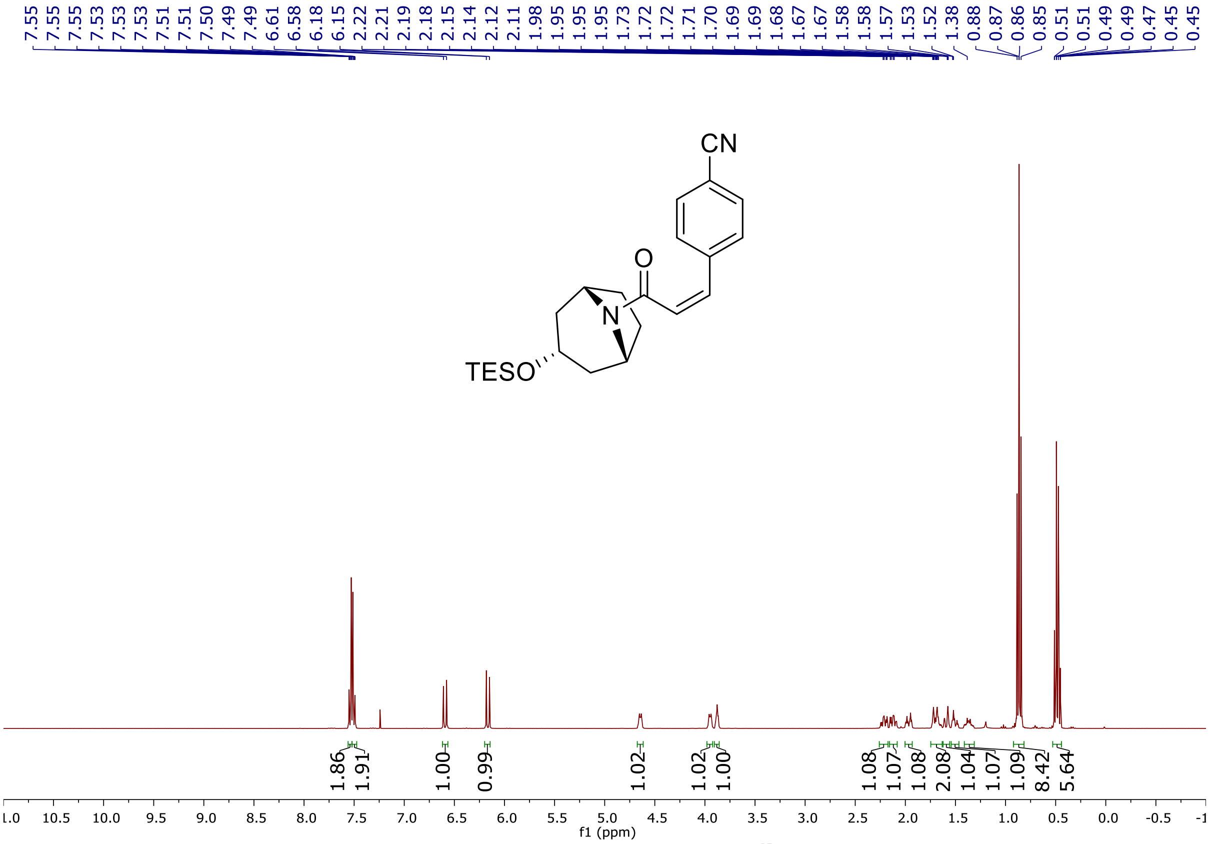

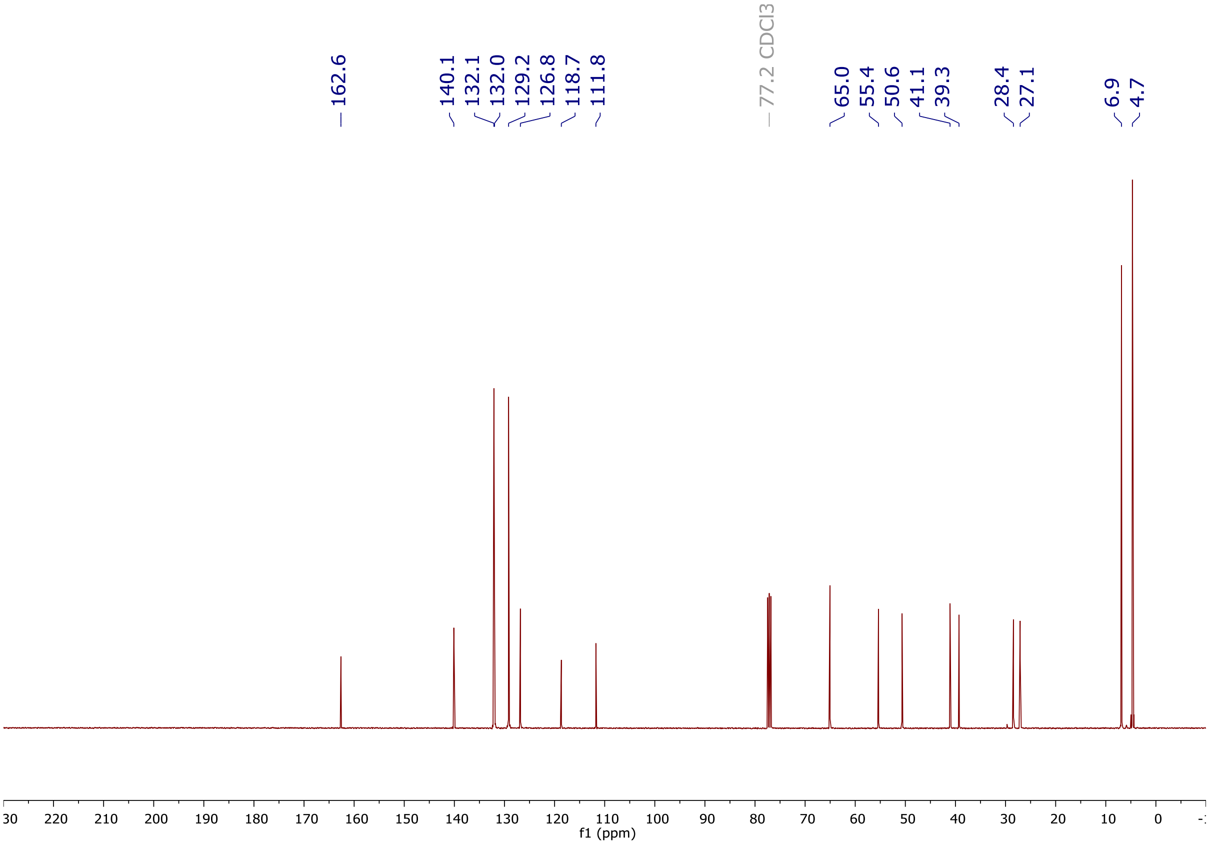

_N_,_N_-Dibutyl-3-oxocyclohex-1-enecarboxamide (13r)

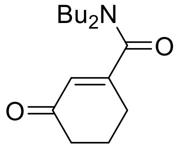

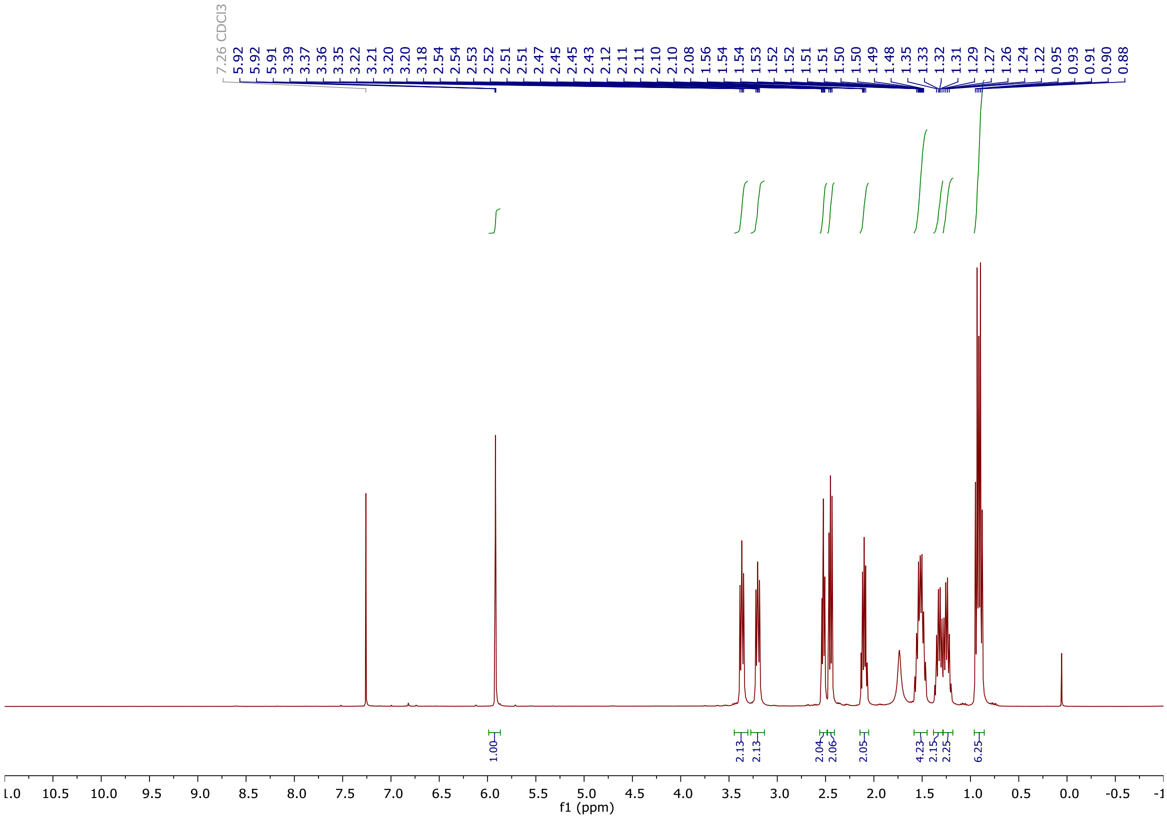

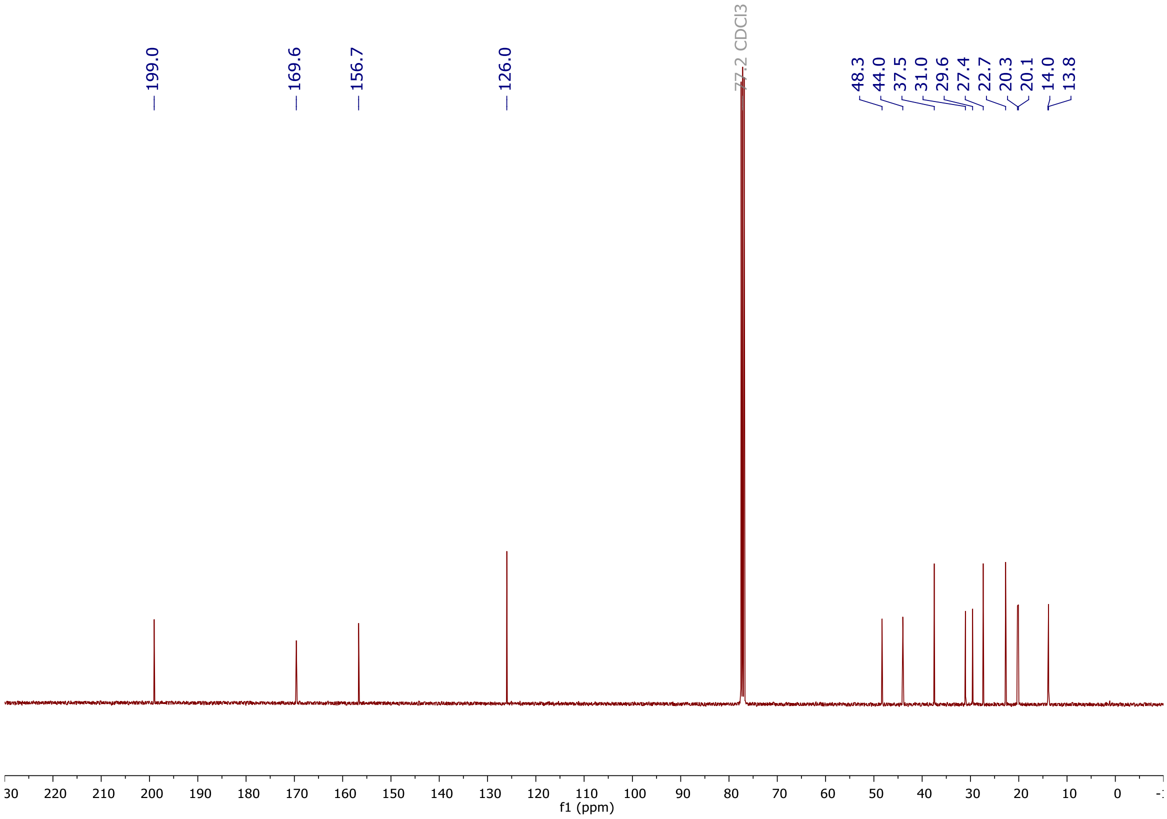

(*Z*)-Methyl 4-(dibutylamino)-4-oxobut-2-enoate (13s)

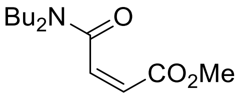

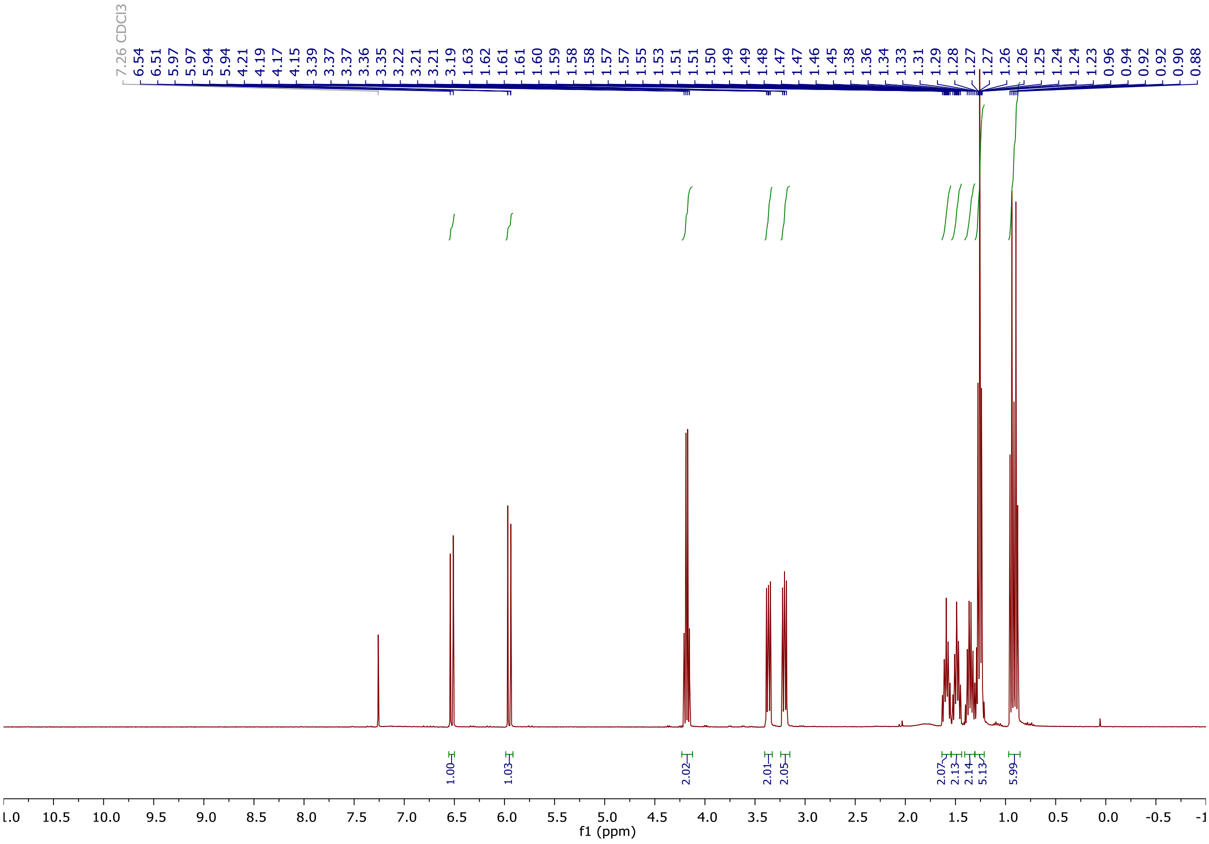

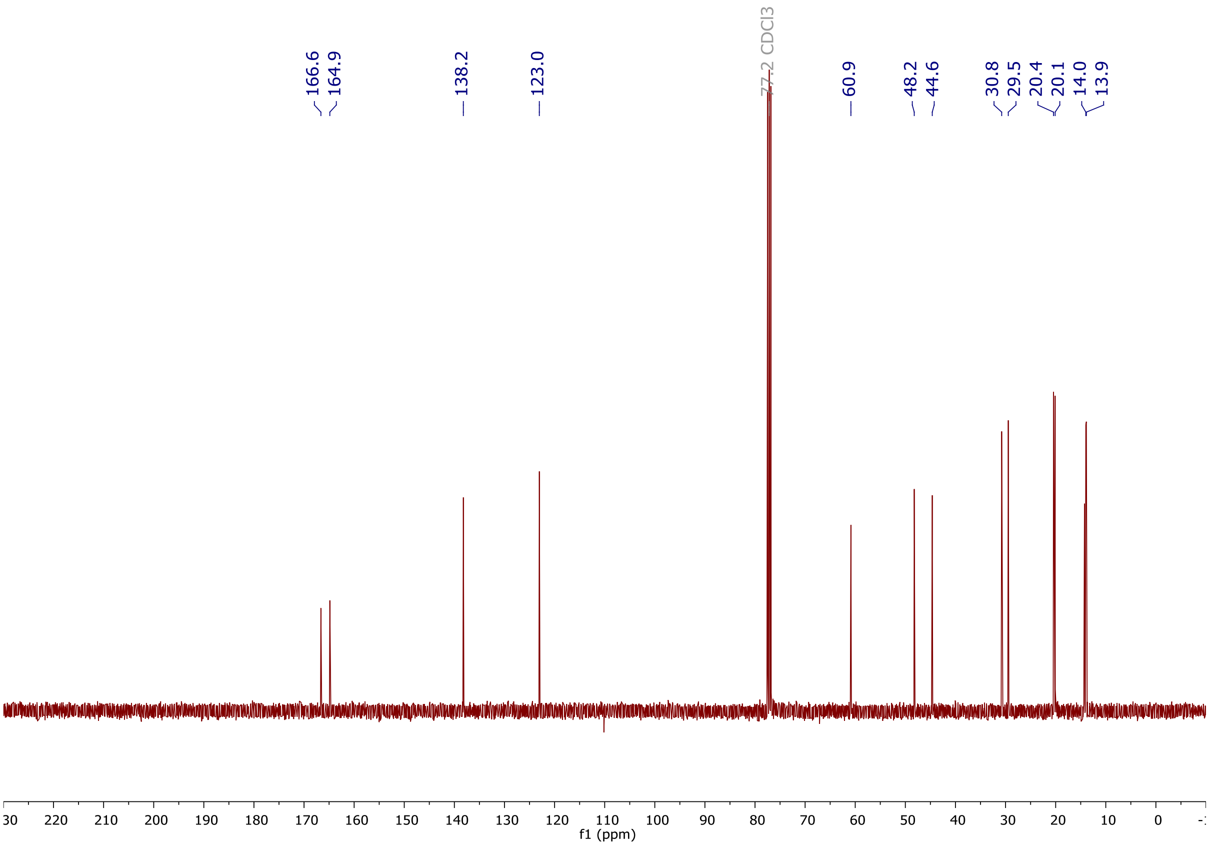

NMR characterization of carbamoylzinc reagents:

Metalation in d8-THF with TMPLi (0.5 equiv $ZnCl_2$ compared to *N*,*N*-dibutylformamide **4a**):

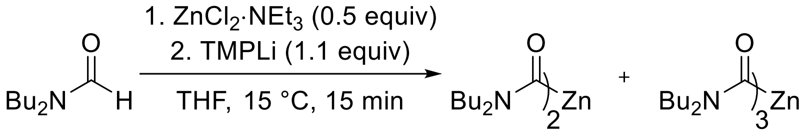

Sample preparation:

In a clean, flame dried, argon filled flask was added *n*-BuLi 1.72 M in *n*-hexane (320 µL, 0.55 mmol). High vacuum was slowly released (water bath 25 °C). After ca. 2 h crystals of *n*-BuLi were observed on the walls of the flask. Flask was placed into -78 °C acetone bath (stirring stopped due to freezing of *n*-BuLi slime on the bottom of the flask). d_8-THF ampule was opened and solvent quickly taken into argon flushed syringe. d_8-THF was dropwise added to *n*-BuLi. To ensure stirring the flask was shaken gently outside of the acetone bath till stirring bar was released from the bottom. When stirring was ensured, TMPH (72 mg, 0.5 mmol) was added in one portion and mixture stirred for 4 h to obtain white suspension of TMPLi. Then flask was transferred into ice/water bath and stirred for 15 min (yellowish solution obtained).
In another a clean, flame dried, argon filled flask was added $ZnCl_2$ 1M in THF (0.2 mL, 0.2 mmol) and solvent removed under high vacuum (over 1 h). To the $ZnCl_2$ powder was added d_8-THF (0.75 mL). To the solution was added Bu_2NCHO (64 mg, 0.4 mmol, 1.0 equiv) and reaction mixture tempered to 15 °C. Solution of TMPLi in d8-THF was dropwise added to the solution of $Bu_2NCHO/ZnCl_2$ in d8-THF at 15 °C and reaction mixture was stirred for additional 15 min. Into the flame dried and argon flushed NMR tube 1 mL of the solution was transferred

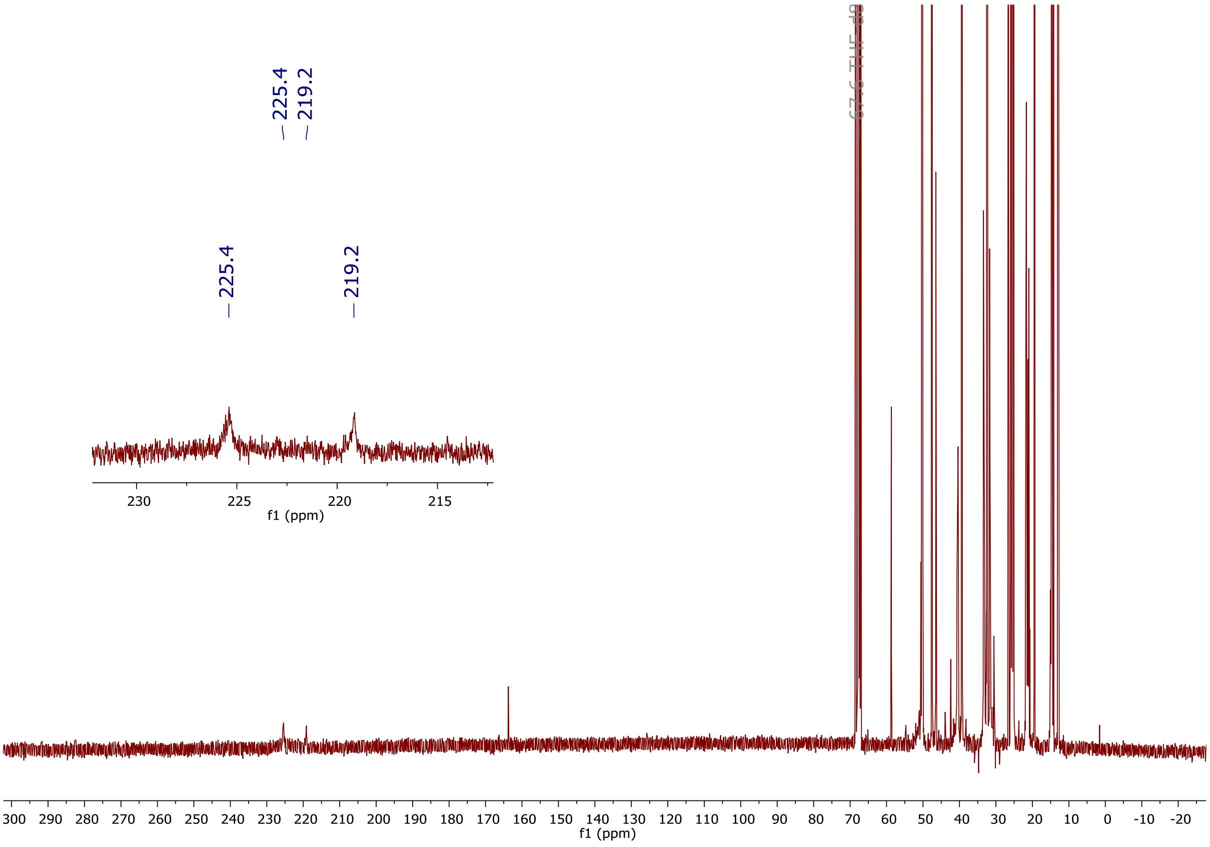

Metalation in THF with capillary d6-benzene with $TMP_2Zn\cdot 2LiCl$ (0.55 equiv compared to *N,N*-dibutylformamide **4a**):

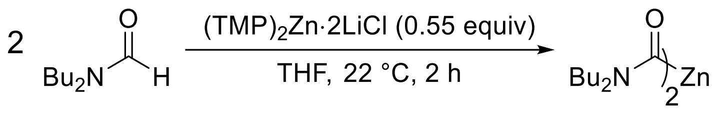

Sample preparation:

A heat and vacuum dried flask flushed with argon was placed into acetone/dry ice bath at -78 °C. Tetramethylpiperidine (184 mg, 1.3 mmol) was dissolved in THF (2 mL) and cooled to –78 °C. Then, *n*-BuLi 1.63 M in hexane (0.8 mL, 1.3 mmol) was dropwise added and the reaction allowed to warm to 0 °C over 1-2 h. Then $ZnCl_2$ (600 µL, 0.6 mmol, 0.6 equiv) and mixture was stirred for 30 minutes to produce $TMP_2Zn\cdot 2LiCl$. Neat *N*,*N*-dibutylformamide (1.00 mmol, 1.0 equiv) was added and mixture stirred for additional 2 h. Into the flame dried and argon flushed NMR tube equipped with capillary C_6D_6 1 mL of the solution was transferred.

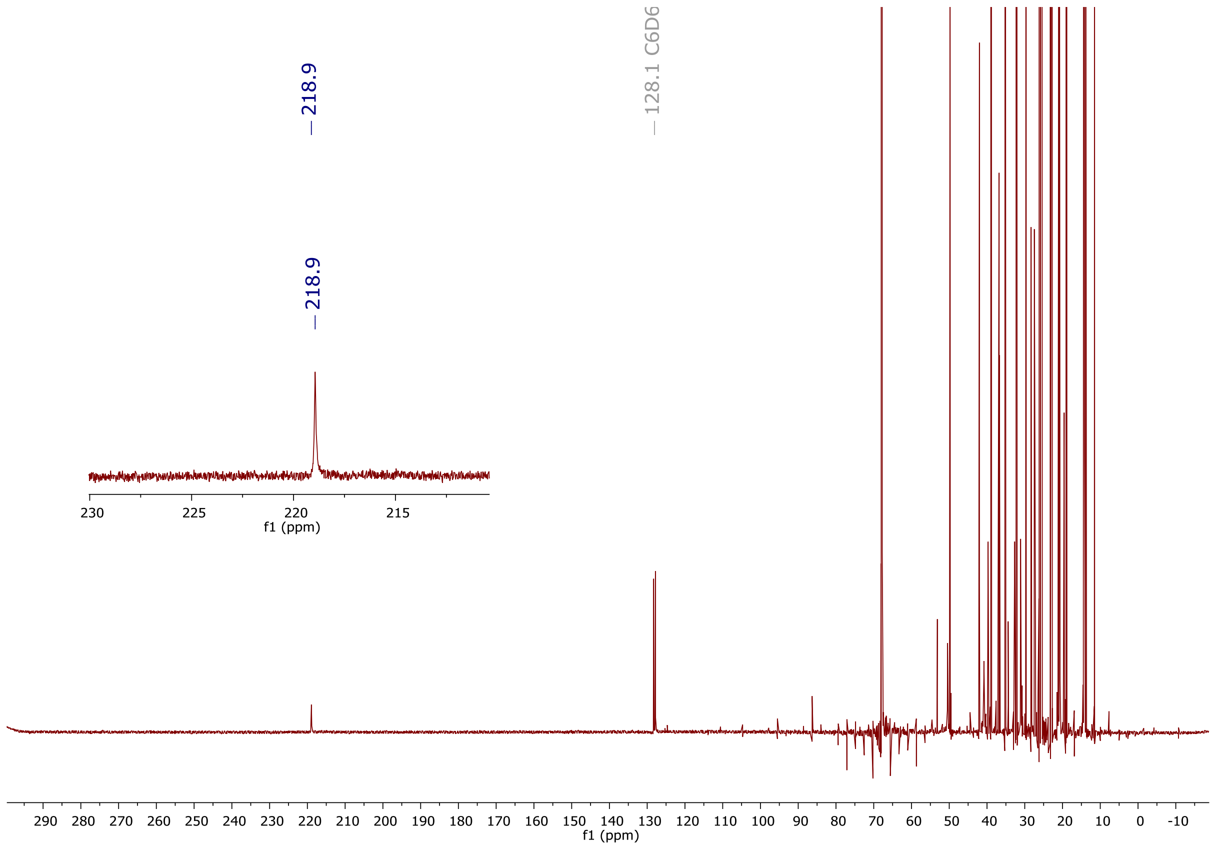

Metalation in THF with capillary d6-benzene with TMPLi (0.3 equiv $ZnCl_2$ compared to *N,N*-dibutylformamide **4a**):

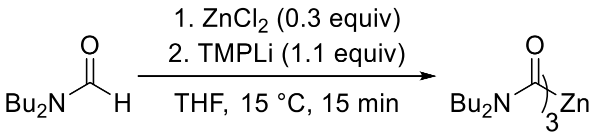

Sample preparation:

A heat and vacuum dried flask flushed with argon was placed into ice/water bath at 0 °C. Formamide (1.00 mmol, 1.0 equiv), $ZnCl_2$ solution 1 M in THF (330 µL, 0.330 mmol, 0.33 equiv), Et_3N (51.0 mg, 0.500 mmol, 0.5 equiv) and THF (2 mL) were added. Freshly prepared TMPLi as ca. 0.5 M solution in THF (1.2 mmol, 1.2 equiv) was dropwise added over 1-2 minutes. Reaction mixture was stirred for additional 15 minutes. Into the flame dried and argon flushed NMR tube equipped with capillary C_6D_6 1 mL of the solution was transferred.

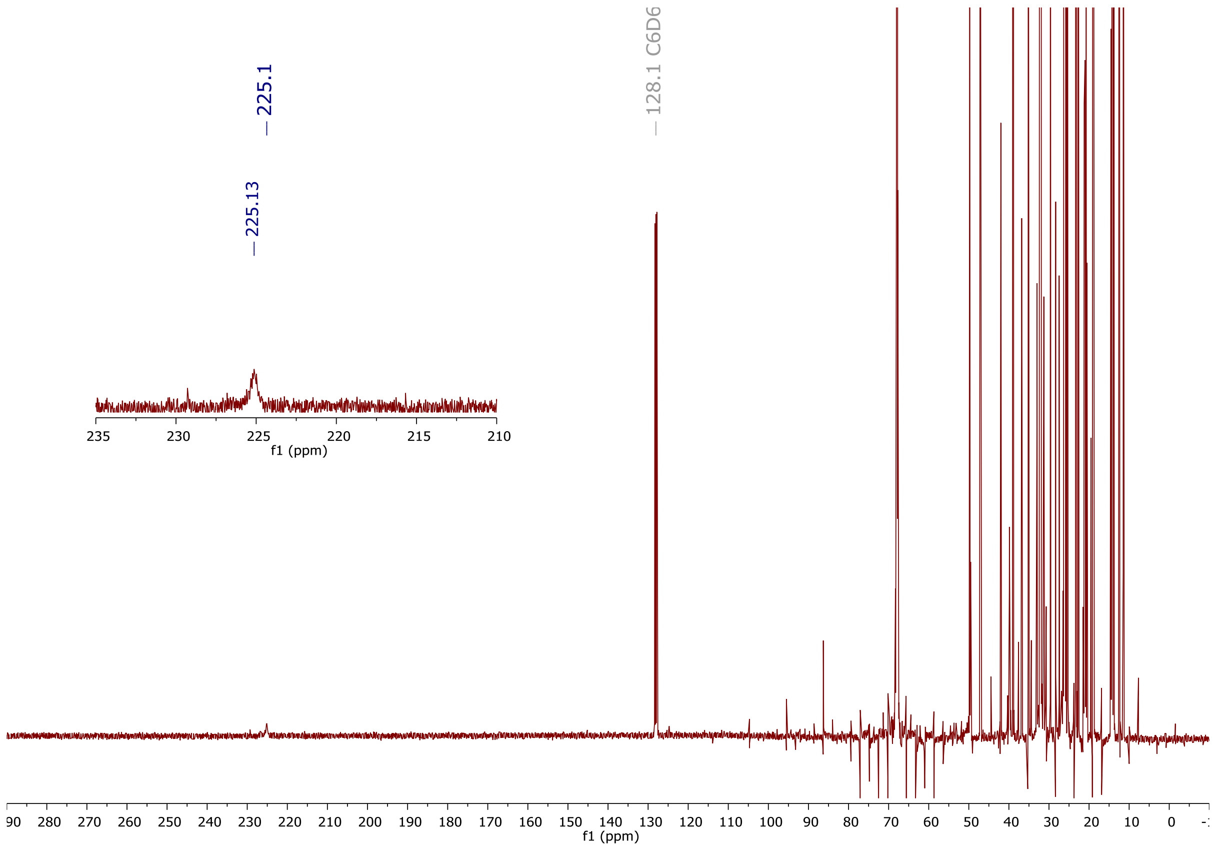

Transmetalation of *N*,*N*-dibutylcarbamoyllithium with 0.3 equiv $ZnCl_2$

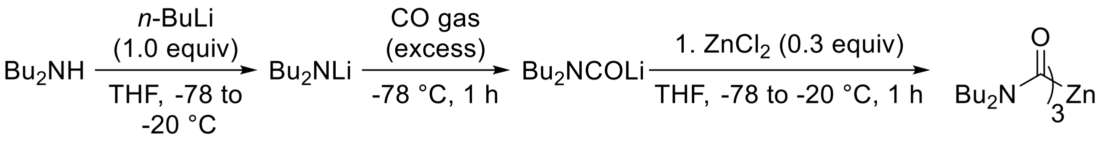

A heat and vacuum dried two-necked Schlenk flask flushed with argon was filled with Bu_2NH (10.0 mmol, 1.0 equiv) as 0.5 M solution in THF and cooled to −78 °C. After dropwise addition of *n*-BuLi (10 mmol, 1.0 equiv) 1.6 M in hexane the mixture was allowed to slowly warm to −20 °C. The mixture was again cooled to −78 °C and CO gas was introduced (yellow color developed) into the flask *via* tubing through quick-fit attachment. CO atmosphere was maintained for 1 h and then $ZnCl_2$ (0.3 equiv) was added. Reaction mixture was stirred for 1 h (allowed to slowly warm up) to enable full transmetalation. Into the flame dried and argon flushed NMR tube equipped with capillary C_6D_6 1 mL of the solution was transferred.

CAUTION: Working with highly toxic CO gas should be done only with proper equipment, validated CO-gas detector and by a trained chemist.

Note: Reaction of aliquots with allyl bromide in the presence of CuCN·2LiCl afforded **8a** as the sole product.

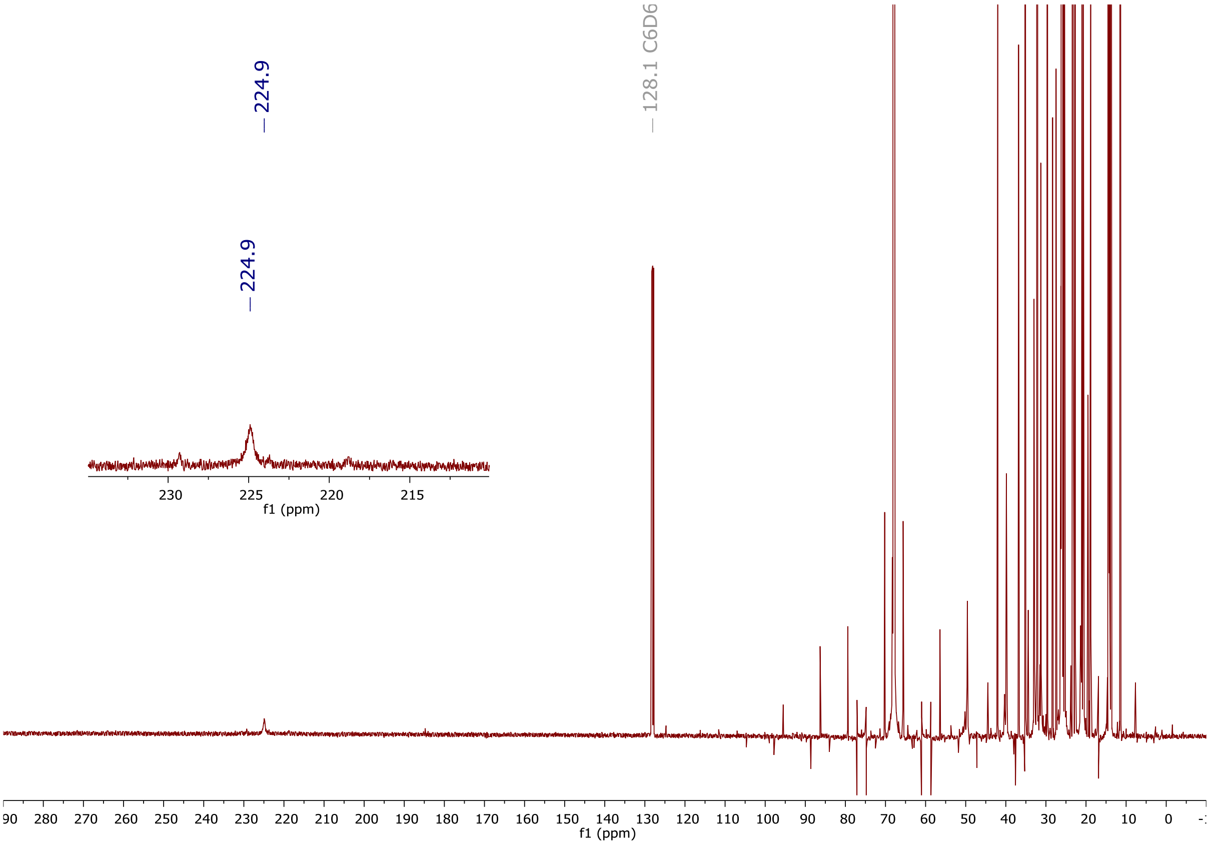

Transmetalation of *N,N*-dibutylcarbamoyllithium with 0.5 equiv $ZnCl_2$

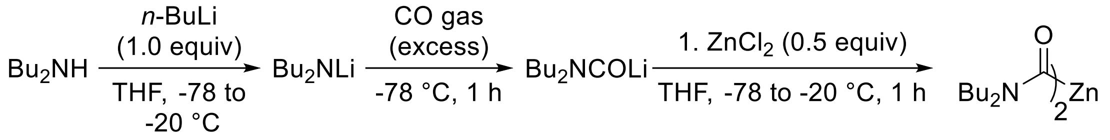

A heat and vacuum dried two-necked Schlenk flask flushed with argon was filled with Bu_2NH (10.0 mmol, 1.0 equiv) as 0.5 M solution in THF and cooled to –78 °C. After dropwise addition of *n*-BuLi (10 mmol, 1.0 equiv) 1.6 M in hexane the mixture was allowed to slowly warm to –20 °C. The mixture was again cooled to –78 °C and CO gas was introduced (yellow color developed) into the flask *via* tubing through quick-fit attachment. CO atmosphere was maintained for 1 h and then $ZnCl_2$ (0.5 equiv) was added. Reaction mixture was stirred for 1 h (allowed to slowly warm up) to enable full transmetalation. Into the flame dried and argon flushed NMR tube equipped with capillary C_6D_6 1 mL of the solution was transferred.

CAUTION: Working with highly toxic CO gas should be done only with proper equipment, validated CO-gas detector and by a trained chemist.

Note: Reaction of aliquots with allyl bromide in the presence of CuCN·2LiCl afforded **8a** as the sole product.

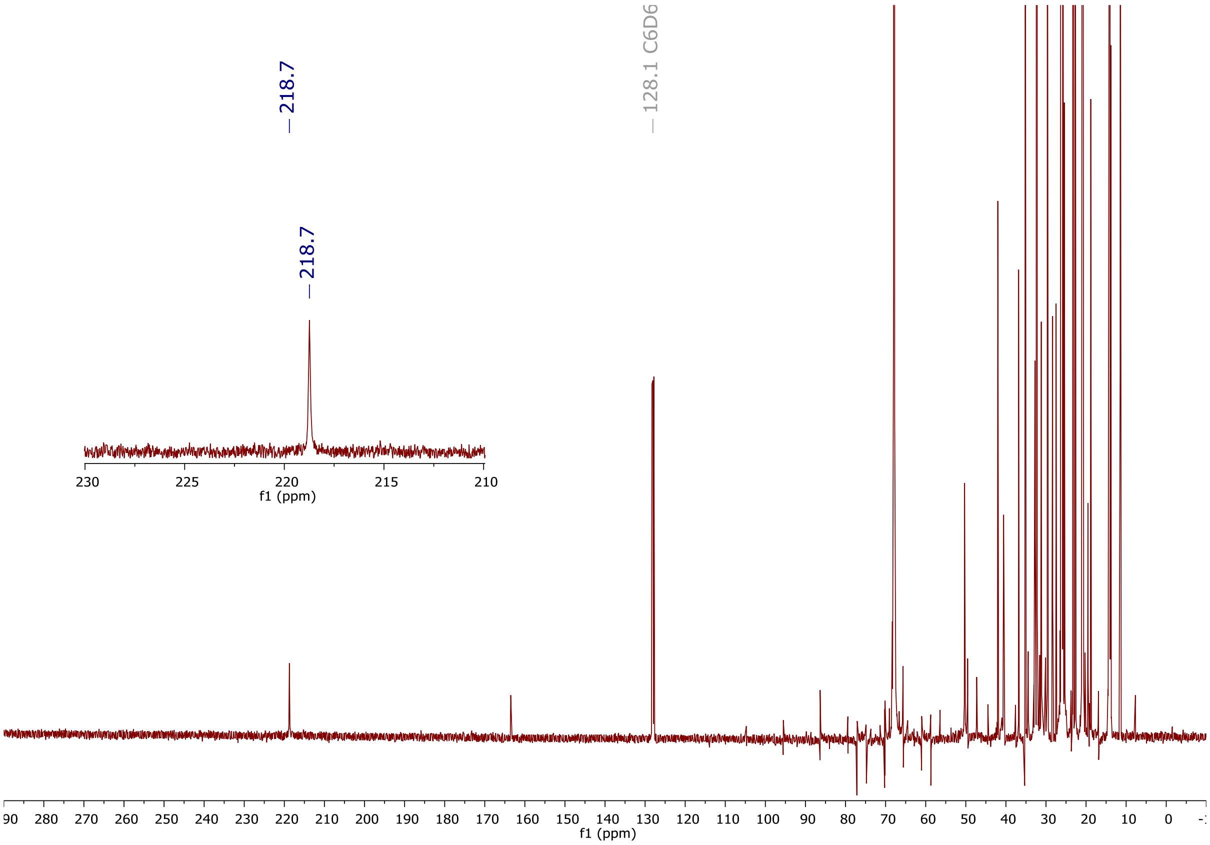

Formation of tentative monocarbamoylzinc reagent by addition of +1 equiv $ZnCl_2$ to the mixture of dicarbamoylzinc and tricarbamoylzinc:

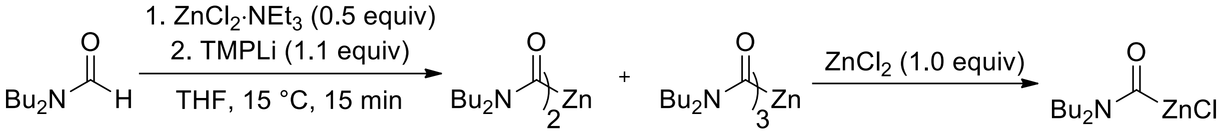

In a clean, flame dried, argon filled flask was added *n*-BuLi 1.72 M in *n*-hexane (320 µL, 0.55 mmol). High vacuum was slowly released (water bath 25 °C). After ca. 2 h crystals of *n*-BuLi were observed on the walls of the flask. Flask was placed into -78 °C acetone bath (stirring stopped due to freezing of *n*-BuLi slime on the bottom of the flask). d_8-THF ampule was opened and solvent quickly taken into argon flushed syringe. d_8-THF was dropwise added to *n*-BuLi. To ensure stirring the flask was shaken gently outside of the acetone bath till stirring bar was released from the bottom. When stirring was ensured, TMPH (72 mg, 0.5 mmol) was added in one portion and mixture stirred for 4 h to obtain white suspension of TMPLi. Then flask was transferred into ice/water bath and stirred for 15 min (yellowish solution obtained).

In another a clean, flame dried, argon filled flask was added $ZnCl_2$ 1M in THF (0.5 mL, 0.5 mmol) and solvent removed under high vacuum (over 1 h). To the $ZnCl_2$ powder was added d8-THF (0.75 mL). To the solution was added Bu_2NCHO (64 mg, 0.4 mmol, 1.0 equiv) and reaction mixture tempered to 15 °C. Solution of TMPLi in d8-THF was dropwise added to the solution of $Bu_2NCHO/ZnCl_2$ in d8-THF at 15 °C and reaction mixture was stirred for additional 15 min.

The solution of carbamoylzinc reagent was transferred to the new flask containing dry $ZnCl_2$ powder (0.4 mmol, 0.4 equiv) (obtained by evaporating the THF solution of $ZnCl_2$ under high vacuum). Into the flame dried and argon flushed NMR tube 1 mL of the solution was transferred.

Note: HMBC experiment was performed at 0 °C.

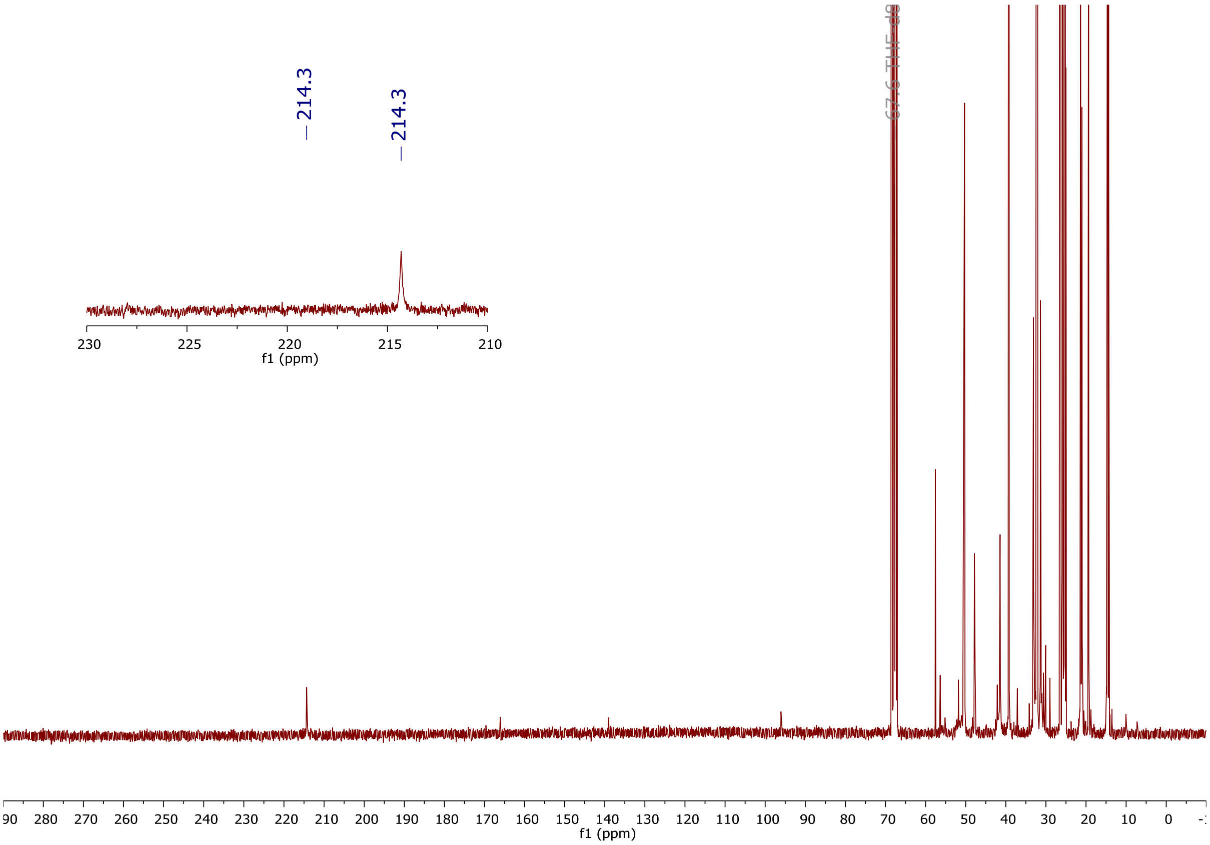

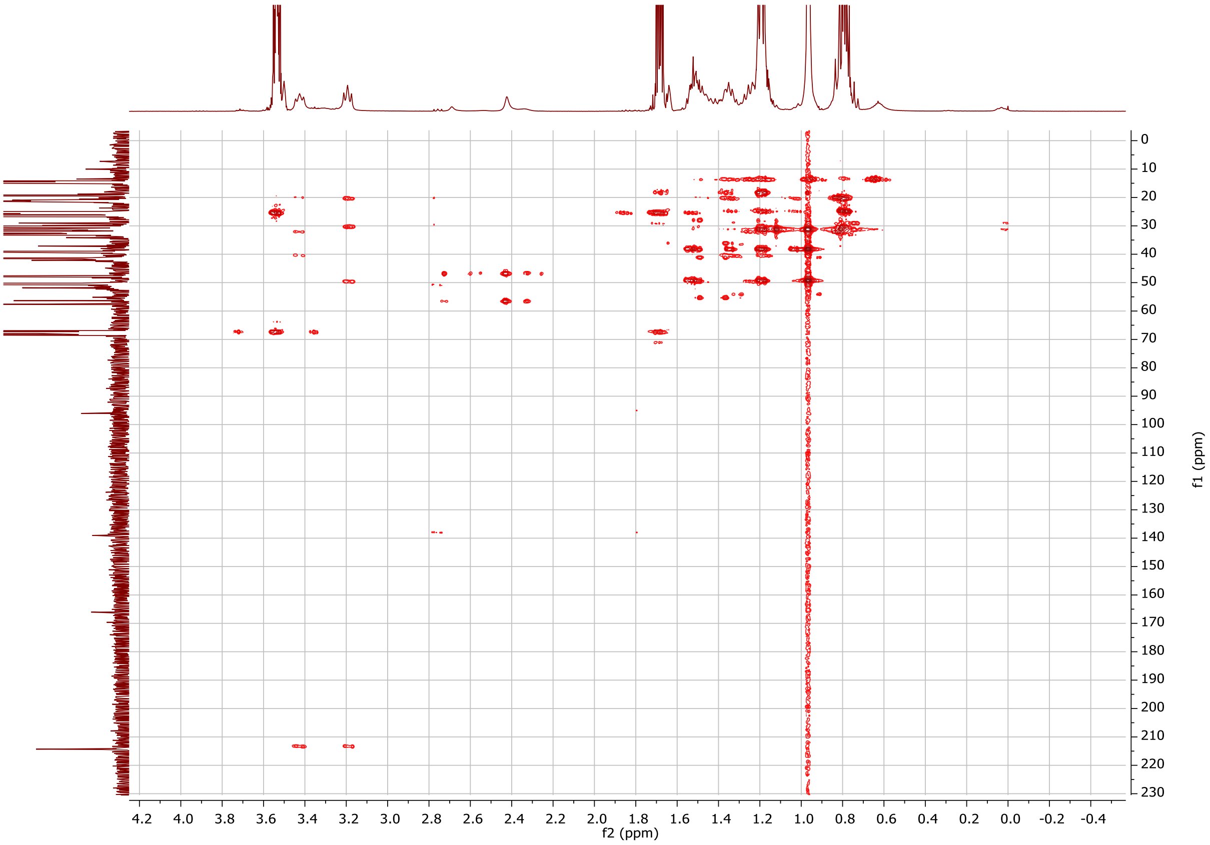

4.2
4.0
3.8
3.6
3.4
3.2
3.0
2.8
2.6
2.4
2.2
2.0
1.8
1.6
1.4
1.2
1.0
0.8
0.6
0.4
0.2
0.0
-0.2
-0.4
f2 (ppm)
0
10
20
30
40
50
60
70
80
90
100
110
120
130
140
150
160
170
180
190
200
210
220
230
f1 (ppm)

Single Crystal X-Ray Diffraction Studies

Single crystals of compound **4m**, suitable for X-ray diffraction, were obtained by slow evaporation of **ethyl acetate** solution. The crystals were introduced into perfluorinated oil and a suitable single crystal was carefully mounted on the top of a thin glass wire. Data collection was performed with an Oxford Xcalibur 3 diffractometer equipped with a Spellman generator (50 kV, 40 mA) and a Kappa CCD detector, operating with Mo-K_α radiation (λ = 0.71071 Å).

Data collection and data reduction were performed with the CrysAlisPro software.[8] Absorption correction using the multiscan method[8] was applied. The structures were solved with SHELXS-97,[9] refined with SHELXL-97[10] and finally checked using PLATON.[11] Details for data collection and structure refinement are summarized in Table 1.

CCDC-**2164155** contains supplementary crystallographic data for this compound. These data can be obtained free of charge from The Cambridge Crystallographic Data Centre *via* www.ccdc.cam.ac.uk/data_request/cif.

---

[8] Program package 'CrysAlisPro 1.171.40.84a (Rigaku OD, 2020)'.
[9] Sheldrick, G. M. (1997) SHELXS-97: *Program for Crystal Structure Solution*, University of Göttingen, Germany.
[10] Sheldrick, G. M. (1997) SHELXL-97: *Program for the Refinement of Crystal Structures*, University of Göttingen, Germany.
[11] Spek, A. L. (1999) PLATON: *A Multipurpose Crystallographic Tool*, Utrecht University, Utrecht, The Netherlands.

Table 1. Details for X-ray data collection and structure refinement for compound **4m**.

	**4m**
Empirical formula	$C_{15}H_{18}N_2O_3$
Formula mass	274.31
T[K]	123(2)
Crystal size [mm]	0.40 × 0.30 × 0.25
Crystal description	colorless block
Crystal system	monoclinic
Space group	*P*21/*c*
a [Å]	8.0549(4)
b [Å]	16.1122(7)
c [Å]	11.0039(6)
α [°]	90.0
β [°]	103.830(5)
γ [°]	90.0
V [$Å^3$]	1386.71(12)
Z	4
$\rho_{calcd.}$ [g cm^{-3}]	1.314
μ [mm^{-1}]	0.092
F(000)	584
Θ range [°]	2.29 – 25.24
Index ranges	$-11 \leq h \leq 11$
	$-23 \leq k \leq 23$
	$-15 \leq l \leq 15$
Reflns. collected	28469
Reflns. obsd.	3488
Reflns. unique	4238 (R_{int} = 0.0323)
R_1, wR_2 (2σ data)	0.0445, 0.1106
R_1, wR_2 (all data)	0.0553, 0.1179
GOOF on F^2	1.027
Peak/hole [e $Å^{-3}$]	0.392 / -0.181

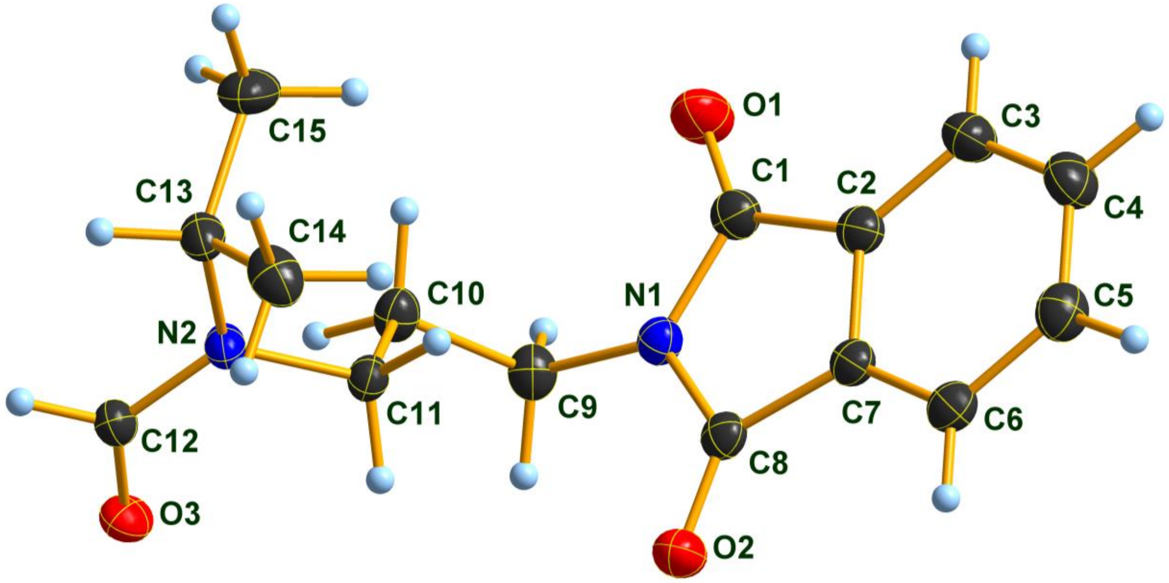

Figure 1. Molecular structure of compound **4m** in the crystal. DIAMOND[12] representation; thermal ellipsoids are drawn at 50 % probability level.

Table 2. Selected bond lengths (Å) of compound **4m**.

O2 – C8	1.208(1)	C13 – C15	1.521(2)
N1 – C1	1.392(1)	C13 – C14	1.523(2)
N1 – C8	1.394(1)	C6 – C5	1.398(2)
N1 – C9	1.459(1)	C3 – C4	1.394(2)
C10 – C9	1.520(2)	C5 – C4	1.391(2)
C10 – C11	1.525(1)	C7 – C6	1.381(1)
N2 – C12	1.341(1)	C7 – C2	1.389(1)
N2 – C11	1.462(1)	C7 – C8	1.489(1)
N2 – C13	1.469(1)	O3 – C12	1.233(1)
C1 – O1	1.207(1)	C2 – C3	1.379(2)
C1 – C2	1.488(2)		

Table 3. Selected bond angles (°) of compound **4m**.

C1 – N1 – C8	112.0(1)	C7 – C6 – C5	117.0(1)
C1 – N1 – C9	123.6(1)	N2 – C11 – C10	112.5(1)
C8 – N1 – C9	124.3(1)	O3 – C12 – N2	124.6(1)
C9 – C10 – C11	112.3(1)	C2 – C3 – C4	117.4(1)

[12] DIAMOND, Crystal Impact GbR., Version 3.2i.

N1 – C9 – C10	113.5(1)	C4 – C5 – C6	121.2(1)
C12 – N2 – C11	118.7(1)	C5 – C4 – C3	121.2(1)
C12 – N2 – C13	120.6(1)	C3 – C2 – C1	130.5(1)
C11 – N2 – C13	120.6(1)	C7 – C2 – C1	108.2(1)
O1 – C1 – N1	124.5(1)	O2 – C8 – N1	124.6(1)
O1 – C1 – C2	129.6(1)	O2 – C8 – C7	129.4(1)
N1 – C1 – C2	105.9(1)	N1 – C8 – C7	106.0(1)
C6 – C7 – C2	121.9(1)	N2 – C13 – C15	111.1(1)
C6 – C7 – C8	130.2(1)	N2 – C13 – C14	111.3(1)
C2 – C7 – C8	107.9(1)	C15 – C13 – C14	111.7(1)
C3 – C2 – C7	121.4(1)		

Table 4. Selected torsion angles (°) of compound **4m**.

C1 – N1 – C9 – C10	-85.2(1)	C2 – C7 – C8 – O2	178.3(1)
C8 – N1 – C9 – C10	98.2(1)	C6 – C7 – C8 – N1	178.1(1)
C11 – C10 – C9 – N1	-61.3(1)	C2 – C7 – C8 – N1	-0.9(1)
C8 – N1 – C1 – O1	179.5(1)	C12 – N2 – C13 – C15	-120.8(1)
C9 – N1 – C1 – O1	2.5(2)	C11 – N2 – C13 – C15	62.3(1)
C8 – N1 – C1 – C2	0.0(1)	C12 – N2 – C13 – C14	114.1(1)
C9 – N1 – C1 – C2	-177.0(1)	C11 – N2 – C13 – C14	-62.9(1)
C6 – C7 – C2 – C3	1.0(2)	C2 – C7 – C6 – C5	-0.2(2)
C8 – C7 – C2 – C3	-179.9(1)	C8 – C7 – C6 – C5	-179.2(1)
C6 – C7 – C2 – C1	-178.2(1)	C12 – N2 – C11 – C10	79.5(1)
C8 – C7 – C2 – C1	0.9(1)	C13 – N2 – C11 – C10	-103.5(1)
O1 – C1 – C2 – C3	0.9(2)	C9 – C10 – C11 – N2	-168.4(1)
N1 – C1 – C2 – C3	-179.7(1)	C11 – N2 – C12 – O3	-1.6(2)
O1 – C1 – C2 – C7	180.0(1)	C13 – N2 – C12 – O3	-178.6(1)
N1 – C1 – C2 – C7	-0.6(1)	C7 – C2 – C3 – C4	-0.8(2)
C1 – N1 – C8 – O2	-178.7(1)	C1 – C2 – C3 – C4	178.2(1)
C9 – N1 – C8 – O2	-1.7(2)	C7 – C6 – C5 – C4	-0.6(2)
C1 – N1 – C8 – C7	0.6(1)	C6 – C5 – C4 – C3	0.8(2)
C9 – N1 – C8 – C7	177.5(1)	C2 – C3 – C4 – C5	0.0(2)
C6 – C7 – C8 – O2	-2.7(2)		

Single crystals of compound **4p**, suitable for X-ray diffraction, were obtained by slow

evaporation of **$CDCl_3$** solution. The crystals were introduced into perfluorinated oil and a suitable single crystal was carefully mounted on the top of a thin glass wire. Data collection was performed with an Oxford Xcalibur 3 diffractometer equipped with a Spellman generator (50 kV, 40 mA) and a Kappa CCD detector, operating with Mo-K_α radiation (λ = 0.71071 Å).

Data collection and data reduction were performed with the CrysAlisPro software.[8] Absorption correction using the multiscan method[8] was applied. The structures were solved with SHELXS-97,[9] refined with SHELXL-97[10] and finally checked using PLATON.[11] Details for data collection and structure refinement are summarized in Table 1.

CCDC-**2164152** contains supplementary crystallographic data for this compound. These data can be obtained free of charge from The Cambridge Crystallographic Data Centre *via* www.ccdc.cam.ac.uk/data_request/cif.

Table 5. Details for X-ray data collection and structure refinement for compound **4p**.

	**4p**
Empirical formula	$C_{12}H_{13}N_3O$
Formula mass	215.25
T[K]	123(2)
Crystal size [mm]	0.40 × 0.40 × 0.05
Crystal description	colorless platelet
Crystal system	monoclinic
Space group	*P*21/*c*
a [Å]	9.4066(5)
b [Å]	11.9350(5)
c [Å]	9.7498(5)
α [°]	90.0
β [°]	101.214(5)
γ [°]	90.0
V [$Å^3$]	1073.69(9)
Z	4
$\rho_{calcd.}$ [g cm^{-3}]	1.332
μ [mm^{-1}]	0.088
F(000)	456
Θ range [°]	2.21 – 25.24
Index ranges	$-12 \leq h \leq 12$
	$-15 \leq k \leq 15$
	$-12 \leq l \leq 12$
Reflns. collected	18754
Reflns. obsd.	2128
Reflns. unique	2660 (R_{int} = 0.0355)
R_1, wR_2 (2σ data)	0.0400, 0.1028
R_1, wR_2 (all data)	0.0531, 0.1116
GOOF on F^2	1.043
Peak/hole [e $Å^{-3}$]	0.266 / -0.179

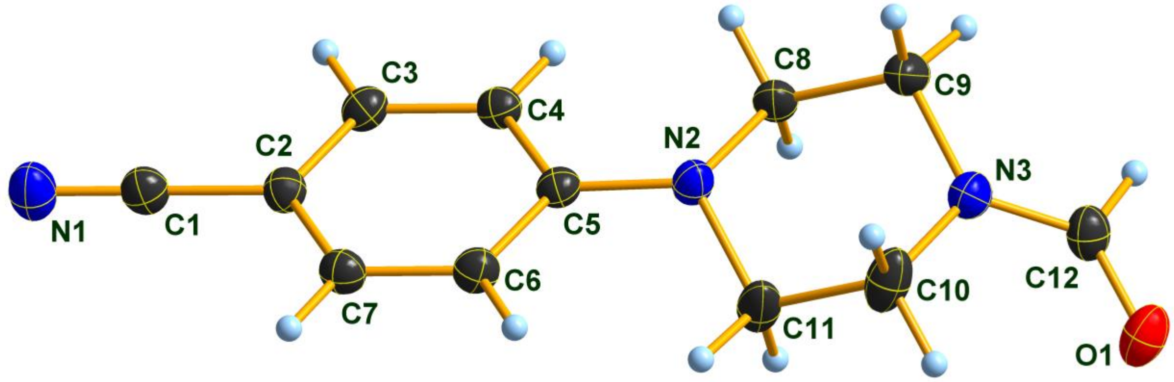

Figure 2. Molecular structure of compound **4p** in the crystal. DIAMOND[12] representation; thermal ellipsoids are drawn at 50 % probability level.

Table 6. Selected bond lengths (Å) of compound **4p**.

C5 – N2	1.396(2)	C8 – N2	1.464(2)
C5 – C4	1.411(2)	C8 – C9	1.524(2)
C5 – C6	1.413(2)	N2 – C11	1.472(2)
C1 – N1	1.150(2)	C10 – C11	1.517(2)
C1 – C2	1.437(2)	N3 – C12	1.340(2)
O1 – C12	1.223(2)	N3 – C10	1.453(2)
C2 – C3	1.397(2)	N3 – C9	1.455(2)
C2 – C7	1.400(2)	C6 – C7	1.377(2)
C3 – C4	1.385(2)		

Table 7. Selected bond angles (°) of compound **4p**.

N2 – C5 – C4	121.8(1)	C5 – N2 – C8	118.5(1)
N2 – C5 – C6	120.4(1)	C5 – N2 – C11	117.6(1)
C4 – C5 – C6	117.8(1)	C8 – N2 – C11	111.2(1)
N1 – C1 – C2	178.2(1)	N3 – C10 – C11	109.9(1)
C3 – C2 – C7	119.1(1)	N3 – C9 – C8	111.3(1)
C3 – C2 – C1	120.8(1)	O1 – C12 – N3	125.8(1)
C7 – C2 – C1	120.1(1)	N2 – C11 – C10	111.0(1)
C4 – C3 – C2	120.5(1)	C7 – C6 – C5	121.1(1)
C12 – N3 – C10	122.1(1)	C6 – C7 – C2	120.7(1)
C12 – N3 – C9	123.1(1)	N2 – C8 – C9	110.4(1)
C10 – N3 – C9	114.5(1)	C3 – C4 – C5	120.9(1)

Table 8. Selected torsion angles (°) of compound **4p**.

C7 – C2 – C3 – C4	-0.6(2)	C6 – C5 – N2 – C11	37.8(2)
C1 – C2 – C3 – C4	179.4(1)	C9 – C8 – N2 – C5	162.4(1)
C2 – C3 – C4 – C5	-0.8(2)	C9 – C8 – N2 – C11	-56.6(1)
N2 – C5 – C4 – C3	-176.3(1)	C12 – N3 – C10 – C11	-120.4(1)
C6 – C5 – C4 – C3	1.9(2)	C9 – N3 – C10 – C11	53.3(1)
N2 – C5 – C6 – C7	176.8(1)	C12 – N3 – C9 – C8	120.9(1)
C4 – C5 – C6 – C7	-1.5(2)	C10 – N3 – C9 – C8	-52.7(2)
C5 – C6 – C7 – C2	0.1(2)	N2 – C8 – C9 – N3	53.0(1)
C3 – C2 – C7 – C6	1.0(2)	C10 – N3 – C12 – O1	-4.8(2)
C1 – C2 – C7 – C6	-179.0(1)	C9 – N3 – C12 – O1	-178.0(1)
C4 – C5 – N2 – C8	-5.6(2)	C5 – N2 – C11 – C10	-160.3(1)
C6 – C5 – N2 – C8	176.3(1)	C8 – N2 – C11 – C10	58.4(1)
C4 – C5 – N2 – C11	-144.1(1)	N3 – C10 – C11 – N2	-55.1(1)

Single crystals of compound **8j**, suitable for X-ray diffraction, were obtained by slow

evaporation of **Et_2O** solution. The crystals were introduced into perfluorinated oil and a suitable single crystal was carefully mounted on the top of a thin glass wire. Data collection was performed with an Oxford Xcalibur 3 diffractometer equipped with a Spellman generator (50 kV, 40 mA) and a Kappa CCD detector, operating with Mo-K_α radiation (λ = 0.71071 Ǻ).

Data collection and data reduction were performed with the CrysAlisPro software.[8] Absorption correction using the multiscan method[8] was applied. The structures were solved with SHELXS-97,[9] refined with SHELXL-97[10] and finally checked using PLATON.[11] Details for data collection and structure refinement are summarized in Table 1.

CCDC-**2164153** contains supplementary crystallographic data for this compound. These data can be obtained free of charge from The Cambridge Crystallographic Data Centre *via* www.ccdc.cam.ac.uk/data_request/cif.

Table 9. Details for X-ray data collection and structure refinement for compound **8j**.

	**8j**
Empirical formula	$C_{14}H_{17}NO_2$
Formula mass	231.28
T[K]	123(2)
Crystal size [mm]	0.40 × 0.35 × 0.30
Crystal description	colorless block
Crystal system	monoclinic
Space group	*P*21/*n*
a [Å]	11.9110(8)
b [Å]	7.5895(3)
c [Å]	14.4486(9)
α [°]	90.0
β [°]	111.960(7)
γ [°]	90.0
V [Å^3]	1211.37(13)
Z	4
$\rho_{calcd.}$ [g cm^{-3}]	1.268
μ [mm^{-1}]	0.085
F(000)	496
Θ range [°]	2.79 – 25.24
Index ranges	$-15 \leq h \leq 15$
	$-10 \leq k \leq 10$
	$-19 \leq l \leq 19$
Reflns. collected	20442
Reflns. obsd.	2451
Reflns. unique	2990 (R_{int} = 0.0391)
R_1, wR_2 (2σ data)	0.0442, 0.1043
R_1, wR_2 (all data)	0.0559, 0.1114
GOOF on F^2	1.017
Peak/hole [e Å^{-3}]	0.328 / -0.147

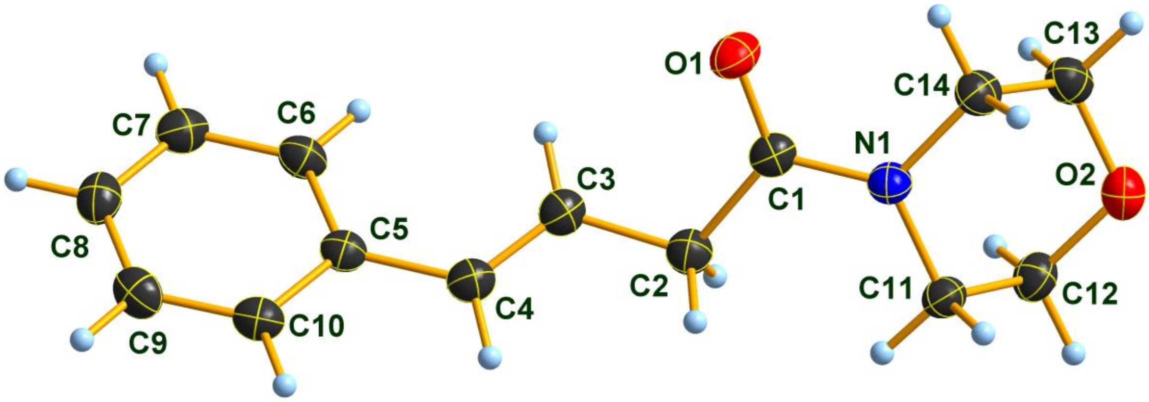

Figure 3. Molecular structure of compound **8j** in the crystal. DIAMOND[12] representation; thermal ellipsoids are drawn at 50 % probability level.

Table 10. Selected bond lengths (Å) of compound **8j**.

C1 – O1	1.230(2)	C8 – C9	1.384(2)
C1 – N1	1.350(2)	C8 – C7	1.389(2)
C1 – C2	1.520(2)	C11 – C12	1.511(2)
N1 – C14	1.463(2)	C10 – C9	1.388(2)
N1 – C11	1.464(2)	C14 – C13	1.517(2)
C2 – C3	1.498(2)	C5 – C10	1.399(2)
O2 – C13	1.425(2)	C5 – C6	1.402(2)
O2 – C12	1.428(2)	C5 – C4	1.473(2)
C3 – C4	1.322(2)	C6 – C7	1.384(2)

Table 11. Selected bond angles (°) of compound **8j**.

O1 – C1 – N1	122.0(1)	C9 – C10 – C5	121.2(1)
O1 – C1 – C2	121.1(1)	C8 – C9 – C10	120.3(1)
N1 – C1 – C2	116.9(1)	C6 – C7 – C8	120.5(1)
C1 – N1 – C14	121.0(1)	N1 – C14 – C13	109.1(1)
C1 – N1 – C11	126.3(1)	O2 – C12 – C11	110.6(1)
C14 – N1 – C11	112.5(1)	O2 – C13 – C14	111.5(1)
C3 – C2 – C1	113.1(1)	C6 – C5 – C4	122.8(1)
C13 – O2 – C12	110.2(1)	C3 – C4 – C5	126.4(1)
C4 – C3 – C2	123.7(1)	C7 – C6 – C5	121.0(1)
C10 – C5 – C6	117.6(1)	C9 – C8 – C7	119.3(1)
C10 – C5 – C4	119.5(1)	N1 – C11 – C12	109.7(1)

Table 12. Selected torsion angles (°) of compound **8j**.

O1 – C1 – N1 – C14	-4.5(2)	C14 – N1 – C11 – C12	-54.7(1)
C2 – C1 – N1 – C14	175.1(1)	C6 – C5 – C10 – C9	-1.3(2)
O1 – C1 – N1 – C11	-178.5(1)	C4 – C5 – C10 – C9	179.3(1)
C2 – C1 – N1 – C11	1.1(2)	C7 – C8 – C9 – C10	0.9(2)
O1 – C1 – C2 – C3	-0.1(2)	C5 – C10 – C9 – C8	-0.1(2)
N1 – C1 – C2 – C3	-179.8(1)	C5 – C6 – C7 – C8	-1.3(2)
C1 – C2 – C3 – C4	-139.4(1)	C9 – C8 – C7 – C6	-0.2(2)
C2 – C3 – C4 – C5	-174.6(1)	C1 – N1 – C14 – C13	-121.1(1)
C10 – C5 – C4 – C3	-165.2(1)	C11 – N1 – C14 – C13	53.7(1)
C6 – C5 – C4 – C3	15.5(2)	C13 – O2 – C12 – C11	-60.5(1)
C10 – C5 – C6 – C7	2.0(2)	N1 – C11 – C12 – O2	57.2(1)
C4 – C5 – C6 – C7	-178.7(1)	C12 – O2 – C13 – C14	60.3(1)
C1 – N1 – C11 – C12	119.8(1)	N1 – C14 – C13 – O2	-56.0(1)

Single crystals of compound **11d**, suitable for X-ray diffraction, were obtained by slow evaporation of **Et_2O** solution. The crystals were introduced into perfluorinated oil and a suitable single crystal was carefully mounted on the top of a thin glass wire. Data collection was performed with an Oxford Xcalibur 3 diffractometer equipped with a Spellman generator (50 kV, 40 mA) and a Kappa CCD detector, operating with Mo-K_α radiation (λ = 0.71071 Ǻ).
Data collection and data reduction were performed with the CrysAlisPro software.[8] Absorption correction using the multiscan method[8] was applied. The structures were solved with SHELXS-97,[9] refined with SHELXL-97[10] and finally checked using PLATON.[11] Details for data collection and structure refinement are summarized in Table 1.
CCDC-**2164154** contains supplementary crystallographic data for this compound. These data can be obtained free of charge from The Cambridge Crystallographic Data Centre *via* www.ccdc.cam.ac.uk/data_request/cif.

Table 13. Details for X-ray data collection and structure refinement for compound **11d**.

	**11d**
Empirical formula	$C_{17}H_{20}INO_2$
Formula mass	397.24
T[K]	123(2)
Crystal size [mm]	0.35 × 0.25 × 0.10
Crystal description	yellow block
Crystal system	monoclinic
Space group	*P*21/*c*
a [Å]	13.0951(7)
b [Å]	8.9157(6)
c [Å]	14.5136(9)
α [°]	90.0
β [°]	110.787(7)
γ [°]	90.0
V [$Å^3$]	1584.19(18)
Z	4
$\rho_{calcd.}$ [g cm^{-3}]	1.666
μ [mm^{-1}]	2.026
F(000)	792
Θ range [°]	2.83 – 25.24
Index ranges	$-17 \leq h \leq 17$
	$-11 \leq k \leq 11$
	$-19 \leq l \leq 19$
Reflns. collected	26840
Reflns. obsd.	3137
Reflns. unique	3914 (R_{int} = 0.0430)
R_1, wR_2 (2σ data)	0.0274, 0.0612
R_1, wR_2 (all data)	0.0412, 0.0671
GOOF on F^2	1.034
Peak/hole [e $Å^{-3}$]	0.824 / -0.460

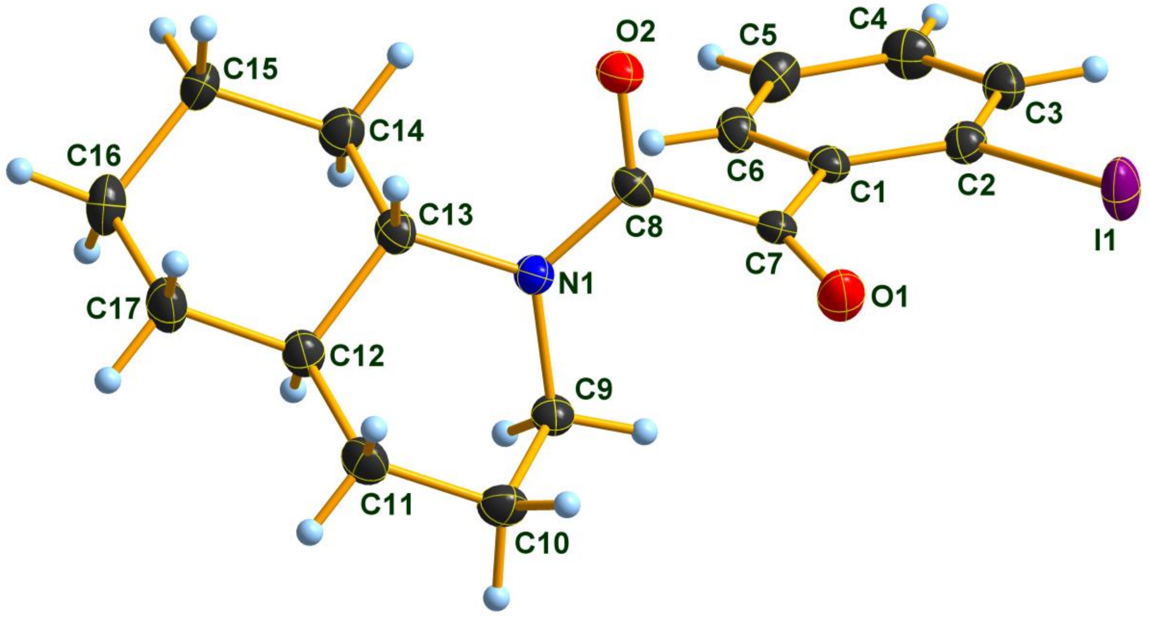

Figure 4. Molecular structure of compound **11d** in the crystal. DIAMOND[12] representation; thermal ellipsoids are drawn at 50 % probability level.

Table 14. Selected bond lengths (Å) of compound **11d**.

I1 – C2	2.103(2)	C12 – C17	1.527(3)
O1 – C7	1.207(3)	C11 – C10	1.539(3)
N1 – C8	1.334(3)	C14 – C15	1.536(3)
N1 – C9	1.465(3)	C9 – C10	1.527(3)
N1 – C13	1.483(3)	C17 – C16	1.525(4)
C1 – C6	1.397(3)	C16 – C15	1.531(4)
C1 – C2	1.411(3)	C5 – C4	1.381(3)
C1 – C7	1.494(3)	C13 – C14	1.527(3)
O2 – C8	1.227(3)	C13 – C12	1.529(3)
C2 – C3	1.388(3)	C7 – C8	1.532(3)
C3 – C4	1.386(4)	C12 – C11	1.513(3)
C6 – C5	1.378(3)		

Table 15. Selected bond angles (°) of compound **11d**.

C8 – N1 – C9	124.7(2)	C13 – C14 – C15	111.0(2)
C8 – N1 – C13	118.4(2)	N1 – C9 – C10	109.9(2)
C9 – N1 – C13	116.7(2)	C16 – C17 – C12	111.4(2)
C6 – C1 – C2	117.8(2)	C17 – C16 – C15	111.9(2)

C6 – C1 – C7	117.3(2)	C16 – C15 – C14	111.9(2)
C2 – C1 – C7	124.9(2)	C9 – C10 – C11	111.6(2)
C3 – C2 – C1	120.0(2)	O1 – C7 – C1	125.0(2)
C3 – C2 – I1	116.4(2)	O1 – C7 – C8	120.5(2)
C1 – C2 – I1	123.7(2)	C1 – C7 – C8	114.2(2)
C4 – C3 – C2	120.6(2)	O2 – C8 – N1	125.4(2)
C5 – C6 – C1	121.9(2)	O2 – C8 – C7	115.1(2)
C6 – C5 – C4	119.5(2)	N1 – C8 – C7	119.4(2)
C5 – C4 – C3	120.1(2)	C11 – C12 – C17	114.4(2)
N1 – C13 – C14	112.4(2)	C11 – C12 – C13	111.0(2)
N1 – C13 – C12	110.1(2)	C17 – C12 – C13	107.8(2)
C14 – C13 – C12	110.5(2)	C12 – C11 – C10	112.2(2)

Table 16. Selected torsion angles (°) of compound **11d**.

C6 – C1 – C2 – C3	1.7(3)	C13 – N1 – C8 – C7	-177.8(2)
C7 – C1 – C2 – C3	178.4(2)	O1 – C7 – C8 – O2	100.9(3)
C6 – C1 – C2 – I1	-179.4(2)	C1 – C7 – C8 – O2	-73.3(3)
C7 – C1 – C2 – I1	-2.6(3)	O1 – C7 – C8 – N1	-79.0(3)
C1 – C2 – C3 – C4	-2.0(4)	C1 – C7 – C8 – N1	106.8(2)
I1 – C2 – C3 – C4	179.0(2)	N1 – C13 – C12 – C11	47.4(3)
C2 – C1 – C6 – C5	0.0(3)	C14 – C13 – C12 – C11	172.1(2)
C7 – C1 – C6 – C5	-177.0(2)	N1 – C13 – C12 – C17	173.4(2)
C1 – C6 – C5 – C4	-1.4(4)	C14 – C13 – C12 – C17	-61.9(3)
C6 – C5 – C4 – C3	1.1(4)	C17 – C12 – C11 – C10	180.0(2)
C2 – C3 – C4 – C5	0.6(4)	C13 – C12 – C11 – C10	-57.8(3)
C8 – N1 – C13 – C14	71.2(3)	N1 – C13 – C14 – C15	-178.2(2)
C9 – N1 – C13 – C14	-113.0(2)	C12 – C13 – C14 – C15	58.4(3)
C8 – N1 – C13 – C12	-165.2(2)	C8 – N1 – C9 – C10	116.6(2)
C9 – N1 – C13 – C12	10.6(3)	C13 – N1 – C9 – C10	-58.9(3)
C6 – C1 – C7 – O1	157.8(2)	C11 – C12 – C17 – C16	-175.7(2)
C2 – C1 – C7 – O1	-19.0(4)	C13 – C12 – C17 – C16	60.3(3)
C6 – C1 – C7 – C8	-28.3(3)	C12 – C17 – C16 – C15	-55.4(3)
C2 – C1 – C7 – C8	154.9(2)	C17 – C16 – C15 – C14	50.4(3)
C9 – N1 – C8 – O2	-173.1(2)	C13 – C14 – C15 – C16	-51.9(3)
C13 – N1 – C8 – O2	2.3(3)	N1 – C9 – C10 – C11	46.2(3)
C9 – N1 – C8 – C7	6.8(3)	C12 – C11 – C10 – C9	9.5(3)

Single crystals of compound **13g**, suitable for X-ray diffraction, were obtained by slow evaporation of **$CDCl_3$** solution. The crystals were introduced into perfluorinated oil and a suitable single crystal was carefully mounted on the top of a thin glass wire. Data collection was performed with an Oxford Xcalibur 3 diffractometer equipped with a Spellman generator (50 kV, 40 mA) and a Kappa CCD detector, operating with Mo-K_α radiation (λ = 0.71071 Å).

Data collection and data reduction were performed with the CrysAlisPro software.[8] Absorption correction using the multiscan method[8] was applied. The structures were solved with SHELXS-97,[9] refined with SHELXL-97[10] and finally checked using PLATON.[11] Details for data collection and structure refinement are summarized in Table 1.

CCDC-**2164156** contains supplementary crystallographic data for this compound. These data can be obtained free of charge from The Cambridge Crystallographic Data Centre *via* www.ccdc.cam.ac.uk/data_request/cif.

Table 17. Details for X-ray data collection and structure refinement for compound **13g**.

	**13g**
Empirical formula	$C_{21}H_{27}NO_3$
Formula mass	341.43
T[K]	123(2)
Crystal size [mm]	0.30 × 0.18 × 0.07
Crystal description	colorless block
Crystal system	monoclinic
Space group	*P*21/*n*
a [Å]	8.4082(3)
b [Å]	9.9782(4)
c [Å]	22.7287(8)
α [°]	90.0
β [°]	94.143(3)
γ [°]	90.0
V [Å^3]	1901.93(12)
Z	4
$\rho_{calcd.}$ [g cm^{-3}]	1.192
μ [mm^{-1}]	0.079
F(000)	736
Θ range [°]	2.23 – 25.24
Index ranges	$-10 \le h \le 10$
	$-12 \le k \le 12$
	$-29 \le l \le 29$
Reflns. collected	30212
Reflns. obsd.	3245
Reflns. unique	4182 (R_{int} = 0.0420)
R_1, wR_2 (2σ data)	0.0441, 0.1020
R_1, wR_2 (all data)	0.0610, 0.1114
GOOF on F^2	1.042
Peak/hole [e Å^{-3}]	0.257 / -0.198

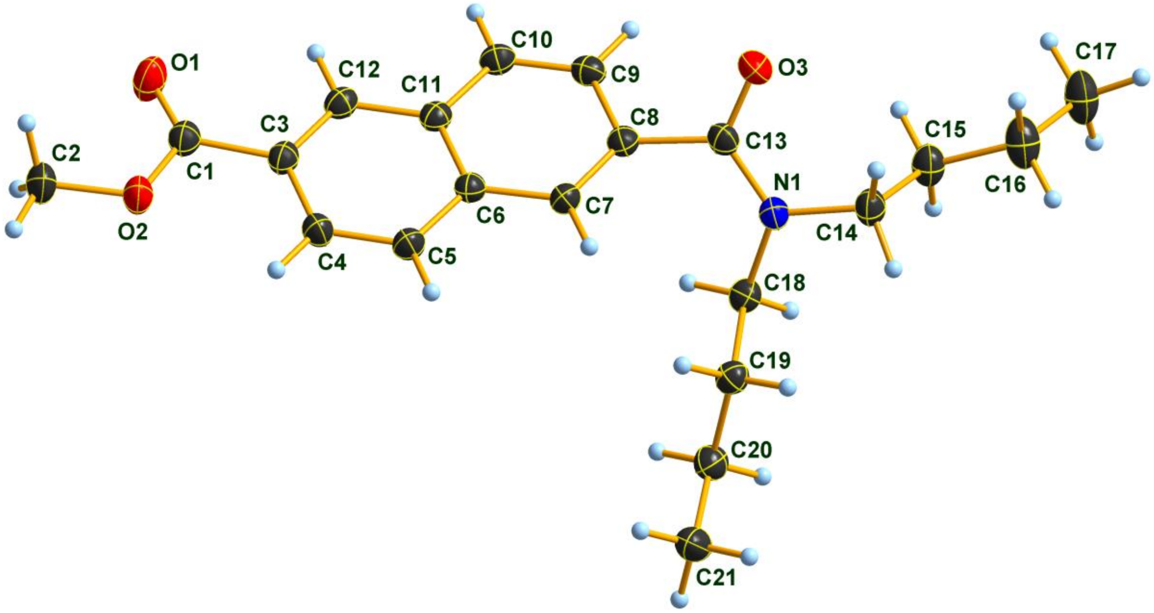

Figure 5. Molecular structure of compound **13g** in the crystal. DIAMOND[12] representation; thermal ellipsoids are drawn at 50 % probability level.

Table 18. Selected bond lengths (Å) of compound **13g**.

C6 – C5	1.420(2)	C9 – C10	1.366(2)
C6 – C11	1.425(2)	C15 – C14	1.520(2)
C6 – C7	1.425(2)	C15 – C16	1.528(2)
C12 – C3	1.376(2)	C16 – C17	1.513(2)
C12 – C11	1.420(2)	C1 – O1	1.205(2)
C13 – O3	1.239(2)	C8 – C9	1.422(2)
C13 – N1	1.344(2)	C20 – C19	1.522(2)
C13 – C8	1.510(2)	C20 – C21	1.525(2)
N1 – C18	1.467(2)	C11 – C10	1.422(2)
N1 – C14	1.472(2)	C18 – C19	1.523(2)
C5 – C4	1.367(2)	C7 – C8	1.369(2)
C3 – C4	1.420(2)	O2 – C1	1.338(2)
C3 – C1	1.495(2)	O2 – C2	1.445(2)

Table 19. Selected bond angles (°) of compound **13g**.

C5 – C6 – C11	119.1(1)	C5 – C4 – C3	120.1(1)
C5 – C6 – C7	121.7(1)	C14 – C15 – C16	111.4(1)

C11 – C6 – C7	119.2(1)	C20 – C19 – C18	111.6(1)
C3 – C12 – C11	120.4(1)	N1 – C14 – C15	113.3(1)
O3 – C13 – N1	122.0(1)	C17 – C16 – C15	113.1(1)
O3 – C13 – C8	117.3(1)	N1 – C18 – C19	114.0(1)
N1 – C13 – C8	120.7(1)	C8 – C7 – C6	120.6(1)
C13 – N1 – C18	125.1(1)	C1 – O2 – C2	116.2(1)
C13 – N1 – C14	117.3(1)	O1 – C1 – O2	123.4(1)
C18 – N1 – C14	117.0(1)	O1 – C1 – C3	125.1(1)
C4 – C5 – C6	120.8(1)	O2 – C1 – C3	111.5(1)
C12 – C3 – C4	120.5(1)	C7 – C8 – C9	120.0(1)
C12 – C3 – C1	118.5(1)	C7 – C8 – C13	122.4(1)
C4 – C3 – C1	120.9(1)	C9 – C8 – C13	116.9(1)
C12 – C11 – C10	122.2(1)	C19 – C20 – C21	111.9(1)
C12 – C11 – C6	119.0(1)	C10 – C9 – C8	120.7(1)
C10 – C11 – C6	118.8(1)	C9 – C10 – C11	120.7(1)

Table 20. Selected torsion angles (°) of compound **13g**.

O3 – C13 – N1 – C18	-169.5(1)	C12 – C3 – C1 – O2	-177.7(1)
C8 – C13 – N1 – C18	11.4(2)	C4 – C3 – C1 – O2	1.3(2)
O3 – C13 – N1 – C14	1.8(2)	C6 – C7 – C8 – C9	-1.1(2)
C8 – C13 – N1 – C14	-177.3(1)	C6 – C7 – C8 – C13	168.7(1)
C11 – C6 – C5 – C4	-0.8(2)	O3 – C13 – C8 – C7	-118.4(2)
C7 – C6 – C5 – C4	177.3(1)	N1 – C13 – C8 – C7	60.7(2)
C11 – C12 – C3 – C4	-1.9(2)	O3 – C13 – C8 – C9	51.7(2)
C11 – C12 – C3 – C1	177.1(1)	N1 – C13 – C8 – C9	-129.2(1)
C3 – C12 – C11 – C10	-178.0(1)	C7 – C8 – C9 – C10	-0.1(2)
C3 – C12 – C11 – C6	0.4(2)	C13 – C8 – C9 – C10	-170.4(1)
C5 – C6 – C11 – C12	1.0(2)	C8 – C9 – C10 – C11	1.8(2)
C7 – C6 – C11 – C12	-177.2(1)	C12 – C11 – C10 – C9	175.9(1)
C5 – C6 – C11 – C10	179.4(1)	C6 – C11 – C10 – C9	-2.4(2)
C7 – C6 – C11 – C10	1.3(2)	C6 – C5 – C4 – C3	-0.7(2)
C13 – N1 – C18 – C19	-110.1(2)	C12 – C3 – C4 – C5	2.0(2)
C14 – N1 – C18 – C19	78.7(2)	C1 – C3 – C4 – C5	-176.9(1)
C5 – C6 – C7 – C8	-177.6(1)	C21 – C20 – C19 – C18	-177.2(1)
C11 – C6 – C7 – C8	0.5(2)	N1 – C18 – C19 – C20	-174.4(1)
C2 – O2 – C1 – O1	1.7(2)	C13 – N1 – C14 – C15	-83.6(2)

C2 – O2 – C1 – C3	-179.2(1)	C18 – N1 – C14 – C15	88.4(2)
C12 – C3 – C1 – O1	1.4(2)	C16 – C15 – C14 – N1	168.3(1)
C4 – C3 – C1 – O1	-179.6(1)	C14 – C15 – C16 – C17	-177.8(2)

References

1. A. Krasovskiy, P. Knochel, *Angew. Chem. Int. Ed. Engl.* **2004**, *43*, 3333.
2. P. Knochel, M. C. P. Yeh, S. C. Berk, J. Talbert, *J. Org. Chem.* **1988**, *53*, 2390.
3. A. Krasovskiy, V. Krasovskaya, P. Knochel, *Angew. Chem. Int. Ed.* **2006**, *45*, 2958-2961.
4. R. A. Olofson, C. M. Dougherty, *J. Am. Chem. Soc.* **1973**, *95*, 582-584; M. Campbell, V. Snieckus, E. W. Baxter *Encyclopedia of Reagents for Organic Synthesis*, Wiley, **2001**.
5. S. H. Wunderlich, P. Knochel, *Angew. Chem. Int. Ed.* **2007**, *46*, 7685.
6. P. Knochel, A. Krasovskiy, *Synthesis* **2006**, *5*, 890.
7. Z. Blum, K. Nyberg, *Acta Chem. Scand. Ser. B* **1981**, *35*, 743.
8. Program package 'CrysAlisPro 1.171.40.84a (Rigaku OD, 2020)'.
9. Sheldrick, G. M. (1997) SHELXS-97: *Program for Crystal Structure Solution*, University of Göttingen, Germany.
10. Sheldrick, G. M. (1997) SHELXL-97: *Program for the Refinement of Crystal Structures*, University of Göttingen, Germany.
11. Spek, A. L. (1999) PLATON: *A Multipurpose Crystallographic Tool*, Utrecht University, Utrecht, The Netherlands.
12. DIAMOND, Crystal Impact GbR., Version 3.2i.
